# Paracrystalline IrCoO_*x*_ stabilization by rutile SnO_2_ towards durable acidic oxygen evolution

**DOI:** 10.1038/s41467-026-76919-0

**Published:** 2026-08-21

**Authors:** Han Tian, Ziyi Yu, Hongchang Hao, Rui Deng, Wenshu Luo, Xu Yu, Han Wu, Xiangzhi Cui, Jianlin Shi

**Affiliations:** 1 State Key Laboratory of High Performance Ceramics, Shanghai Institute of Ceramics, Chinese Academy of Sciences, Shanghai, 200050 China; 2 Materials Science and Engineering Program & Texas Materials Institute, The University of Texas at Austin, Austin, TX 78712 USA; 3 Center of Materials Science and Optoelectronics Engineering, University of Chinese Academy of Sciences, Beijing, 100049 China; 4 School of Chemistry and Materials Science, Hangzhou Institute for Advanced Study, University of Chinese Academy of Sciences, Hangzhou, 310024 China

**Keywords:** Electrocatalysis, Electrocatalysis, Electrocatalysis, Hydrogen energy

## Abstract

The development of oxygen evolution reaction catalysts featuring high activity with long-term durability in acidic media remains a major challenge for proton exchange membrane water electrolysis. Herein, we report a strategy to stabilize highly active paracrystalline IrCoO_*x*_ phase by integrating it with rutile SnO_2_, achieving an optimal activity-durability balance. The formation of paracrystalline phase, directly visualized by in-situ heating transmission electron microscopy, enhances lattice oxygen activation and oxygen evolution kinetics. Owing to their shared P4_2_/mnm space group, the resulting IrCoO_*x*_−2@SnO_2_ catalyst demonstrates a mass activity of 1192 A g_Ir_^−1^ (114 times that of IrO_2_), and a durable operation for 3880 h at 2 A cm^−2^ in electrolysis cell, representing a very competitive stability under the identical conditions. The SnO_2_ was proven to stabilize the paracrystalline phase by mitigating metal dissolution, agglomeration, and Ir over-oxidation, while favorable interfacial water dynamics enables reversible oxygen species cycling and efficient proton transfer, collectively contributing to the satisfactory durability.

## Abstract (web summary)

Developing acidic catalysts featuring high oxygen evolution activity with long durability bring promise but a challenge for hydrogen production. Here, the authors report a strategy to stabilize active paracrystalline phase with same-space-group SnO_2_, achieving an optimal activity-durability balance.

## Introduction

Proton exchange membrane water electrolysis (PEMWE) is a key technology for producing high-purity hydrogen from renewable yet intermittent energy sources, aligning with global decarbonization goals^1,2^. Its advantages include high operational current density, rapid response, and exceptional gas purity^3–5^. However, the harsh acidic and oxidizing conditions at the anode pose severe challenges for the anodic oxygen evolution reaction (OER), which contributes substantially to the overall energy losses^6,7^. Although iridium oxide (IrO_2_) remains the state-of-the-art OER catalyst under these conditions, its scarcity and high cost limit the large-scale development of PEMWE systems^8,9^. Moreover, the commercial IrO_2_ suffers from moderate intrinsic activity, motivating the development of modified IrO_2_-based catalysts with enhanced performance and reduced noble metal usage^10,11^.

Amorphous or disordered catalysts have attracted considerable interest due to their flexible coordination environments and abundant unsaturated sites, which often lead to improved OER activities^12–15^. In particular, structural disorder can activate the lattice oxygen mechanism (LOM)^16^, offering a pathway to surpass the activity limitations of conventional adsorption evolution mechanism (AEM)-based oxides^17,18^. Generally, introducing a secondary metal such as Co or Ni is a feasible approach to achieve both the amorphization of IrO_*x*_-based catalysts and reduced usage of Ir^19,20^. For instance, Hyung-Suk Oh et al. developed carbon-supported IrCoO_*x*_ core-shell nanoparticles exhibiting efficient OER activity and stability for 216 h in neutral tap water. Nevertheless, amorphous materials still suffer from poor electrochemical and structural stability under acidic OER conditions^21,22^. Key degradation pathways include irreversible over-oxidation of active sites, metal ion dissolution, nanoparticle agglomeration, and recrystallization during operation^23–25^. These issues create a critical trade-off between high OER activity and long-term stability, which has hindered the application of amorphous Ir-based oxides in practical PEMWE systems.

Constructing a coherent interface between oxides of similar crystal structure offers low interfacial energy and suppressed interdiffusion kinetics, thereby enhancing both thermodynamic and kinetic stability^26,27^. Inspired by this, we hypothesized that a rutile-type oxide support could stabilize an active Ir-based paracrystalline phase—a state with short-to-medium-range order^28,29^—by forming a structurally matched interface. Tin oxide (SnO_2_), a typical rutile-type oxide, has recently been recognized as a suitable corrosion-stable substrate material under PEM conditions^30,31^. Bein et al. have reported that the interconnected, ultrasmall IrO_2_ crystallites epitaxially growing along the isostructural SnO_2_ substrate enable excellent conductivity and stability. However, the doped amorphous IrO_*x*_-based material stably anchored in SnO_2_ support in acidic OER still remains unexplored. Such a support is also expected to reasonably regulate the local reaction environment, such as interfacial water structure and proton transfer, which are critical for OER kinetics and catalyst durability, especially under LOM operation^32,33^.

**Fig. 1:**
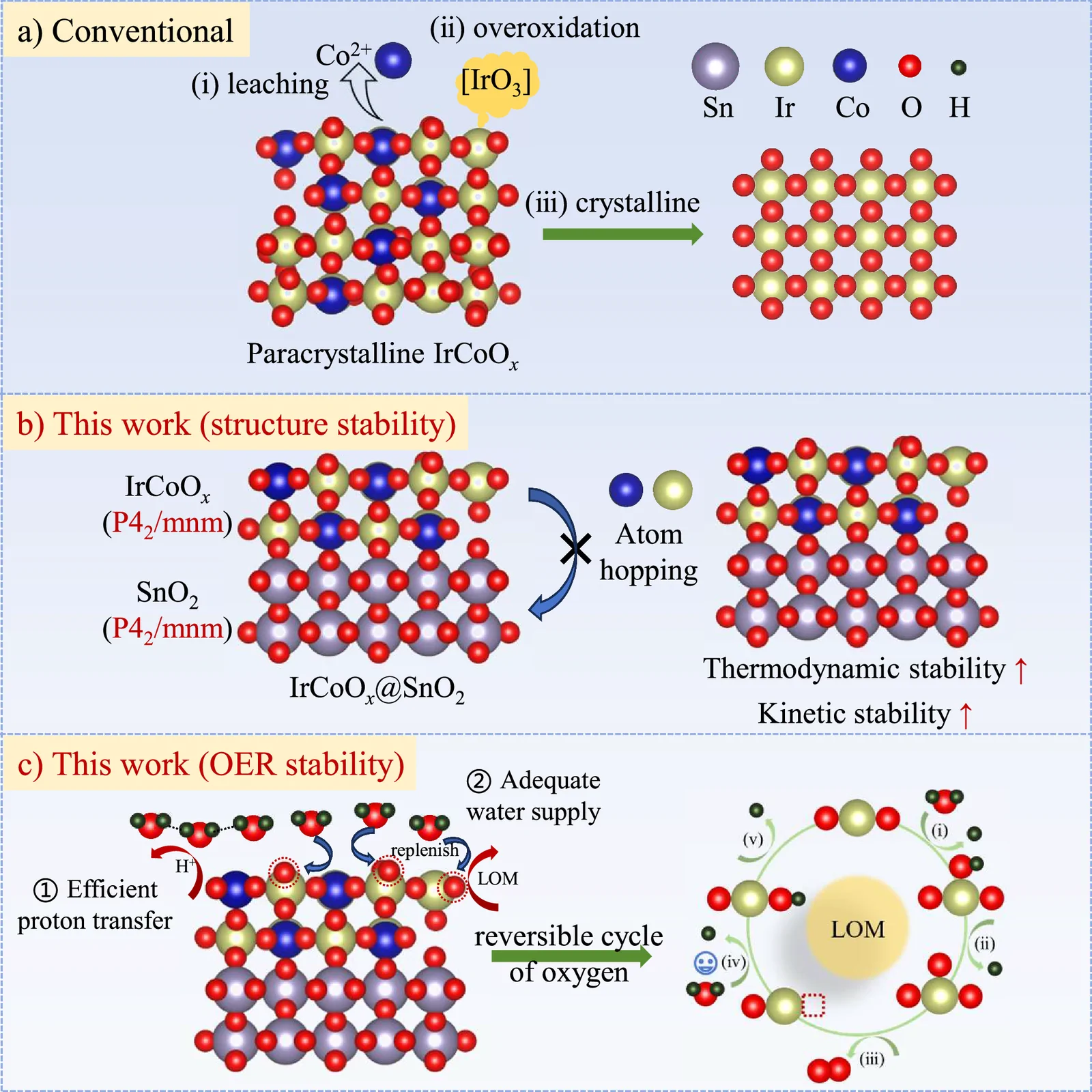
Schematic illustration. The comparison of the structural stability during OER between (**a**) an unsupported paracrystalline IrCoO_*x*_ catalyst and (**b**, **c**) the SnO_2_ stabilized paracrystalline IrCoO_*x*_ catalyst presented in this work. The SnO_2_ framework enhances stability by inhibiting over-oxidation, suppressing particle agglomeration, mitigating Ir/Co dissolution and maintaining reversible oxygen cycle (H_2_O→lattice oxygen→O_2_) during OER.

In this work, we designed and synthesized a paracrystalline IrCoO_*x*_ phase (previously vaguely described as an amorphous phase) on corrosion-stable SnO_2_ with the identical space group of P4_2_/mnm. Using in-situ heating transmission electron microscopy (TEM), we directly observed the formation of this paracrystalline structure. The resulting IrCoO_*x*_−2@SnO_2_ catalyst exhibits competitive OER performance in 0.5 M H_2_SO_4_, with a mass activity 114 times higher than that of commercial IrO_2_ (1192 A g_Ir_^−1^ vs. 10.44 A g_Ir_^−1^), and the satisfactory stability featuring continuous operation for 3880 h at 2 A cm^−2^ in a PEMWE cell using the catalyst as anode. As illustrated in Fig. 1, the rutile SnO_2_ support not only stabilizes the paracrystalline IrCoO_*x*_ phase by suppressing metal dissolution, particle agglomeration, and Ir over-oxidation, but also promotes favorable interfacial water management, enabling reversible cycling of oxygen and efficient proton transfer during OER.

## Results

### Catalyst synthesis and structural analysis

**Fig. 2:**
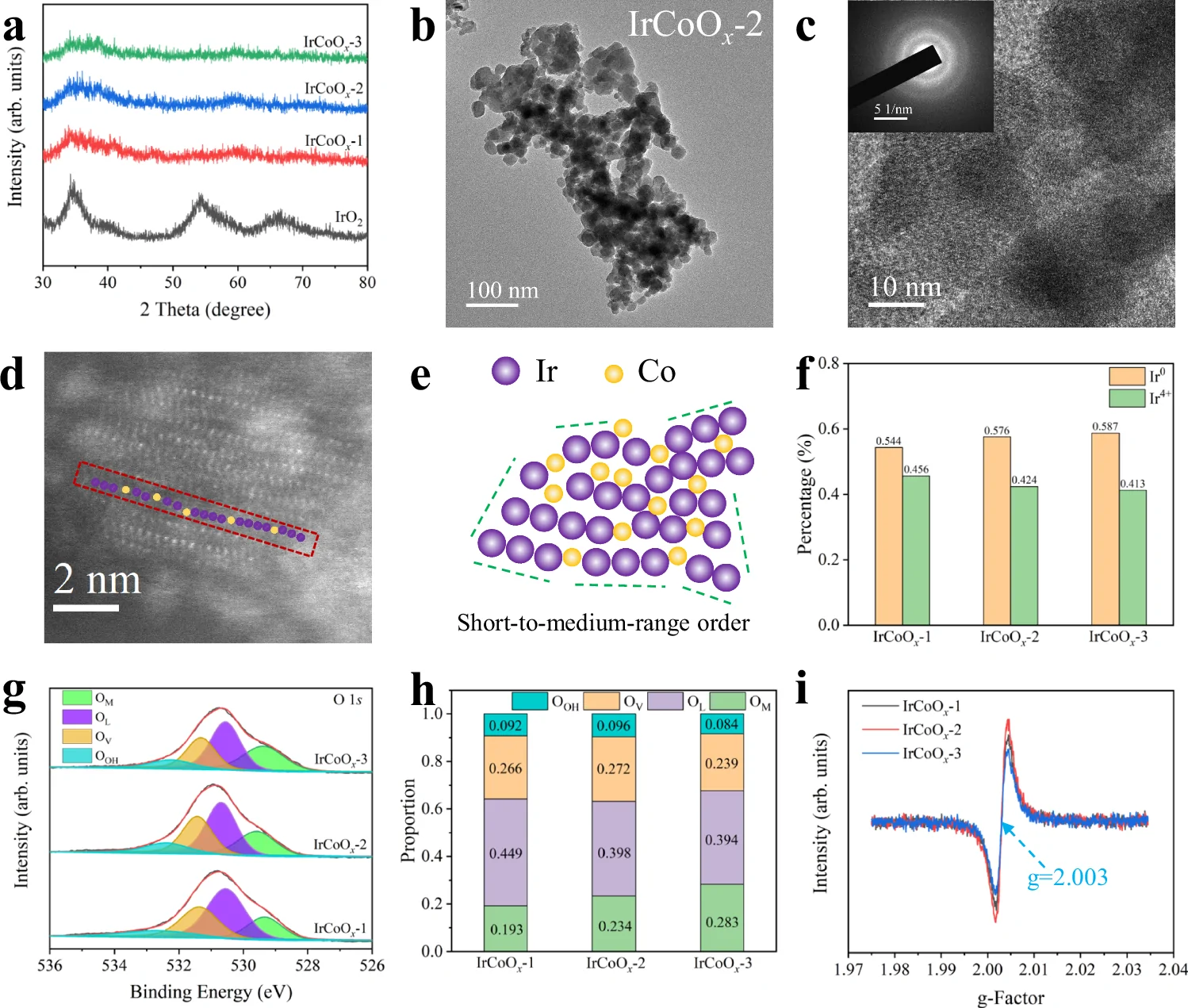
Structural characterization of paracrystalline IrCoO_*x*_. **a** XRD patterns for IrCoO_*x*_ samples with various Ir:Co ratio. **b** TEM, **c** HRTEM (inset: SAED pattern), and **d** the atomic-scale HAADF-STEM images of IrCoO_*x*_-2 sample. **e** The schematic diagram of paracrystalline IrCoO_*x*_. **f** The histograms percentages of Ir^0^ and Ir^4+^, **g** XPS O 1 *s* spectra, **h** the proportion of O_OH_, O_V_, O_L_, O_M_ species, and **i** ESR results for IrCoO_*x*_-1, IrCoO_*x*_-2 and IrCoO_*x*_-3.

Addressing the urgent challenge of the high solubility and poor electrochemical stability inherent to low-crystallinity amorphous catalysts, we combined paracrystalline IrCoO_*x*_ and commercial SnO_2_ by simple ball milling in this work. Firstly, the paracrystalline IrCoO_*x*_ catalysts were synthesized via a modified one-step molten salt method (the method is described in detail in the Methods section), wherein the high miscibility of Ir and Co source enables the formation of an unusual paracrystalline IrCoO_*x*_ phase. The degrees of structural disorder for IrCoO_*x*_−1, IrCoO_*x*_−2 and IrCoO_*x*_−3 could be controllably regulated by adjusting the relative amounts of IrCl_3_ and CoCl_2_ · 6H_2_O (see more experimental details in the Methods section). As shown in Fig. 2a, the synthesized IrCoO_*x*_ samples all exhibit broad and featureless X-ray diffraction (XRD) peaks, indicative of a non-crystalline structure (Fig. S1). From the TEM image in Fig. 2b, IrCoO_*x*_-2 consists of many nanoparticles (about 10-20 nm). No obvious lattice fringe can be found in the high-resolution TEM (HRTEM) image (Fig. 2c). Further, the selected area electron diffraction (SAED) in the inset of Fig. 2c shows the uniform diffraction ring pattern without any diffraction spot, proving the paracrystalline phase of as-prepared IrCoO_*x*_.

Given the inherent sensitivity of paracrystalline or amorphous structures to electron beams, atomic-scale imaging requires minimized electron exposure^34,35^. By employing low electron currents and applying the Fourier filter, we successfully obtained the atomic-scale high-angle annular dark field scanning TEM (HAADF-STEM) image (Fig. 2d, e) that reveals a distorted lattice arrangement, providing direct visualization of the short-to-medium-range order characteristic of the paracrystalline phase. After the incorporation of SnO_2_, the absence of lattice fringe proves the maintenance of IrCoO_*x*_-2 paracrystalline phase (Figs. S2–S4).

The calculated Ir^4+^/Ir^0^ ratio in X-ray photoelectron spectroscopy (XPS, Table S1) decreases progressively with Co incorporation (Figs. 2f and S5), a modulation that is anticipated to enhance the catalyst’s operational stability during OER^36,37^. Analysis of the O 1 *s* spectra (Figs. 2g, 2h) reveals four constituent peaks corresponding to metal oxygen (O_M_), lattice oxygen (O_L_), oxygen vacancy (O_V_) and adsorbed OH (O_OH_). Here, O_M_ and O_L_ represent oxygen atoms directly bonded to metal ions in a disordered configuration and oxygen in a periodic crystalline lattice, respectively^38^. To quantitatively describe the structural disorder (Table S2), we introduce an amorphous factor (AF), defined as AF=Area (O_M_)/(Area (O_M_)+Area (O_L_)). The calculated AF value shows a clear increasing trend with Co doping (0.3 → 0.37 → 0.42), confirming the role of Co in promoting paracrystalline formation. IrCoO_*x*_-2 exhibits the most intense signal at g = 2.003 from Electron Spin Resonance (ESR) spectroscopy (Fig. 2i), indicative of the highest concentration of oxygen vacancies, validating the reliability of the XPS peak deconvolution. Moreover, a moderate reduction in the AF value (0.37 → 0.28) was reasonably observed for IrCoO_*x*_-2@SnO_2_ (Figs. S6 and S7), attributable to the increased O_L_ content owing to the contribution of crystalline SnO_2_.

### Evolution mechanism for paracrystalline structure

Control experiments with Fe, Mn, Ni, and Cu were conducted to probe the paracrystalline formation mechanism. Unlike the featureless patterns of IrCoO_*x*_, all control samples (IrFeO_*x*_, IrMnO_*x*_, IrNiO_*x*_, and IrCuO_*x*_) showed sharp crystalline diffraction peaks (Fig. S8a–d), highlighting the unique nature of the IrCoO_*x*_ system and motivating the detailed investigation presented herein.

Under the molten state at 360 °C, NaOH serves two purposes:

Firstly, NaOH can react with IrCl_3_ and CoCl_2_ · 6H_2_O following the equations:

1 $${{{\rm{IrCl}}}}_{3}+3{{\rm{NaOH}}}\to {{{\rm{Ir}}}({{\rm{OH}}})}_{3}+3{{\rm{NaCl}}}$$

2 $$\begin{array}{ccc}{2{{\rm{Ir}}}({{\rm{OH}}})}_{3}\to {{{\rm{Ir}}}}_{2}{{{\rm{O}}}}_{3}+{3{{\rm{H}}}}_{2}{{\rm{O}}} & {{\rm{or}}} & {{{\rm{IrO}}}}_{2}+{{{\rm{H}}}}_{2}{{\rm{O}}}\end{array}$$

3 $${{\mathrm{CoCl}}}_{2}\cdot {6{{\rm{H}}}}_{2}{{\rm{O}}}\to {{\mathrm{CoCl}}}_{2}+{6{{\rm{H}}}}_{2}{{\rm{O}}}\left( > 86^\circ {{\rm{C}}}\right)$$

4 $${{{\rm{CoCl}}}}_{2}+2{{\rm{NaOH}}}\to {{{\rm{Co}}}({{\rm{OH}}})}_{2}+2{{\rm{NaCl}}}$$

5 $${{{\rm{Co}}}({{\rm{OH}}})}_{2}\to {{\rm{CoO}}}+{{{\rm{H}}}}_{2}{{\rm{O}}}$$

Secondly, molten NaOH acts as a solvent to thoroughly mix the IrO_*x*_ and CoO_*x*_ precursors, enabling their transformation into paracrystalline IrCoO_*x*_. Whereas the IrO_*x*_ sample is highly amorphous (Figs. S9–S11), the incorporation of Co induces a slight increase in structural ordering level (i.e., paracrystallinity), as evidenced by the appearance of more featured XRD peaks. Meanwhile, the diffraction peaks shift to higher angles due to the lattice contraction due to Co incorporation, and the peak broadening reflects the retained paracrystalline nature.

**Fig. 3:**
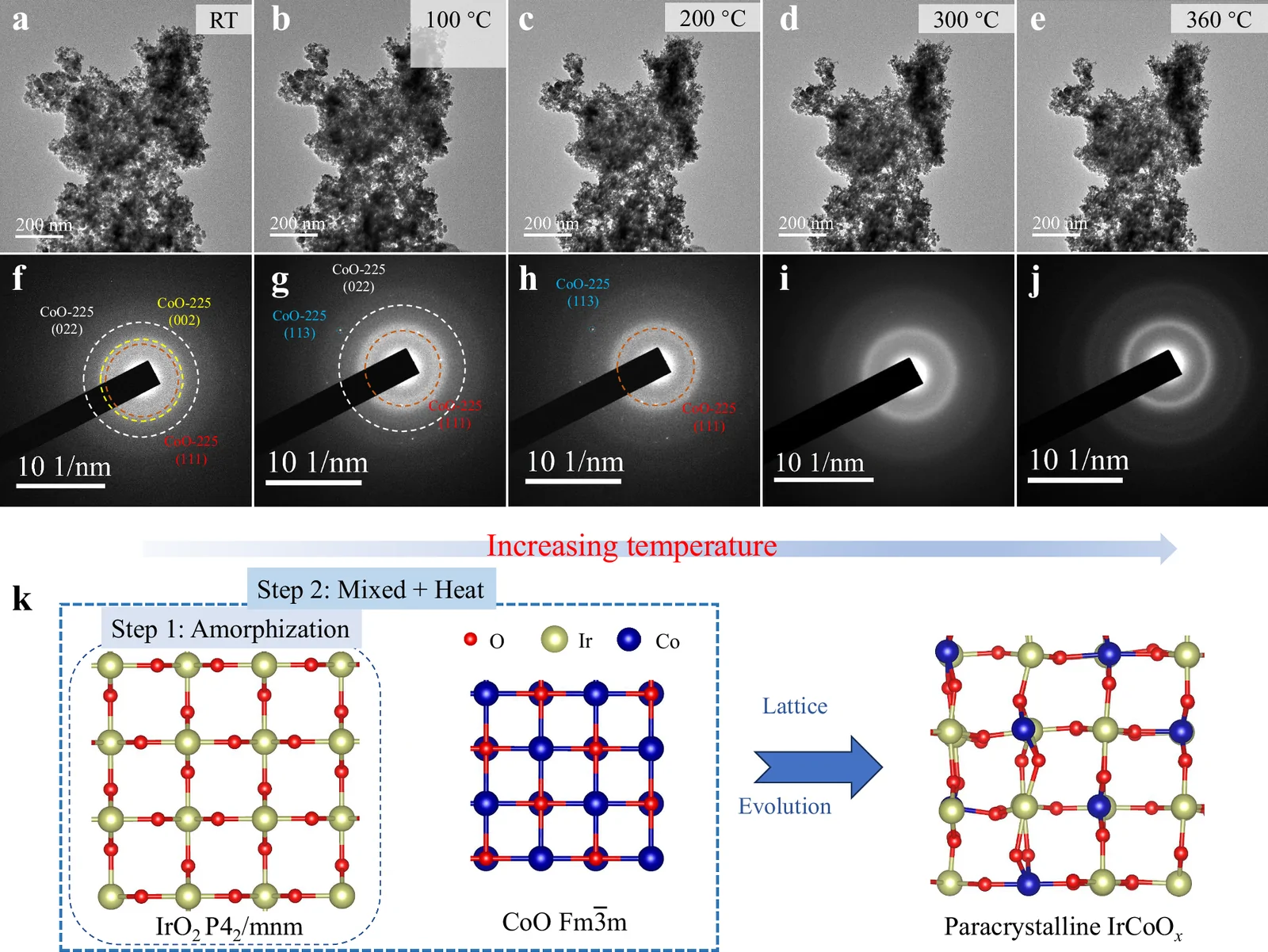
In-situ heating TEM analysis for the lattice evolution of paracrystalline IrCoO_*x*_. TEM images and the corresponding SAED patterns of IrO_*x*_ and CoO_*x*_ mixture at (**a**, **f**) RT, (**b**, **g**) 100 °C, (**c**, **h**) 200 °C, (**d**, **i**) 300 °C and (**e**, **j**) 360 °C. **k** The schematic illustration for the evolution of the paracrystalline IrCoO_*x*_ structure^49^.

To gain deeper insight into the formation mechanism of paracrystalline IrCoO_*x*_, the in-situ heating TEM measurements were conducted on a heating holder by a SmartSet Hot Stage controller (Gatan Model 901) from room temperature (RT) to 360 °C^39,40^. TEM images (Fig. 3a–e) revealed no significant morphological changes for the IrO_*x*_ and CoO_*x*_ mixture apart from a gradual reduction in particle size with increasing temperature. However, the sample exhibits intriguing phase transitions by SAED patterns at various temperatures. At RT, the SAED pattern (Fig. 3f) showed distinct diffraction spots corresponding to (002), (022), and (111) planes of the CoO Fm^3^m phase^41^, superimposed on a diffuse halo from amorphous IrO_*x*_. As the temperature increased, these CoO diffraction spots successively diminished and eventually disappeared (Fig. 3g–i), indicating the miscibility of CoO phase into IrO_*x*_ lattice. At 100 °C, (113) plane of CoO Fm^3^m appeared transiently owing to the lattice transformation and disappeared again at 300 °C. Impressively, the paracrystalline diffraction rings emerge without any diffraction spots as the temperature rises to 360 °C (Fig. 3j), consistent with as-prepared IrCoO_*x*_ sample, firmly confirming the growth process of unique paracrystalline IrCoO_*x*_ (Fig. 3k). All lattice plane changes from RT to 360 °C were quantified in Table S3. The further incorporation of SnO_2_ did not change the diffraction characteristics, which will be discussed in detail in the subsequent section.

### Acidic OER evaluation and water splitting performance

**Fig. 4:**
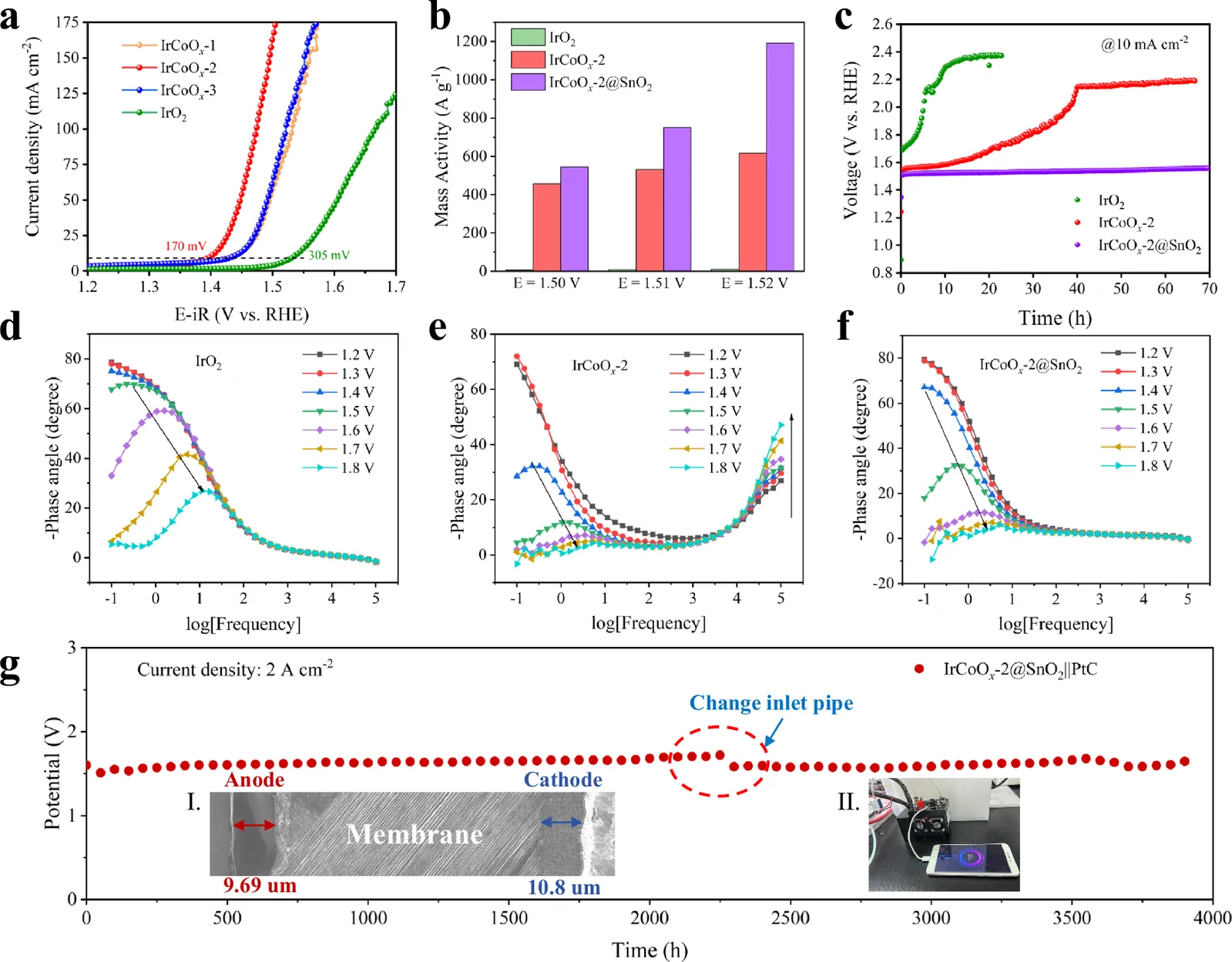
Electrocatalytic OER and PEM water splitting performances. **a** LSV curves of IrCoO_*x*_-1, IrCoO_*x*_-2, IrCoO_*x*_-3 and commercial IrO_2_ catalysts with iR correction (non-iR corrected voltammograms for all catalysts were displayed in Fig. S19; R: 4 ± 0.1 ohm; electrode surface area: 0.196 cm^2^) in 0.5 M H_2_SO_4_ (pH: ~0.3; scan rate: 5 mV s^-1^; temperature: 25 °C). **b** The comparison of mass activities among IrO_2_, IrCoO_*x*_−2 and IrCoO_*x*_-2@SnO_2_ at 1.50, 1.51 and 1.52 V, respectively. **c** The long-term chronopotentiometry curves of IrO_2_, IrCoO_*x*_-2 and IrCoO_*x*_-2@SnO_2_ catalysts. Bode phase plots of the operando EIS for **d** IrO_2_, **e** IrCoO_*x*_-2 and **f** IrCoO_*x*_-2@SnO_2_. **g** The stability tests at 2.0 A cm^-2^ in a PEMWE electrolyzer (reaction temperature: 80 °C) using IrCoO_*x*_-2@SnO_2_ and commercial Pt/C as anode and cathode catalysts with the loading amount of 1.5 mg cm^-2^ and 0.8 mg cm^-2^ (Inset I: Cross-section SEM image of catalyst-coated membrane (CCM) using proton exchange membrane-212; Inset II: The photograph of a mobile phone powered by constructing a water splitting-fuel cell linkage system).

The linear sweep voltammetry (LSV) curves in Fig. 4a reveal that IrCoO_*x*_-2 exhibits the highest OER activity in 0.5 M H_2_SO_4_, requiring only 170 mV (±1) overpotential to reach 10 mA cm^-2^, which is obviously lower than that of commercial IrO_2_ (305 mV), as-prepared IrO_*x*_ (253 mV, Fig. S12) and recently reported catalysts (Table S4). This enhancement is rationalized by its improved reaction kinetics and increased electrochemical active surface area (Figs. S13–S16). After incorporating SnO_2_, the optimal IrCoO_*x*_-2@SnO_2_ catalyst exhibits faster reaction kinetics than IrCoO_*x*_-2 (Figs. S17–S18). Considering the Ir loading as a crucial indicator, mass activity was also evaluated. From Fig. 4b, IrCoO_*x*_-2@SnO_2_ achieves a mass activity of 1192 A g_Ir_^-1^ at 1.52 V, even 114 times higher than that of commercial IrO_2_ (10.44 A g_Ir_^-1^). Moreover, in chronopotentiometry test at 10 mA cm^-2^ (Fig. 4c), IrCoO_*x*_-2@SnO_2_ maintains stability for over 70 h without obvious decay, outperforming both IrCoO_*x*_-2 and IrO_2_. This sustained performance highlights the critical role of SnO_2_ in enhancing catalytic durability.

Analysis of the low-frequency region (Fig. 4d–f) in operando electrochemical impedance spectroscopy (EIS) confirms lower OER onset potentials for IrCoO_*x*_-2 and IrCoO_*x*_-2@SnO_2_ than IrO_2_, consistent with the LSV curves. Differing obviously from IrO_2_ (Fig. 4d), the increasing phase angle of IrCoO_*x*_-2 (Fig. 4e) in the high-frequency region as the applied voltage increases is associated with the catalyst oxidation (including Co dissolution and crystallinity change)^42^. The introduction of SnO_2_ improves the structure stability, as evidenced by the re-stabilized phase angles of IrCoO_*x*_-2@SnO_2_ (Fig. 4f) even when the potential reached 1.8 V.

Finally, a PEM electrolyzer was successfully assembled (Figs. S20–S23) using as-prepared IrCoO_*x*_-2@SnO_2_ and commercial Pt/C as anode and cathode catalysts respectively. Remarkably, the PEM cell can operate stably for 3880 h even at 2 A cm^-2^ (Fig. 4g), confirming the competitive stability of the as-prepared IrCoO_*x*_-2@SnO_2_ catalyst. Notably, the increase of cell potential around 2200 h was traced to a deteriorated inlet pipe (Fig. S24), which can be recovered by changing the new inlet pipe. The cross-section SEM image of CCM (inset I of Fig. 4g) consolidates the homogeneity of the catalyst layer with the thickness of 9.69 um (anode) and 10.8 um (cathode), respectively. A comparison with recently reported high-performance OER electrocatalysts (Fig. S25 and Table S5) highlights that our IrCoO_*x*_-2@SnO_2_ enables the competitive documented continuous operation at 2 A cm^-2^ to date. Besides, the assembled standard PEM electrolyzer with a working area of 25 cm^2^ (Figs. S26–S28) demonstrated stable operation at currents up to 25 A, further validating the scalability and application potential of our catalyst. To evaluate the reliability of the produced H_2_, we constructed an integrated water splitting-fuel cell linkage system. The H_2_ fuel generated was used to effectively power a mobile phone (inset II of Fig. 4g) or LED panel (Fig. S29), demonstrating its practical utility.

### Role of SnO_2_ in paracrystalline structure stabilization

The XPS O 1 s spectra of IrCoO_*x*_-2 catalyst (Fig. S30) changes greatly after the stability test, including the complete disappearance of the O_M_ species associated with long-range disorder. Quantificationally, the AF of IrCoO_*x*_-2 drops from 0.37 to 0 (Fig. S32), confirming the collapse of paracrystalline structure. In contrast, no decrease can be found for the AF of IrCoO_*x*_-2@SnO_2_-after (Fig. S31 and Table S6). Instead, it increases from 0.28 to 0.34, which can be perfectly explained by the STEM-EDS elemental mapping (Fig. S33). The post-reaction IrCoO_*x*_-2@SnO_2_ surprisingly turn into core-shell morphology (Figs. S34–S35) with a SnO_2_ core and an IrCoO_*x*_-2 shell. Therefore, the AF increases to 0.34 because of the full exposure of IrCoO_*x*_-2, which is almost same with unreacted IrCoO_*x*_-2 (0.37). XPS Ir 4 f spectra (Fig. S36) reveals a positive binding energy shift by 0.37 eV for the post-OER IrCoO_*x*_-2, proving the undesired increase of Ir valence, which is usually considered as the critical deactivation indicator for Ir-based catalysts. In stark contrast, IrCoO_*x*_-2@SnO_2_ exhibits a much smaller Ir 4 f shift (0.17 eV, Fig. S37). The same trend is also found in XPS Co 2p spectra (2.25 eV vs. 1.55 eV) in Figs. S38–S39, consolidating the role of SnO_2_ for stability enhancement by inhibiting the valence increase. The above results strongly demonstrate the stability enhancement effect of SnO_2_ for paracrystalline IrCoO_*x*_-2. Furthermore, the inductively coupled plasma (ICP) results of the electrolyte after the stability test (Table S7) clearly demonstrated that the content of dissolved Ir decreased from 0.56 μg/mL to 0.38 μg/mL thanks to the introduction of SnO_2_.

**Fig. 5:**
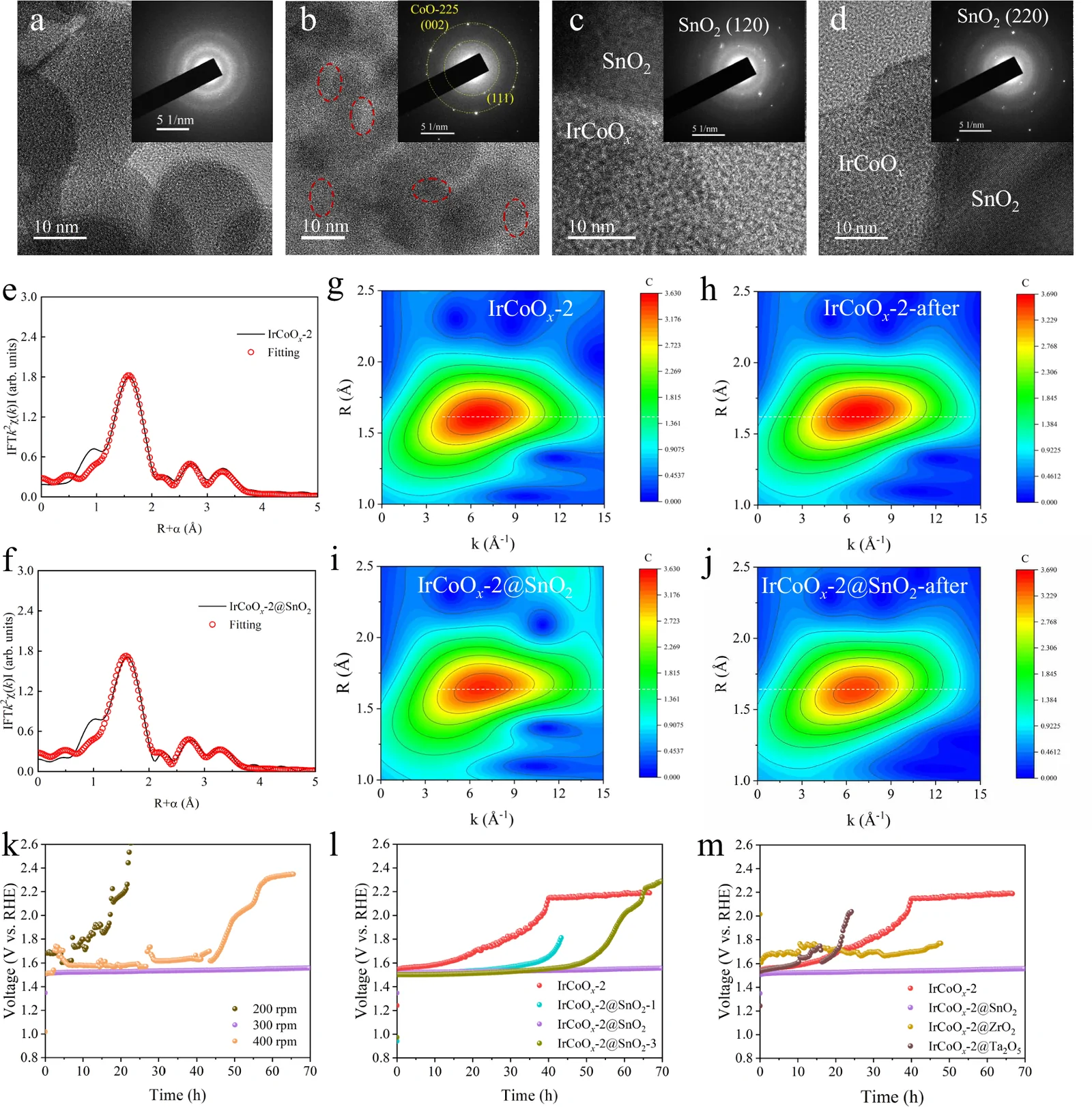
Exploration of the role of SnO_2_ in the composite catalyst. The TEM images of **a** IrCoO_*x*_-2, **b** IrCoO_*x*_-2-after, **c** IrCoO_*x*_-2@SnO_2_ and **d** IrCoO_*x*_-2@SnO_2_-after. (Insets: the corresponding SAED images). EXAFS fitting results of **e** IrCoO_*x*_-2 and **f** IrCoO_*x*_-2@SnO_2_ catalysts (R: Radial Distance). The corresponding wavelet transform (WT) patterns for **g** IrCoO_*x*_-2, **h** IrCoO_*x*_-2-after, **i** IrCoO_*x*_-2@SnO_2_ and **j** IrCoO_*x*_-2@SnO_2_-after. The chronopotentiometry curves at 10 mA cm^-2^ for as-prepared IrCoO_*x*_-2@SnO_2_ catalysts with **k** various ball milling rates and **l** varied introduction amounts of SnO_2_. **m** The chronopotentiometry curves at 10 mA cm^-2^ for IrCoO_*x*_-2, IrCoO_*x*_-2@SnO_2_, IrCoO_*x*_-2@ZrO_2_ and IrCoO_*x*_-2@Ta_2_O_5_ catalysts.

Compared with the paracrystalline characteristics of IrCoO_*x*_-2 (Fig. 5a), some clear lattice fringes (marked with red circles) and diffraction spots corresponding to CoO-225 phase were found for IrCoO_*x*_-2-after (Fig. 5b), confirming the disappearance of paracrystalline structure. The EDS elemental mapping and HAADF images (Figs. S40–S42) unambiguously prove the obvious crystallization and agglomeration of Ir and Co elements. For IrCoO_*x*_-2@SnO_2_, the SAED pattern (Fig. 5c) reveals the co-existence of paracrystalline IrCoO_*x*_-2 and diffraction spots for SnO_2_^43^. Correspondingly, the paracrystalline characteristics of IrCoO_*x*_-2@SnO_2_ can be well maintained after the stability test (Fig. 5d), providing direct visual evidence of the stabilizing role of SnO_2_.

The Fourier-transformed extended X-ray absorption fine structure (EXAFS) and the fitting results (Figs. 5e, f and S43 and Table S8) reveal a longer Ir-O bond in IrCoO*x*-2 compared with commercial IrO2, attributed to the expanded lattice owing to Co incorporation. Crucially, the negligible difference in Ir-O bond length (1.99 ± 0.01 Å *vs*. 2.00 ± 0.01 Å) between IrCoO_*x*_-2 and IrCoO_*x*_-2@SnO_2_ indicates that this Ir-O bond is well preserved after introducing the SnO_2_ support, confirming the undisturbed paracrystalline structure in IrCoO_*x*_-2@SnO_2_. For the IrCoO_*x*_-2 catalyst, there are obvious changes in both the coordination number (5.9 ± 0.3 → 5.5 ± 0.3) and bond length (1.99 ± 0.01 → 2.02 ± 0.01) after OER, differing from those of IrCoO_*x*_-2@SnO_2_. Thus, we can reasonably conclude that the introduction of SnO_2_ is an effective strategy for maintaining the paracrystalline structure of IrCoO_*x*_-2. Moreover, by combining the fitting results of Ir L_3_-edge and Co K-edge EXAFS (Fig. S44 and Table S9), the presence of Co-Ir scattering contributions was confirmed, supporting the substitution of Co into the IrO_*x*_ lattice. Besides, the WT patterns of Ir L_3_-edge EXAFS signals (Fig. 5g–j) display the predominated Ir-O signals. Differing from the obvious shift of Ir-O signal for IrCoO_*x*_-2 after OER, there is hardly any alteration for IrCoO_*x*_-2@SnO_2_ after the stability test (Figs. S45–S46), providing definitive evidence for the stabilizing function of SnO_2_ at the atomic level.

A series of control samples were prepared by varying the ball-milling speed and the amounts of SnO_2_ introduced. Through the direct comparisons (Fig. 5k, l) among the several chronopotentiometry curves at 10 mA cm^-2^, the optimal parameters were determined to be a ball-milling rate of 300 rpm and m(IrCoO_*x*_-2):m(SnO_2_) ratio of 2:1. It is believed that excessively high ball-milling speed may damage the paracrystalline structure of IrCoO_*x*_-2 catalyst. Furthermore, excessive SnO_2_ loading was found to compromise stability, likely due to inadequate mixing and dispersion. Importantly, IrCoO_*x*_-2@SnO_2_ demonstrates the best OER stability (Fig. 5m) among three common acid-resistant oxides, including SnO_2_, ZrO_2_ and Ta_2_O_5_, underscoring the uniqueness of SnO_2_ for paracrystalline IrCoO_*x*_-2.

The stability enhancement by SnO_2_ can be attributed to its triple role: (1) maintaining the paracrystalline structure of IrCoO_*x*_; (2) inhibiting the over-oxidation; (3) mitigating the dissolution and agglomeration of Ir and Co during OER. This is enabled by a well-matched IrCoO_*x*_-2@SnO_2_ heterostructure, which is thermodynamically stabilized by low interfacial energy and kinetically locked by high lattice matching, collectively suppressing atomic diffusion and structural degradation.

### Mechanistic insights in the combined high activity and stability

**Fig. 6:**
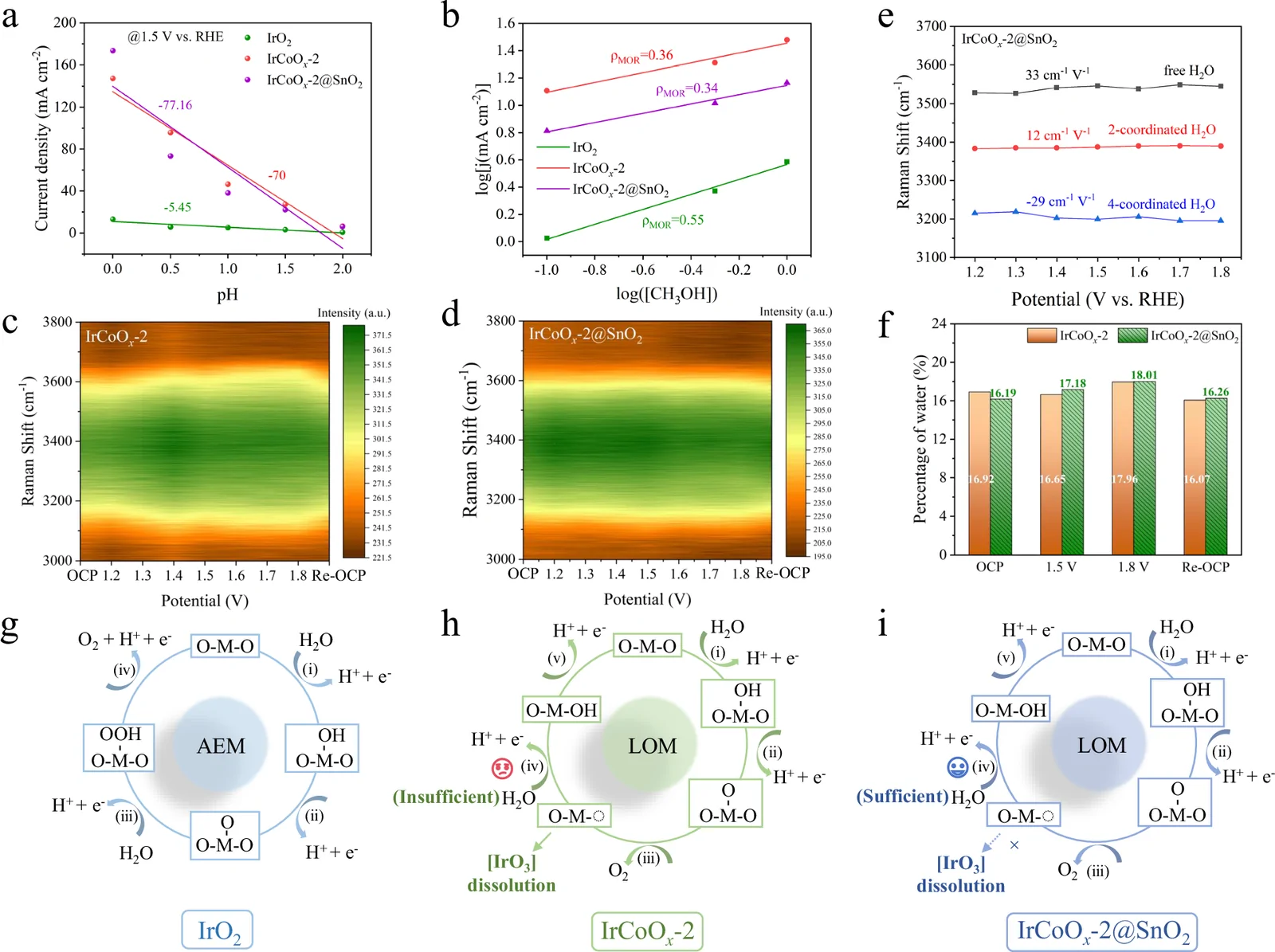
Detailed catalytic mechanism exploration for OER. **a** pH-dependent current densities at 1.5 V (vs. RHE) for IrO_2_, IrCoO_*x*_-2 and IrCoO_*x*_-2@SnO_2_. **b** Logarithmic plot of MOR current density versus methanol concentration for IrO_2_, IrCoO_*x*_-2 and IrCoO_*x*_-2@SnO_2_. Two-dimensional color maps showing the shift of in-situ Raman peaks on **c** IrCoO_*x*_-2 and **d** IrCoO_*x*_-2@SnO_2_. **e** Potential-dependent Raman peak shifts of varied interfacial water types in IrCoO_*x*_-2@SnO_2_. **f** Percentage comparison of free H_2_O structure among IrCoO_*x*_-2 and IrCoO_*x*_-2@SnO_2_ at applied potentials of OCP, 1.5 V, 1.8 V and Re-OCP. The schematic illustration of the reaction pathway for **g** IrO_2_, **h** IrCoO_*x*_-2 and **i** IrCoO_*x*_-2@SnO_2_.

To experimentally delve into the enhancement mechanism and the specific role of SnO_2_, the detailed OER pathway was studied. The pH-dependent current densities at 1.5 V (vs. RHE) were directly compared, among which commercial IrO_2_ exhibited a slope of -5.45 mA cm^-2^ (Figs. 6a and S47–S49), consolidating the insensitive pH response for AEM pathway. Largely differing from AEM prevalent in commercial IrO_2_, the LOM involves non-proton-coupled electron transfer (non-PCET) steps, rendering it highly sensitive to pH variations. Correspondingly, the as-prepared IrCoO_*x*_-2 and IrCoO_*x*_-2@SnO_2_ catalysts show obviously higher slopes of -70 mA cm^-2^ and -77.16 mA cm^-2^, respectively, proving the predominant LOM pathway for the created paracrystalline structure^44,45^. Moreover, the over-oxidation of Ir (Ir^IV^O_2_ → Ir^V^O_2_-OH→Ir^VI^O_3_) to soluble high-valence [IrO_3_] species is known as the main deactivation cause of commercial IrO_2_. Therefore, the undesired electrophilic group-OH* should be rigorously monitored during OER, which can be quantified by adding nucleophilic reagent-methanol (Figs. S50–S52). Specifically, methanol is used as a molecular probe to assess the *OH surface coverage, as the methanol oxidation reaction (MOR) proceeds via nucleophilic attack of methanol molecules on electrophilic *OH species. Consequently, a catalyst that is less active toward MOR (indicated by a lower slope) reflects lower *OH coverage on its surface. The IrCoO_*x*_-2 (0.36) and IrCoO_*x*_-2@SnO_2_ (0.34) catalysts possess lower slopes than commercial IrO_2_ (0.55), confirming lower *OH coverage on the catalyst surface (Fig. 6b). This suppressed *OH accumulation inhibits the irreversible Ir oxidation, directly contributing to the superior stability of the paracrystalline catalysts^14,30^.

To gain molecular-level insight into water-catalyst interactions, we conducted in-situ electrochemical surface-enhanced Raman spectroscopy (SERS) measurements. It is recognized that water networks with either excessively strong or weak hydrogen bonding are suboptimal for OER. Impressively, both IrCoO_*x*_-2 and IrCoO_*x*_-2@SnO_2_ catalysts (Figs. S53–S54 and Tables S10–S11) exhibited the predominant 2-coordinated H_2_O species across all detected potentials. This moderate configuration suggests a favorably structured interfacial water layer, which is crucial for efficient proton transfer and overall OER kinetics^46^.

From the two-dimensional color maps in Fig. 6c, d, IrCoO_*x*_-2@SnO_2_ catalyst demonstrates more stable water distribution at 3200–3600 cm^-1^ than IrCoO_*x*_-2 with voltage variation, suggesting better preservation of an optimal hydrogen-bonding network and more sustainable lattice oxygen participation. Both IrCoO_*x*_-2 and IrCoO_*x*_-2@SnO_2_ catalysts (Figs. 6e and S55) exhibit the steepest Stark slope (45 cm^-1^ V^-1^ and 33 cm^-1^ V^-1^) for free H_2_O compared with 2-coordinated H_2_O and 4-coordinated H_2_O, confirming the most sensitive response of free H_2_O to local electric fields^47^. Therefore, the percentage of free H_2_O as a key influencing factor was further studied in Fig. 6f. Under anodic polarization, both catalysts show an increase in free water content, with IrCoO_*x*_-2@SnO_2_ retaining a higher proportion than IrCoO_*x*_-2 at 1.5 V and 1.8 V, ensuring sufficient interfacial water supply during OER. Notably, compared with the percentage of free H_2_O (16.92) at open-circuit potential (OCP), IrCoO_*x*_-2 catalyst exhibits a marked decrease after returning to the OCP (16.07), which will lead to the loss of lattice oxygen. In stark contrast, a slight increase (16.19 → 16.26) was observed for IrCoO_*x*_-2@SnO_2_ catalyst, strongly consolidating the role of SnO_2_ in enhancing catalytic stability through improved water management and structural preservation.

To clearly elucidate the OER pathway of as-prepared IrCoO_*x*_-2@SnO_2_, O^18^ isotope-labeling differential electrochemical mass spectrometry (DEMS) was employed. Firstly, the working electrode was immersed in 0.5 M H_2_SO_4_ prepared with H_2_O^18^ and then multiple LSV scans were made between 1.1-1.5 V (vs. RHE). After rinsing 10 times with H_2_O^16^, the same electrochemical procedure was repeated in 0.5 M H_2_SO_4_ prepared with H_2_O^16^, while recording the gaseous products and their isotopic composition in real time by mass spectrometry. As shown in Fig. S56, the presence of O^18^O^18^ and O^16^O^18^ peaks confirms the exchange of O^18^ into the lattice of IrCoO_*x*_-2@SnO_2_, thus consolidating the LOM mechanism.

The mechanism of SnO_2_ in altering the OER pathway is clearly illustrated in Fig. 6g–i. The relatively sluggish AEM pathway occurs for commercial IrO_2_, resulting in a high OER overpotential. Although improving the reaction kinetics, the paracrystalline IrCoO_*x*_-2 triggers LOM in virtue of the cycle of lattice oxygen. When the free water supply at the interface is insufficient, the consumed lattice oxygen cannot be efficiently replenished (Step iv), leading to irreversible structural damage and oxidation of Ir sites. Resultantly, the formation and dissolution of soluble [IrO_3_] species compromise the catalyst stability. Upon the introduction of SnO_2_, the increased abundance of free water at the interface can supply necessary reactants at Step iv (O-M-◌ + H_2_O → H^+^ + e^-^ + O-M-OH) and sustain a reversible oxygen cycle during OER. This mechanistic insight clearly explains the critical role of SnO_2_ in enhancing OER stability.

### Thermodynamic and kinetic mechanisms by theoretical calculations

**Fig. 7:**
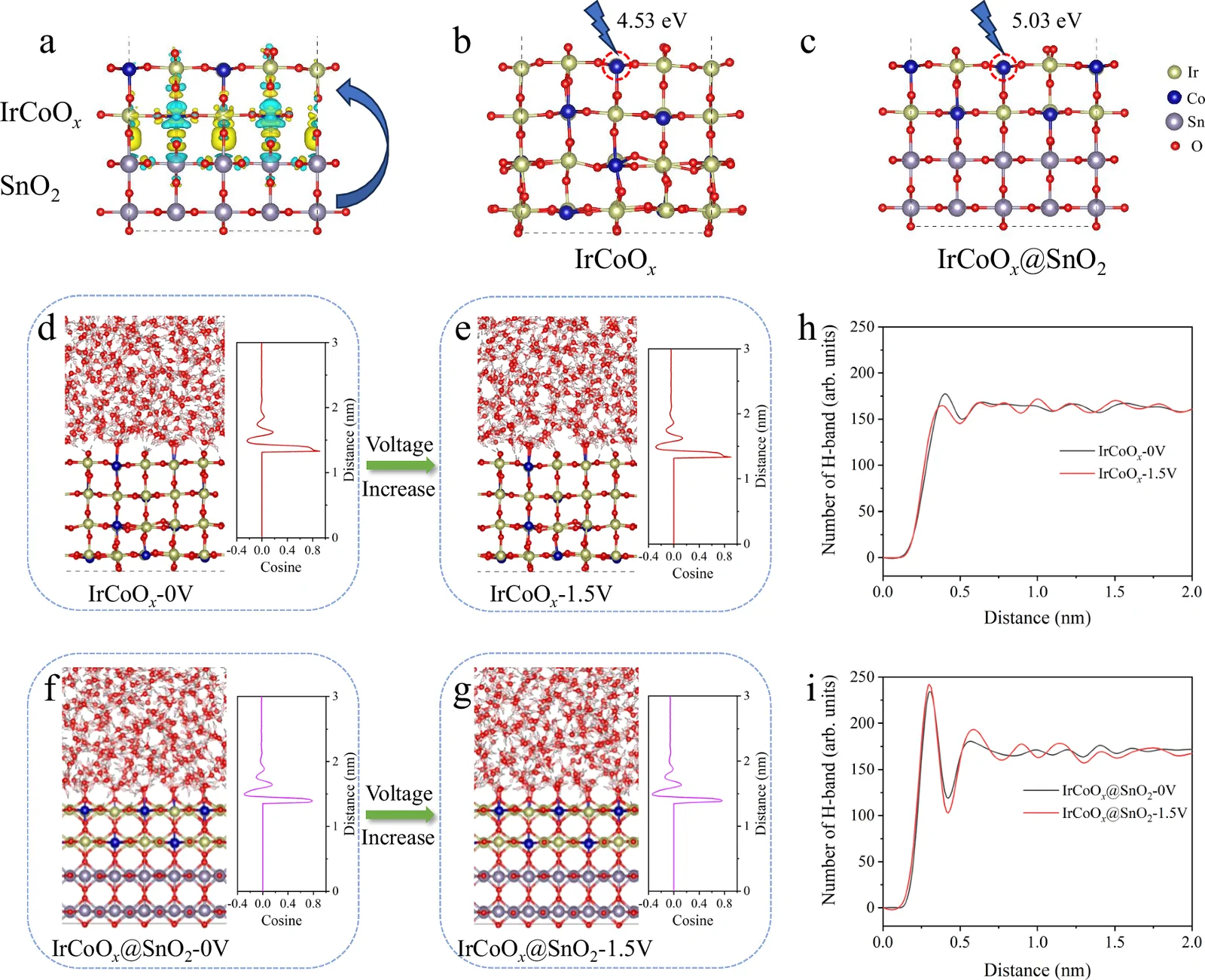
Mechanism studies and interfacial water distributions by theoretical calculations. **a** The differential charge density of IrCoO_*x*_@SnO_2_ heterostructure (cyan and yellow represent electron dissipation and accumulation, respectively). The formation energy of Co vacancy on **b** IrCoO_*x*_ and **c** IrCoO_*x*_@SnO_2_. The schematic diagram of interfacial water molecules as simulated by AIMD for **d** IrCoO_*x*_-0V, **e** IrCoO_*x*_-1.5 V, **f** IrCoO_*x*_@SnO_2_-0V, and **g** IrCoO_*x*_@SnO_2_-1.5 V (Insets: The corresponding orientation distribution). The interfacial distribution of hydrogen bond for **h** IrCoO_*x*_ and **i** IrCoO_*x*_@SnO_2_ at the applied potential of 0 V and 1.5 V.

To further elucidate the role of SnO_2_ in modulating the paracrystalline IrCoO_*x*_ catalyst, we performed combined density functional theory (DFT) and ab initio molecular dynamics (AIMD) calculations (Supplementary Data 1 file). From the differential charge density in Figs. 7a and S57, a clear interfacial electron accumulation occurs at the IrCoO_*x*_/SnO_2_ interface and about 0.412 |e| transferred from SnO_2_ to IrCoO_*x*_, resulting in relatively low-valence Ir sites for excellent OER stability. From a thermodynamic perspective, the formation energy of Co vacancy for IrCoO_*x*_@SnO_2_ (5.03 eV) is obviously higher than that of IrCoO_*x*_ (4.53 eV), consolidating the structural stabilization effect imparted by SnO_2_ (Fig. 7b, c).

The distribution and orientation of water molecules in the electrical double-layer microenvironment are shown in Fig. 7d–g. Specifically, water molecules exhibit an overall H-up trend at the closest interface. This oriented arrangement exposes the higher-energy dangling hydrogen atoms at the interface while submerging the lower-energy oxygen atoms into the bulk water phase, thereby maximizing hydrogen-bond formation and achieving system energy minimization^48^.

From the insets in Fig. 7d–g, the surface water molecular orientation polarization in IrCoO_*x*_@SnO_2_ is less pronounced than in IrCoO_*x*_, making it easier for IrCoO_*x*_@SnO_2_ to form stable hydrogen bonds with surrounding free water molecules. Consequently, IrCoO_*x*_@SnO_2_ (234) exhibits a higher number of hydrogen bonds at the closest interface than IrCoO_*x*_ (177) (Fig. 7h, i), demonstrating that SnO_2_ modification achieved an optimal balance of the hydrogen-bond network. Remarkably, the number of hydrogen bonds on IrCoO_*x*_ surface showed a significant decrease (163) after applying a 1.5 V voltage for the system, while IrCoO_*x*_@SnO_2_ surface even exhibited a further increase (242). The disruption of hydrogen bond networks for IrCoO_*x*_ under high voltage is detrimental to catalyst stability, preventing adequate proton transfer and thus leading to structural corrosion, which is proven to be effectively resolved by the introduction of SnO_2_. Importantly, the fact that the water molecule numbers remain unchanged under 1.5 V (Figs. S58-S59) rules out the possibility that the observed changes in hydrogen bonding are caused by a reduction in water content.

## Discussion

In summary, we have successfully addressed the long-standing activity-stability trade-off effect in acidic OER by constructing and stabilizing a paracrystalline IrCoO_*x*_ phase. The formation of this unique structure, directly verified by in-situ heating TEM imaging, was achieved through controlled Co incorporation, which was further stabilized by structure-coherent rutile SnO_2_ support. The resulting IrCoO_*x*_-2@SnO_2_ catalyst achieves a mass activity of 1192 A g_Ir_^-1^, 114 times that of commercial IrO_2_, and long operation stability for at least 3880 h at 2 A cm^-2^ in PEMWE cell. Combining DFT and AIMD calculations with in-situ SERS analysis, we reveal that SnO_2_ support not only ensures the extra-high structural stability of the paracrystalline phase but also optimizes the interfacial water environment, thereby enabling reversible oxygen species cycling and efficient proton transfer during OER. This work provides a highly active and durable catalyst for OER, and in the meantime, a general design principle and mechanism insight for metastable active phase stabilization, which is thought to be highly promising in practical PEM electrolysis in the coming future.

## Methods

### Chemicals

Iridium trichloride (IrCl_3_, 99.9%), Tin oxide (SnO_2_, 99.99%), Zirconium dioxide (ZrO_2_, 99.99%), Tantalum oxide (Ta_2_O_5_, 99.99%) and silver crucible were purchased from Adamas Reagent Co., Ltd. Cobalt dichloride hexahydrate (CoCl_2_ · 6H_2_O, ≥99.0%), Ferric chloride hexahydrate (FeCl_3_ · 6H_2_O, ≥99.0%), Manganese chloride tetrahydrate (MnCl_2_ · 4H_2_O, ≥99.0%), Nickel(II) chloride hexahydrate (NiCl_2_ · 6H_2_O, ≥99.0%), Copper (II) chloride dihydrate (CuCl_2_ · 2H_2_O, ≥99.0%) and sodium hydroxide (NaOH, ≥96.0%) were purchased from Sinopharm Chemical Reagent Co., Ltd. All materials were used without further purification.

### Synthesis of IrCoO_*x*_

0.05 g of IrCl_3_ and a certain amount of CoCl_2_ · 6H_2_O were mixed into 3 g of NaOH. Then, the mixture was placed in a silver crucible and heated to 360 °C in a muffle furnace with a heating rate of 3°C min^-1^. After holding for 6 h, the mixture was washed and centrifuged with deionized water and ethanol three times to obtain IrCoO_*x*_ samples. IrCoO_*x*_-1, IrCoO_*x*_-2 and IrCoO_*x*_-3 were named according to the corresponding addition amount of CoCl_2_ · 6H_2_O with 0.01 g, 0.015 g and 0.025 g, respectively. It’s found that IrCoO_*x*_-2 is optimal for the highest OER catalytic activity.

### Synthesis of IrO_*x*_ and CoO_*x*_

IrO_*x*_ and CoO_*x*_ were prepared using the same procedure with IrCoO_*x*_-2 except for the single addition of IrCl_3_ or CoCl_2_ · 6H_2_O.

### Synthesis of IrFeO_*x*_, IrMnO_*x*_, IrNiO_*x*_ and IrCuO_*x*_

IrFeO_*x*_, IrMnO_*x*_, IrNiO_*x*_ and IrCuO_*x*_ were prepared using the same procedure with IrCoO_*x*_-2 except for the replacement addition of FeCl_3_ · 6H_2_O, MnCl_2_ · 4H_2_O, NiCl_2_ · 6H_2_O and CuCl_2_ · 2H_2_O, respectively.

### Synthesis of IrCoO_*x*_-2@SnO_2_

50 mg of IrCoO_*x*_-2 and a certain amount of SnO_2_ were mixed in a polytetrafluoroethylene ball milling jar with the addition of ethanol and ball-milled for 6 h in a planetary high-speed centrifuge. It’s found that IrCoO_*x*_-2@SnO_2_ is optimal for the best OER catalytic stability with the SnO_2_ addition amount of 25 mg and the ball milling speed of 300 rpm. IrCoO_*x*_-2@SnO_2_-1 and IrCoO_*x*_-2@SnO_2_-3 are referred to the SnO_2_ addition amount of 10 and 35 mg, respectively.

### Synthesis of IrCoO_*x*_-2@ZrO_2_ and IrCoO_*x*_-2@Ta_2_O_5_

IrCoO_*x*_-2@ZrO_2_ and IrCoO_*x*_-2@Ta_2_O_5_ were prepared using the same procedure with IrCoO_*x*_-2@SnO_2_ except for the replacement addition of 25 mg ZrO_2_ and 25 mg Ta_2_O_5_, respectively.

### Synthesis of IrCoO_*x*_-2-after and IrCoO_*x*_-2@SnO_2_-after

IrCoO_*x*_-2-after and IrCoO_*x*_-2@SnO_2_-after were obtained after the OER stability test of 50000 s at 10 mA cm^-2^ for IrCoO_*x*_-2 and IrCoO_*x*_-2@SnO_2_, respectively.

### Materials characterization

Powder X-ray diffraction (XRD) patterns were collected on a Rigaku D/Max-2550 V X-ray diffractometer with a Cu K_α_ radiation target (40 kV, 40 mA). Transmission electron microscopy (TEM) was performed on a JEOL JEM-F200 microscope operated at 200 kV. The probe aberration corrected transmission electron microscope images were obtained on a Sectra300 (FEI, USA, at 300 kV). Raman spectra were acquired on Xplora ONE-532. X-ray photoelectron spectroscopy (XPS) was performed on a Thermo Fisher Scientific ESCAlab250 XPS instrument with monochromatic Al K_α_ X-rays and calibrated with carbon base (284.8 eV). Electron spin resonance (ESR) signals were measured on a Bruker A300 ESR instrument. Inductively coupled plasma atomic emission spectroscopy (ICP-AES) was undertaken on a Varian Vista-AX ICP emission spectrometer.

The in-situ TEM measurements were also conducted on JEOL JEM-F200 microscope operated at 200 kV. The in-situ heating experiments were performed on a heating holder with the addition of IrO_*x*_ and CoO_*x*_ mixture by a SmartSet Hot Stage controller (Gatan Model 901) from room temperature (RT, 25 °C) to 360 °C and 0.5 h holding time at RT, 100, 200, 300, 360 °C, respectively.

The in-situ electrochemical surface-enhanced Raman spectroscopy (SERS) measurements with the pinhole-free Au@SiO_2_ shell-isolated nanoparticles (SHINs) were recorded simultaneously during constant potential scans using a dedicated three-electrode spectro-electrochemical Raman cell (Xplora Plus). The applied potentials were OCP, 1.2 V, 1.3 V, 1.4 V, 1.5 V, 1.6 V, 1.7 V, 1.8 V and Re-OCP, respectively. The acquisition time for every potential was 1 min. Electrode inks were casted on the glassy carbon working electrode and dried in air and tested in 0.5 M H_2_SO_4_. The excitation wavelength of Raman testing is 785 nm and the Raman shift ranged from 3000 to 3800 cm^-1^.

Online differential electrochemical mass spectrometry (DEMS) was carried out using a QAS 100 system (LingLu Instruments) coupled with a CS350MA electrochemical workstation to monitor volatile intermediates and products generated during the electrochemical reaction in real time. Volatile species were transferred into the mass spectrometer under vacuum through a hydrophobic polytetrafluoroethylene (PTFE) membrane. The membrane permitted the permeation of gaseous and volatile species while effectively preventing liquid water from entering the vacuum chamber. The electrochemical reaction occurred on the electrolyte-facing side of the membrane, whereas the volatile products permeating through the membrane were continuously extracted and sequentially analyzed by mass spectrometry. The specific electrochemical configuration and operating conditions were as follows: The sample was loaded onto an Au film electrode, with a Ag/AgCl electrode serving as the reference electrode and a Pt wire as the counter electrode. The working electrode was immersed in 0.5 M H_2_SO_4_ prepared with H_2_^18^O, and multiple linear sweep voltammetry (LSV) scans were performed over a potential range of 1.1-1.5 V (vs. RHE) to label the catalyst surface with ^18^O. In this test, the LSV cycle was repeated 6 times. Specifically, the alternation between an LSV test (1.1-1.5 V vs. RHE, 4 mV s^-1^) and a power-off test (100 s), with the 100-second power-off period used to flush out any bubbles remaining in the chamber from the previous LSV test. The ^18^O-labeled electrode was then thoroughly rinsed repeatedly (10 times total) with a H_2_^16^O solution to remove adsorbed or free H_2_^18^O (flow rate: 3 mL min^-1^). Finally, the same electrochemical procedure was repeated in 0.5 M H_2_SO_4_ prepared with H_2_^16^O, while the gaseous products and their isotopic composition generated during the acidic OER process were monitored in real time by mass spectrometry.

### Electrochemical measurements

Electrochemical characterization was conducted using a CHI760 electrochemical workstation (Chenhua, Shanghai) within a standard three-electrode configuration. A 0.5 M H_2_SO_4_ solution, prepared by diluting concentrated acid with deionized water and stored at ambient temperature (25 °C) in a PTFE bottle, served as the electrolyte. A graphite rod and an Ag/AgCl (saturated KCl) electrode were utilized as the counter and reference electrodes, respectively.

The working electrode was prepared as follows: 10 mg of the catalyst was dispersed in a mixture of 50 μL Nafion (5 wt. %), 50 μL deionized water, and 900 μL isopropanol. The suspension was ultrasonicated for 30 min to achieve a homogeneous ink. Subsequently, 15 μL of this dispersion was drop-cast onto an L-type glassy carbon rotating disk electrode (RDE) with a 5 mm diameter (geometric area of 0.196 cm^2^). After drying under ambient conditions, the catalyst loading on the working electrode was determined to be approximately 0.76 mg cm^-2^.

Prior to use, the Ag/AgCl reference electrode, stored in saturated KCl, was rinsed with deionized water. All measured potentials were converted to the reversible hydrogen electrode (RHE) scale using the Nernst equation (E_RHE_ = E_Ag/AgCl_ + 0.059 × pH + 0.1989 V), where E_Ag/AgCl_ is the external potential measured against the Ag/AgCl reference electrode, and 0.1989 V is the theoretical potential for the Ag/AgCl reference electrode.

Cyclic voltammetry (CV) was performed at a scan rate of 50 mV s^-1^ within a potential window of 0.92 to 1.82 V (vs. RHE). Linear sweep voltammetry (LSV) was carried out at a scan rate of 10 mV s^-1^ over the same potential range while the electrolyte was agitated using a magnetic stirrer at 400 rpm. The uncompensated solution resistance (R_s_), measured to be 3.9 ohm (±0.1) on average, was determined via electrochemical impedance spectroscopy (EIS) at the open-circuit potential, employing a frequency sweep from 100 kHz to 100 mHz with a 10 mV amplitude.

The double layer capacitance was evaluated by cyclic voltammogram curves in non-Faraday region at different scan rates of 10 mV s^-1^, 20 mV s^-1^, 30 mV s^-1^, 40 mV s^-1^ and 50 mV s^-1^. The current density (|Ja-Jc|) is proportional to the scan rate (v), as described in equation: C_dl_ = (|Ja-Jc|)/v. Besides, the electrochemical active areas (ECSA) was acquired by the electrochemical double layer capacitance: ECSA = C_dl_/C_s_ (cm^2^/mg), where Cs is widely used as 40 μF cm^-2^ for acidic Ir-based catalysts.

### MEA preparation and PEM water electrolysis tests

PEM water electrolysis tests were conducted with a KIKUSUI PWR401L power supply. To fabricate the membrane electrode assembly (MEA), the as-prepared IrCoO_*x*_-2@SnO_2_ and commercial 40% Pt/C were used as the anode and cathode catalyst, respectively. Firstly, 12 mg of IrCoO_*x*_-2@SnO_2_ was added to the mixture of 600 μL isopropanol and 87 μL Nafion to obtain anode ink. 6.4 mg of 40 wt. % Pt/C was added in the mixture of 407 μL isopropanol and 58 μL Nafion to obtain cathode ink. Besides, 102 μL isopropanol and 17 μL Nafion were mixed to prepare transition layers. After sonication in a low-temperature water bath for 2h, the well-dispersed ink was sprayed on both sides of the proton exchange membrane (Nafion 212) with the anode and cathode catalyst loading amounts of 1.5 mgcm-2 and 0.8 mgcm-2, respectively. The thickness of Nafion 212 membrane is ~50 μm. To build the PEM electrolyzer for performance evaluation, titanium felt and carbon paper were used as different porous transport layers (PTL) on the anode and cathode, respectively. The geometric active area of the MEA was 4 or 25 cm^2^. The PEM electrolyzer was operated at 80 °C with deionized water as the reactant with the anode flow rate of 20 mL min^-1^. The stability of the PEM electrolyzer was also measured at 80 °C with a constant current density of 2 A cm^-2^.

### Computation details

#### Molecular dynamics (MD) simulations

All MD simulations were carried out using GROMACS (version 2021.3). Interatomic interactions were described using the Universal Force Field (UFF), with atomic charges derived via the charge equilibration (QEq) scheme. Following energy minimization, each system underwent a 1 ns pre-equilibration phase in the NPT ensemble, employing the Berendsen barostat. Subsequent production runs were performed under NPT conditions at 300 K with a 1 fs integration time step. Temperature regulation was achieved using a V-rescale thermostat (τT = 0.1 ps), while pressure was maintained via the Parrinello-Rahman approach (τP = 2 ps). A total simulation time of 20 ns was accumulated, and the resulting trajectories were processed and analyzed using the built-in GROMACS utilities.

#### Density functional theory (DFT) calculations

DFT computations were conducted with the projector-augmented wave (PAW) method, as encoded in the Vienna Ab initio Simulation Package (VASP). The exchange-correlation functional was described by the generalized gradient approximation (GGA) in the Perdew-Burke-Ernzerhof (PBE) form. A plane-wave cutoff energy of 500 eV was employed. During the self-consistent field (SCF) procedure, the convergence threshold for total energy was set to 10^-5^ eV. A vacuum spacing of 15 Å was applied normal to the slab to minimize spurious periodic interactions. The k-point mesh for Brillouin zone sampling was chosen as 2x1x1. Structural optimizations were considered converged when the maximum residual force on each atom dropped below 0.05 eV/Å.

#### Ab Initio molecular dynamics (AIMD)

To investigate the structural stability of IrCoO_*x*_, AIMD simulations were performed. The system was gradually heated from 100 K to 2000 K over a 20 ps period using velocity scaling. The resultant high-temperature configuration was subsequently relaxed to its ground state for further property evaluations. Owing to the robust nature of the metal oxide framework, no appreciable melting was observed during the AIMD heating process.

#### EXAFS data processing and fitting

X-ray absorption fine structure (EXAFS) data reduction and fitting were carried out using the Athena and Artemis interfaces of the Demere package, which relies on the FEFF6 code for theoretical scattering-path calculations. Energy calibration was performed by simultaneously measuring a reference Ir foil alongside the sample. Pre-edge background subtraction was conducted with a linear function, followed by normalization of the edge jump using Athena. The extracted χ(k) signal was isolated by removing a smooth, third-order polynomial background representing the isolated-atom absorption. The k^3^-weighted χ(k) spectra were Fourier transformed over a Hanning-type window (Δk = 1.0 Å^-1^). Structural parameters—including coordination numbers (CN), bond distances (R), Debye-Waller factors (σ^2^), and energy shift (ΔE_0_)—were derived by least-squares fitting of the EXAFS equation to the Fourier-transformed R-space data via Artemis. A fixed amplitude reduction factor (S_0_^2^ = 0.855) was determined from a reference IrO_2_ fit and subsequently applied to the sample fits to extract reliable CN values.

## Supplementary information

Supplementary Information

Description of Additional Supplementary Files

Supplementary Data 1

Transparent Peer Review file

## Source data

Source Data

## Data Availability

The data generated in this study are provided in the Source Data file. All data supporting the findings of this study are available within the paper and its Supplementary information or from the corresponding author upon request. Source data are provided with this paper.
