# Supplementary material for: Paracrystalline IrCoOx stabilization by rutile SnO2 towards durable acidic oxygen evolution
Source: Nat Commun. 2026 Aug 21;17:10577. doi: 10.1038/s41467-026-76919-0 (PMC13646295; doi:10.1038/s41467-026-76919-0)
Supplement: Supplementary file 1 [file 41467_2026_76919_MOESM1_ESM.pdf]

Supplementary Information

Paracrystalline $IrCoO_x$ Stabilization by Rutile SnO_2 Towards Durable Acidic Oxygen Evolution

Han Tian [1#], Ziyi Yu [1#], Hongchang Hao [2], Rui Deng [1], Wenshu Luo [1, 3], Xu Yu [1, 4], Han Wu [1, 4], Xiangzhi Cui [1, 3, 4 *], Jianlin Shi [1, 3 *]

[1] State Key Laboratory of High Performance Ceramics, Shanghai Institute of Ceramics, Chinese Academy of Sciences, Shanghai 200050, China

[2] Materials Science and Engineering Program & Texas Materials Institute, The University of Texas at Austin, Austin, Texas 78712, United States

[3] Center of Materials Science and Optoelectronics Engineering, University of Chinese Academy of Sciences, Beijing 100049, China

[4] School of Chemistry and Materials Science, Hangzhou Institute for Advanced Study, University of Chinese Academy of Sciences, Hangzhou 310024, China

*Corresponding Author E-mail addresses: jlshi@mail.sic.ac.cn (J. Shi), cuixz@mail.sic.ac.cn (X. Cui).

[#]These authors contributed equally to this work.

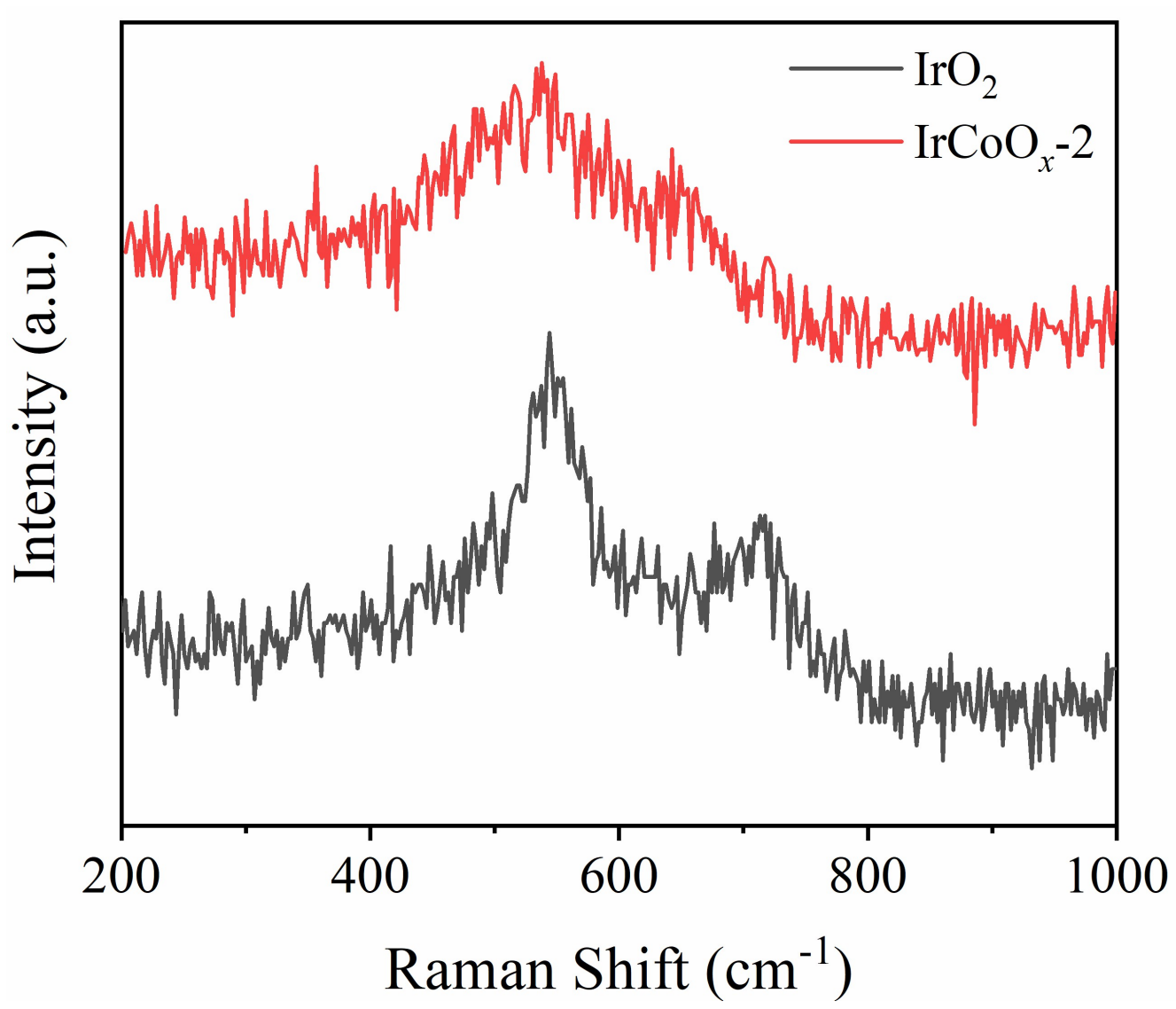

Fig. S1. The Raman spectra of $IrCoO_x$-2 and commercial IrO_2.

The space group assignment of the paracrystalline $IrCoO_x$ structure was derived based on a combination of XRD patterns and *in-situ* heating SAED images. First, unlike the featureless XRD peaks of amorphous phase, the paracrystalline $IrCoO_x$ samples display broadened but distinctive diffraction peaks. The as-prepared $IrCoO_x$ and commercial IrO_2 all show their highest XRD peaks at nearly identical positions at ~35°, suggesting the same space group for paracrystalline $IrCoO_x$ and IrO_2. Second, *in-situ* heating SAED images reveal that an amorphous diffuse halo at 300 °C transforms into paracrystalline diffraction rings at 360 °C. The observed diffraction pattern is almost identical to that of commercial IrO_2, which has a space group of $P4_2/mnm$. Therefore, it is reasonable to derive that the space group of paracrystalline $IrCoO_x$ structure is also $P4_2/mnm$.

The Raman spectrum of $IrCoO_x$-2 exhibits a broadened Ir-O vibration band compared to that of crystalline commercial IrO_2, further supporting this paracrystalline structural assignment.

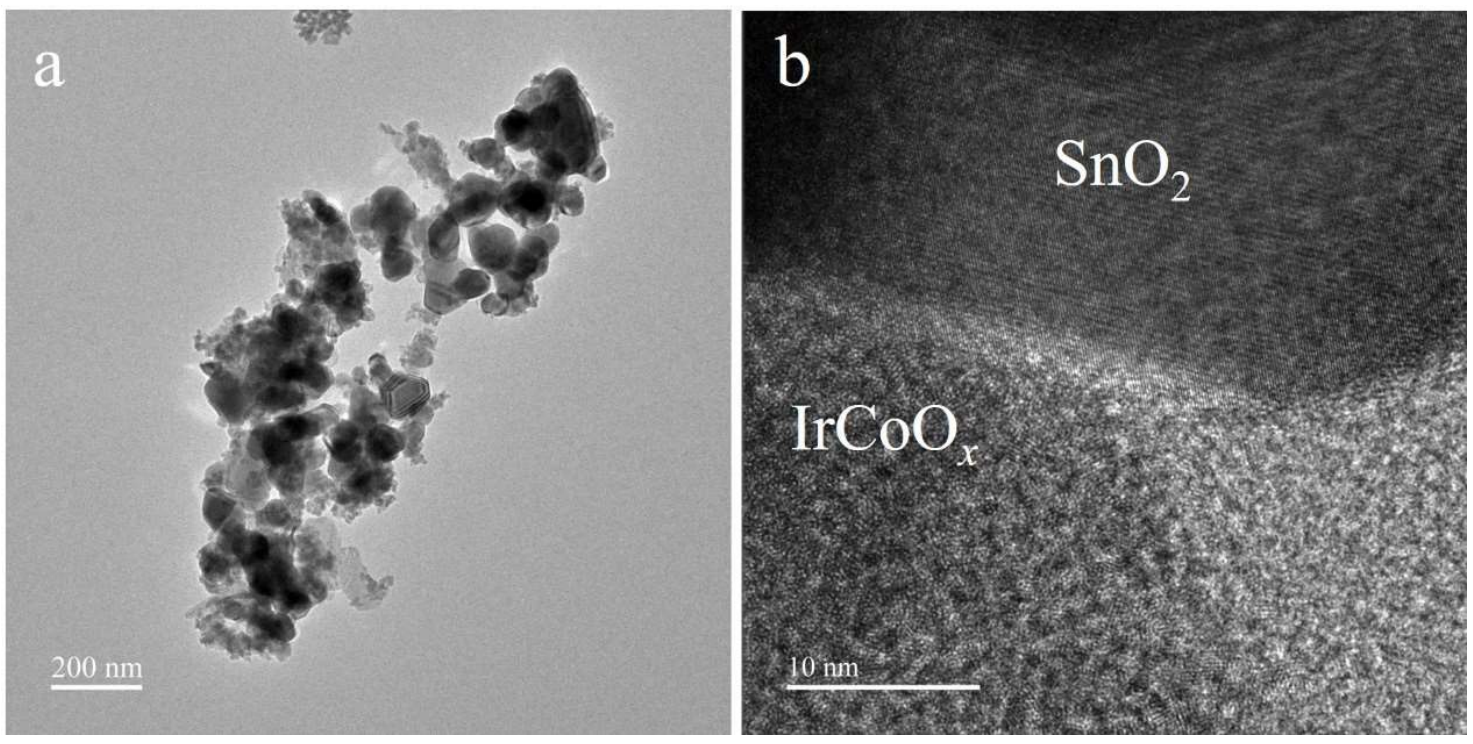

Fig. S2. (a) TEM and (b) HRTEM images of $IrCoO_x$-2@SnO_2.

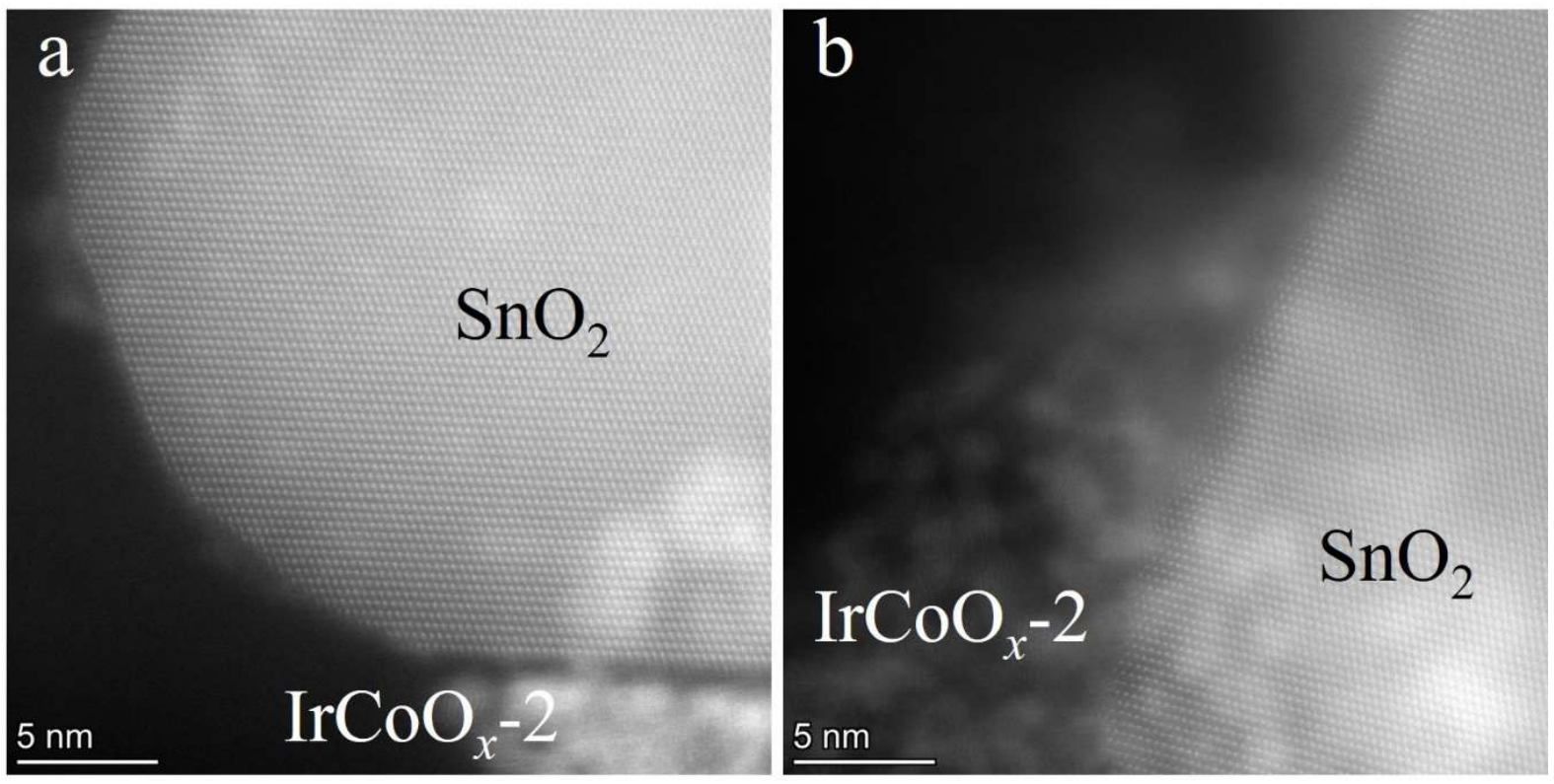

Fig. S3. The atomic-scale TEM images of $IrCoO_x$-2@SnO_2.

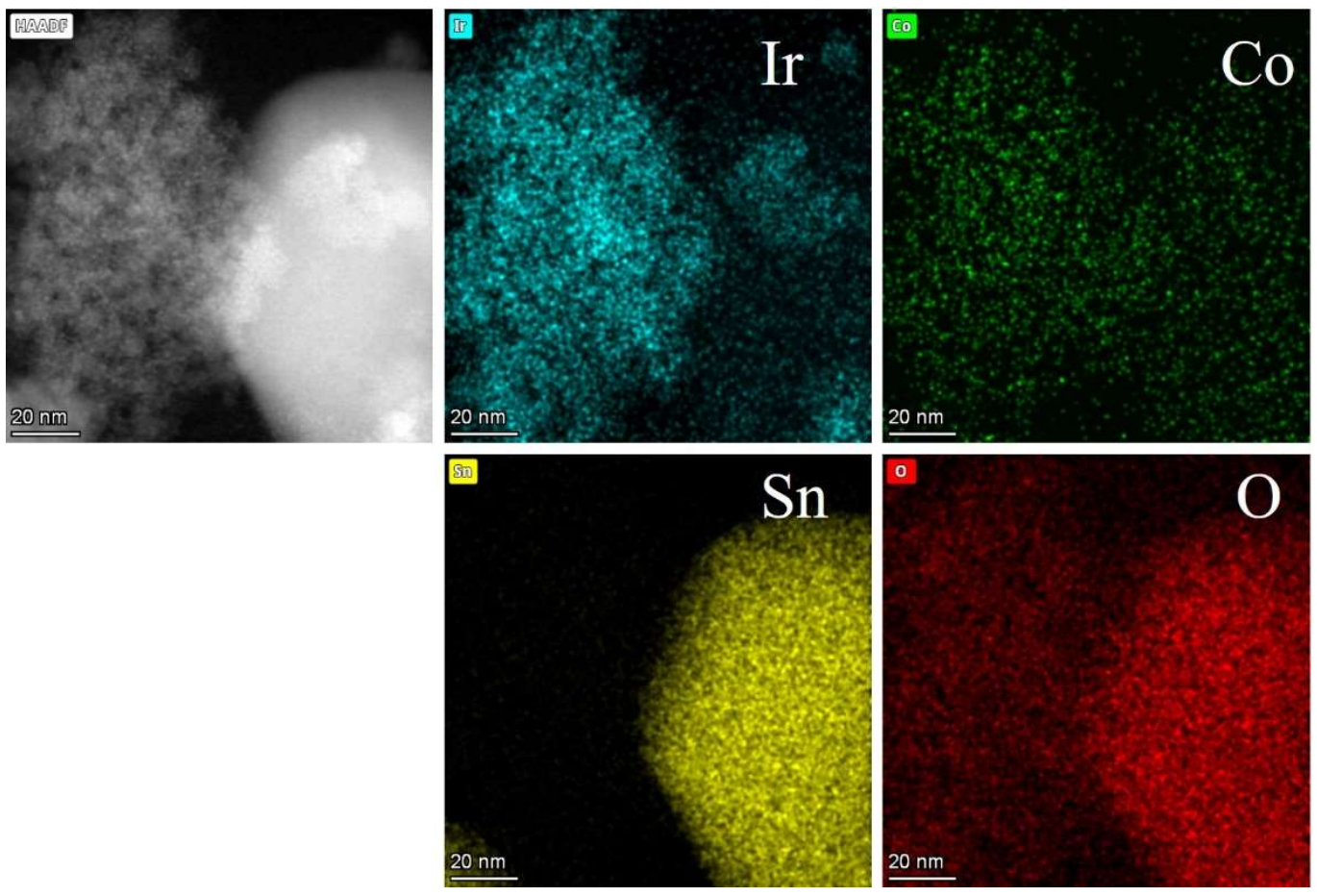

Fig. S4. STEM-EDS elemental mapping of $IrCoO_x$-2@SnO_2.

The EDS elemental mappings of $IrCoO_x$-2@SnO_2 confirms the uniform distribution of Ir, Co, Sn and O elements, as well as the successful construction of $IrCoO_x$-2 and SnO_2 interface.

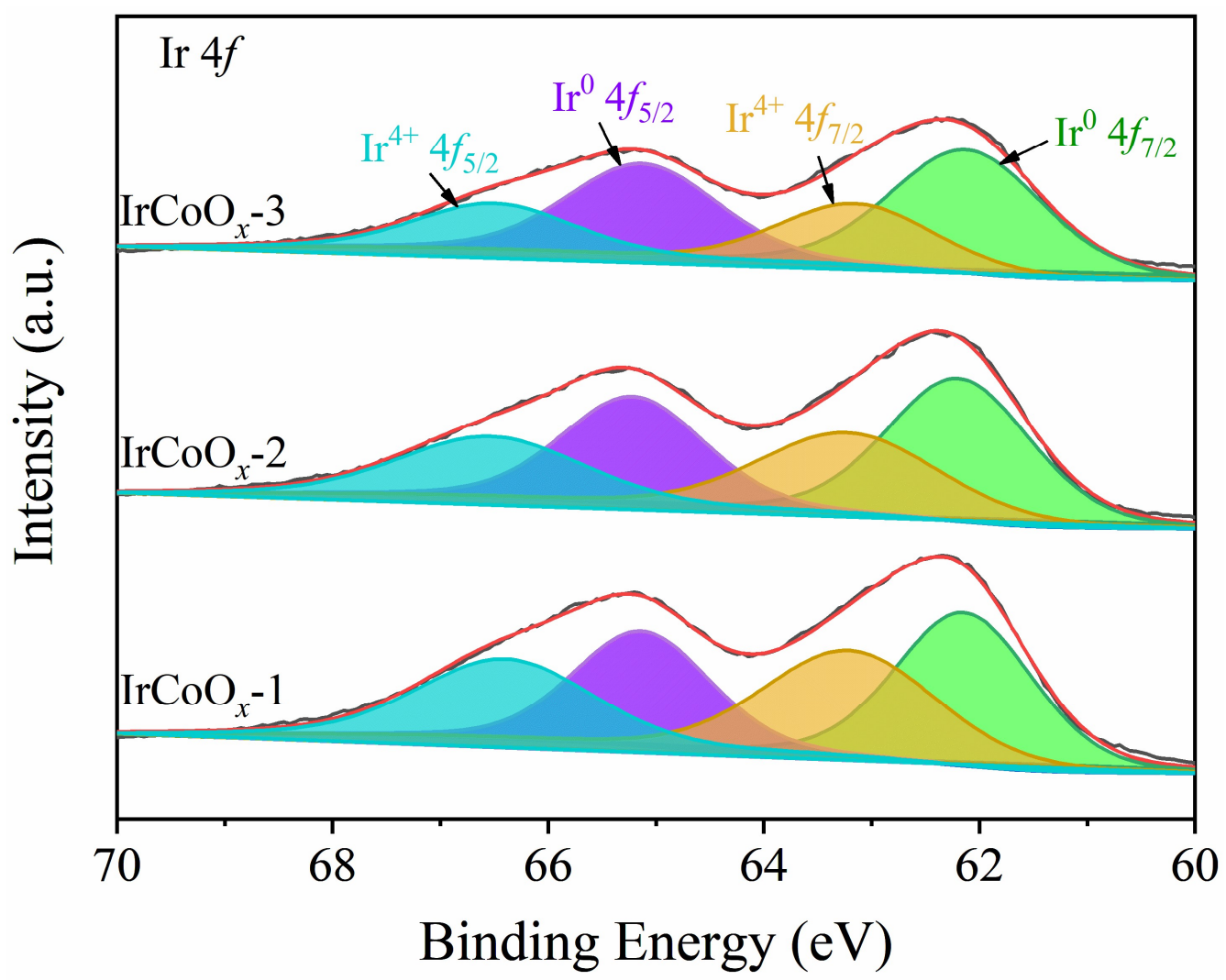

Fig. S5. XPS Ir 4*f* spectra of $IrCoO_x$-1, $IrCoO_x$-2 and $IrCoO_x$-3 samples.

The X-ray photoelectron spectroscopy (XPS) Ir 4*f* spectra (Fig. S5) for all $IrCoO_x$ samples could be deconvoluted into four components, corresponding to Ir^0 $4f_{7/2}$, Ir^{4+} $4f_{7/2}$, Ir^0 $4f_{5/2}$ and Ir^{4+} $4f_{5/2}$ (Table S1).

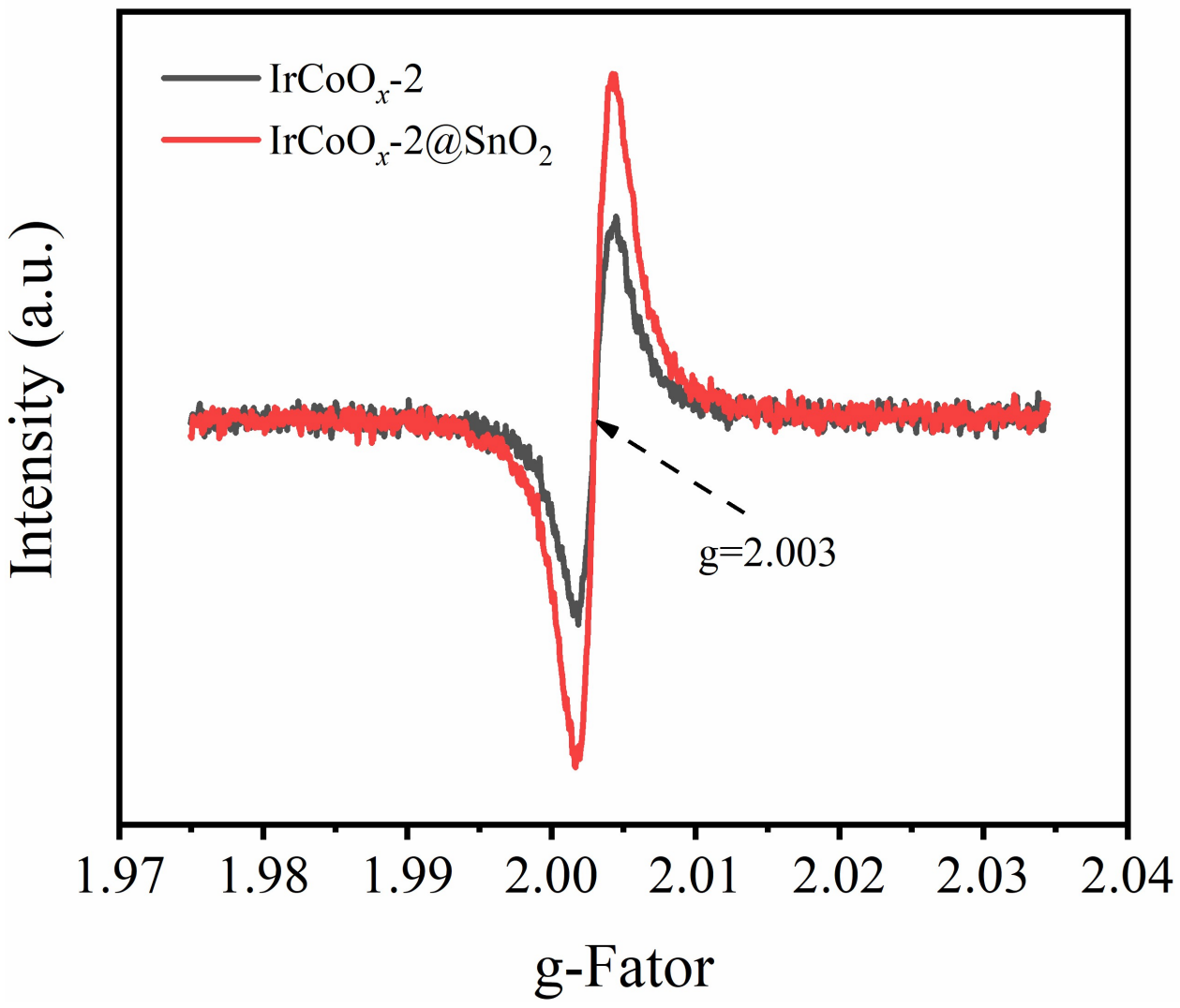

Fig. S6. The ESR results for $IrCoO_x$-2 and $IrCoO_x$-2@SnO_2.

The increase in oxygen vacancy concentration upon introduction of SnO_2 was also confirmed by ESR (Fig. S6), which further validates the reliability of the XPS peak deconvolution (Fig. S7).

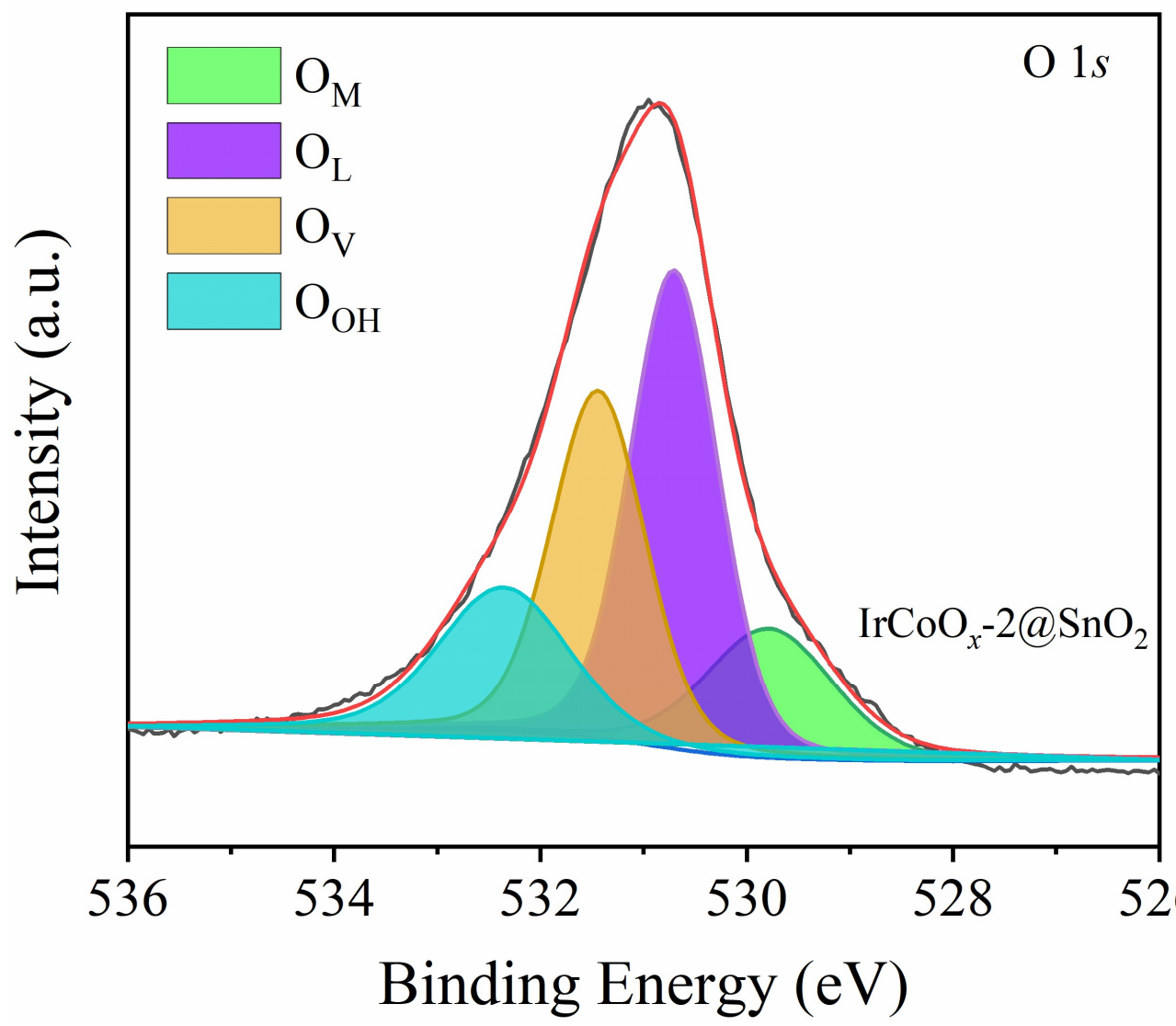

Fig. S7. XPS O 1*s* spectra of $IrCoO_x$-2@SnO_2.

We attribute the O 1*s* XPS spectra to lattice oxygen (O_L) and metal oxygen (O_M) based on the guidance of corresponding reference (Ram *et al.*, *Science* **2024**, 384, 1373-1380), among which O_M represents oxygen atoms directly bonded to metal ions in a disorder configuration.

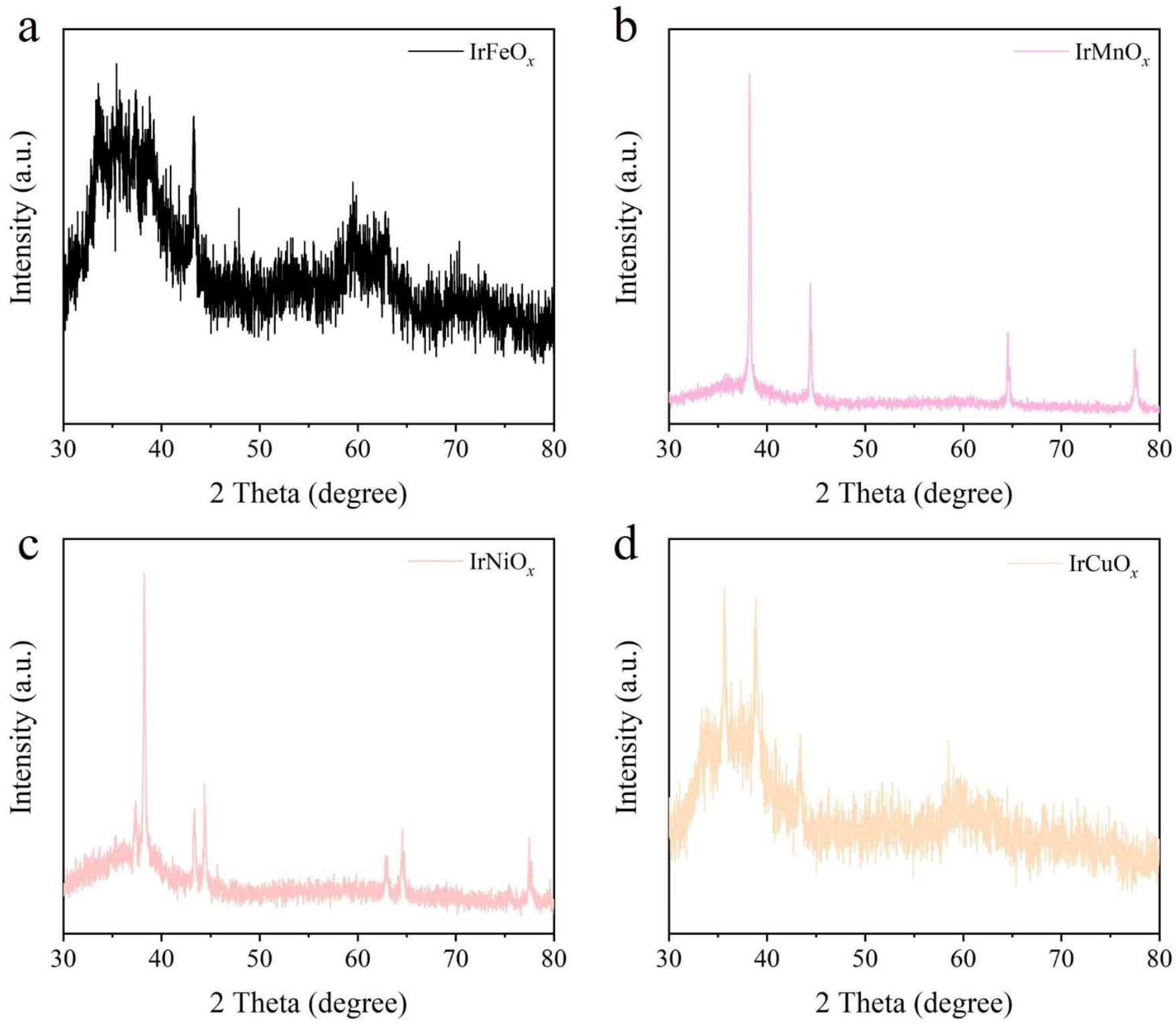

Fig. S8. XRD patterns of (a) $IrFeO_x$, (b) $IrMnO_x$, (c) $IrNiO_x$ and (d) $IrCuO_x$ samples.

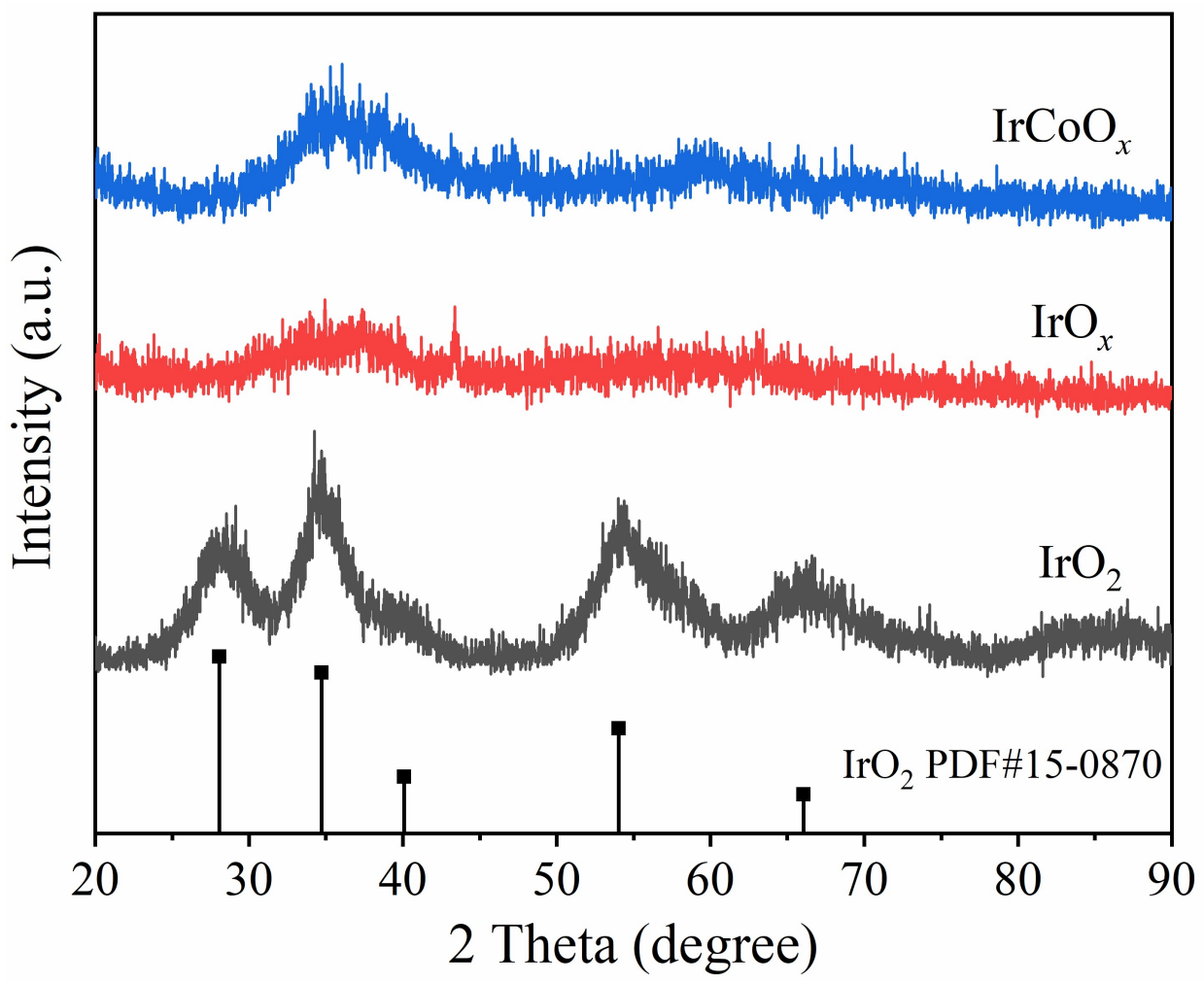

Fig. S9. The comparison of XRD patterns for commercial IrO_2, IrO_x and $IrCoO_x$.

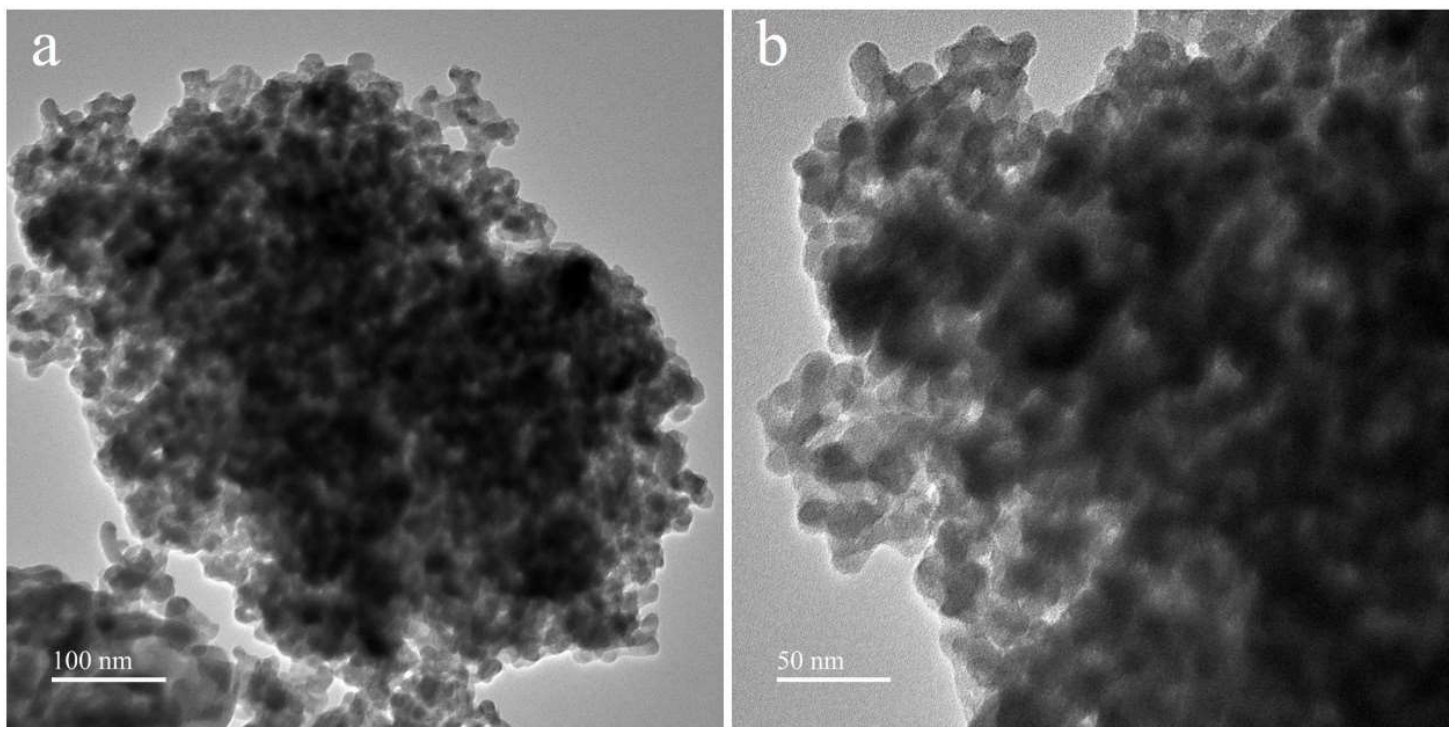

Fig. S10. TEM images of IrO_x sample at different magnifications.

The as-prepared IrO_x sample shows similar morphology with $IrCoO_x$.

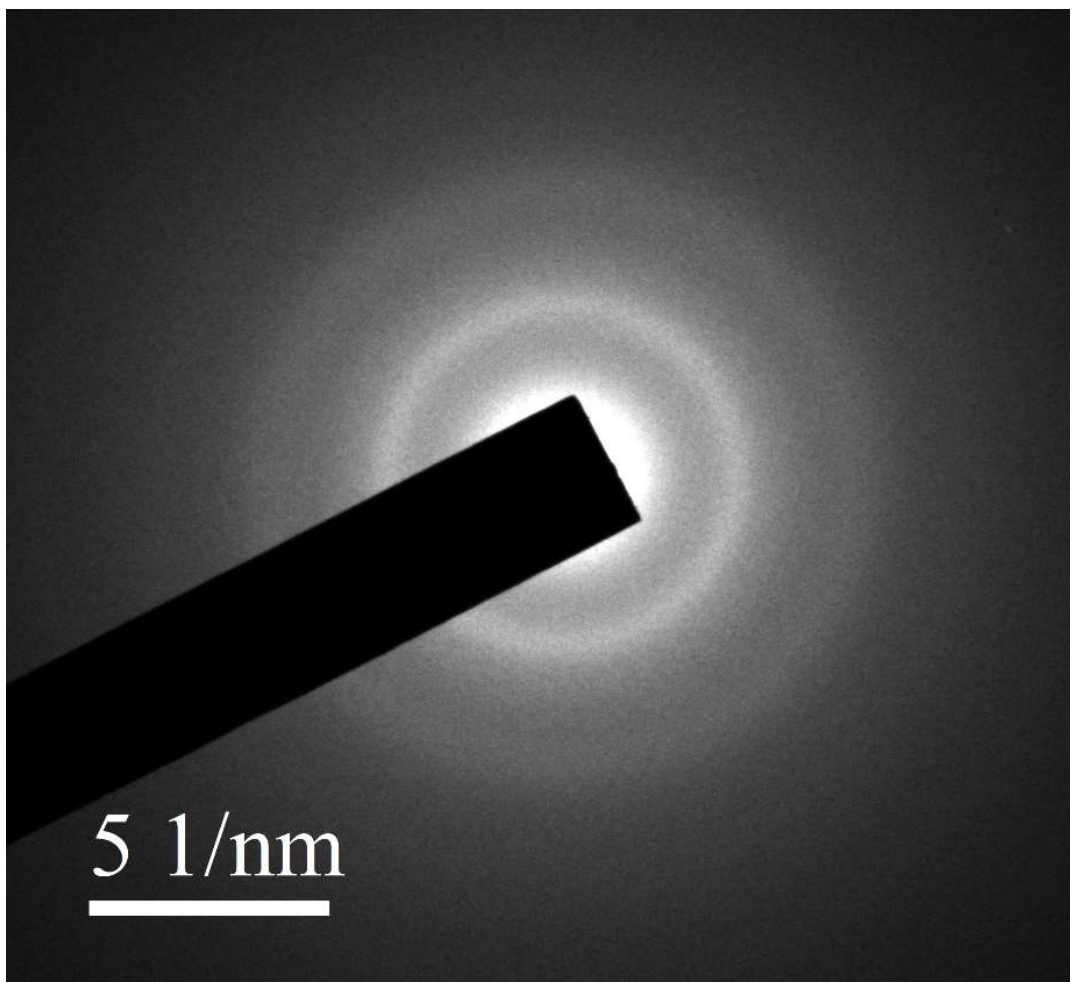

Fig. S11. The SAED pattern of as-prepared IrO_x sample.

Compared with $IrCoO_x$ sample, the sharpness of the diffraction ring is significantly reduced, consistent with amorphous features.

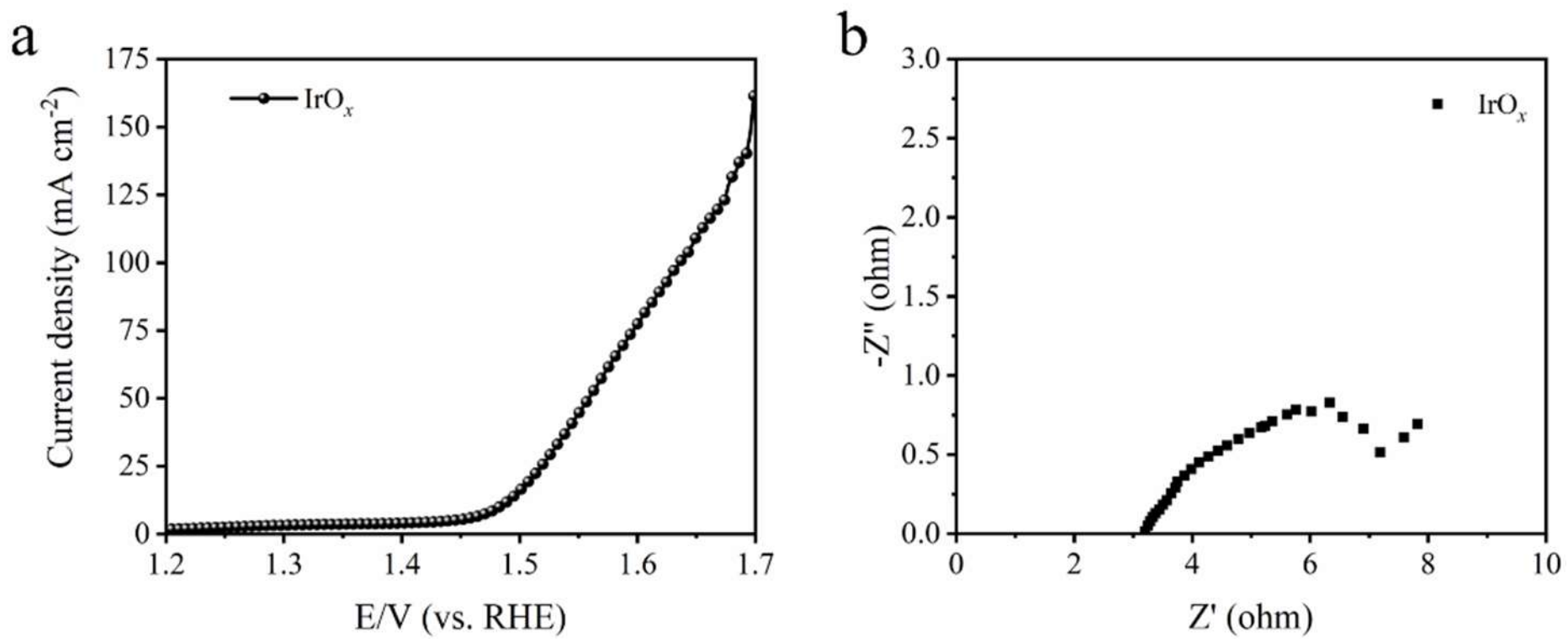

Fig. S12. The LSV curve (a) and ESI curve (b) of as-prepared IrO_x catalyst.

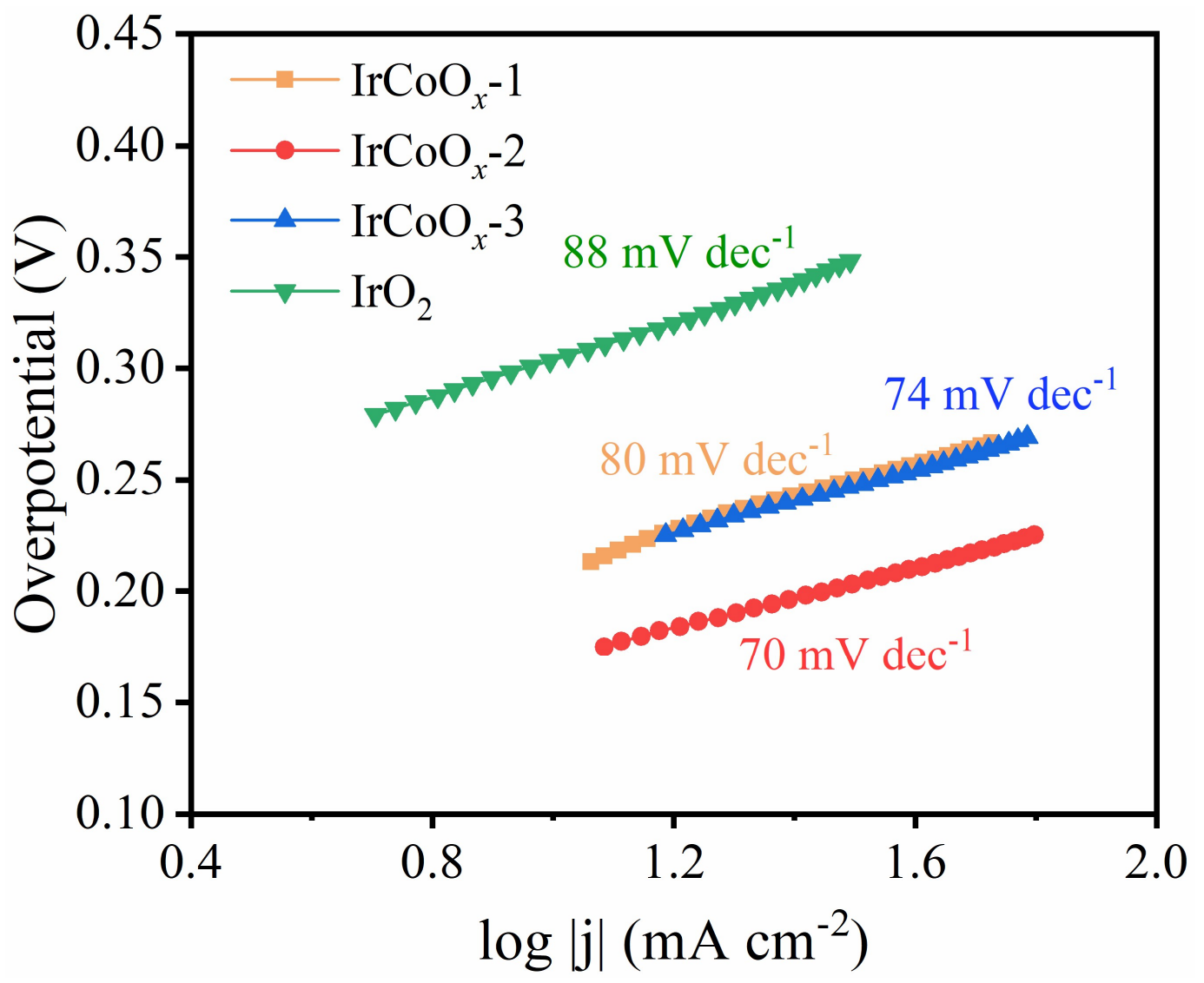

Fig. S13. Tafel slope plots for $IrCoO_x$-1, $IrCoO_x$-2, $IrCoO_x$-3 and IrO_2 catalysts.

$IrCoO_x$-2 exhibits the obviously smallest Tafel slope and the lowest charge-transfer resistance than $IrCoO_x$-1, $IrCoO_x$-3 and commercial IrO_2 (Figs. S13-S14).

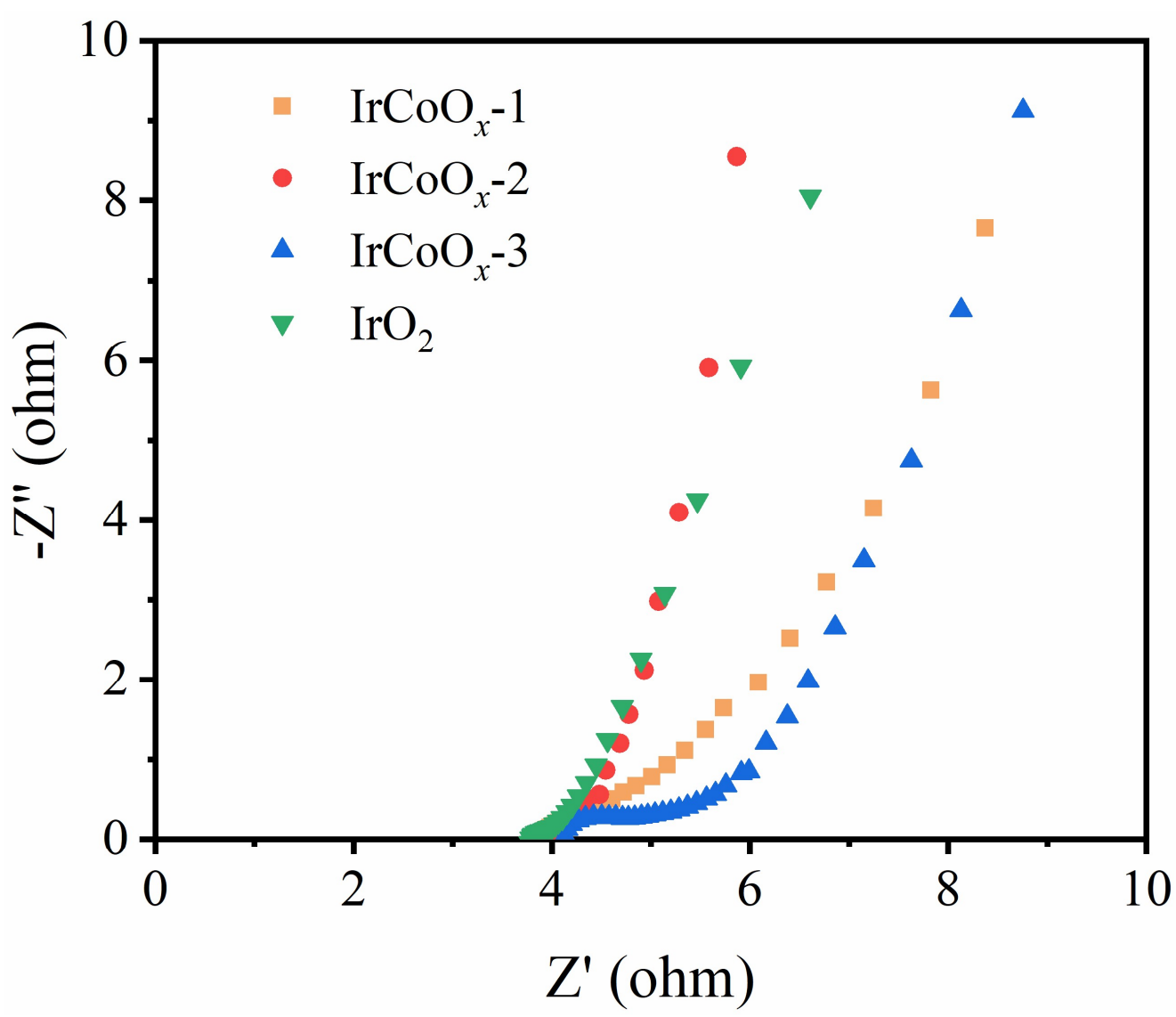

Fig. S14. Nyquist EIS plots for IrCoO$_x$-1, IrCoO$_x$-2, IrCoO$_x$-3 and IrO_2 catalysts.

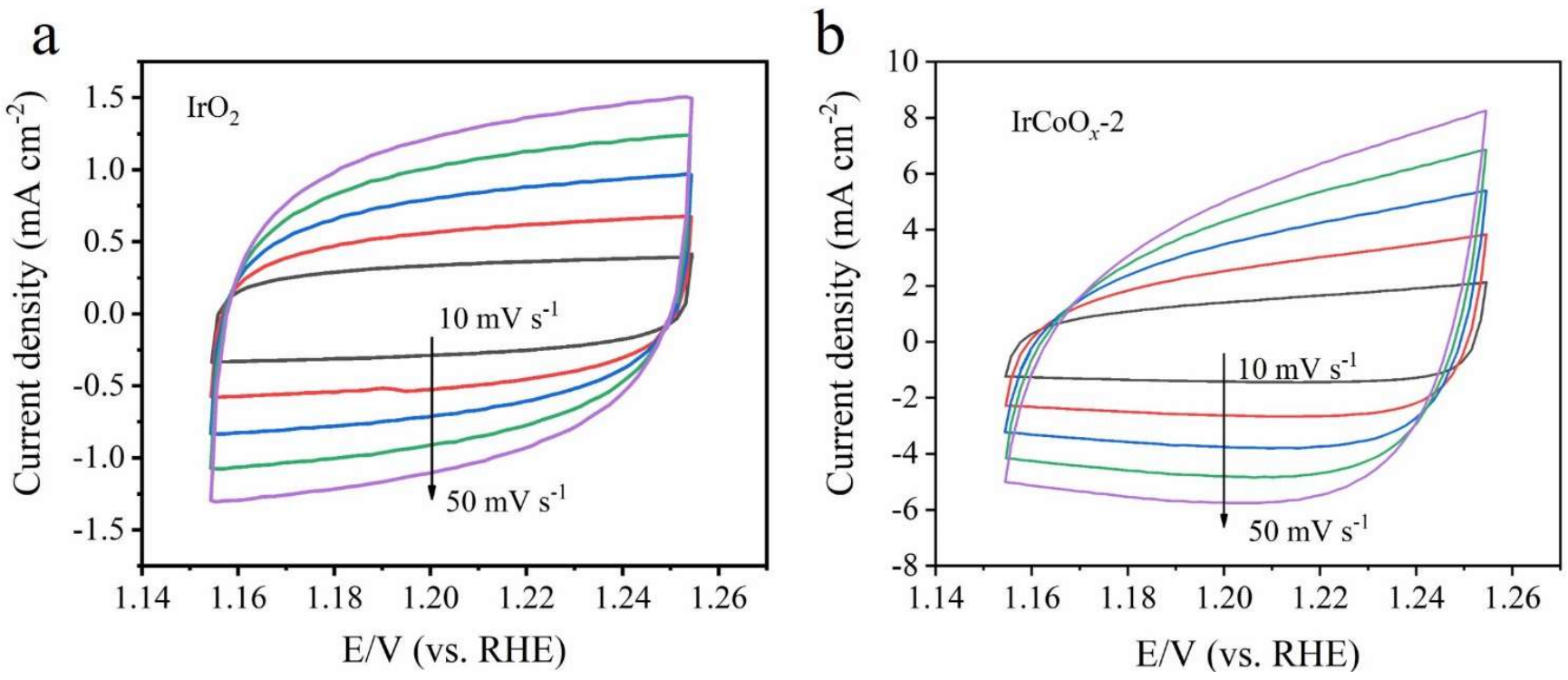

Fig. S15. Cycle voltammogram (CV) curves for (a) IrO_2 and (b) $IrCoO_x$-2 in non-Faraday region at different scan rates (10 mV/s, 20 mV/s, 30 mV/s, 40 mV/s and 50 mV/s).

$IrCoO_x$-2 (Figs. S15-S16) exhibits higher double-layer capacitance (C_{dl}) and electrochemical active surface area (ECSA) than commercial IrO_2 (98 mF cm^{-2} vs. 21 mF cm^{-2}), which can be attributed to higher disorder degree for paracrystalline $IrCoO_x$-2.

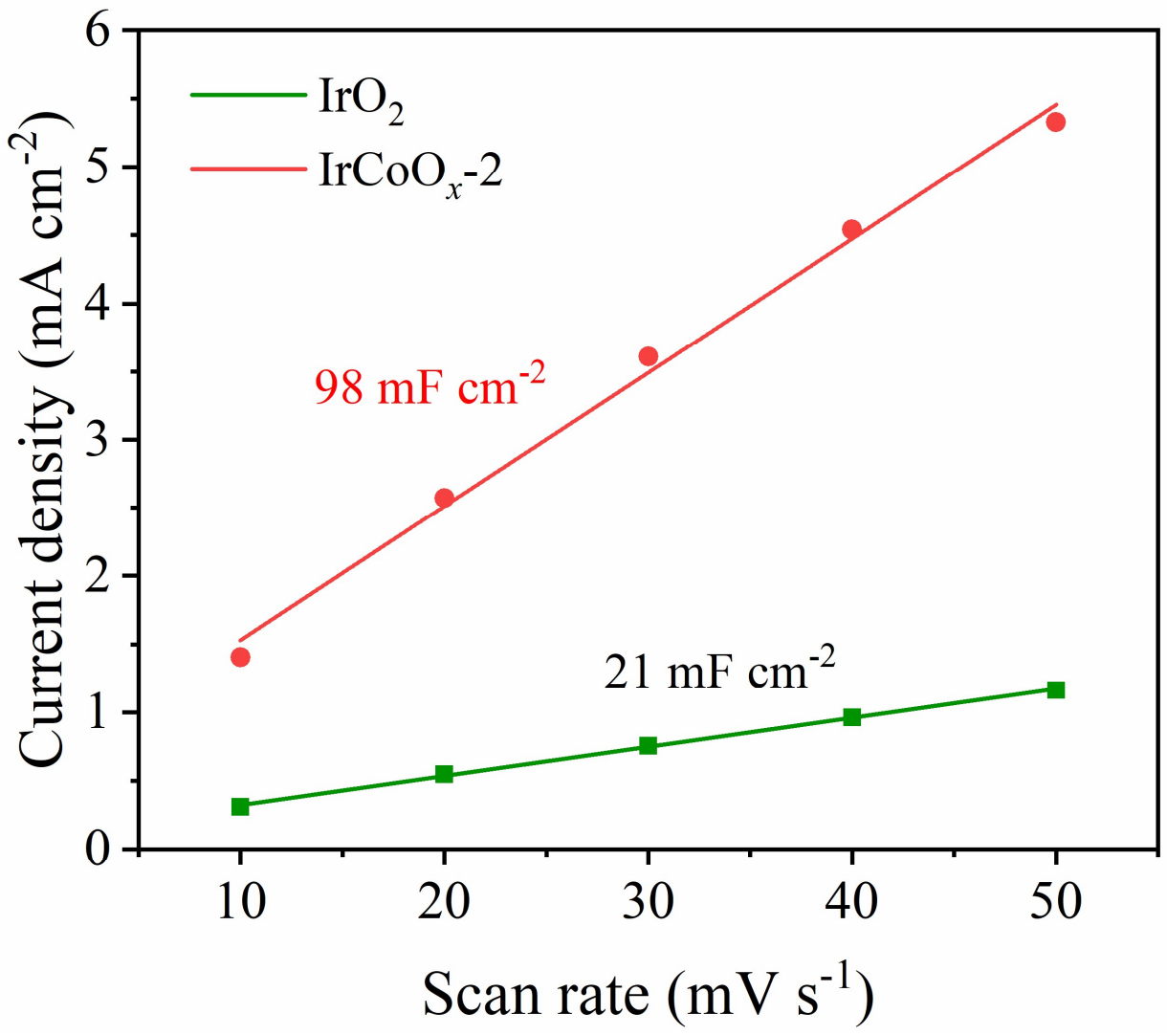

Fig. S16. The comparison of electrochemical double layer capacitance (C_{dl}) for IrO_2 and $IrCoO_x$-2.

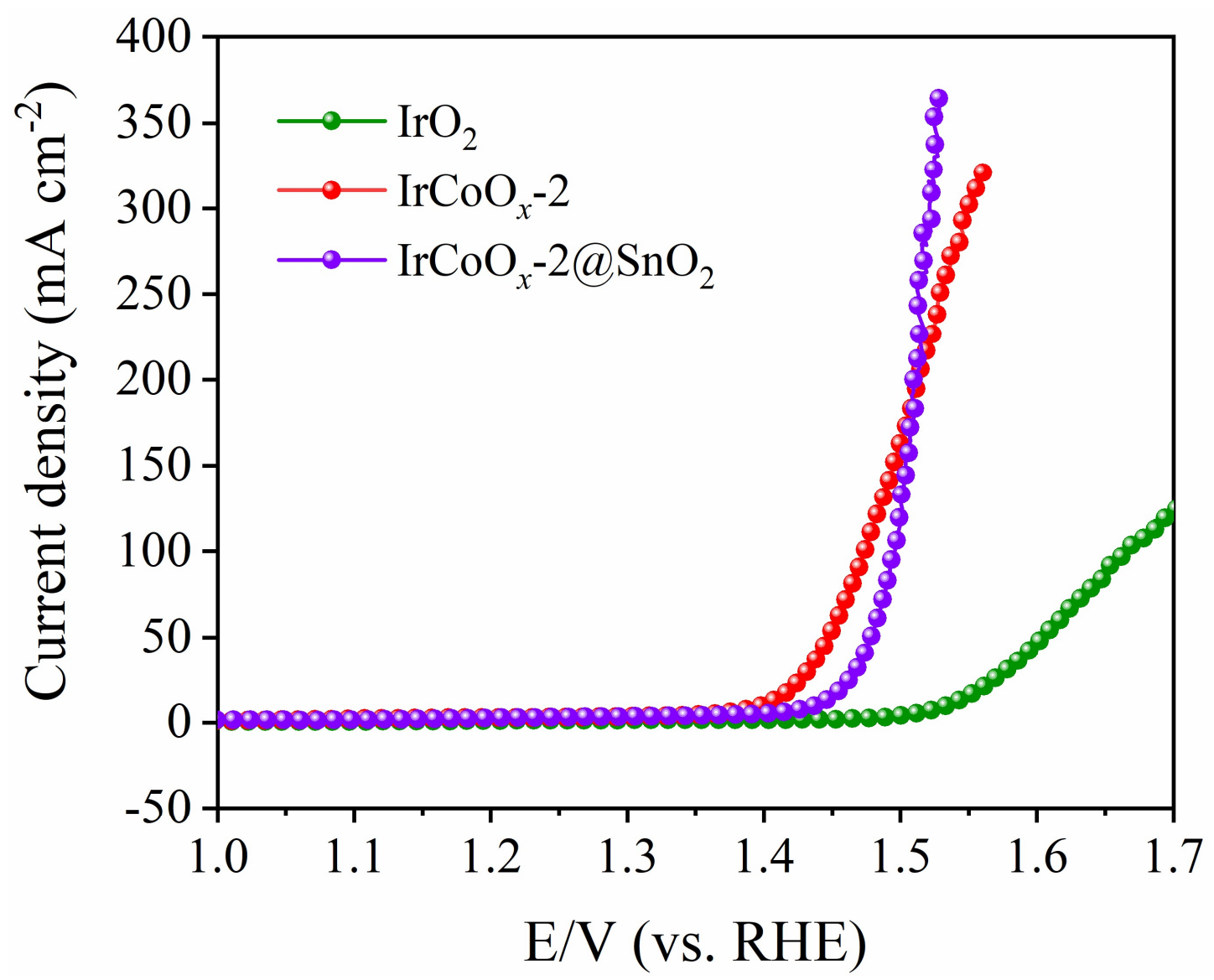

Fig. S17. LSV curves of IrO_2, $IrCoO_x$-2 and $IrCoO_x$-2@SnO_2 catalysts.

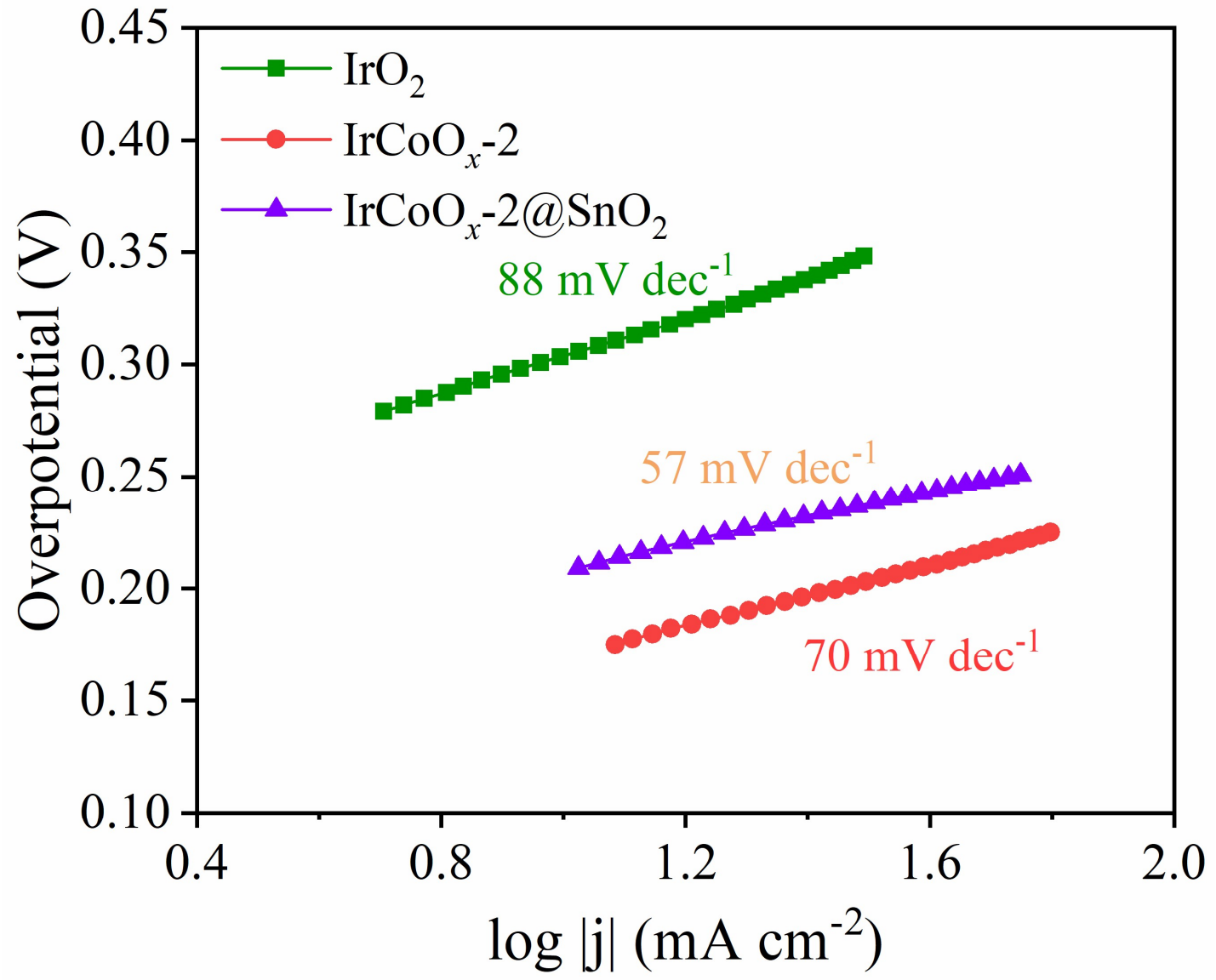

Fig. S18. Tafel slope plots for IrO_2, $IrCoO_x$-2 and $IrCoO_x$-2@SnO_2 catalysts.

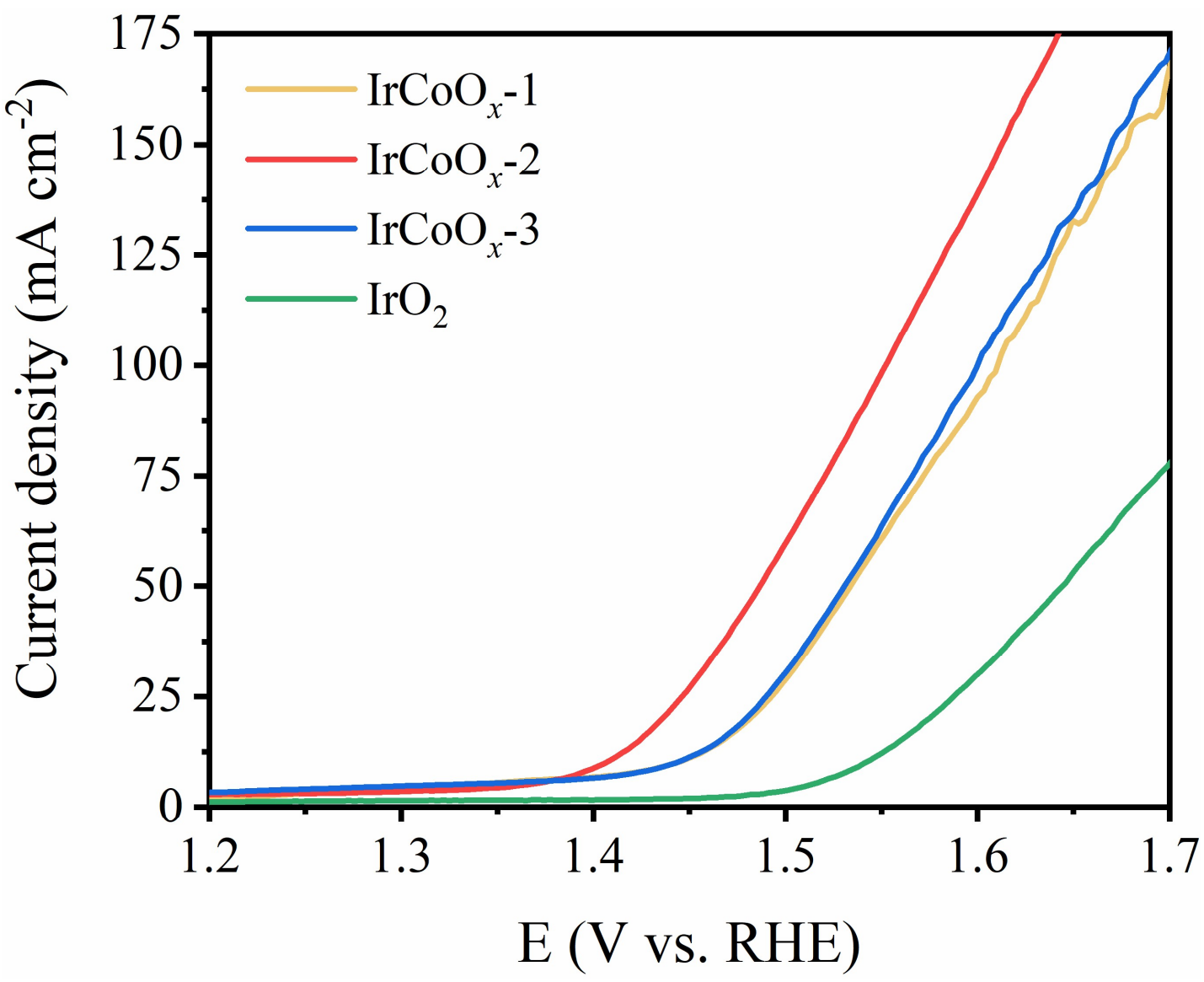

Fig. S19. LSV curves of $IrCoO_x$-1, $IrCoO_x$-2, $IrCoO_x$-3 and commercial IrO_2 catalysts with non-iR correction in 0.5 M H_2SO_4.

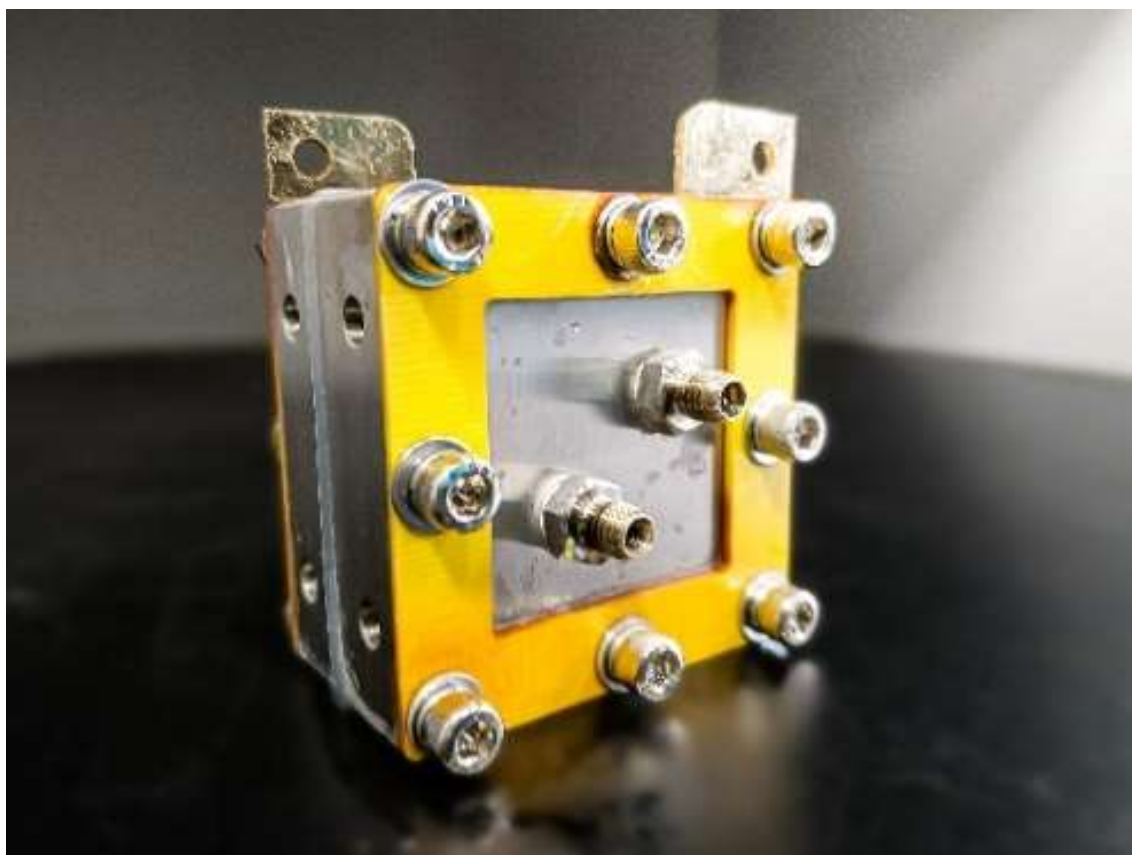

Fig. S20. The photograph of the PEM electrolyzer with a working area of 4 cm^2.

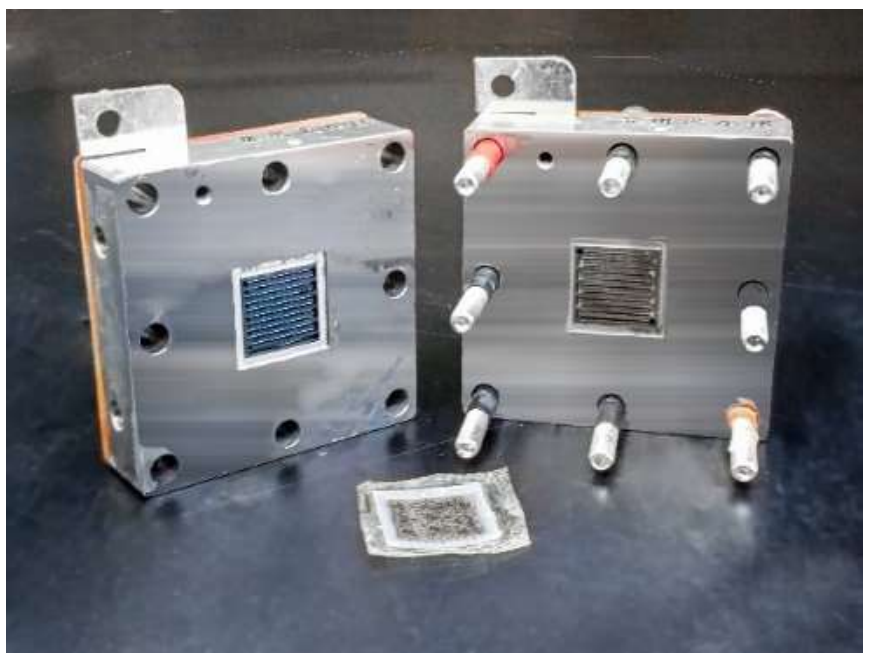

Fig. S21. The photograph of the detached PEM electrolyzer using as-prepared $IrCoO_x$-2@SnO_2 and commercial 40 wt. % Pt/C as anode and cathode catalysts respectively.

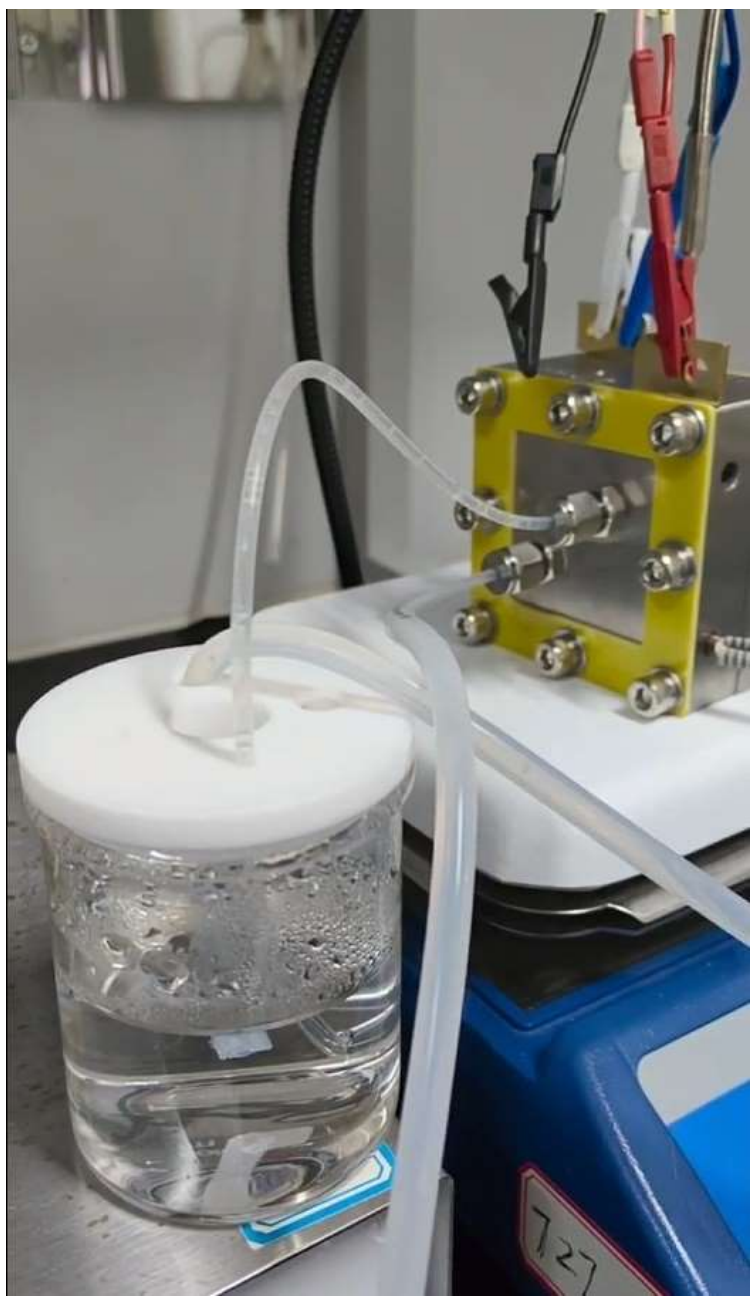

Fig. S22. The photograph of the PEM electrolyzer at working condition.

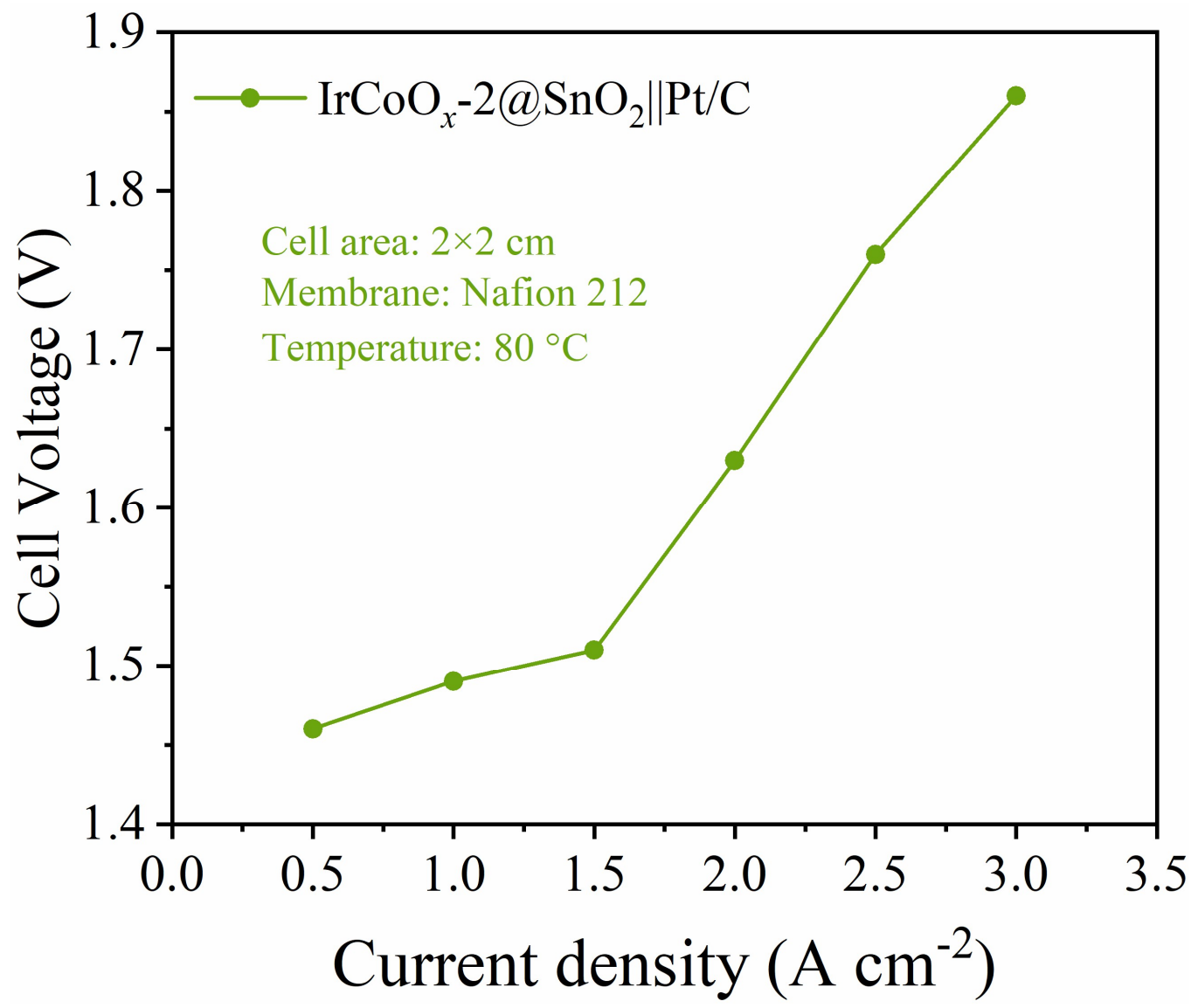

Fig. S23. Polarization curve of the PEM electrolyzer using $IrCoO_x$-2@SnO_2 and commercial 40 wt. % Pt/C as anode and cathode catalysts, respectively.

From the polarization curve (Fig. S22), the electrolyzer can reach a high current density of 2 A cm^{-2} at only 1.63 V with a working area of 4 cm^2.

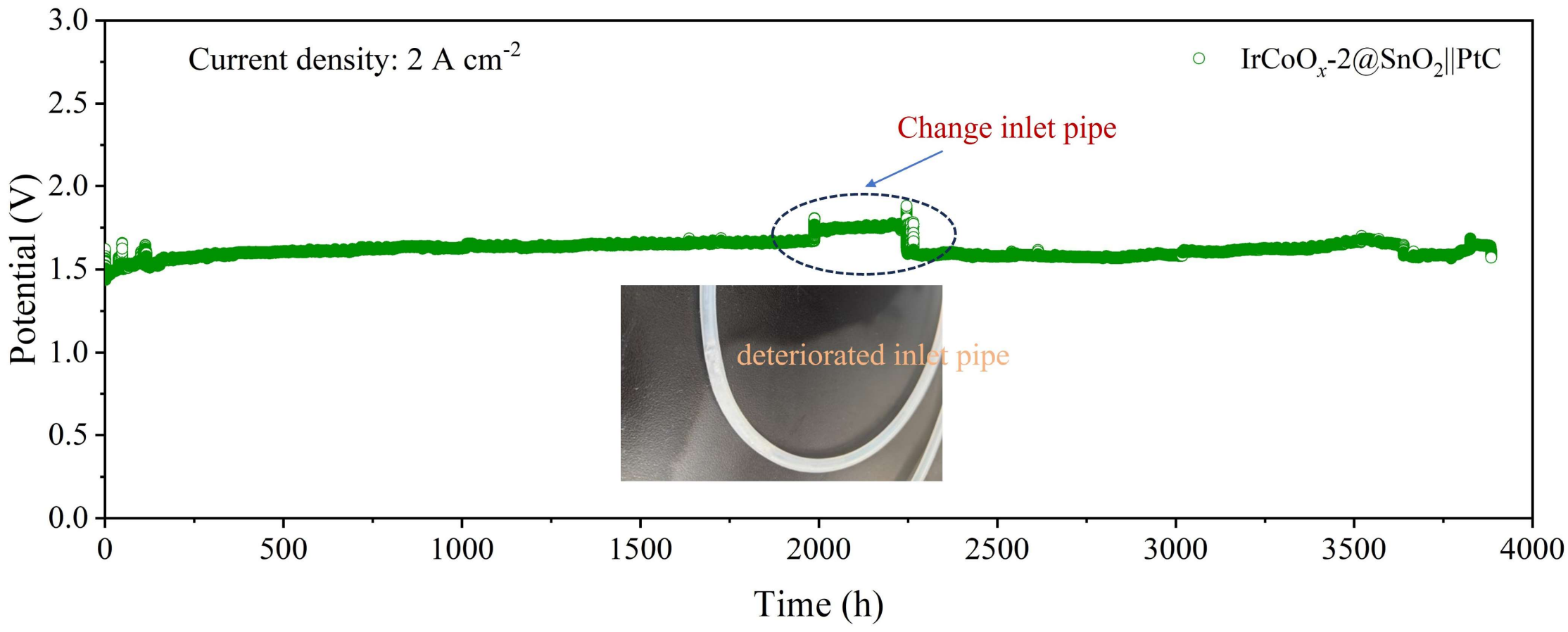

Fig. S24. The original V-t curve at 2.0 A cm^{-2} in a PEMWE electrolyzer using $IrCoO_x$-2@SnO_2 and commercial Pt/C as anode and cathode catalysts (Inset: The photograph for the deterioration of inlet pipe).

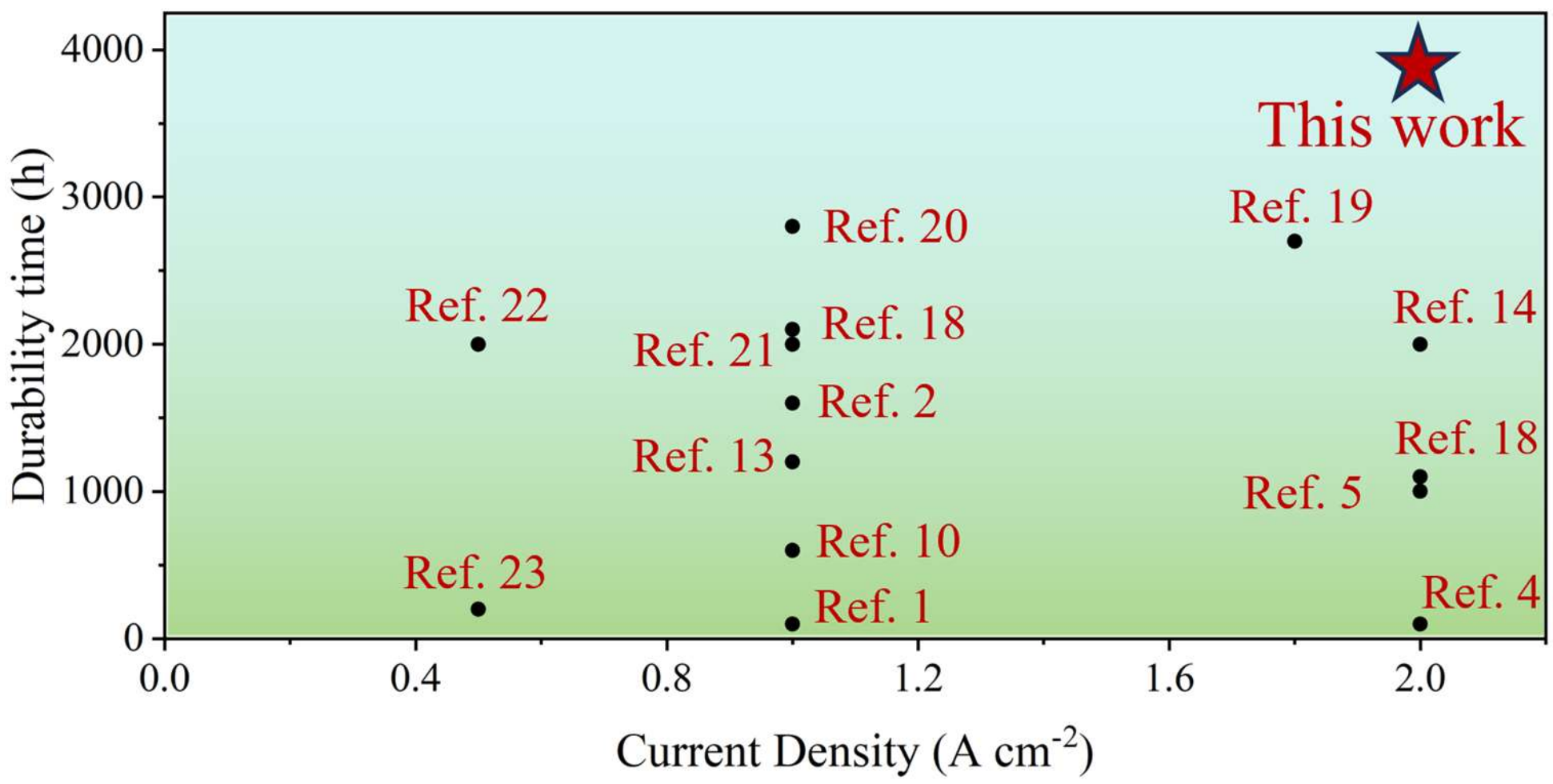

Fig. S25. The comparison of the PEM operational stability between $IrCoO_x$-2@SnO_2 and recent-reported Ir/Ru-based anode catalysts (The numbers in the figure corresponds to the reference numbers in the Supplementary Information).

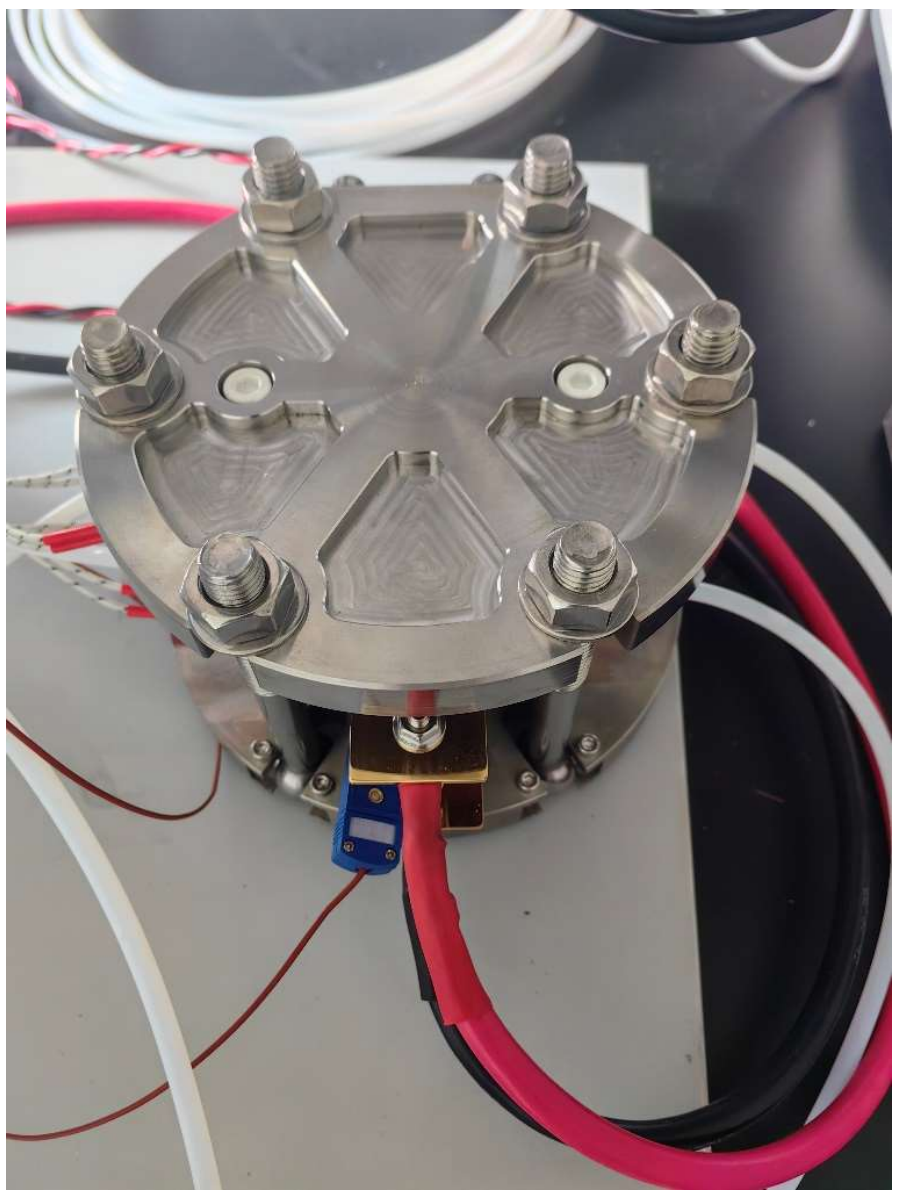

Fig. S26. The photograph of the standard PEM electrolyzer with the working area of 25 cm^2.

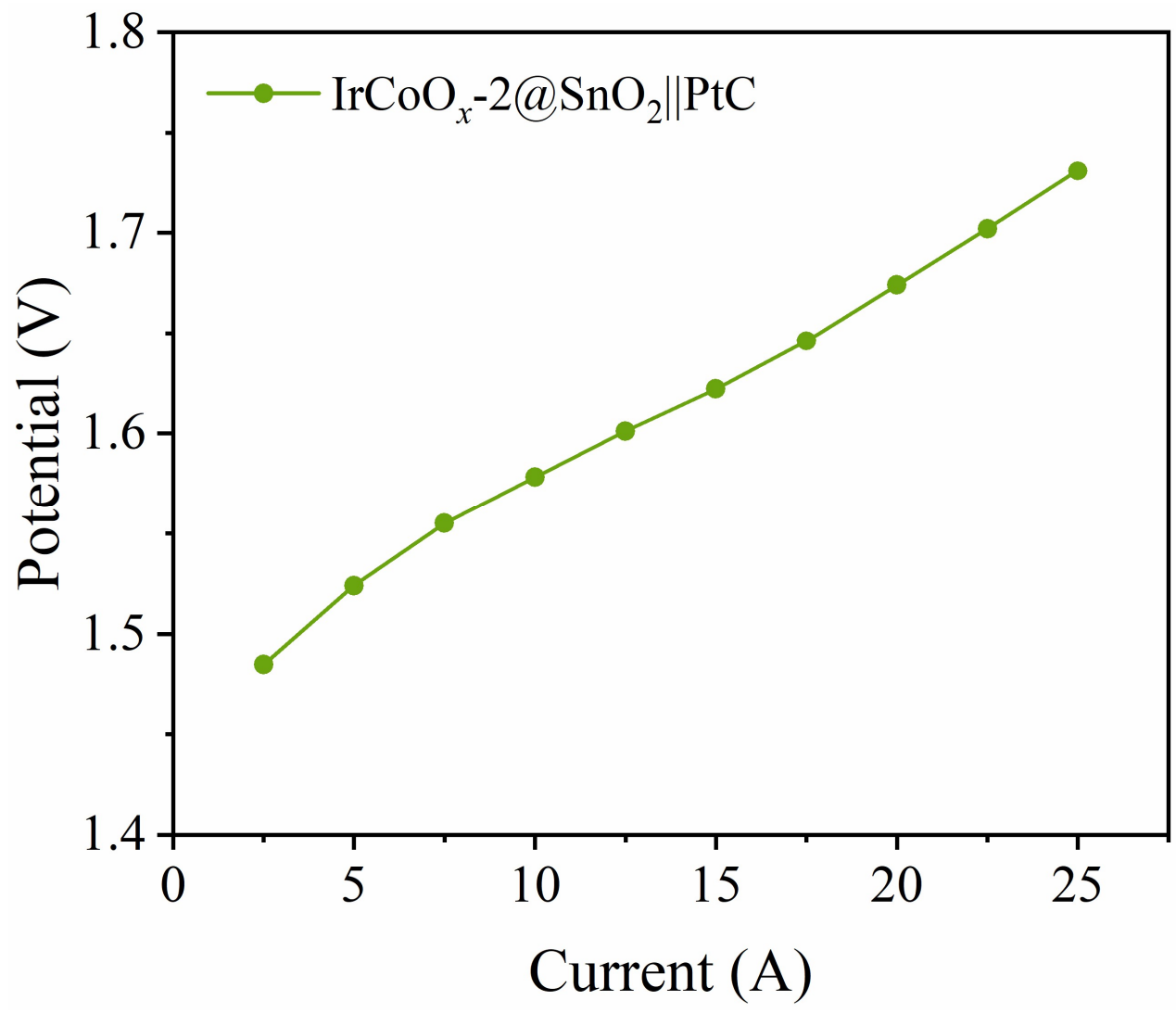

Fig. S27. Polarization curve of the standard PEM electrolyzer with the working area of 25 cm^2.

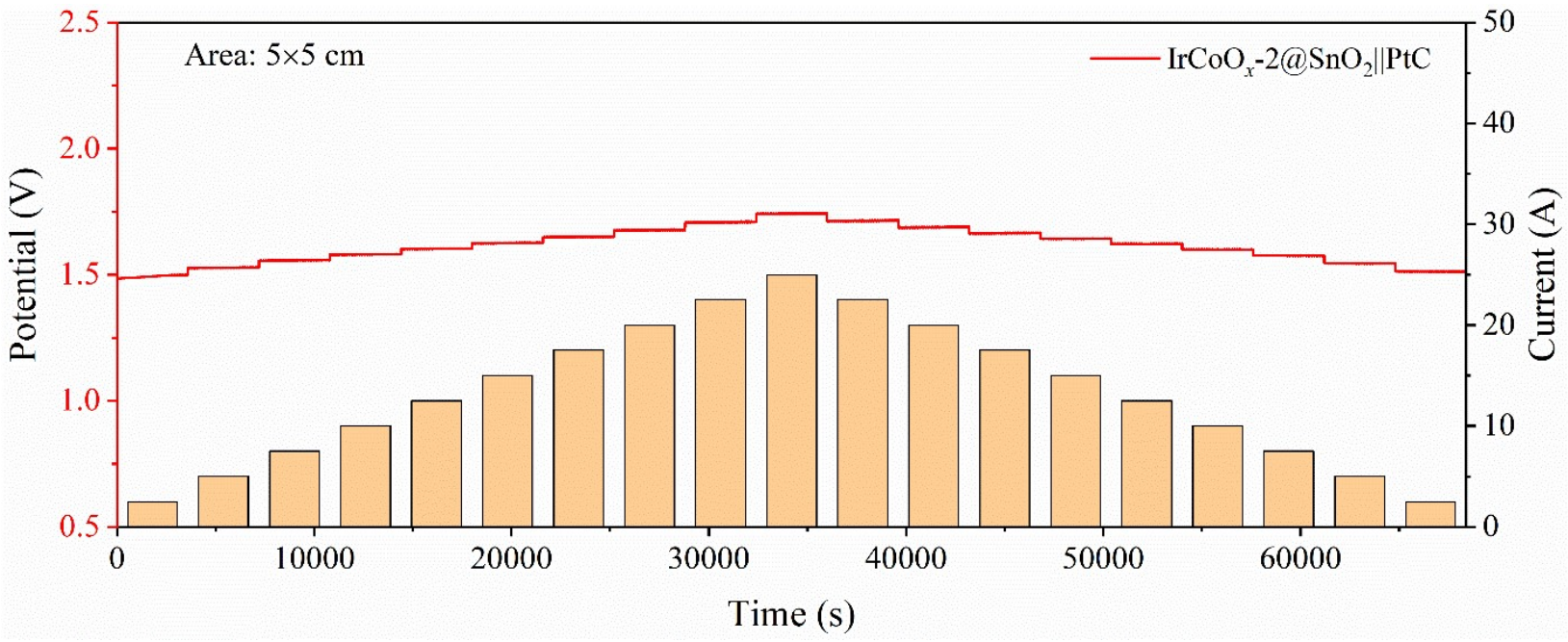

Fig. S28. The stability curves of standard PEM electrolyzer (25 cm^2) at the current of 2.5, 5, 7.5, 10, 12.5, 15, 17.5, 20, 22.5 and 25 A, respectively.

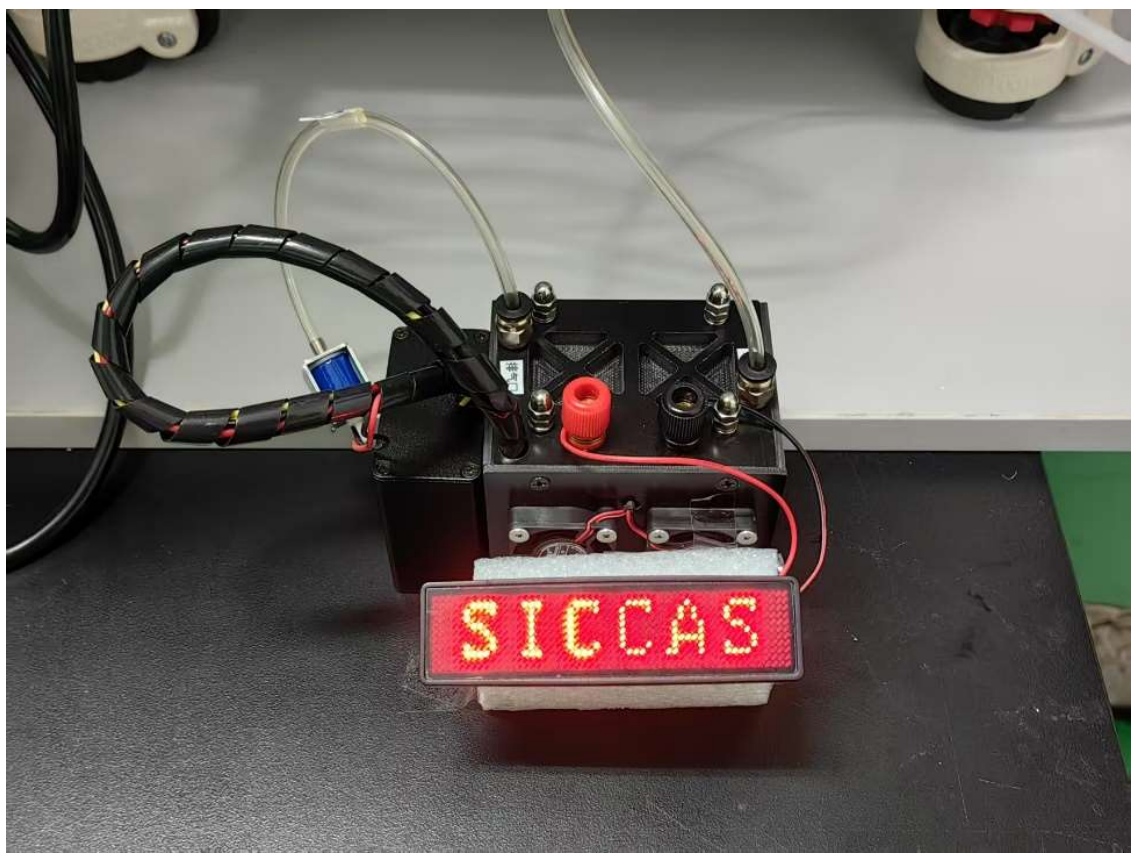

Fig. S29. The photograph of the LED panel by constructing water splitting-fuel cell linkage devices.

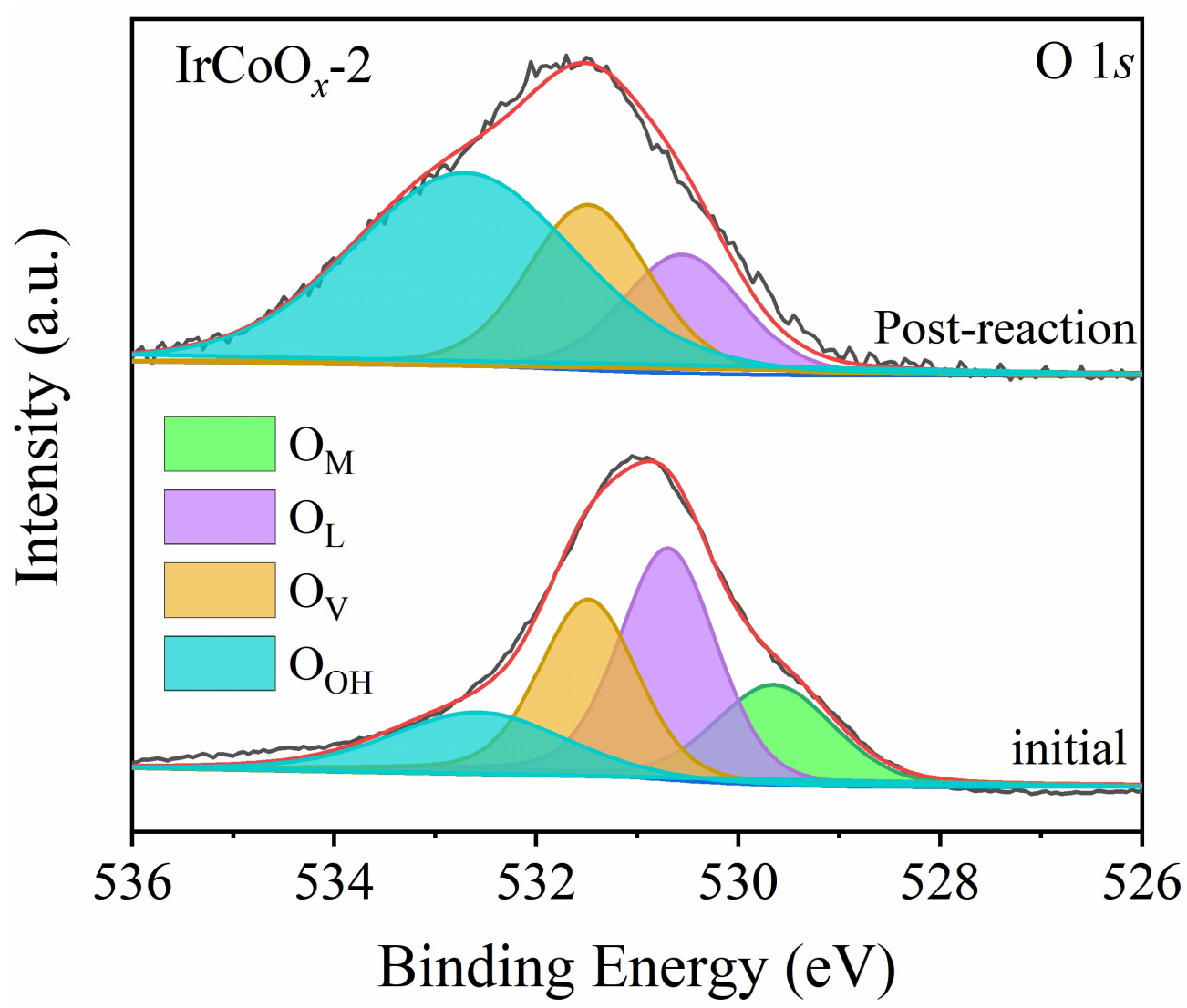

Fig. S30. The comparison of XPS O 1s spectra before and after OER reaction for IrCoO$_x$-2.

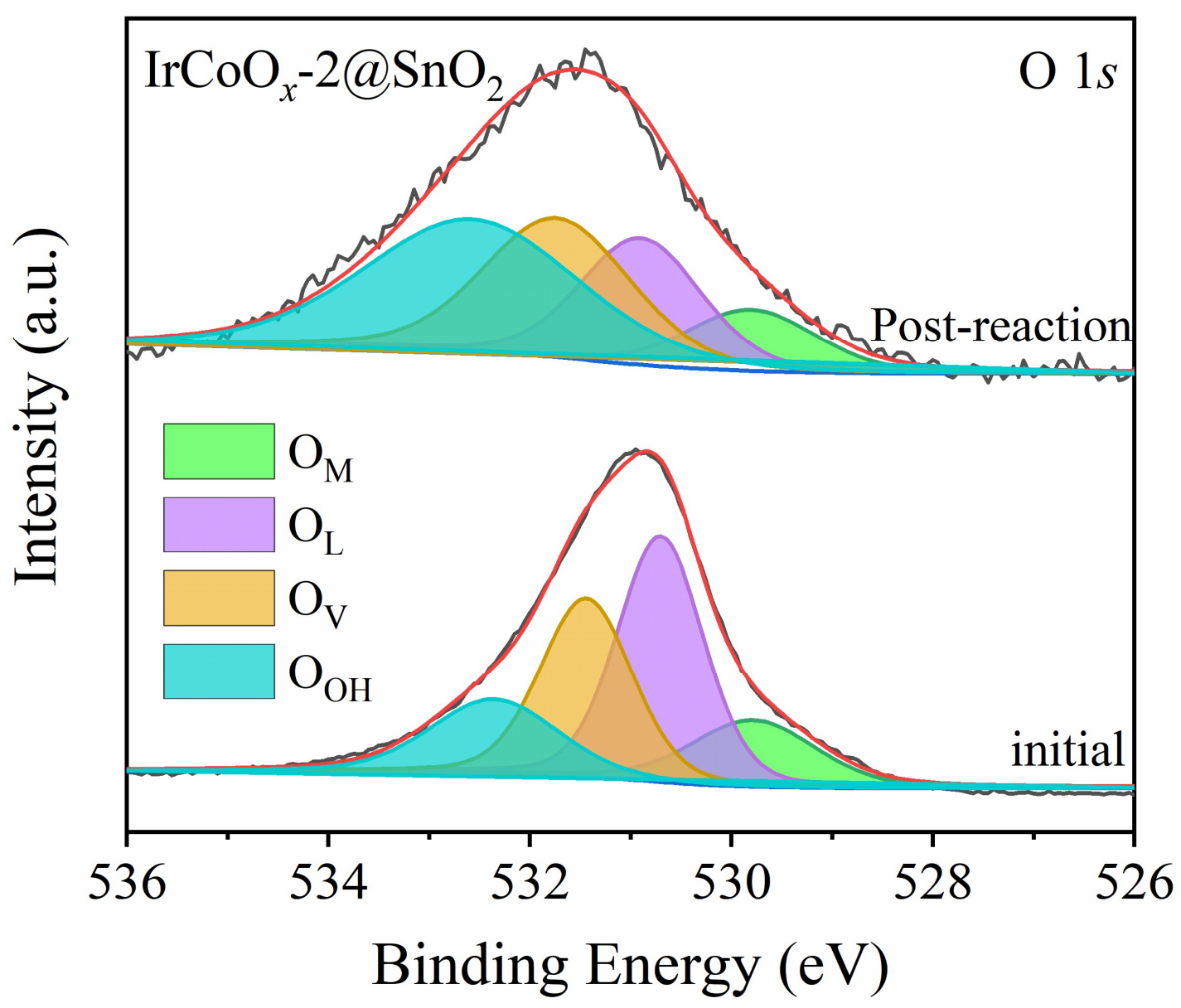

Fig. S31. The comparison of XPS O 1s spectra before and after OER reaction for IrCoO$_x$-2@SnO$_2$.

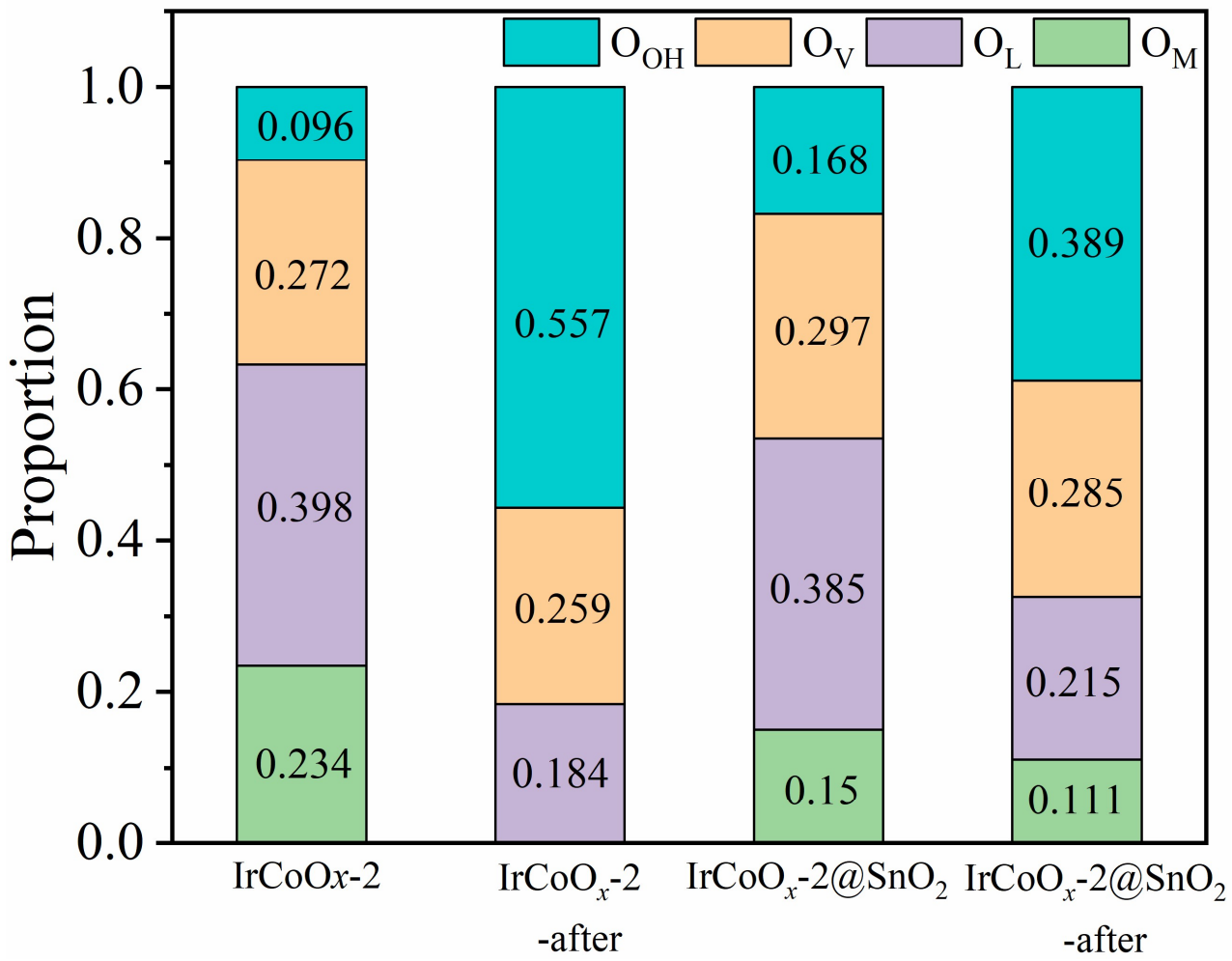

Fig. S32. The proportion of O_{OH}, O_V, O_L, O_M species for IrCoO$_x$-2, IrCoO$_x$-2-after, IrCoO$_x$-2@SnO$_2$ and IrCoO$_x$-2@SnO$_2$-after.

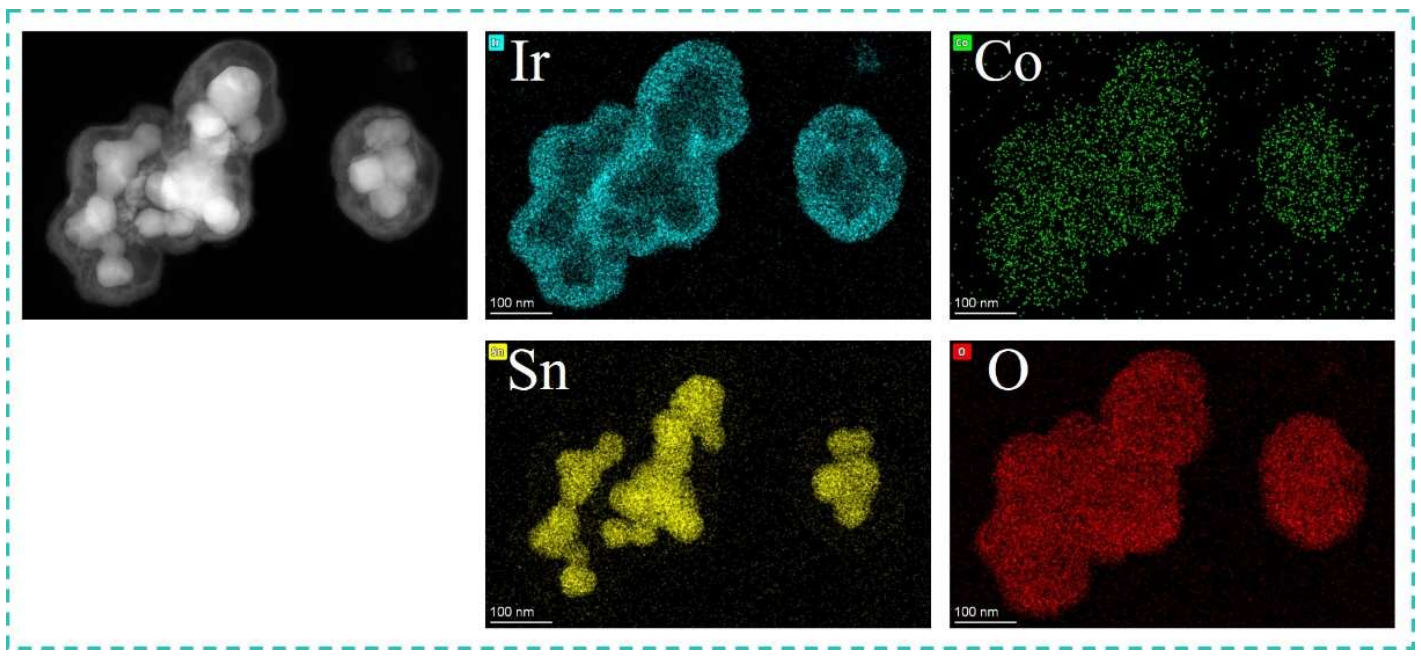

Fig. S33. STEM-EDS elemental mapping of $IrCoO_x$-2@SnO_2-after.

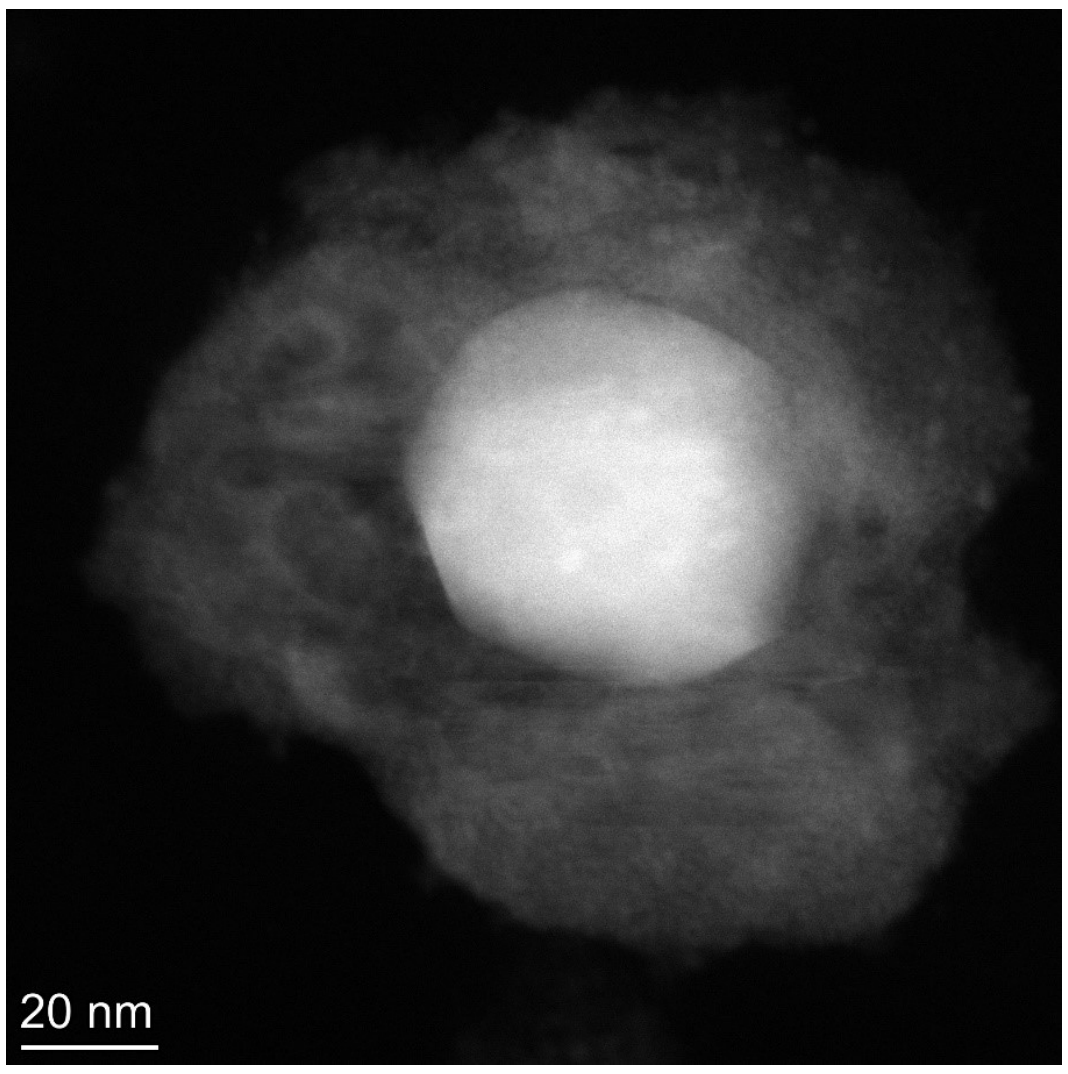

Fig. S34. The yolk-like STEM-HAADF image of $IrCoO_x$-2@SnO_2-after.

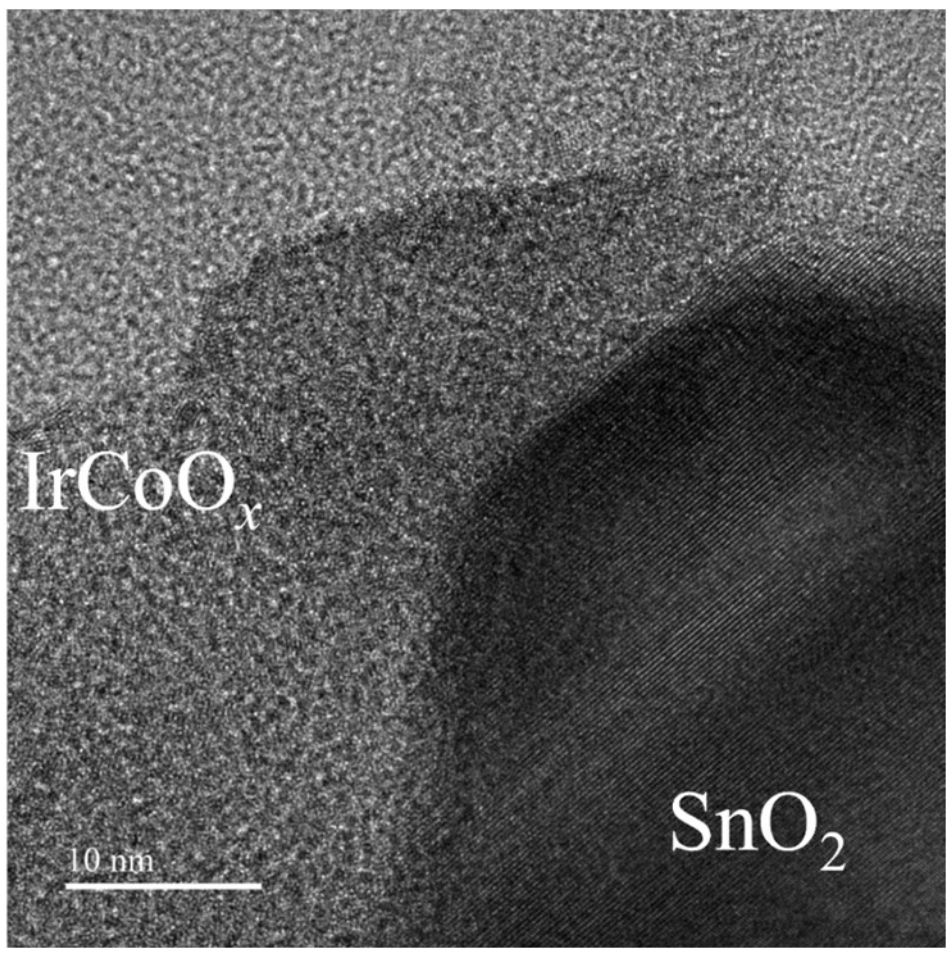

Fig. S35. The TEM image of IrCoO$_x$-2@SnO$_2$-after.

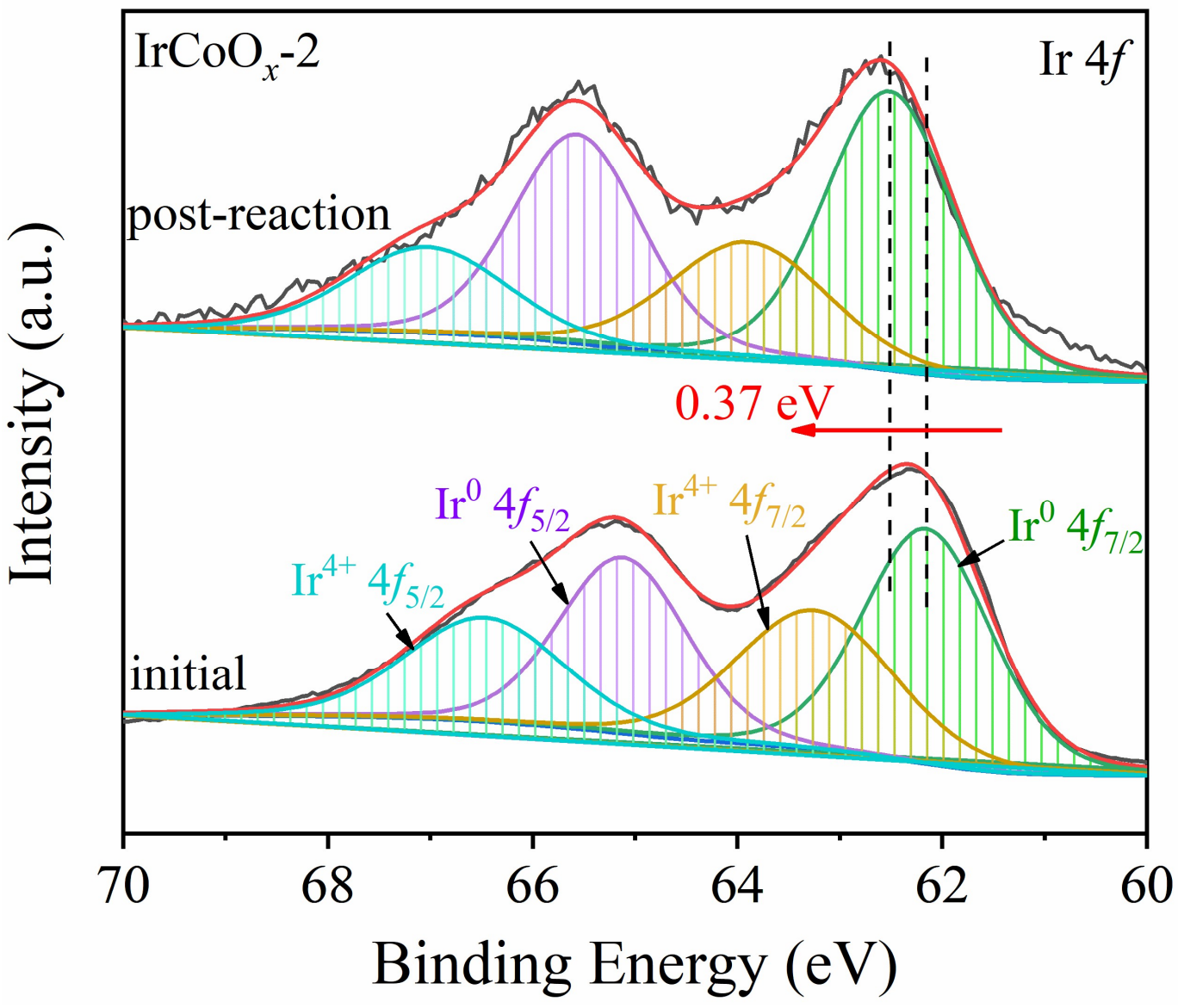

Fig. S36. The comparison of XPS Ir 4*f* spectra before and after OER reaction for $IrCoO_x$-2.

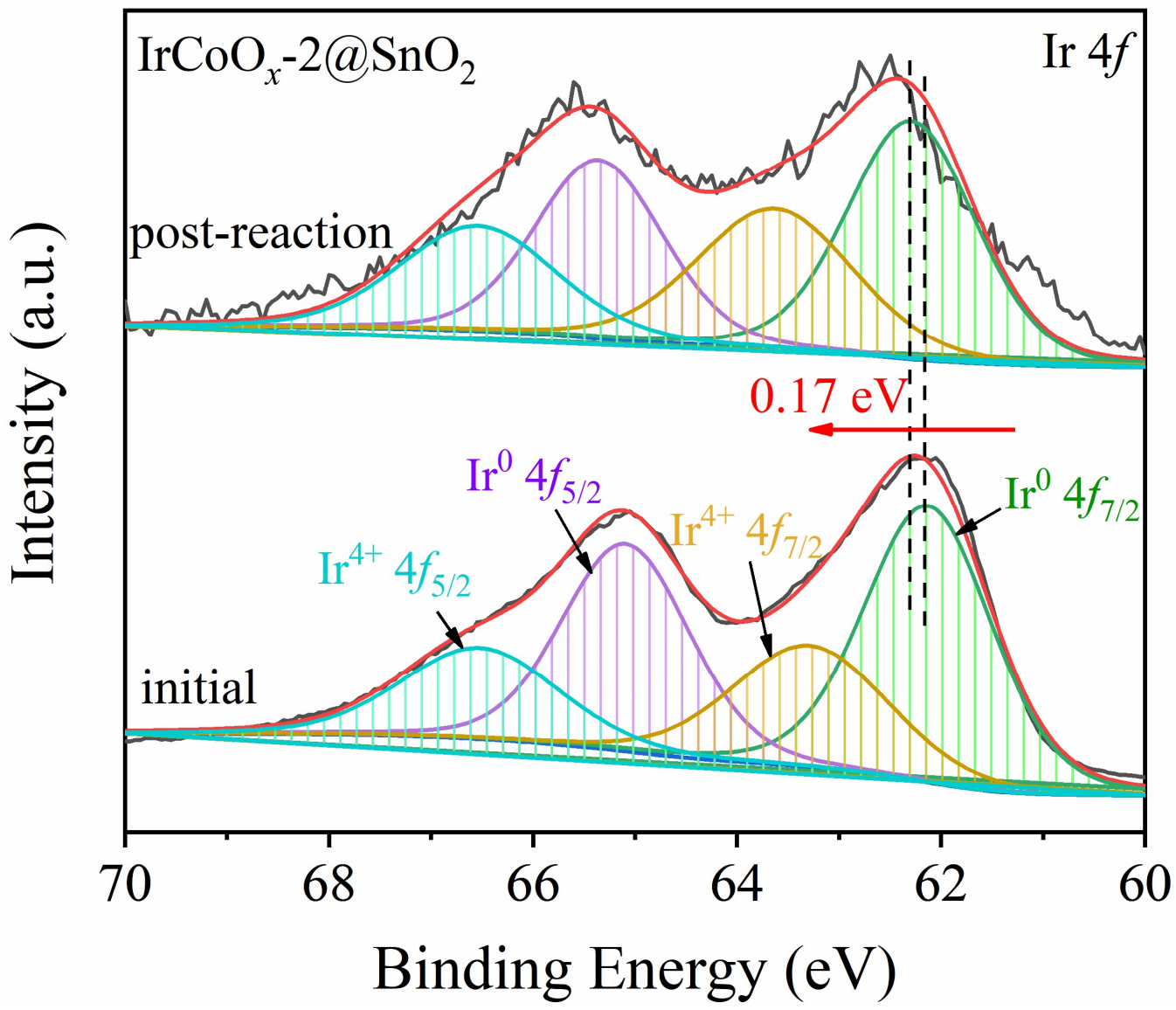

Fig. S37. The comparison of XPS Ir 4*f* spectra before and after OER reaction for $IrCoO_x$-2@SnO_2.

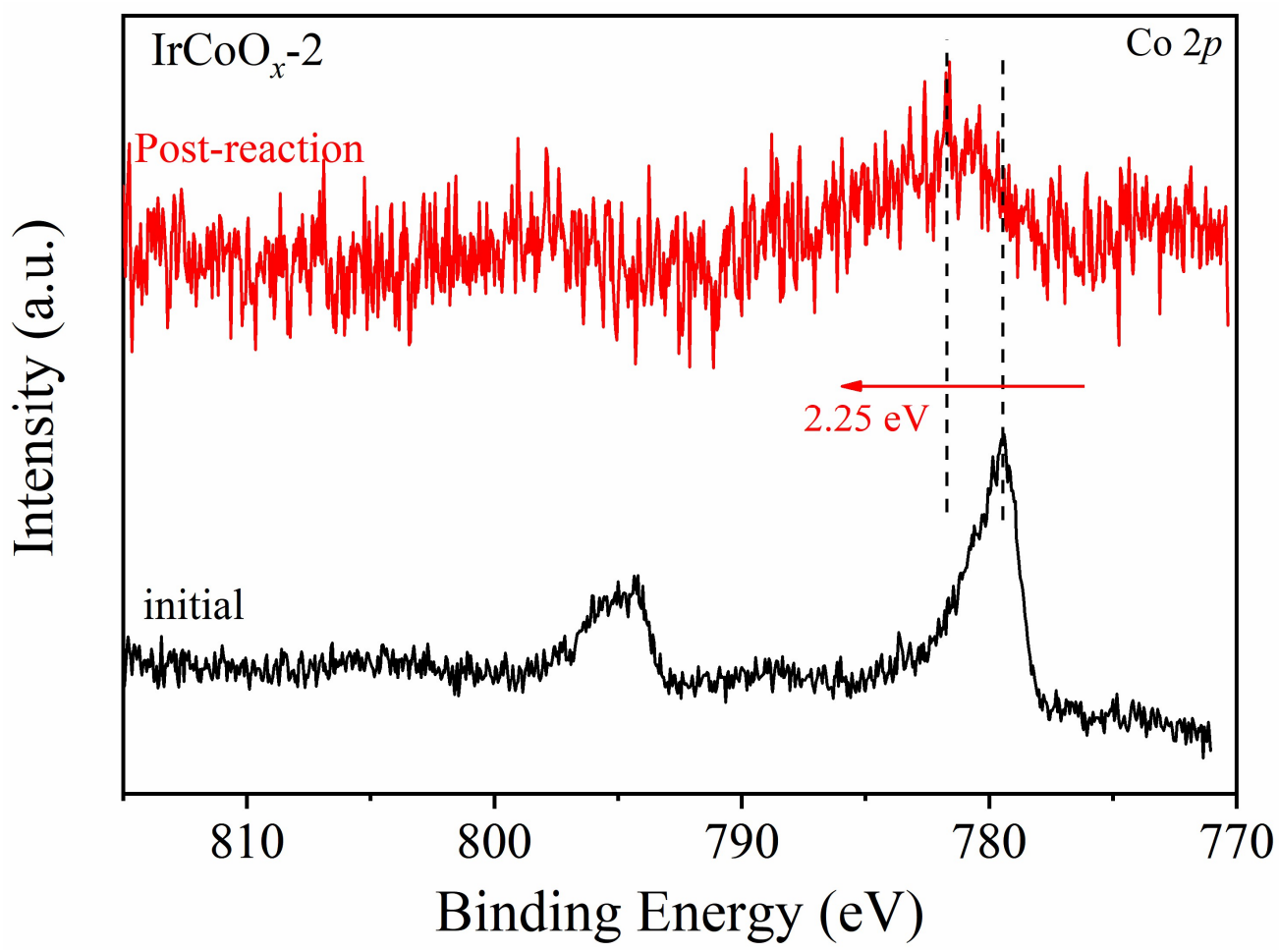

Fig. S38. The comparison of XPS Co 2p spectra for IrCoO$_x$-2 before and after the reaction.

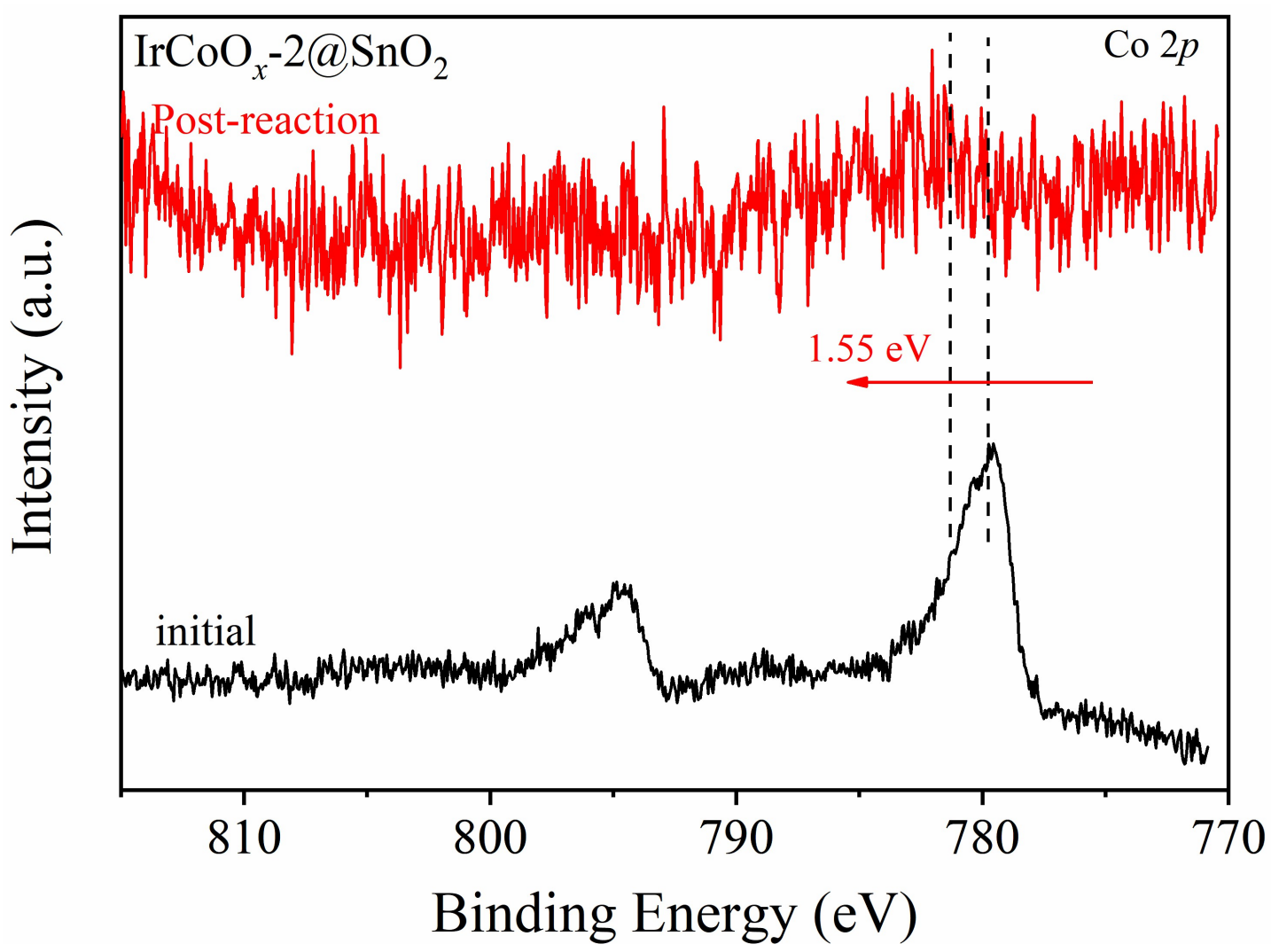

Fig. S39. The comparison of XPS Co 2p spectra for IrCoO$_x$-2@SnO$_2$ before and after the reaction.

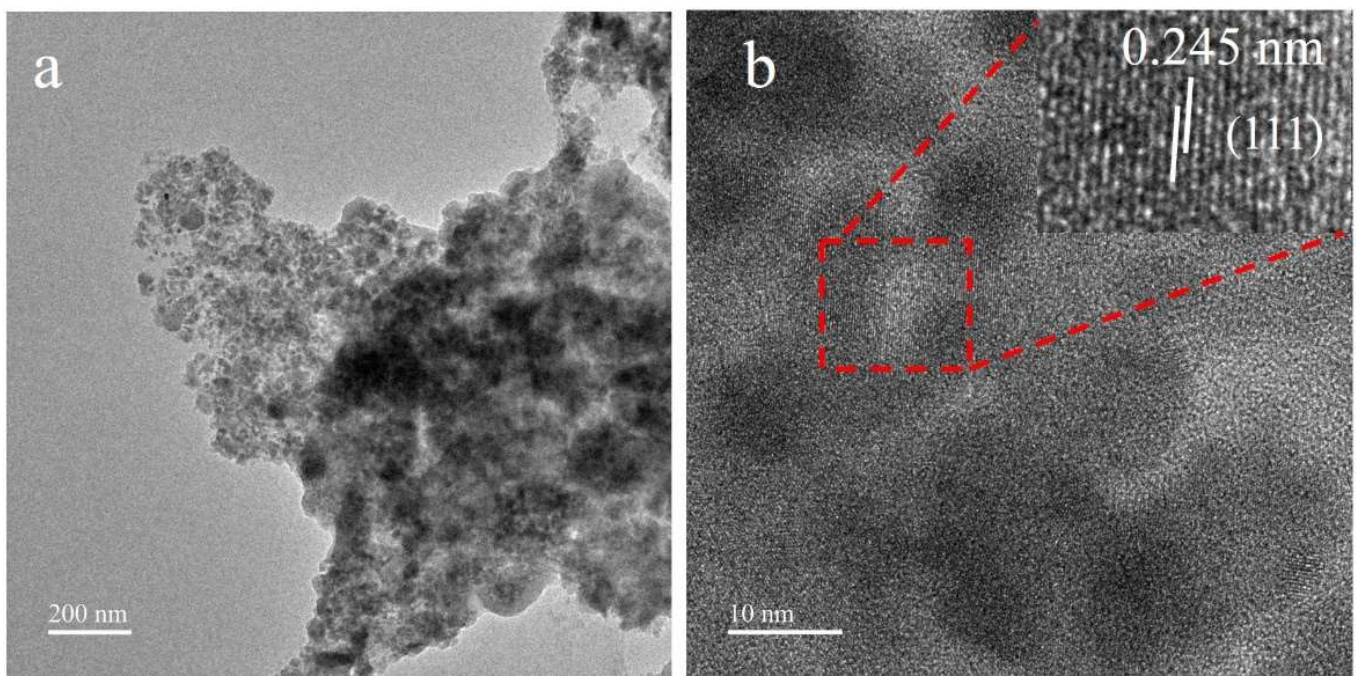

Fig. S40. The TEM images of $IrCoO_x$-2-after at different magnifications.

The clear lattice fringe is attributed to (111) plane of CoO-225.

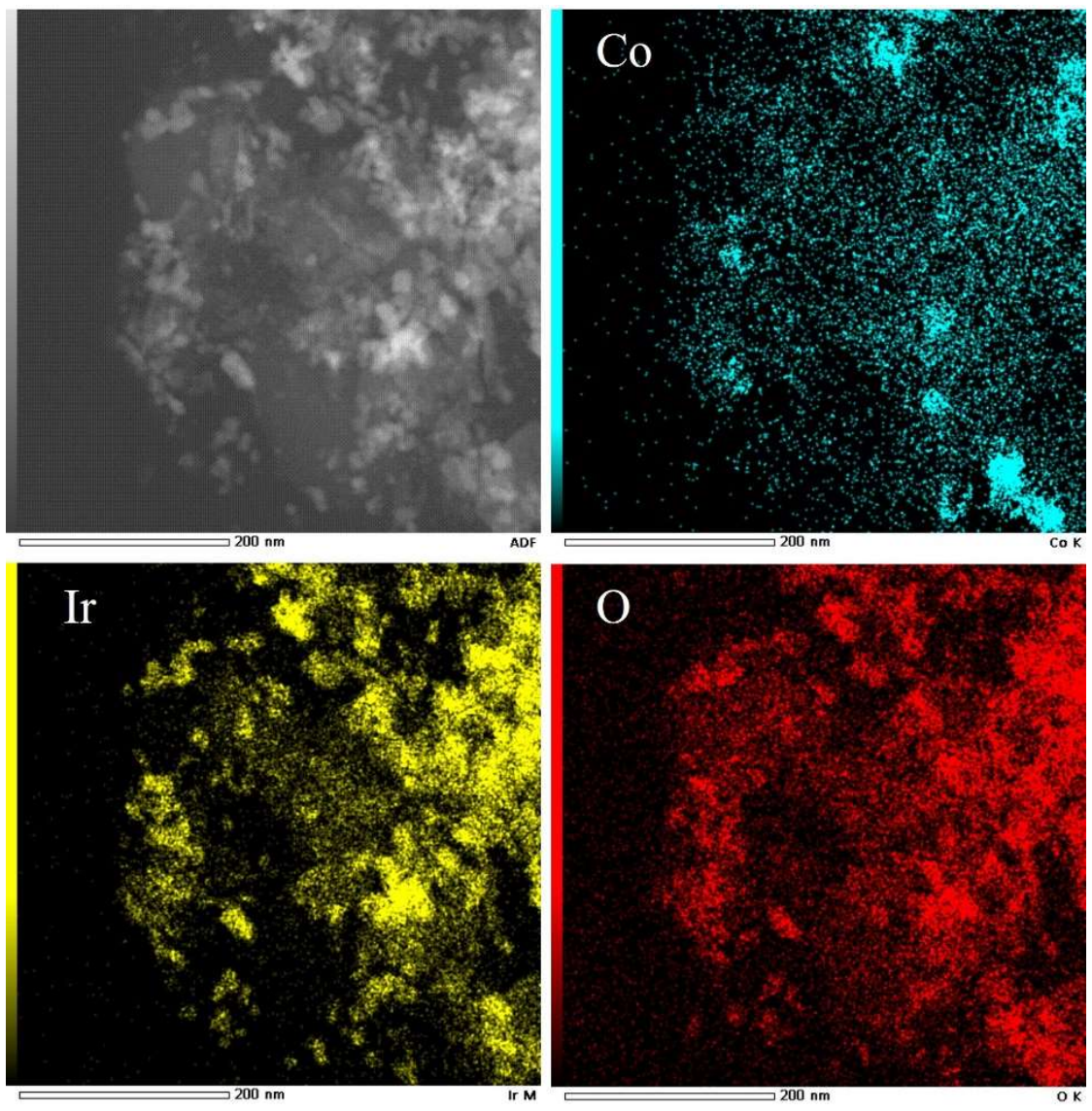

Fig. S41. STEM-EDS elemental mapping of $IrCoO_x$-2-after.

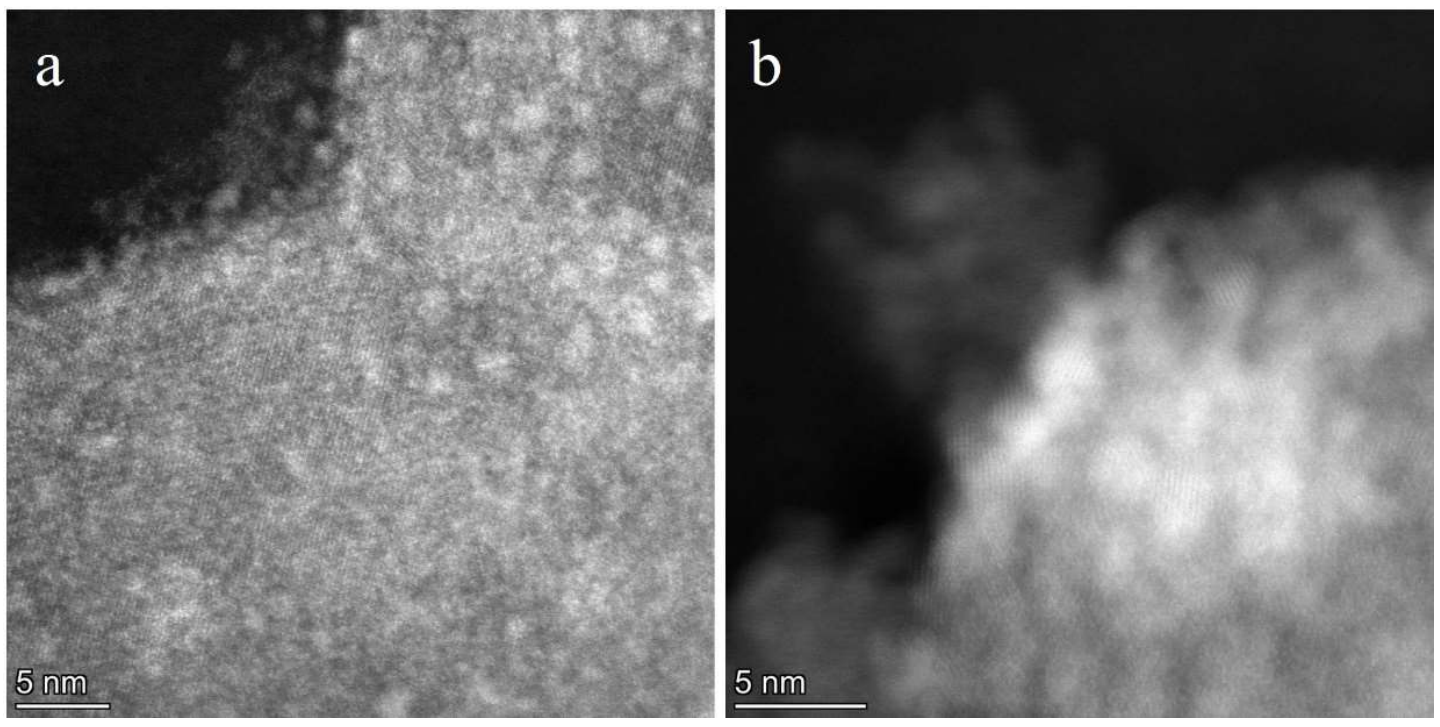

Fig. S42. The STEM-HAADF images of $IrCoO_x$-2-after under the spherical aberration electron microscope.

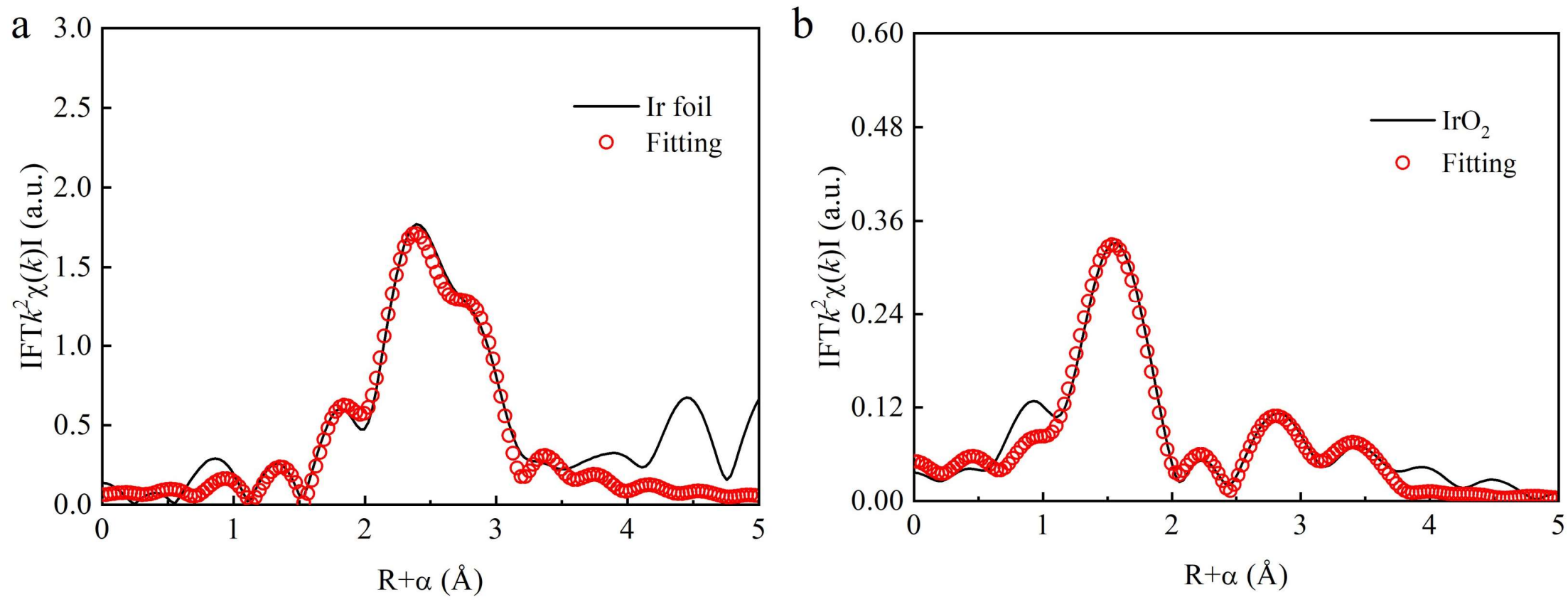

Fig. S43. EXAFS fitting results of (a) Ir foil and (b) commercial IrO_2 samples.

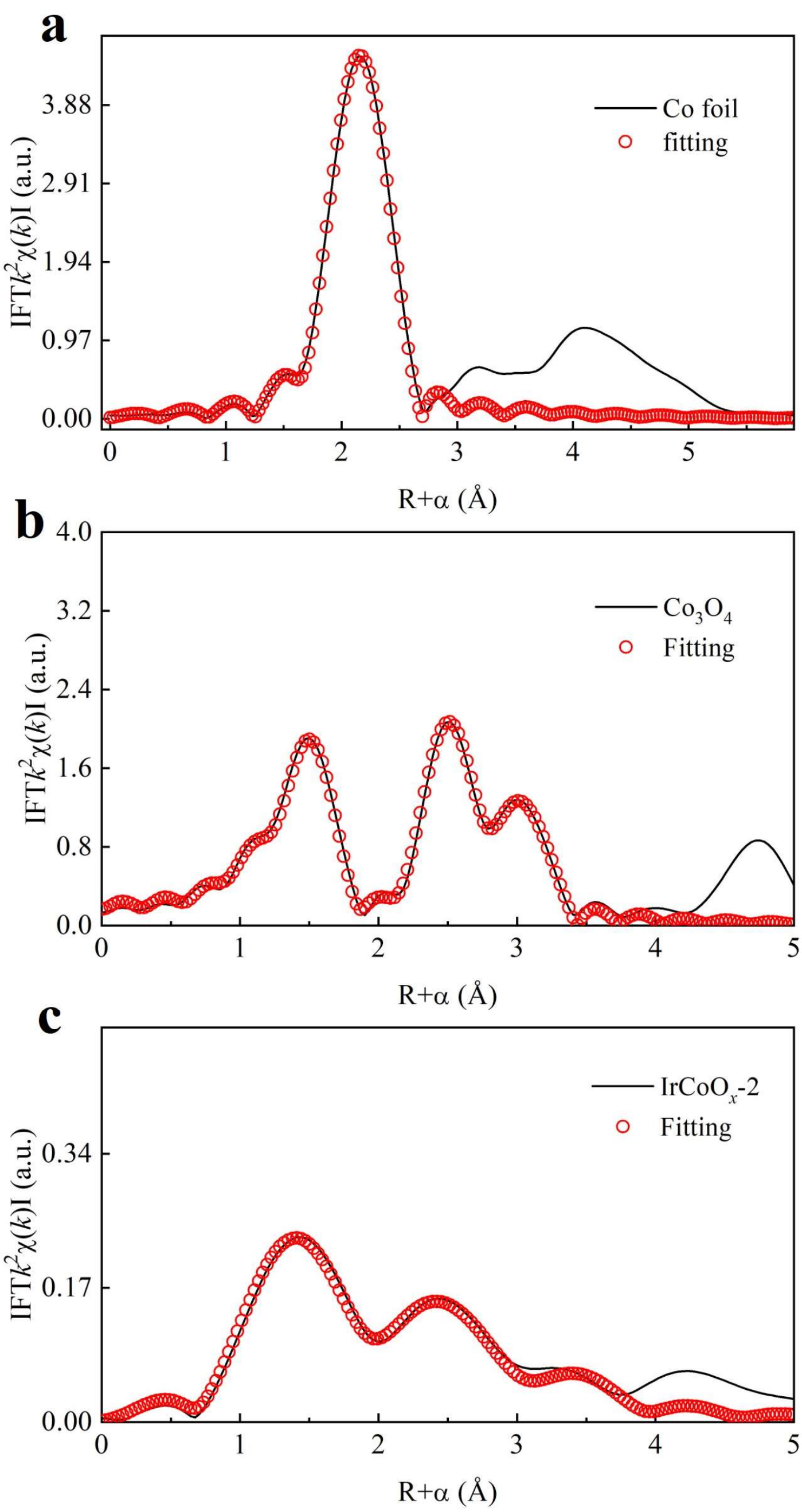

Fig. S44. EXAFS fitting results of (a) Co foil, (b) commercial Co_3O_4 and (c) $IrCoO_x$-2 samples.

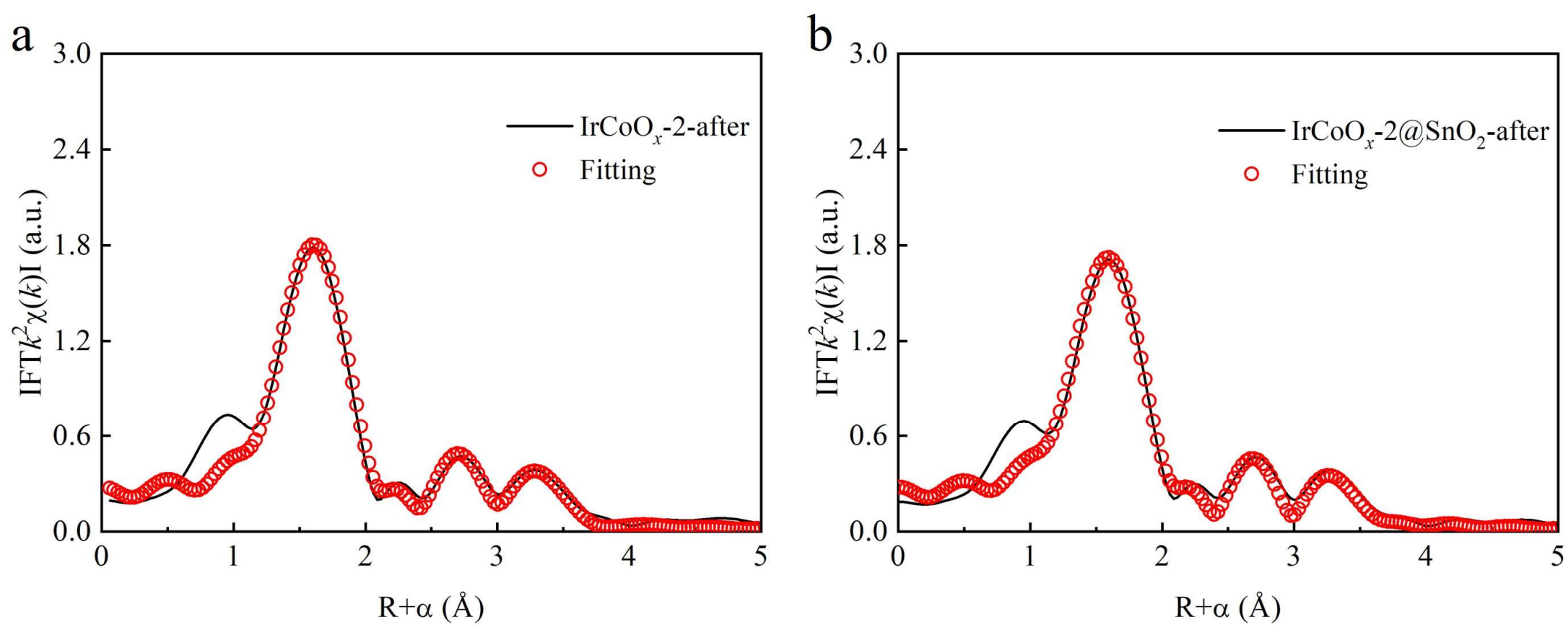

Fig. S45. EXAFS fitting results of (a) $IrCoO_x$-2-after and (b) $IrCoO_x$-2@SnO_2-after samples.

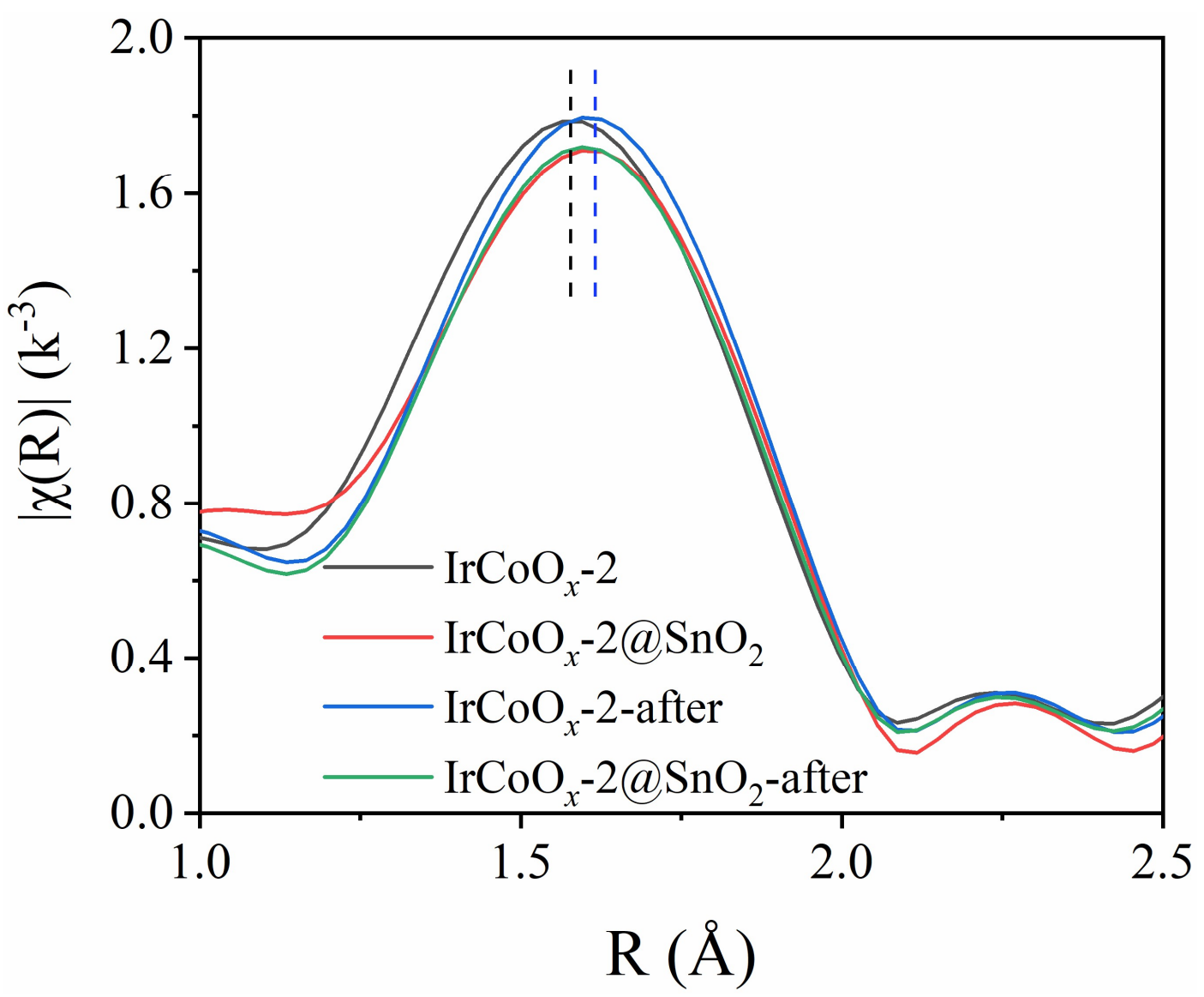

Fig. S46. Comparison of Ir L_3-edge EXAFS spectra for $IrCoO_x$-2@SnO_2 and $IrCoO_x$-2 before and after the OER stability test.

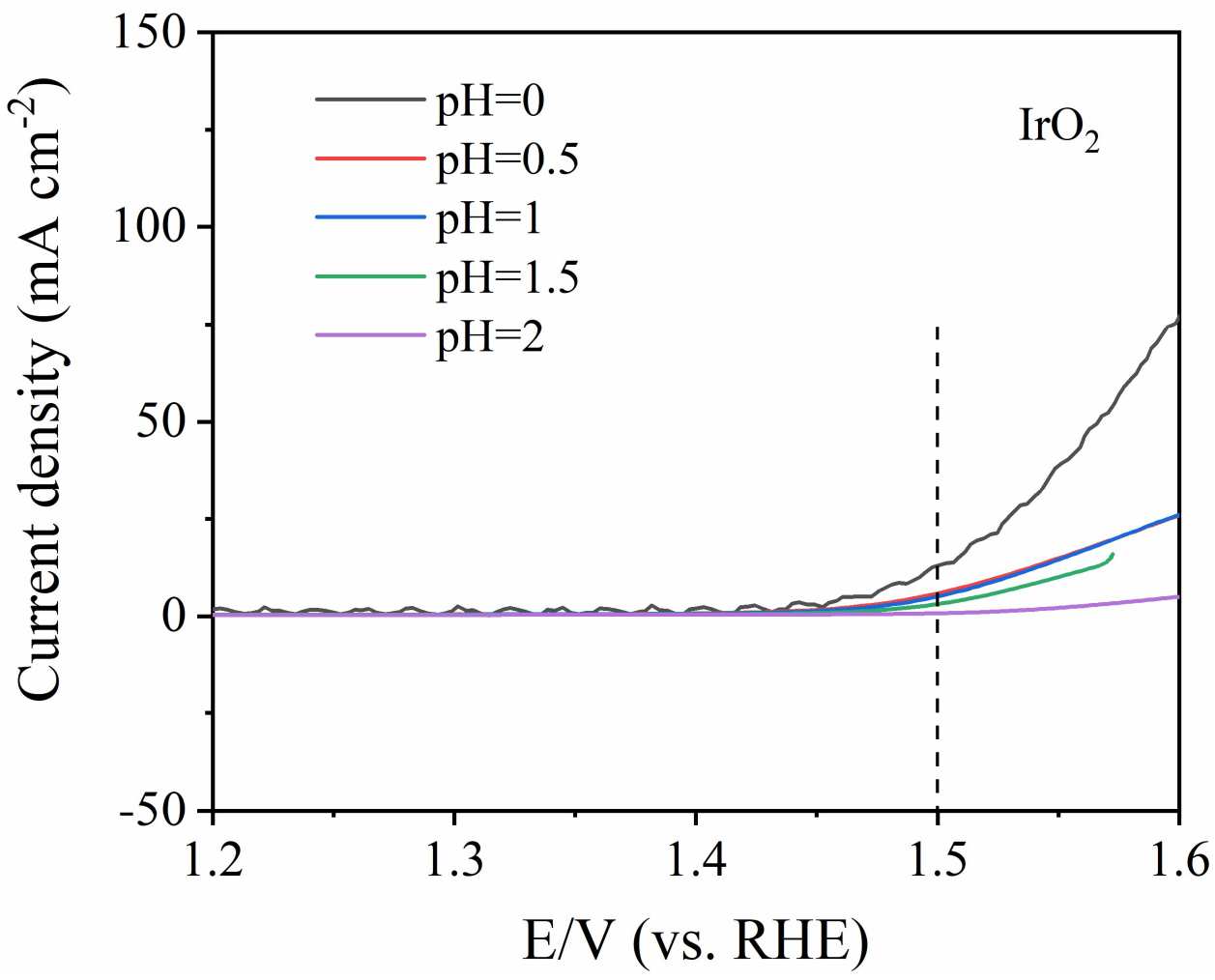

Fig. S47. LSV curves under different pH values for commercial IrO_2.

As shown in Figs. S47-S49, the LSV curves under different pH values for IrO_2, $IrCoO_x$-2 and $IrCoO_x$-2@SnO_2 were conducted.

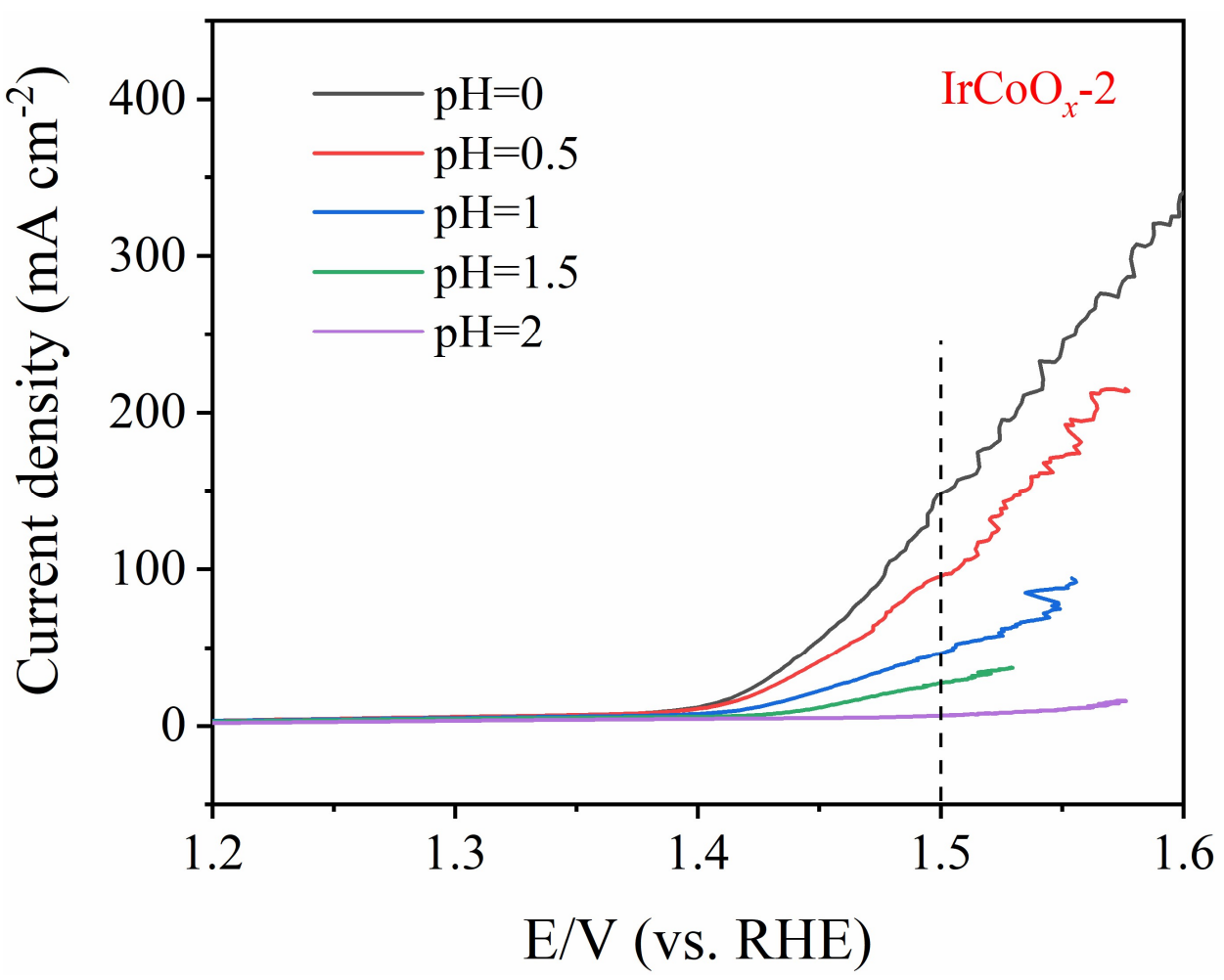

Fig. S48. LSV curves under different pH values for IrCoO$_x$-2.

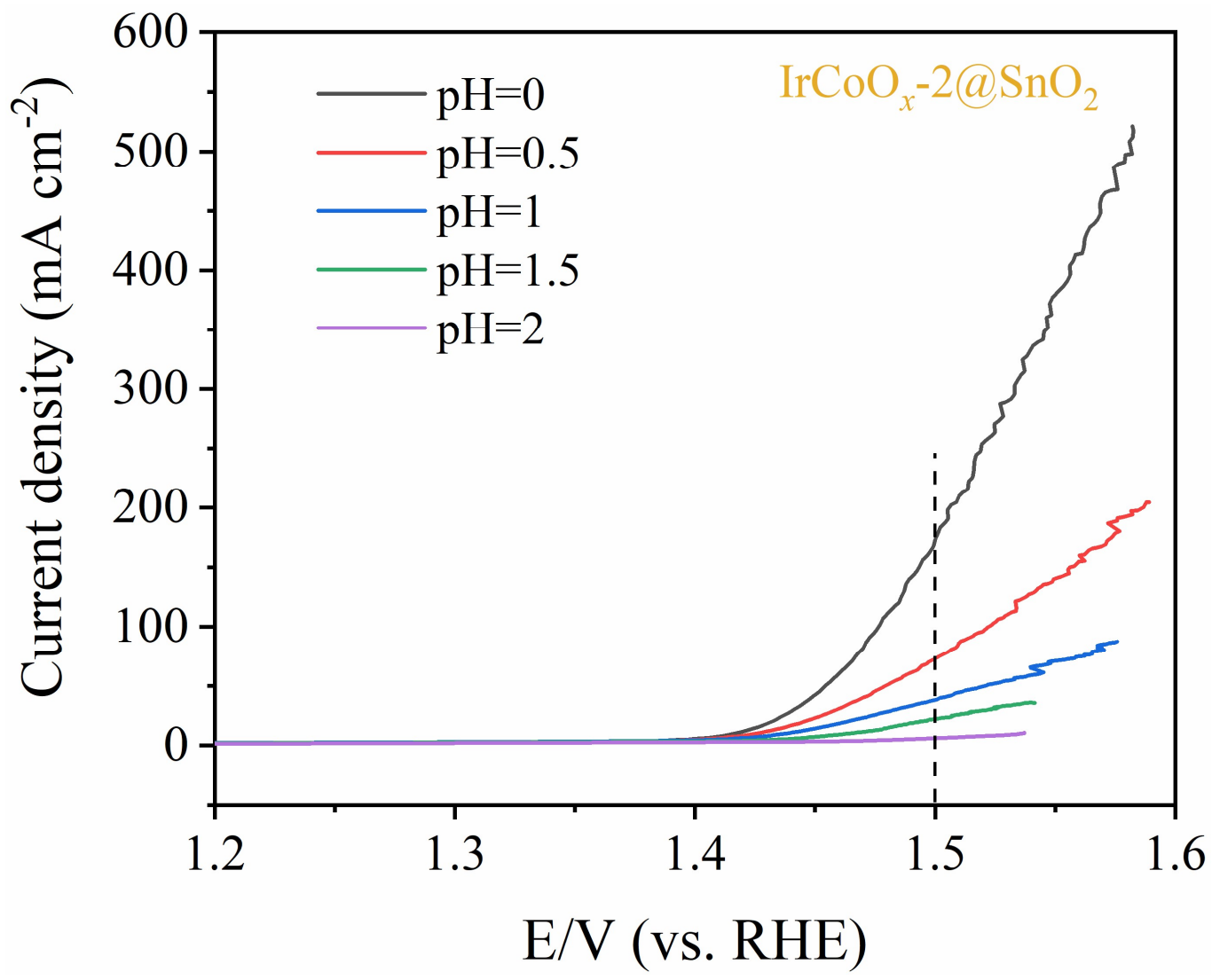

Fig. S49. LSV curves under different pH values for $IrCoO_x$-2@SnO_2.

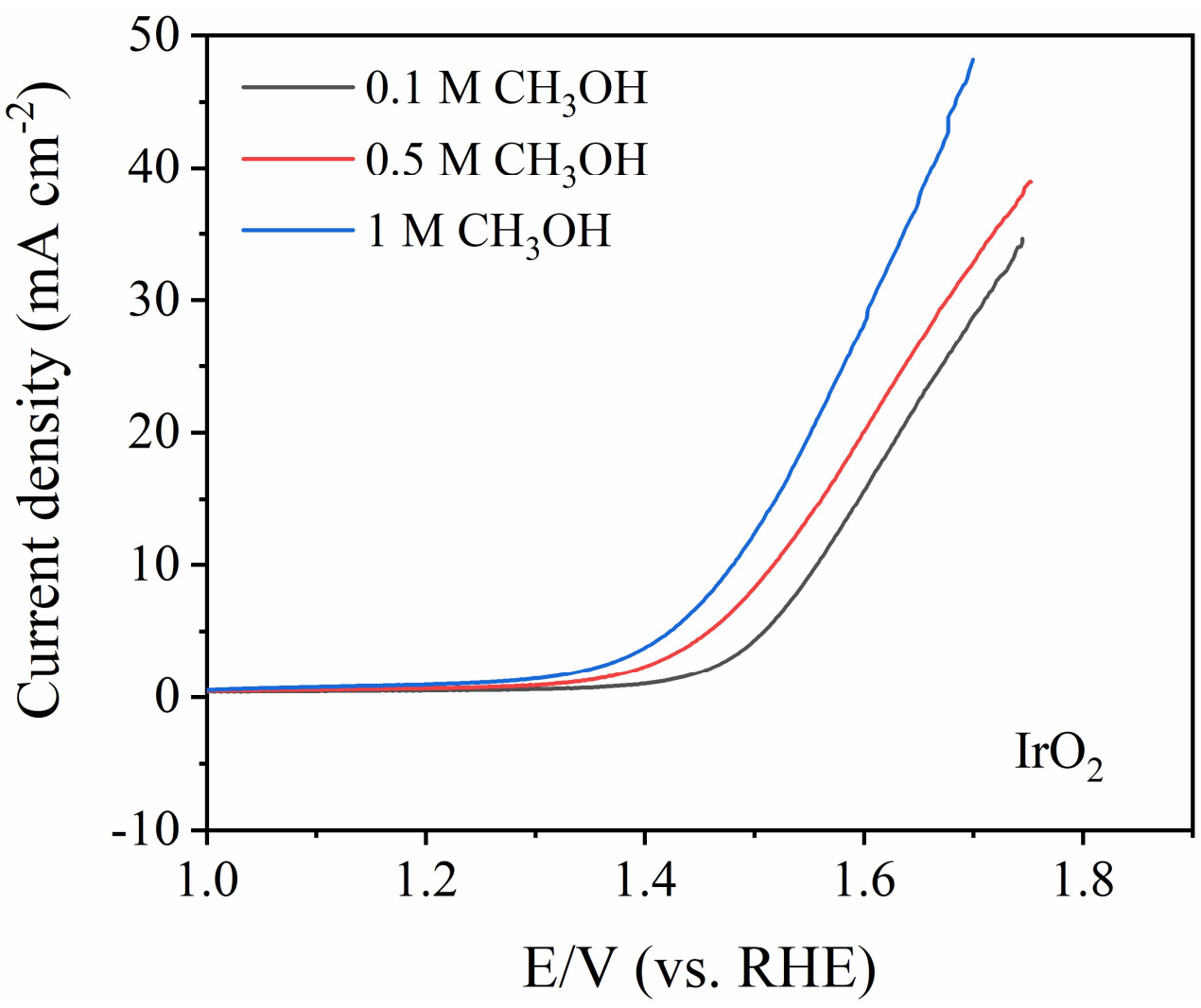

Fig. S50. LSV curves under different methanol concentrations for commercial IrO_2.

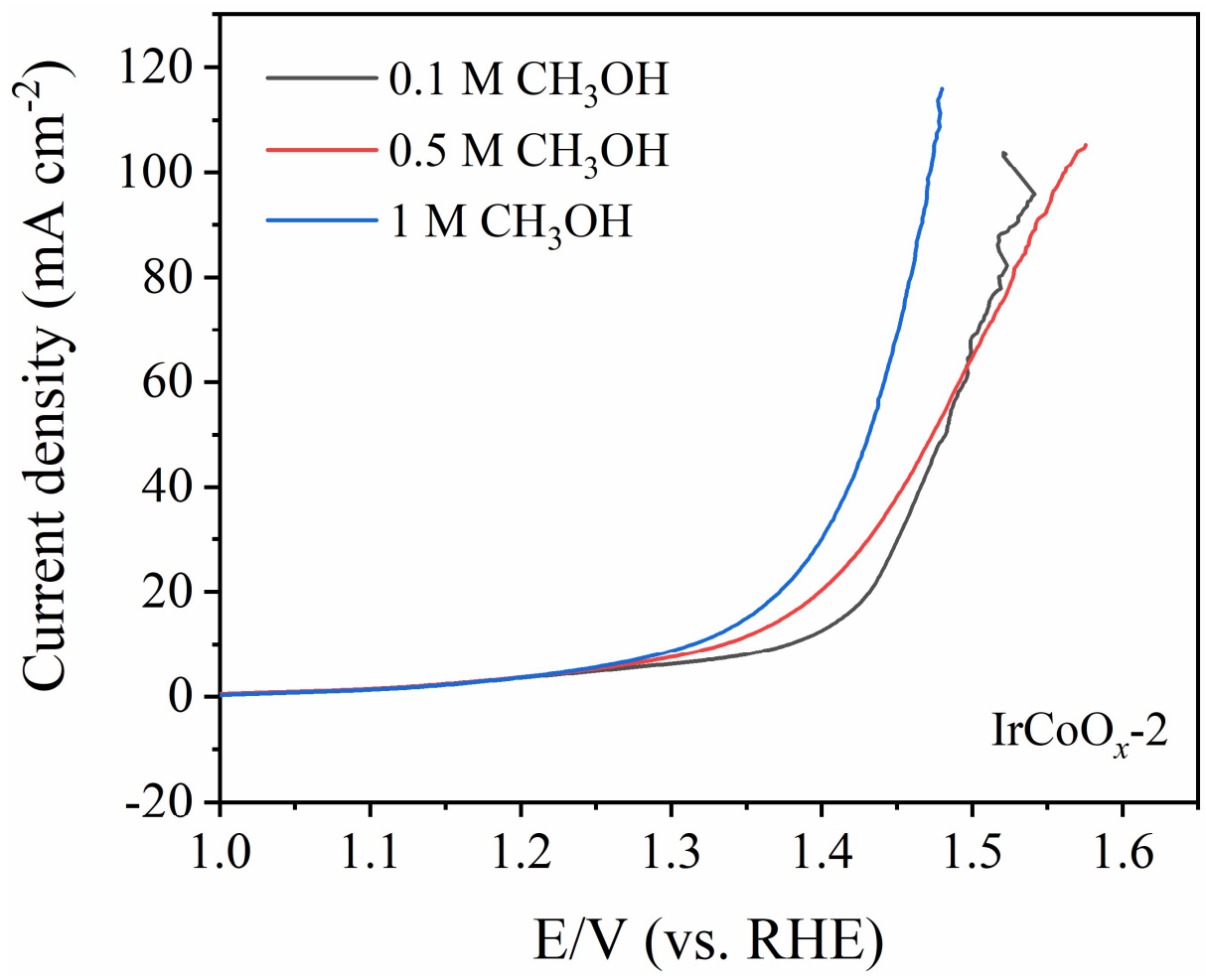

Fig. S51. LSV curves under different methanol concentrations for $IrCoO_x$-2.

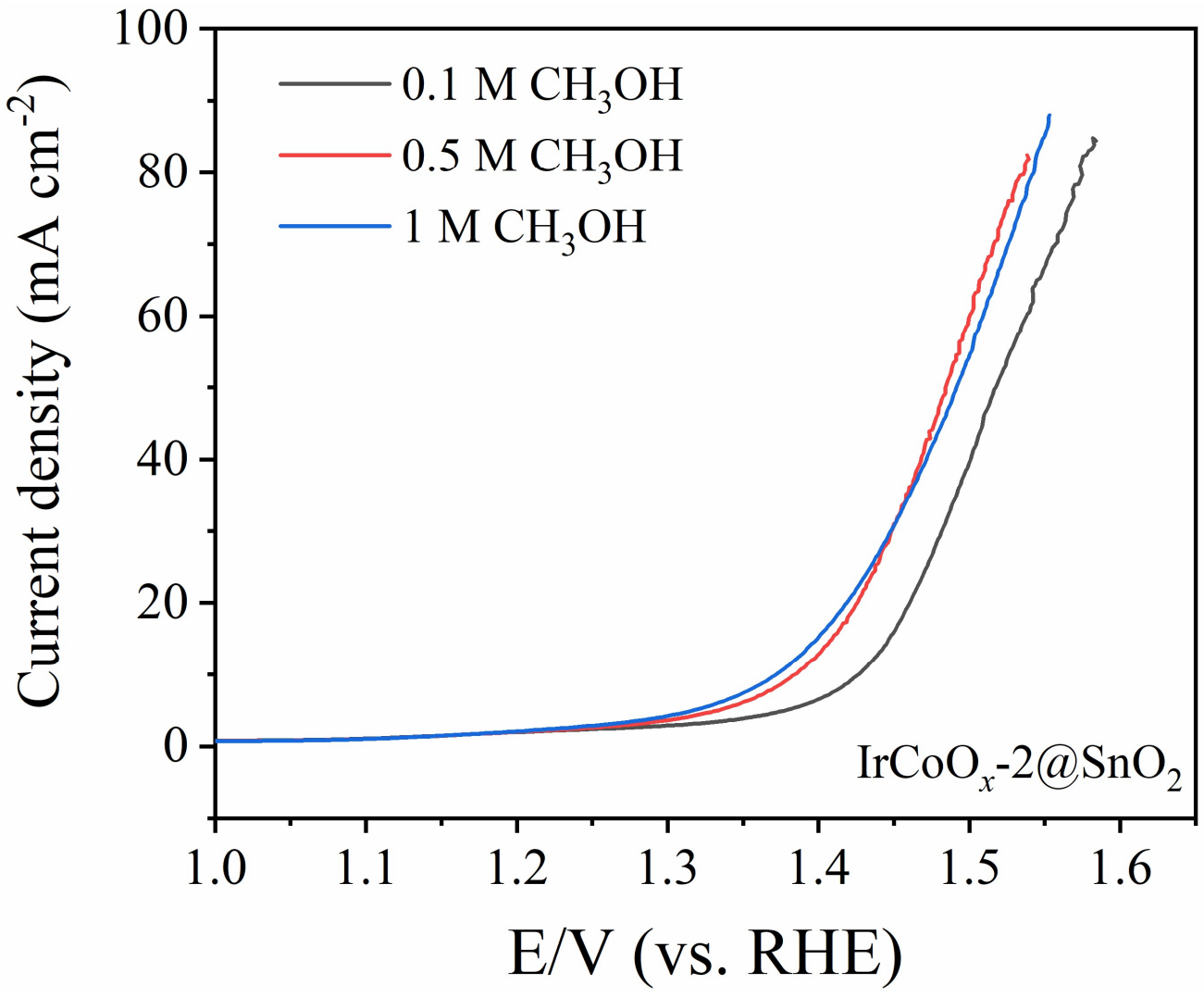

Fig. S52. LSV curves under different methanol concentrations for $IrCoO_x$-2@SnO_2.

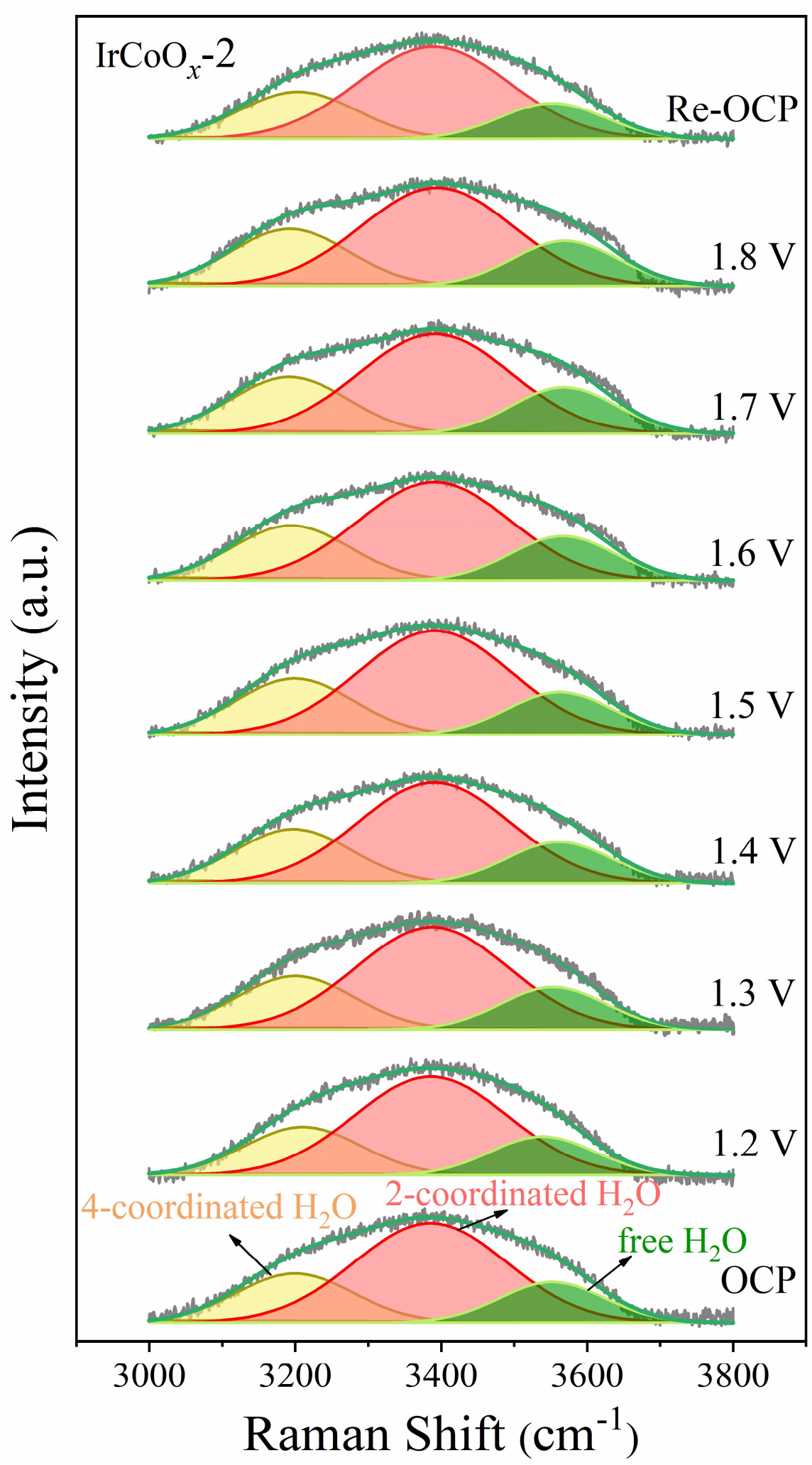

Fig. S53. The *in-situ* Raman spectra were recorded at potentials from OCV to 1.8 V (an interval of 0.1 V) on IrCoO$_x$-2.

The broad O-H stretching region in the Raman spectra could be deconvoluted into three components at approximately 3200, 3400, and 3600 cm^{-1}, corresponding to strongly hydrogen-bonded (4-coordinated), moderately hydrogen-bonded (2-coordinated) and free water molecules, respectively (see Tables S10 and S11 for fitting details). Typically, free water molecules as a reactant can promptly replenish lattice oxygen, avoiding the possible loss of lattice oxygen for LOM. While free water molecules exhibit great rotational freedom, their poorly connected networks are inefficient for proton transfer. Correspondingly, 4-coordinated H_2O molecules establish

a robust hydrogen-bonded network, facilitating rapid proton transfer, which can mitigate over-oxidation and dissolution of Ir active sites by counteracting electron depletion induced by proton accumulation. However, too strong hydrogen-bonded water network, despite its good connectivity, also impedes the movement of water molecules as it requires high energy for molecular reorientation, potentially affecting the dissociation and coupling processes.

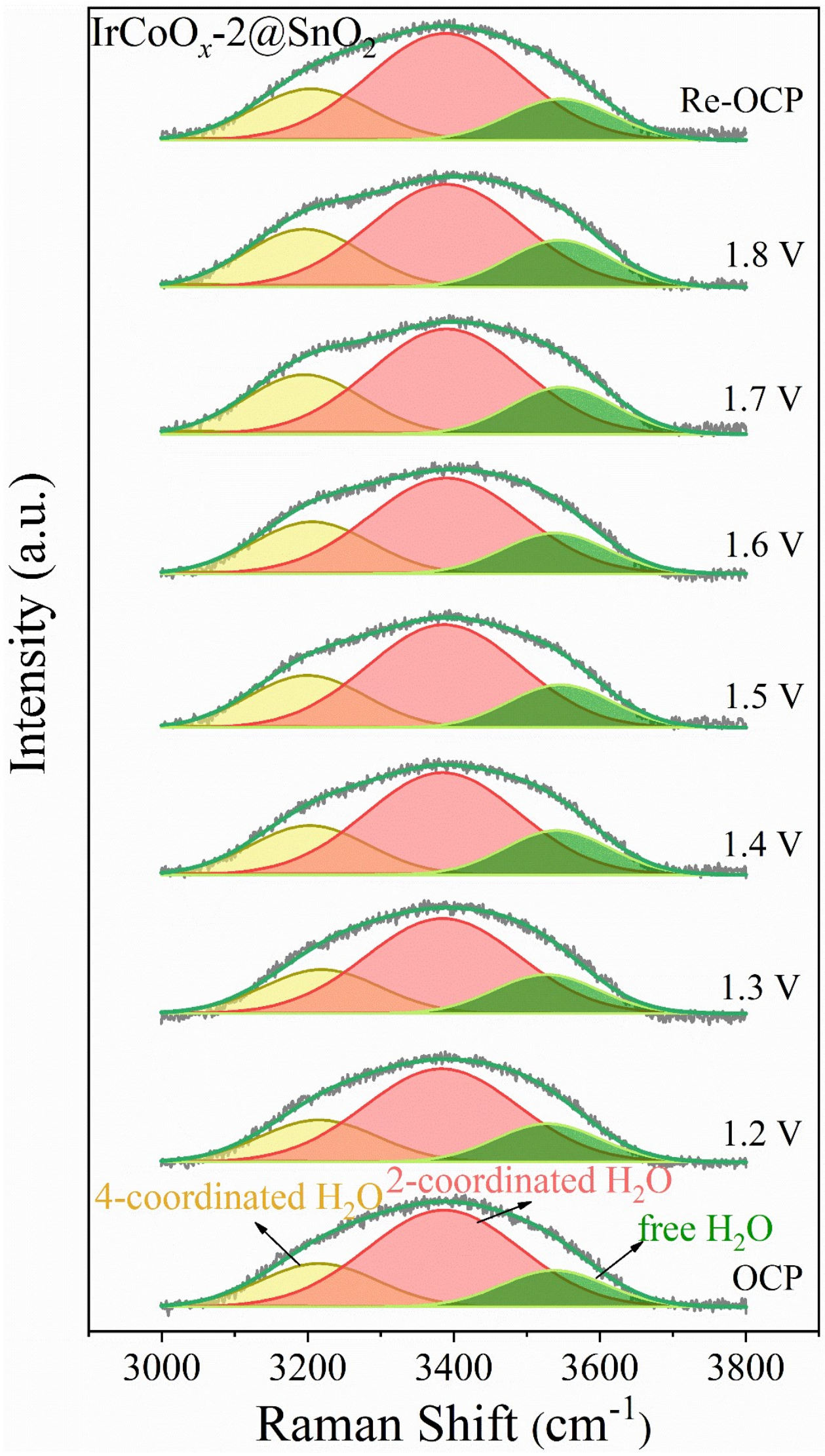

Fig. S54. The *in-situ* Raman spectra were recorded at potentials from OCV to 1.8 V (an interval of 0.1 V) on IrCoO$_x$-2@SnO$_2$.

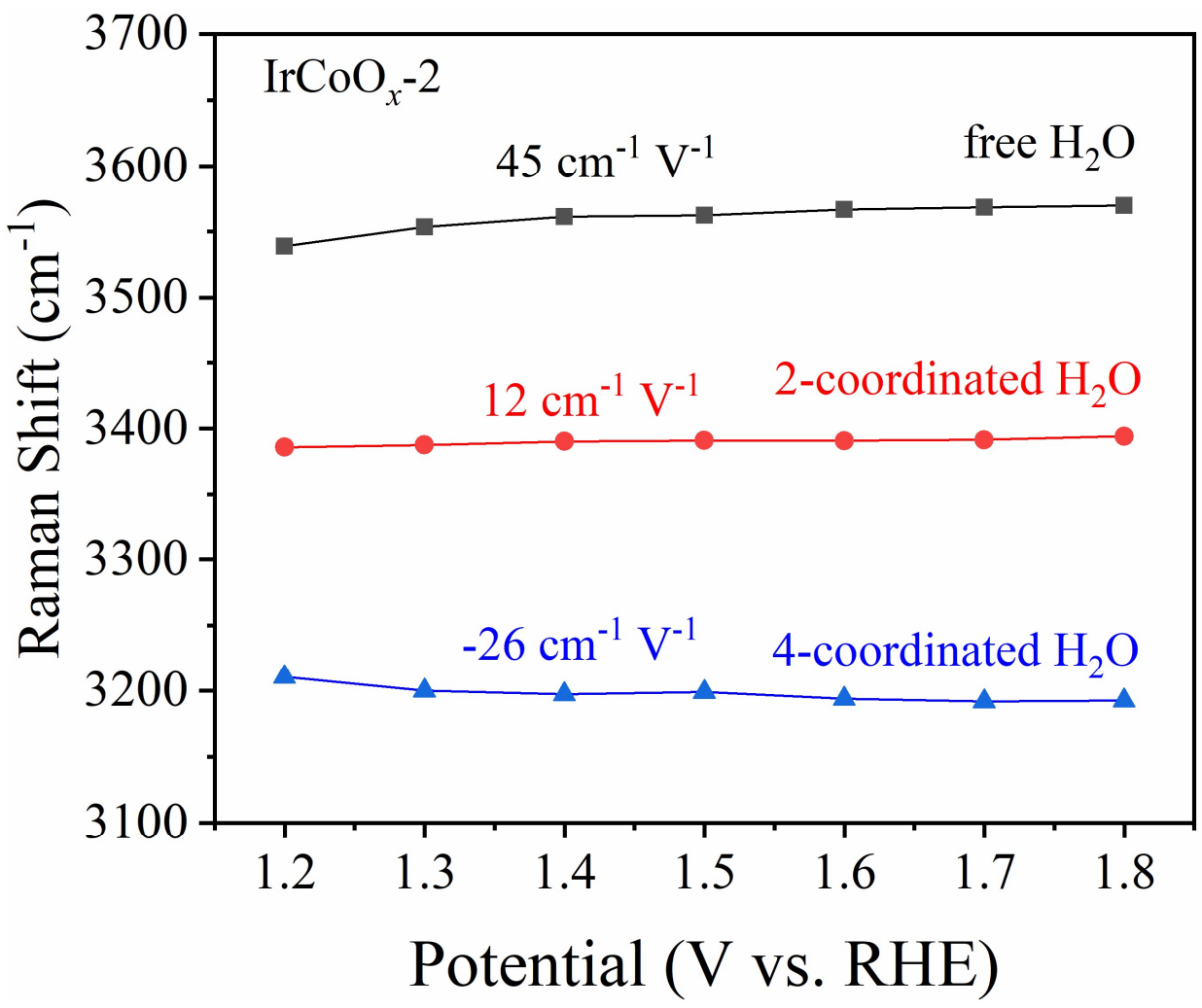

Fig. S55. Potential-dependent Raman peak shifts of various interfacial water types in IrCoO$_x$-2.

For all three water species, the vibrational frequencies vary with electrode potential and are attributed to the vibrational Stark effect.

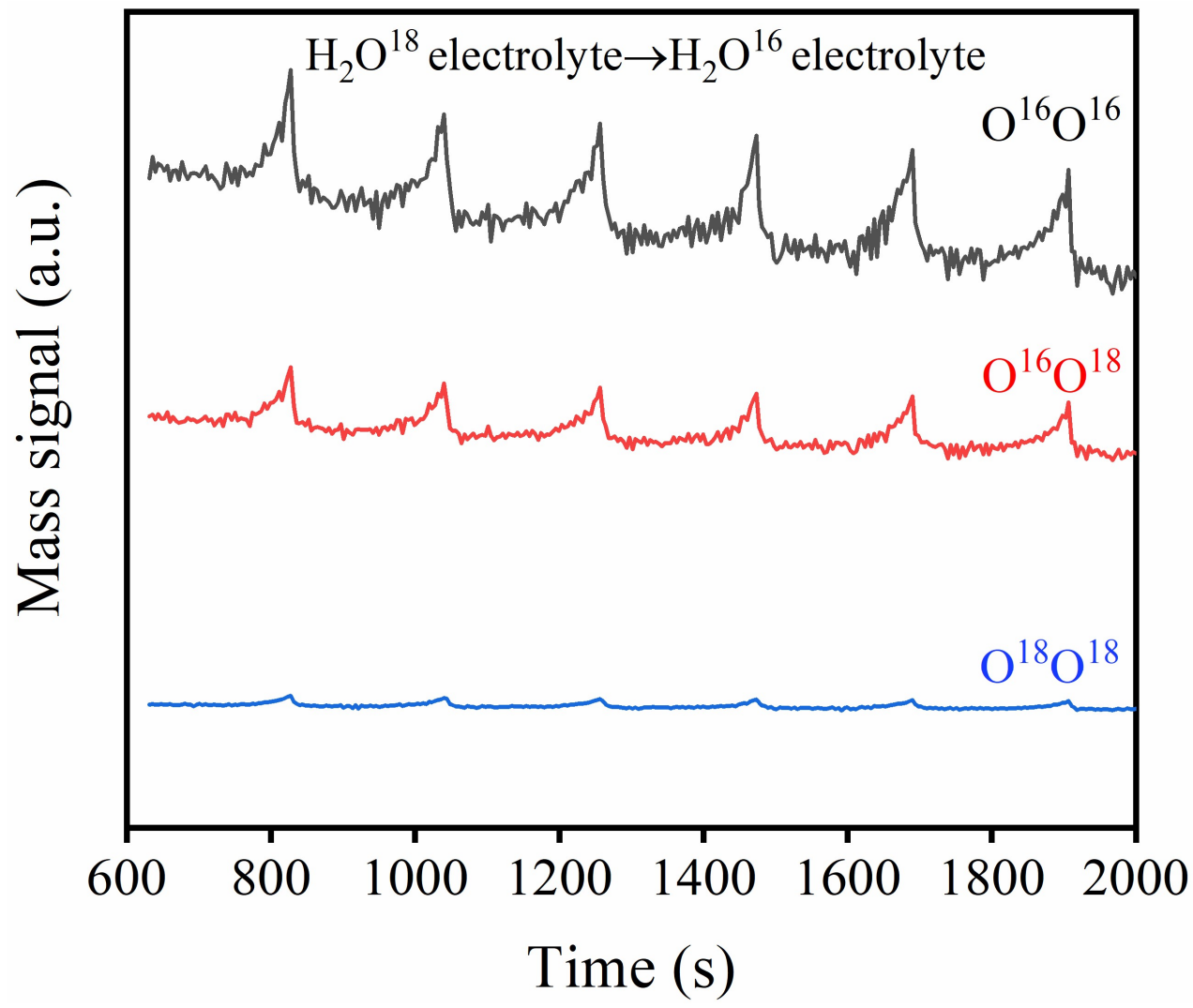

Fig. S56. DEMS signals of different O_2 products for O^{18} labeled $IrCoO_x$-2@SnO_2 in 0.5 M H_2SO_4 using H_2O^{16} as electrolyte.

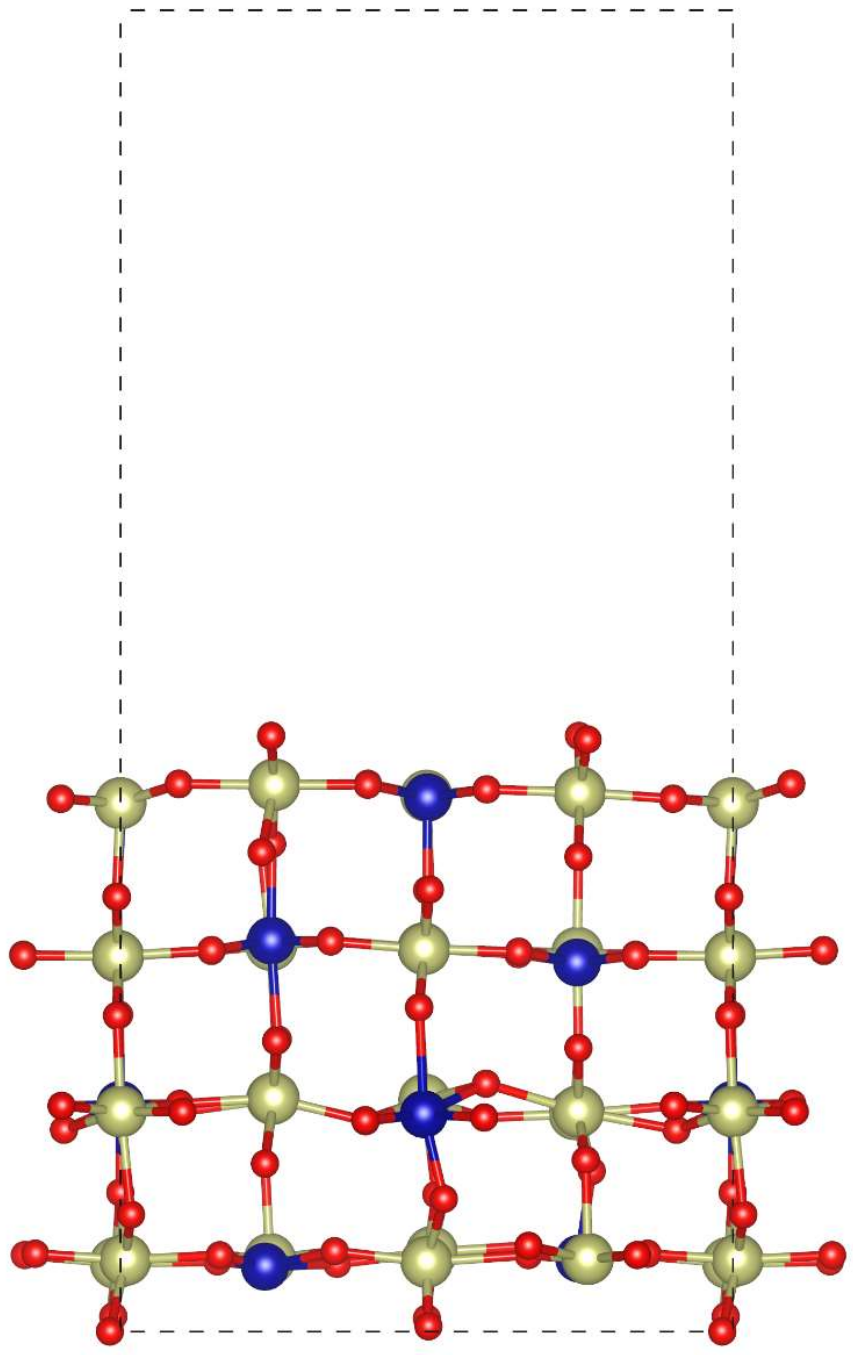

Fig. S57. The structure model of $IrCoO_x$.

A stable paracrystalline $IrCoO_x$ structure (Fig. S57) was obtained *via* AIMD by gradually heating the system from 100 K to 2000 K over 20 ps using velocity scaling. The resulting configuration was then fully relaxed to its ground state for subsequent analysis.

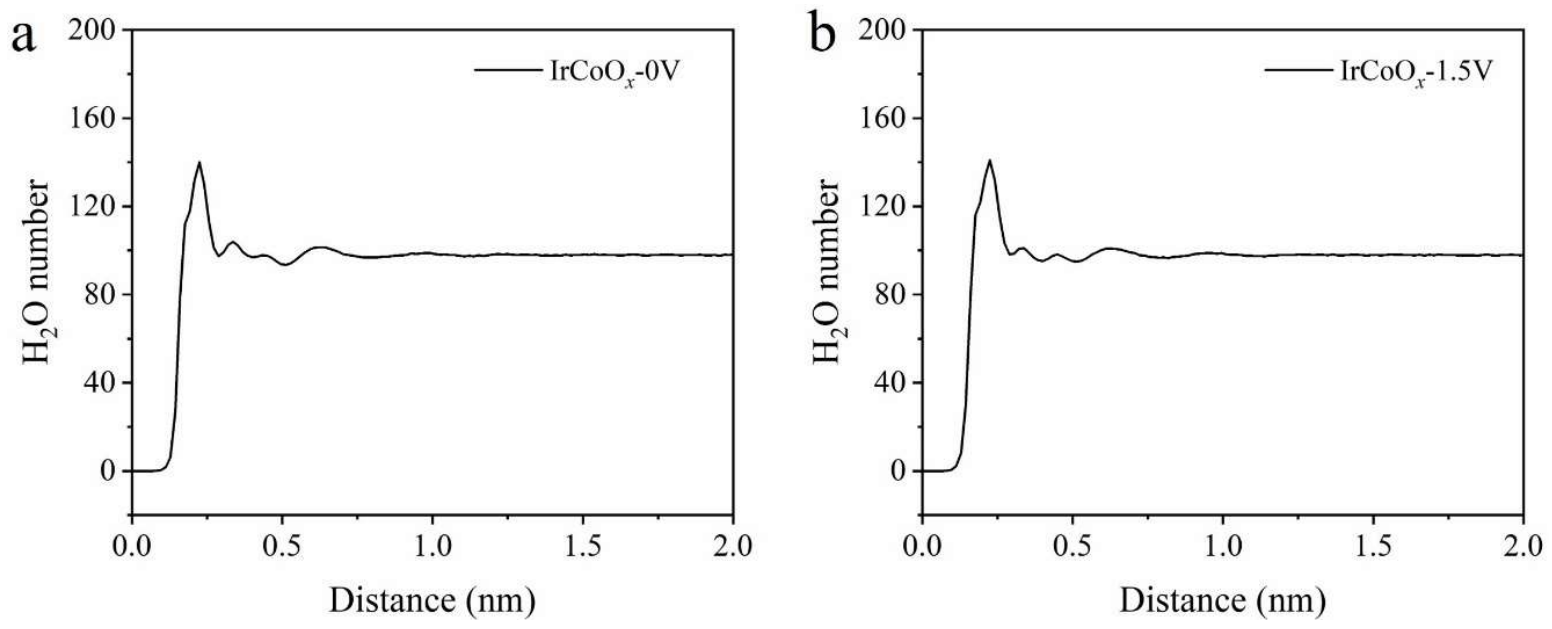

Fig. S58. The interfacial H_2O number for $IrCoO_x$ at 0 V and 1.5 V.

The number of interfacial H_2O molecules for $IrCoO_x@SnO_2$ is also higher than that of $IrCoO_x$ (Figs. S58-S59).

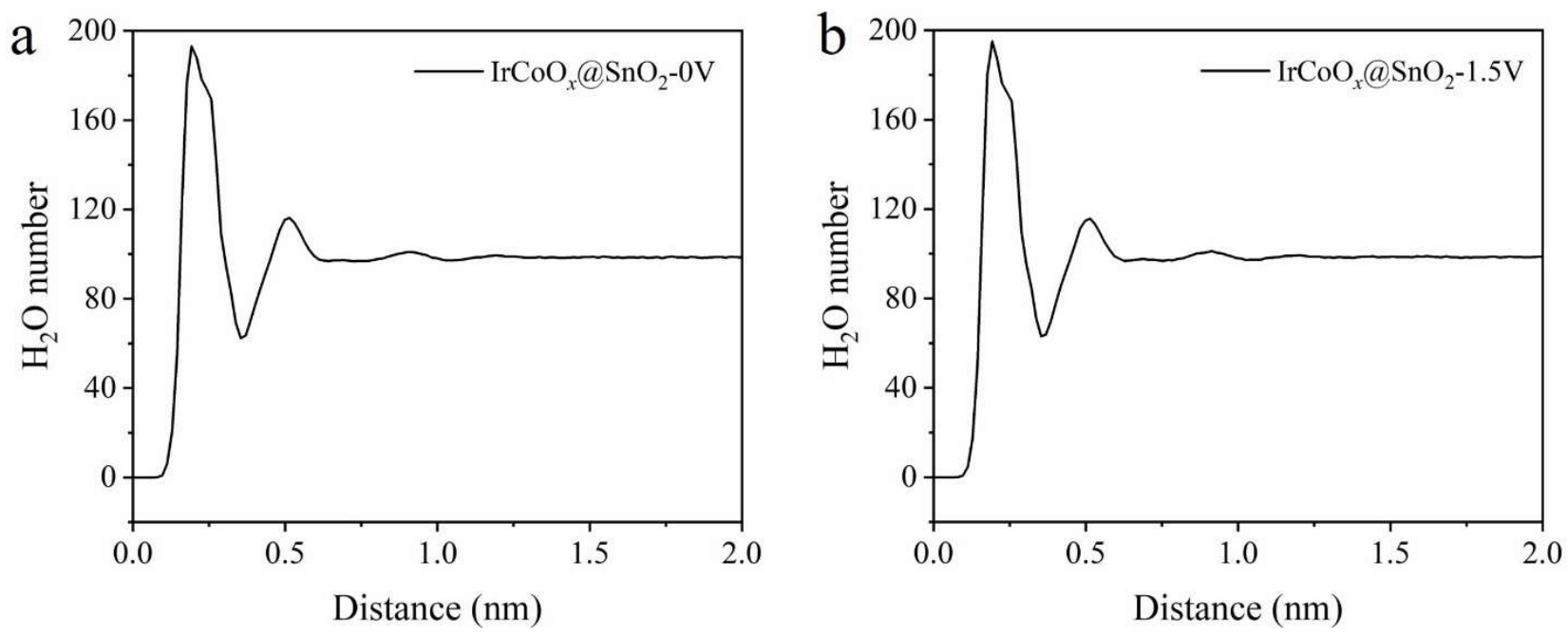

Fig. S59. The interfacial H_2O number for IrCoO$_x$@SnO$_2$ at 0 V and 1.5 V.

Table S1. The specific values of four sub-bands in XPS Ir 4*f* spectra for $IrCoO_x$-1, $IrCoO_x$-2 and $IrCoO_x$-3.

Sample	Sub-bands	Area	Lorentzian-Gaussian	Percentage/%
$IrCoO_x$-1	Ir^0 $4f_{7/2}$	24647	20	**0.544**
	Ir^0 $4f_{5/2}$	18485.25	20	**0.544**
	Ir^{4+} $4f_{7/2}$	20664.87	20	**0.456**
	Ir^{4+} $4f_{5/2}$	15498.65	20	**0.456**
$IrCoO_x$-2	Ir^0 $4f_{7/2}$	23305.83	20	**0.576**
	Ir^0 $4f_{5/2}$	17479.37	20	**0.576**
	Ir^{4+} $4f_{7/2}$	17132.71	20	**0.424**
	Ir^{4+} $4f_{5/2}$	12849.54	20	**0.424**
$IrCoO_x$-3	Ir^0 $4f_{7/2}$	19275.59	20	**0.587**
	Ir^0 $4f_{5/2}$	14456.69	20	**0.587**
	Ir^{4+} $4f_{7/2}$	13587.55	20	**0.413**
	Ir^{4+} $4f_{5/2}$	10190.66	20	**0.413**

Table S2. The specific values of four sub-bands in XPS O 1*s* spectra for $IrCoO_x$-1, $IrCoO_x$-2 and $IrCoO_x$-3.

Sample	Sub-bands	Area	Lorentzian-Gaussian	Percentage/%
$IrCoO_x$-1	O_M	14947.64	20	**0.193**
	O_L	34857.7	20	**0.449**
	O_V	20690.82	20	**0.266**
	O_{OH}	7121.225	20	**0.092**
$IrCoO_x$-2	O_M	17151.34	20	**0.234**
	O_L	29121.37	20	**0.398**
	O_V	19875.9	20	**0.272**
	O_{OH}	7049.853	20	**0.096**
$IrCoO_x$-3	O_M	18936.39	20	**0.283**
	O_L	26368.16	20	**0.394**
	O_V	15969.66	20	**0.239**
	O_{OH}	5603.366	20	**0.084**

Table S3. The specific values of lattice spacing for $IrCoO_x$ formation from RT to 360 °C.

Temperature/°C	Lattice spacing/nm	Lattice plane
RT	0.154	CoO-225 (022)
	0.218	CoO-225 (002)
	0.252	CoO-225 (111)
100	0.152	CoO-225 (022)
	0.251	CoO-225 (111)
200	0.25	CoO-225 (111)

Table S4. The comparison of OER performances for Ir/Ru-based electrocatalysts using glass carbon electrode (GCE) in acidic electrolyte. We here only list the data of relatively high catalytic activities of the catalysts reported in high-impact literature.

Electrocatalyst	Electrolyte	Overpotential (at 10 mA cm^{-2})/mV	Reference
$IrCoO_x$-2@SnO_2	0.5 M H_2SO_4	170	This work
$Ta_{0.1}Ru_{0.9}O_{2-x}$	0.5 M H_2SO_4	226	[1]
IrO_x/t-ZrO_2	0.1 M $HClO_4$	287.5	[2]
Ir_{SAE}-CMO	0.5 M H_2SO_4	235	[3]
ZrO_2-RuO_2	0.5 M H_2SO_4	170	[4]
O-IrVMn/IrO_x	0.1 M $HClO_4$	279	[5]
IrO_x/TiN	0.1 M $HClO_4$	293	[6]
$Mn_{0.2}RuO_2$	0.5 M H_2SO_4	188	[7]
Er-RuO_x	0.5 M H_2SO_4	200	[8]
Ni-RuO_2	0.1 M $HClO_4$	214	[9]
$IrO_x\cdot nH_2O$	0.1 M $HClO_4$	283	[10]
Ir-Sn PSC	0.5 M H_2SO_4	193	[11]
$RuCoO_x$	1 M $HClO_4$	200	[12]
IrO_x	0.5 M H_2SO_4	231	[13]
Ir/Nb_2O_{5-x}	0.5 M H_2SO_4	218	[14]
Ir-IrO_x/C	0.5 M H_2SO_4	198	[15]
GB-$Tm_{0.1}Tm_{0.1}Ir_{0.8}O_{2-\delta}$	0.5 M H_2SO_4	198	[16]
1T-IrO_2	0.5 M H_2SO_4	197	[17]

Table S5. The comparison of PEMWE performance using $IrCoO_x$-2@SnO_2 as the anode catalyst with the reported Ru/Ir-based catalysts in high-impact papers.

Catalyst	Current Density (A cm^{-2})	Voltage (V)	Temperature (°C)	Loading amount	Time (h)	Reference
$IrCoO_x$@SnO_2	2	1.63	80	1.5 mg cm^{-2}	3880	This work
RIE-Ir/CeO_x	1	1.5	80	0.3 mg_{Ir} cm^{-2}	2100	[18]
	2	1.631	80	0.3 mg_{Ir} cm^{-2}	1100	
Ir^{VI}-ado	1.8	1.8	80	0.08 mg_{Ir} cm^{-2}	2700	[19]
$Ta_{0.1}Ru_{0.9}O_{2-x}$	1	1.704	50	0.8 mg cm^{-2}	2800	[1]
IrO_x	1	1.72	80	2.5 mg cm^{-2}	1200	[13]
$IrO_x\cdot nH_2O$	1	1.77	60	2 mg cm^{-2}	600	[10]
TaO_x-IrO_2	1	1.67	80	0.15 mg_{Ir} cm^{-2}	100	[20]
ZrO_2-RuO_2	2	1.78 V	80	2.5 mg cm^{-2}	100	[4]
IrO_x/t-ZrO_2	1	1.72	80	0.1 mg_{Ir} cm^{-2}	1600	[2]
Ir/Nb_2O_{5-x}	2	1.7	80	3 mg cm^{-2}	2000	[14]
dh-IrO_x-230	2	1.75	80	0.5 mg_{Ir} cm^{-2}	1000	[21]
	1	1.6	80	0.5 mg_{Ir} cm^{-2}	2000	
Ir-$SrMnO_3$	0.5	1.53	80	0.7 mg_{Ir} cm^{-2}	2000	[22]
TS-Ir/MnO_2	0.5	1.75	80	0.039 mg_{Ir} cm^{-2}	200	[23]

Table S6. The specific values of four sub-bands in XPS O 1*s* spectra for $IrCoO_x$-2, $IrCoO_x$-2-after, $IrCoO_x$-2@SnO_2 and $IrCoO_x$-2@SnO_2-after.

Sample	Sub-bands	Area	Lorentzian-Gaussian	Percentage/%
$IrCoO_x$-2	O_M	17151.34	20	**0.234**
	O_L	29121.37	20	**0.398**
	O_V	19875.9	20	**0.272**
	O_{OH}	7049.853	20	**0.096**
$IrCoO_x$-2-after	O_M	-	20	**-**
	O_L	2273.257	20	**0.184**
	O_V	3199.228	20	**0.259**
	O_{OH}	6884.099	20	**0.557**
$IrCoO_x$-2@SnO_2	O_M	11719.5	20	**0.150**
	O_L	30043.37	20	**0.385**
	O_V	23137.16	20	**0.297**
	O_{OH}	13138.66	20	**0.168**
$IrCoO_x$-2@SnO_2-after	O_M	1001.93	20	**0.111**
	O_L	1931.235	20	**0.215**
	O_V	2567.489	20	**0.285**
	O_{OH}	3507.897	20	**0.389**

Table S7. The ICP results of electrolyte after the stability test for commercial IrO_2, $IrCoO_x$-2 and $IrCoO_x$-2@SnO_2.

Element (ug/mL)	Post-rection electrolyte		
	IrO_2	$IrCoO_x$-2	$IrCoO_x$-2@SnO_2
Ir	<0.05	0.56	0.38
Co	-	0.34	0.30
Sn	-	-	<0.05

Table S8. EXAFS fitting parameters at the Ir L^3-edge for various samples.

Sample	Shell	CN[a]	R(Å)[b]	σ^2(Å^2)[c]	ΔE_0(eV)[d]	R factor
Ir foil	Ir-Ir	12*	2.70±0.01	0.0030	6.9	0.0073
IrO_2	Ir-O	6.0*	1.97±0.01	0.0028	9.3	0.0145
	Ir-Ir	6.0*	3.19±0.01	0.0042	9.3	
	Ir-Ir	6.0*	3.57±0.01	0.0192	4.0	
$IrCoO_x$-2	Ir-O	5.9±0.3	1.99±0.01	0.0060	13.0	0.0158
	Ir-Co	4.8±1.0	3.07±0.01	0.0187	8.4	
	Ir-Co	5.8±1.2	3.70±0.01	0.0140	-2.0	
$IrCoO_x$-2@SnO_2	Ir-O	5.7±0.3	2.00±0.01	0.0059	12.4	0.0173
	Ir-Co	3.9±0.8	3.10±0.01	0.0160	8.8	
	Ir-Co	5.8±1.2	3.70±0.01	0.0152	-2.5	
$IrCoO_x$-2-after	Ir-O	5.5±0.3	2.02±0.01	0.0048	13.2	0.0137
	Ir-Co	4.2±0.8	3.08±0.01	0.0169	7.1	
	Ir-Co	7.1±1.4	3.72±0.01	0.0166	-3.4	
$IrCoO_x$-2@SnO_2-after	Ir-O	5.5±0.3	2.02±0.01	0.0054	12.1	0.0156
	Ir-Co	2.5±0.5	3.08±0.01	0.0123	5.1	
	Ir-Co	3.4±0.7	3.72±0.01	0.0100	-3.2	

Note: (a) CN, coordination number; (b) R, distance between absorber and backscatter atoms; (c) σ^2, Debye-Waller factor to account for both thermal and structural disorders; (d) $\Delta E(0)$, inner potential correction; R factor indicates the goodness of the fit. S_0^2 was fixed to 0.855, according to the experimental EXAFS fit of Ir foil by fixing CN as the known crystallographic value. A reasonable range of EXAFS fitting parameters: $0.700 < S_0^2 < 1.000$; CN > 0; $\sigma^2 Å^2 > 0$; $|\Delta E_0| < 15$ eV; R factor < 0.02.

Table S9. EXAFS fitting parameters at the Co K-edge for various samples.

Sample	Shell	CN[a]	R(Å)[b]	σ^2(Å^2)[c]	ΔE_0(eV)[d]	R factor
Co foil	Co-Co	12*	2.49±0.01	0.0059	5.8	0.0023
Co_3O_4	Co-O	5.5*	1.92±0.01	0.0042	4.5	0.0133
	Co-Co	6.0*	2.86±0.01	0.0065	-2.2	
	Co-Co	6.0*	3.36±0.01	0.0057	-2.8	
$IrCoO_x$-2	Co-O	0.8±0.2	1.94±0.01	0.0030	5.6	0.0095
	Co-Co	3.3±0.7	2.88±0.01	0.0030	0.2	
	Co-Ir	1.1±0.2	3.10±0.01	0.0196	1.9	

Note: (a) CN, coordination number; (b) R, distance between absorber and backscatter atoms; (c) σ^2, Debye-Waller factor to account for both thermal and structural disorders; (d) ΔE(0), inner potential correction; R factor indicates the goodness of the fit. S_0^2 was fixed to 0.855, according to the experimental EXAFS fit of Ir foil by fixing CN as the known crystallographic value. A reasonable range of EXAFS fitting parameters: $0.700 < S_0^2 < 1.000$; CN > 0; σ^2Å^2 > 0; $|\Delta E_0| < 15$ eV; R factor < 0.02.

Table S10. The specific *in-situ* Raman peak data of 4-coordinated H_2O, 2-coordinated H_2O and free H_2O for $IrCoO_x$-2 during OER.

	4-coordinated H_2O		2-coordinated H_2O		Free H_2O	
Voltage	Percentage/ %	Raman Shift/cm^{-1}	Percentage/ %	Raman Shift/cm^{-1}	Percentage/ %	Raman Shift/cm^{-1}
OCP	22.75	3199.37	60.33	3385.20	16.92	3552.13
1.2 V	22.79	3210.42	60.78	3385.78	16.43	3538.79
1.3 V	23.70	3199.95	59.35	3387.51	16.95	3553.56
1.4 V	24.30	3197.36	58.97	3390.17	16.73	3561.47
1.5 V	24.42	3199.07	58.93	3390.93	16.65	3562.40
1.6 V	24.59	3193.89	57.53	3390.70	17.88	3566.71
1.7 V	24.80	3191.92	56.93	3391.48	18.27	3568.38
1.8 V	25.20	3192.49	56.84	3394.18	17.96	3569.72
Re-OCP	23.60	3203.79	60.33	3389.27	16.07	3552.20

Table S11. The specific *in-situ* Raman peak data of 4-coordinated H_2O, 2-coordinated H_2O and free H_2O for $IrCoO_x$-2@SnO_2 during OER.

	4-coordinated H_2O		2-coordinated H_2O		Free H_2O	
Voltage	Percentage/ %	Raman Shift/cm^{-1}	Percentage/ %	Raman Shift/cm^{-1}	Percentage/ %	Raman Shift/cm^{-1}
OCP	21.42	3215.26	62.39	3386.48	16.19	3536.82
1.2 V	21.25	3215.14	61.39	3383.26	17.36	3528.39
1.3 V	21.68	3219.03	61.09	3384.84	17.23	3526.62
1.4 V	22.11	3202.66	59.96	3384.72	17.93	3541.74
1.5 V	23.23	3199.56	59.59	3387.15	17.18	3546.10
1.6 V	24.33	3205.95	58.49	3389.92	17.18	3538.59
1.7 V	24.85	3195.48	57.42	3390.44	17.73	3549.16
1.8 V	24.70	3195.54	57.29	3389.72	18.01	3545.04
Re-OCP	22.56	3205.18	61.18	3387.97	16.26	3546.35

References

[1] J. Zhang, X. Fu, W. A. Goddard III, Y. Kang, et al. Tantalum-stabilized ruthenium oxide electrocatalysts for industrial water electrolysis, *Science* **2025**, 387, 48-55.

[2] S. Fang, H. Yang, P. Liu, H. Yang, et al. Tetragonal ZrO_2 supported low-iridium catalyst activating oxygen spillover stabilized lattice oxygen for proton exchange membrane water electrolysis, *Energy Environ. Sci.* **2025**, 18, 5470-5481.

[3] A. Kumar, M. Gil-Sepulcre, H. Tuysuz, et al. Iridium Single-Atom-Ensembles Stabilized on Mn-Substituted Spinel Oxide for Durable Acidic Water Electrolysis, *Adv. Mater.* **2024**, 36, 2401648.

[4] L. Deng, H. Chen, S. Peng, et al. Lewis Acid-Mediated Interfacial Water Supply for Sustainable Proton Exchange Membrane Water Electrolysis, *J. Am. Chem. Soc.* **2024**, 146, 35438-35448.

[5] H. Luo, F. Lv, S. Guo, et al. Atomic-Layer IrO_x Enabling Ligand Effect Boosts Water Oxidation Electrocatalysis, *J. Am. Chem. Soc.* **2024**, 146, 19327.

[6] X. Han, P. Liu, J. Chen, et al. Theoretical Prediction and Experimental Verification of IrO_x Supported on Titanium Nitride for Acidic Oxygen Evolution Reaction, *J. Am. Chem. Soc.* **2024**, 146, 16499.

[7] Q. Ji, X. Cai, W. Yan, et al. Operando identification of the oxide path mechanism with different dual-active sites for acidic water oxidation, *Nat. Commun.* **2024**, 15, 8089.

[8] L. Li, M. Luo, S. Guo, et al. Lanthanide-regulating Ru-O covalency optimizes acidic oxygen evolution electrocatalysis, *Nat. Commun.* **2024**, 15, 4974.

[9] Z. Wu, G. Wang, H. Wang, et al. Non-iridium-based electrocatalyst for durable acidic oxygen evolution reaction in proton exchange membrane water electrolysis, *Nat. Mater.* **2023**, 22, 100.

[10] J, Xu, Y. Zheng, S. Qiao, et al. $IrO_x \cdot nH_2O$ with lattice water-assisted oxygen

exchange for high-performance proton exchange membrane water electrolyzers, *Sci. Adv.*, **2023**, 9, eadh1718.

[11] X. Zheng, J. Yang, Y. Li, et al. Ir-Sn pair-site triggers key oxygen radical intermediate for efficient acidic water oxidation, *Sci. Adv.* **2023**, 9, eadi8025.

[12] W. Zhu, F. Yao, H. Liang, et al. Direct Dioxygen Radical Coupling Driven by Octahedral Ruthenium-Oxygen-Cobalt Collaborative Coordination for Acidic Oxygen Evolution Reaction, *J. Am. Chem. Soc.* **2023**, 145, 17995-18006.

[13] H. Gao, Z. Xiao, S. Wang, et al. Reducing the Ir-O Coordination Number in Anodic Catalysts based on IrO_x Nanoparticles towards Enhanced Proton-exchange membrane Water Electrolysis, *Angew. Chem. Int. Ed.* **2023**, 62, e202313954.

[14] Z. Shi, J. Ge, W. Xing, et al. Enhanced Acidic Water Oxidation by Dynamic Migration of Oxygen Species at the Ir/Nb_2O_{5-x} Catalyst/Support Interfaces, *Angew. Chem. Int. Ed.* **2022**, 61, e202212341.

[15] L. Zu, A. Yu, D. Zhao, et al. Self-Assembly of Ir-Based Nanosheets with Ordered Interlayer Space for Enhanced Electrocatalytic Water Oxidation, *J. Am. Chem. Soc.* **2022**, 144, 2208.

[16] S. Hao, X. Zhang, S. Jin, et al. Torsion strained iridium oxide for efficient acidic water oxidation in proton exchange membrane electrolyzers, *Nat. Nanotechnol.* **2021**, 16, 1371.

[17] Q. Dang, X. Huang, M. Shao, et al. Iridium metallene oxide for acidic oxygen evolution catalysis, *Nat. Comm.* **2021**, 12, 6007.

[18] W. Shi, Y. Xu, B. Zhang, et al. Ultrastable supported oxygen evolution electrocatalyst formed by ripening-induced embedding, *Science* **2025**, 387, 791-796.

[19] A. Li, S. Kong, R. Nakamura, et al. Atomically dispersed hexavalent iridium oxide from MnO_2 reduction for oxygen evolution catalysis, *Science*, **2024**, 384, 666-670.

[20] Y. Wang, H. Chen, X. Zou, et al. Nano-metal diborides-supported anode catalyst with strongly coupled TaO_x/IrO_2 catalytic layer for low-iridium-loading proton exchange membrane electrolyzer, *Nat. Commun.* **2023**, 14, 5119.

[21] K. Sun, H, Chen, X. Zou, et al. Highly Efficient and Durable Anode Catalyst Layer Constructed with Deformable Hollow IrO_x Nanospheres in Low-Iridium PEM Water Electrolyzer, *Angew. Chem. Int. Ed.* **2025**, 64, e202504531.

[22] W. Liu, Z. Fu, Z. Liang, et al. Extremely Active and Robust Ir-Mn Dual-Atom Electrocatalyst for Oxygen Evolution Reaction by Oxygen-Oxygen Radical Coupling Mechanism, *Angew. Chem. Int. Ed.* **2024**, 63, e202411014.

[23] H, Su, S, Lian, Q, Liu, et al. Tensile straining of iridium sites in manganese oxides for proton-exchange membrane water electrolysers, *Nat. Commun.* **2024**, 15, 95.
