# Supplementary material for: Paracrystalline IrCoOx stabilization by rutile SnO2 towards durable acidic oxygen evolution
Source: Nat Commun. 2026 Aug 21;17:10577. doi: 10.1038/s41467-026-76919-0 (PMC13646295; doi:10.1038/s41467-026-76919-0)
Supplement: Supplementary file 2 [file 41467_2026_76919_MOESM2_ESM.pdf]

Description of Additional Supplementary Files

File name: Supplementary Data 1

Description: The original cif structure files for DFT models (including $IrCoO_x$, $IrCoO_x$-Co_{vac}, $IrCoO_x$@SnO_2 and $IrCoO_x$@SnO_2-Co_{vac}) and MD simulation structures (including $IrCoO_x$-0V, $IrCoO_x$-1.5V, $IrCoO_x$@SnO_2-0V and $IrCoO_x$@SnO_2-1.5V).
