# Supplementary material for: Paracrystalline IrCoOx stabilization by rutile SnO2 towards durable acidic oxygen evolution
Source: Nat Commun. 2026 Aug 21;17:10577. doi: 10.1038/s41467-026-76919-0 (PMC13646295; doi:10.1038/s41467-026-76919-0)
Supplement: Supplementary file 3 [file 41467_2026_76919_MOESM4_ESM.pdf]

nature portfolio

Peer Review File

Paracrystalline $IrCoO_x$ Stabilization by Rutile SnO_2 Towards Durable Acidic Oxygen Evolution

Corresponding Author: Professor Jianlin Shi

This file contains all reviewer reports in order by version, followed by all author rebuttals in order by version.

Version 0:

Reviewer comments:

Reviewer #1

(Remarks to the Author)
In this manuscript, the authors report IrCoOx-2 as a novel catalyst for the oxygen evolution reaction (OER) in acidic media. Through in-situ heating TEM, they directly verify the formation of a unique paracrystalline IrCoOx phase. By compositing with SnO_2, the authors successfully address the long-standing activity–stability trade-off in acidic OER, achieving both high performance and durability. Furthermore, combining DFT and AIMD calculations with in-situ SERS analysis, they reveal that the SnO_2 support not only stabilizes the paracrystalline structure but also optimizes the interfacial water environment, thereby facilitating reversible oxygen-species cycling and efficient proton transfer during OER. The work demonstrates significant novelty and is suitable for publication after the following points are addressed:
1. In lines 147 and 153, “KOH” should be corrected to “NaOH.”
2. What is the stability of IrCoOx-2@SnO_2 when tested in a practical PEMWE electrolyzer at high current densities (e.g., 5 A cm^{-2}), using commercial Pt/C as the cathode catalyst?
3. To assess the practical viability of the newly developed IrCoOx-2@SnO_2 anode, its compatibility with common cathode materials must be evaluated. Specifically, does pairing it with a commercial Pt/C cathode influence the overall stability and cycling performance of the electrolysis system? Additionally, investigating other potential cathode catalysts could provide insights into optimal pairings. Comparative experimental data would be highly valuable in clarifying these points.
4. The ball-milling rate (with 300 rpm found optimal) and the amount of SnO_2 added appear to significantly affect catalyst performance. Please provide a detail discussion or explanation for the selection of these parameters.
5. The post-reaction IrCoOx-2@SnO_2 catalyst exhibits a yolk-like morphology with a SnO_2 core and an IrCoOx-2 shell. What is the possible formation mechanism of this structure? Could this transformation accelerate Ir dissolution, and if so, how might it impact long-term stability?

Reviewer #2

(Remarks to the Author)
This study by Tian et al. reports the development of an IrCoOx@SnO_2 catalyst. The authors adopt a dual approach to advance OER performance and durability, boosting the intrinsic activity via Co doping into iridium oxide, and concurrently enhancing stability by supporting the material on SnO_2. The catalyst demonstrates remarkable OER durability, operating steadily for over 3,000 hours at a current density of 2 A cm^{-2}. This work presents highly interesting findings. Below are detailed comments.
(1) The schematic diagram in Scheme 1 illustrates the structural innovation and advantages of the catalyst. However, corresponding experimental evidence, particularly regarding the intrinsic role of Co doping in enhancing performance, is lacking.
(2) Although the authors prepared an IrOx catalyst, its electrochemical performance was not evaluated.
(3) The XRD peak broadening is relatively subtle, which could also be attributed to the small nanoparticle size.
(4) What is the thickness of the membrane shown in Figure 3g? Additionally, it is necessary to calculate the energy efficiency of the hydrogen to electricity conversion process.
(5) Deuterium isotope tracing experiments should be included to further confirm the lattice oxygen oxidation mechanism (LOM).

Reviewer #3

(Remarks to the Author)
In their manuscript, the authors present a new method of stabilizing the OER catalyst IrCoOx, which they identify as paracrystalline based on HAADF-STEM, by adding SnO2. The electrochemical stability and metal dissolution rate of the SnO2-modified material was compared with the SnO2-free phase. From correlating these results to other comparative ex-situ and in-situ characterizations, the authors conclude that the interaction of the synthesized IrCoOx phase with the rutile structure of SnO2 influences its structural properties and reduces metal dissolution, the metal oxidation state and particle agglomeration. In addition, theoretical calculations and SERS data support an OER-promoting influence of the SnO2 support on electrochemical interface properties of the catalyst.

Although the idea of stabilizing the catalytic phase by adding a stable rutile structure seems to be a good approach, the manuscript is currently in a very raw state. The results are partly not well described, and some crucial information is missing in the text, which is also not well structured (e.g. sample names are used without explanation in the main text, the sources are interrupted by other content). Moreover, some of the data analyses need to be revised or explained in more detail. For some of the conclusions the authors draw, additional measurements may be necessary. Overall, my suggestion is that the manuscript can only be published after major revisions addressing at least the following aspects:

1. In the introduction, I missed a short summary of previous work on IrCoOx, e.g. https://doi.org/10.1016/j.apcatb.2020.118820 and others, and a discussion how previous results are correlated to the new insights.
2. Also, using SnO2 for stabilizing Ir-based catalysts is not a completely new approach. The authors should include a brief overview of results presented in the literature.
3. How did the authors derive the space group of the paracrystalline IrCoOx structure?
4. As mentioned, the sample names appear out of the blue in the main text. I would suggest including one sentence here, which describes the main variation between IrCoOx-1, IrCoOx-2 and IrCoOx-3, and a hint to look into the materials section for more details.
5. In the paragraph in which the authors mention the modified one-step molten salt method in the main text for the first time, they should include a hint that the method is described in detail in the material section.
6. The authors should briefly explain how they quantify the “amorphous degree” from XRD.
7. In the sentence “The calculated Ir4+/Ir0 ratio in X-ray photoelectron spectroscopy (XPS, Table S1) decreases progressively with Co incorporation (Figs. 1f and S5)32,33, a modulation that is anticipated to enhance the catalyst’s operational stability during OER.”, do the given sources explain why the catalyst stability is enhanced by the observed modulation? In this case, the should be placed to the end of the sentence, otherwise the authors should prove the statement by other sources.
8. The authors should explain how they attribute the different components they used to fit the O1s XPS spectra to the respective species. Moreover, especially the term “metal oxygen” for the component in XPS fitting seems to be a bit misleading, as metallic usually doesn’t contain oxygen. I would recommend using a more comprehensible term like e.g., following the authors’ explanation in the text, “disordered oxygen”.
9. In the paragraph “Evolution mechanism for paracrystalline structure” the authors mention the use of KOH, but the subsequent chemical equations describe reactions involving NaOH instead. Please clarify which substance was used.
10. Regarding the sentence “Whereas the IrOx sample is highly amorphous (Figs. S9-S11), the incorporation of Co induces a slight increase in structural order (i.e., paracrystalline), which is corroborated by a discernible right-shift in the XRD peaks.”: What is a right-shift? Usually shifts of Bragg-reflections in XRD indicate lattice parameter changes. The authors should rather discuss the intensity differences and broadenings.
11. Why did the authors decide to use IrO2 as a reference for OER activity, which is a rather poor catalyst? State of the art electrolyzers usually use metallic Ir particles, which form a hydrous active surface. Wouldn’t including this IrOx type catalyst as a reference yield a fairer comparison?
12. Is a “yolk-like” morphology different from a core-shell morphology? If not, wouldn’t this be a more comprehensible term?
13. The discussion of the EXAFS results needs to be revised: The maxima of the FT EXAFS do not directly indicate average bond distances, as the main text implies, because the oscillations include not only single but multi scattering paths and contain energy-dependent phase shifts that shift peaks away from the true interatomic distances. Therefore, a model of the Ir-containing phase needs to be considered and fit to the FT-EXAFS. The model must be explained and all fit parameters including statistical errors need to be reported in the SI, e.g. in the form of a table. Given the small differences in the FT EXAFS shown in the manuscript, discussing the errors of the bond distances extracted from the fit is crucial for understanding the significance of differences.
14. What additional information do the Wavelet transform graphs provide, which look very similar for both samples?
15. Why did the authors only study the Ir L-edge? Combination with Co K-edge EXAFS could yield information about the Co-M and Ir-M distances, which might support the proposed substitutional paracrystalline lattice structure, in which Co and Ir occupy equivalent sites.
16. How do the authors derive the conclusion that SnO2 incorporation prevents overoxidation? Do they have evidence for different oxidation states from XANES or from fitting the Ir 4f XPS?
17. When stating the pH-dependent slopes in the text, please don’t omit the units.
18. How can the authors make sure that a different pH dependent slopes are unambiguously connected with the AEM and LOM? Even if it is based on previous results from literature, these should be mentioned and their transferability to the material investigated in this work should be discussed.
19. The authors should explain why a lower pH-slope is connected with a lower *OH coverage.
20. The presented data represent rather indirect indications for the deduced mechanisms. In the current version, these mechanisms are not based on convincing evidence and so they can only represent a speculative interpretation and should

also be described and discussed as such!
21. What do the authors mean by the term "oxygen species cycling"? This should be defined in the text.

Version 1:

Reviewer comments:

Reviewer #2

(Remarks to the Author)
Regarding the third question raised by Reviewer 1, the authors conducted a set of control experiments with the commercial PtRu/C as the cathode material. However, this material operated stably for only 40 hours, which contrasts sharply with the stability of Pt/C as the cathode material (approximately 4,000 hours, as shown in Figure 3g). Although both are platinum-based materials, the difference in stability amounts to two orders of magnitude. What accounts for this significant discrepancy? Detailed discussion should be provided in the response. Furthermore, for the long-term stability test lasting 4,000 hours originally presented in Figure 3g, the original V–t curve must be supplied in the Supporting Information, rather than only a selection of data points.

This manuscript has been well revised with added experimental data and discussion, and it is acceptable for publication.

Reviewer #3

(Remarks to the Author)
In the updated version, the authors put some effort into addressing the remarks, which was partly successful. I still highly recommend the following remaining aspects to be considered prior to publication:
• Regarding 1 and 2: It is good to see that at least the previous studies are mentioned now, but for a journal of this rank I would recommend adding a brief overview of their outcome and how the authors' work is building up on that, i.e. which additional questions they address, which were left open so far.
• Regarding 3 and 8: It would be great if the authors could include the explanation into the SI to make this aspect more transparent to the readers.
• Regarding 6: In this case, the following sentence in line 108 needs to be adjusted: "Notably, the amorphous degree of IrCoOx sample increases with the higher loading of Co element." It implies that the amorphous degree is derived from XRD and it must be made clear how this conclusion was derived.
• Regarding 13: The following passage should be completely rewritten: "The Fourier-transformed extended X-ray absorption fine structure (EXAFS, Fig. 4e) 292 reveals a longer Ir-O bond in IrCoOx-2 compared with commercial IrO2, attributed to 293 the expanded lattice owing to Co incorporation. Moreover, the presence of Co-O-Ir 294 signal in Fig. S41 also supports the substitution of Co into the IrOx lattice. Crucially, 295 this Ir-O bond length is preserved in IrCoOx-2@SnO2, indicating that SnO2 does not 296 perturb the paracrystalline structure. From Fig. 4f, the Ir-O bond of IrCoOx-2 catalyst 297 at ~1.6 Å shifts to larger value after OER while IrCoOx-2@SnO2 does not really change, 298 which is further supported by the fitting results (Fig. S42 and Table S8)." As already alluded to in the last revision, it contains statements which are just wrong. It is not possible to draw any conclusions about the bond length from the FT-EXAFS without fitting! Moreover, the fitting results indicate very minor differences in terms of the bond length change between the both materials (e.g. Ir-O[IrCoOx-2]: 1.99+/-0.01 -> 2.02+/-0.01 and Ir-O[IrCoOx-2 @SnO2]: 2.00+/-0.01 -> 2.02+/-0.01) The significance of these differences should be discussed in the main text.
• Regarding 14: From the explanation given in the text and the rebuttal letter, the wavelet transform shows a shift of the R value. The problem with this explanation is that a simple shift in R-value should be more clearly visible in the FT-EXAFS, in which one can barely see any difference by eye. So, the clearly visible shift in the wavelet transform could only be explained based on the k-space dependence, which should be implemented in the explanation (which should not only be given in the rebuttal letter).
• Regarding 15: Good to see that the authors were able to measure the Co K-edge EXAFS, too. But again, the interpretation can only be done based on EXAFS fitting as in case of the Ir L-edge. In the statement "Moreover, the presence of Co-O-Ir 294 signal in Fig. S41 also supports the substitution of Co into the IrOx lattice." it appears dubious, that the second scattering shell is attributed only to a Co-O-Ir path. If that was the case, where are the usually predominant Co-Co and Ir-Co scattering contributions represented? What the authors would need to do to check whether the structure is substitutional would be to compare the Co-M distances extracted from fitting the FT-EXAFS at the Co K-edge with the Ir-M distances extracted from fitting the FT-EXAFS at the Ir L-edge.
• Regarding 19: Please implement the correlation between the MOR and the *OH coverage into the explanation in the main text to make it more comprehensible.

Version 2:

Reviewer comments:

Reviewer #2

(Remarks to the Author)
The authors supplied critical stability test data in the revised supporting information, and comprehensively addressed the points raised by the other reviewer.

Reviewer #3

(Remarks to the Author)
In the updated version, the authors have addressed all concerns adequately and I suggest to proceed with the publication process.

Open Access This Peer Review File is licensed under a Creative Commons Attribution 4.0 International License, which permits use, sharing, adaptation, distribution and reproduction in any medium or format, as long as you give appropriate credit to the original author(s) and the source, provide a link to the Creative Commons license, and indicate if changes were made.
In cases where reviewers are anonymous, credit should be given to 'Anonymous Referee' and the source.
The images or other third party material in this Peer Review File are included in the article’s Creative Commons license, unless indicated otherwise in a credit line to the material. If material is not included in the article’s Creative Commons license and your intended use is not permitted by statutory regulation or exceeds the permitted use, you will need to obtain permission directly from the copyright holder.
To view a copy of this license, visit https://creativecommons.org/licenses/by/4.0/

Point-to-point response and revisions

(Original Manuscript Number: NCOMMS-25-101304-T)

Note: In the response manuscript, comments suggested by reviewers are in **black font**, our responses are in blue font, and revised parts in the text and SI are highlighted in yellow. The figures in our Manuscript, Supplementary Information and Response documents are displayed in the form of **Fig. XX**, **Fig. SXX** and **Fig. RXX**, respectively.

Responses to Reviewer 1

Comments: In this manuscript, the authors report $IrCoO_x$-2 as a novel catalyst for the oxygen evolution reaction (OER) in acidic media. Through in-situ heating TEM, they directly verify the formation of a unique paracrystalline $IrCoO_x$ phase. By compositing with SnO_2, the authors successfully address the long-standing activity–stability trade-off in acidic OER, achieving both high performance and durability. Furthermore, combining DFT and AIMD calculations with in-situ SERS analysis, they reveal that the SnO_2 support not only stabilizes the paracrystalline structure but also optimizes the interfacial water environment, thereby facilitating reversible oxygen-species cycling and efficient proton transfer during OER. The work demonstrates significant novelty and is suitable for publication after the following points are addressed:

Response: Thank you very much for your positive recommendation. We appreciate the reviewer's time and efforts in reviewing the manuscript. We have supplemented several experiments and revised the manuscript according to reviewers' suggestions, and we hope the revisions have addressed your valuable comments. Please find our point-by-point responses below.

1. In lines 147 and 153, "KOH" should be corrected to "NaOH".

Response: Thank you very much for your careful review. We apologize for our negligence, and have corrected to "NaOH" in the revised manuscript. (Page 8, Line 15/16/22)

2. What is the stability of $IrCoO_x$-2@SnO_2 when tested in a practical PEMWE electrolyzer at high current densities (e.g., 5 A cm^{-2}), using commercial Pt/C as the cathode catalyst?

Response: Many thanks for this insightful question. We fully acknowledge that testing at an ultra-high current density of 5A cm^{-2} in a practical PEMWE electrolyzer is highly challenging under our current laboratory conditions, as the overall performance is constrained by multiple factors beyond the catalyst itself, including the conductive substrate (Ti felt, carbon fiber paper), proton exchange membrane, electrode channel and assembly process, etc. Despite our best efforts, a current density of 5 A cm^{-2} cannot yet be achieved due to limitations of our laboratory-scale electrolyzer setup. Furthermore, through discussions with industry partners, we have learned that operating at such an extreme current density is both cost-prohibitive and unnecessarily harsh, as it leads to severe efficiency loss (cell voltage often > 2.0-2.2 V) and accelerated membrane degradation, making it impractical for real-word applications. Therefore, we believe that the long-term stability test conducted at 2 A cm^{-2} for 3880 h already provides strong evidence for the excellent durability and practical application potential of the as-prepared $IrCoO_x$-2@SnO_2 catalyst.

3. To assess the practical viability of the newly developed $IrCoO_x$-2@SnO_2 anode, its compatibility with common cathode materials must be evaluated. Specifically, does pairing it with a commercial Pt/C cathode influence the overall stability and cycling performance of the electrolysis system? Additionally, investigating other potential cathode catalysts could provide insights into optimal pairings. Comparative experimental data would be highly valuable in clarifying these points.

Response: Thank you for this valuable comment. In an electrolysis system, both the anode and cathode catalysts critically determine the overall performance. To fairly evaluate our $IrCoO_x$-2@SnO_2 anode, we deliberately chose commercial Pt/C as the cathode catalyst, as it remains the only industrially viable cathode material for acidic PEMWE applications. Through discussions with industry partners, we confirmed that

even advanced Pt-based catalysts have not yet seen practical deployment, and Pt/C continues to be the benchmark.

Nevertheless, in response, we tried to conduct the comparative experiment using other commercial PtRu/C as an alternative catalyst under the identical conditions. As shown in **Fig. R1** (see below), the cell performance with PtRu/C cathode is obviously inferior to that with commercial Pt/C cathode with the same loading of noble metal. These results confirm that pairing our $IrCoO_x$-2@SnO_2 anode with commercial Pt/C cathode represents the optimal and most practically relevant combination.

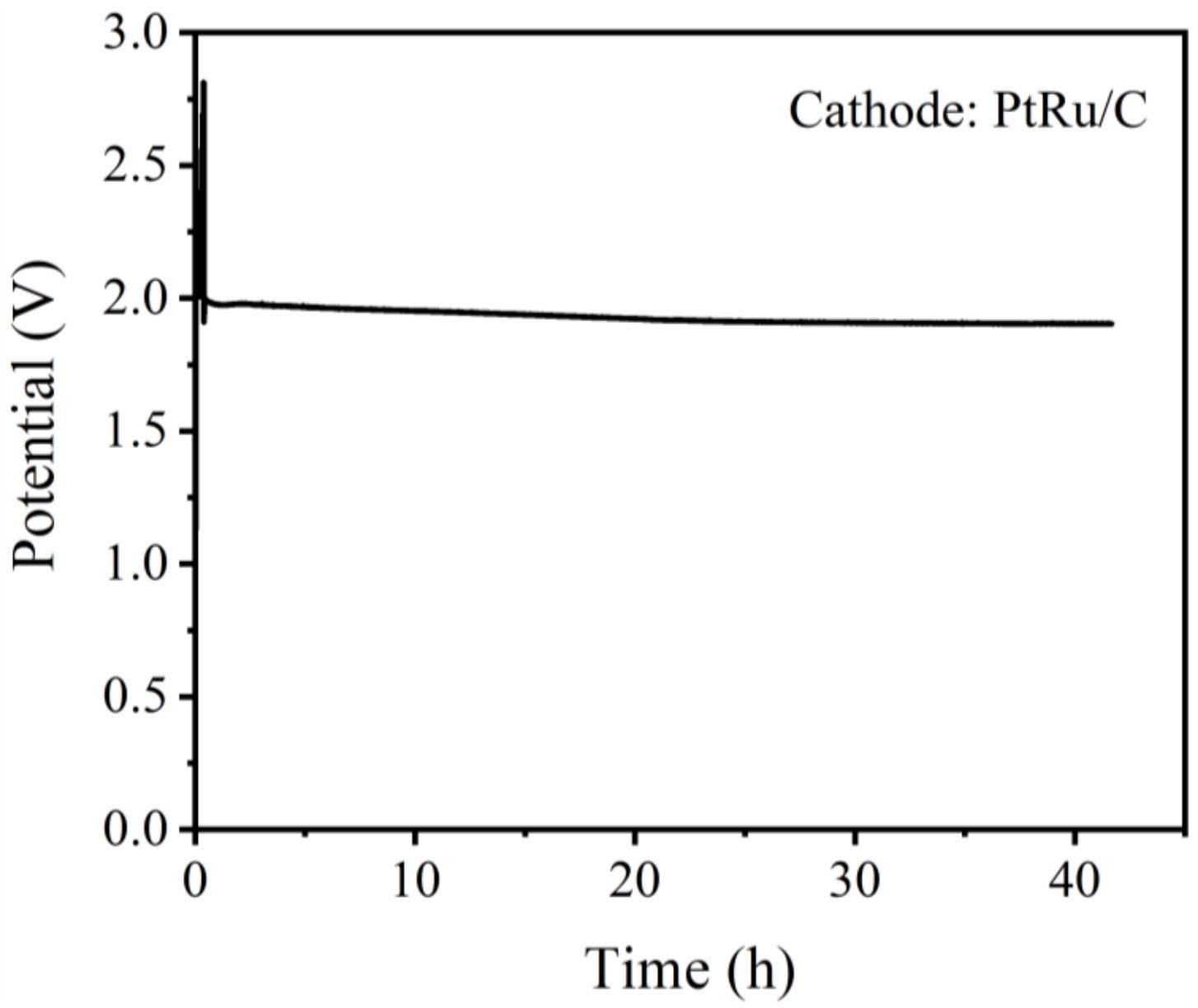

Fig. R1. Stability test of the PEMWE electrolyzer operated at 1 A cm^{-2}, employing $IrCoO_x$-2@SnO_2 and commercial PtRu/C as anode and cathode catalysts, respectively.

4. The ball-milling rate (with 300 rpm found optimal) and the amount of SnO_2 added appear to significantly affect catalyst performance. Please provide a detail discussion or explanation for the selection of these parameters.

Response: Thank you very much for your constructive suggestions. We have supplemented the manuscript with a detailed discussion regarding the selection of ball-milling parameters, and the related texts read: "A series of control samples were prepared by varying the ball-milling speed and the amounts of SnO_2 introduced.

Through the direct comparisons (**Figs. 4k-4l**) among the several chronopotentiometry curves at 10 mA cm^{-2}, the optimal parameters were determined to be a ball-milling rate of 300 rpm and m($IrCoO_x$-2):m(SnO_2) ratio of 2:1. It is believed that excessively high ball-milling speed may damage the paracrystalline structure of $IrCoO_x$-2 catalyst. Furthermore, excessive SnO_2 loading was found to compromise stability, likely due to inadequate mixing and dispersion." (Page 15, Line 22-28).

5. The post-reaction $IrCoO_x$-2@SnO_2 catalyst exhibits a yolk-like morphology with a SnO_2 core and an $IrCoO_x$-2 shell. What is the possible formation mechanism of this structure? Could this transformation accelerate Ir dissolution, and if so, how might it impact long-term stability?

Response: Thank you very much for the insightful questions. Regarding the formation mechanism of the yolk-like core-shell structure (SnO_2 core@$IrCoO_x$-2 shell), we attribute it primarily to the significant difference in electrical conductivity between $IrCoO_x$-2 and SnO_2. Under the strongly acidic local environment and continuous bubble impact associated with oxygen evolution, the less conductive $IrCoO_x$-2 undergoes physical detachment or migration, followed by reattachment onto the stable SnO_2 substance, thereby forming the observed core-shell architecture.

Concerning Ir dissolution and long-term stability, this spontaneously formed core-shell structure is actually beneficial. The $IrCoO_x$-2 shell provides high OER activity, while the SnO_2 core maintains high conductivity and structural stability. Multiple literature reports support that such a configuration does not accelerate Ir dissolution but rather enhances durability (*Adv. Funct. Mater.*, **2023**, 33, 2306889; *J. Mater. Chem. A*, **2023**,11, 3399-3407; *Small*, **2023**, 19, 2205665). Furthermore, the DFT structure model presented in our manuscript accurately reflects this core-shell configuration with an outer $IrCoO_x$-2 and an inner SnO_2, and demonstrates the stability enhancement mechanism conferred by the SnO_2 core.

Responses to Reviewer 2

Comments: This study by Tian et al. reports the development of an $IrCoO_x@SnO_2$ catalyst. The authors adopt a dual approach to advance OER performance and durability, boosting the intrinsic activity *via* Co doping into iridium oxide, and concurrently enhancing stability by supporting the material on SnO_2. The catalyst demonstrates remarkable OER durability, operating steadily for over 3,000 hours at a current density of 2 A cm^{-2}. This work presents highly interesting findings. Below are detailed comments.

Response: Thank you very much for your positive recommendation. We appreciate the reviewer's time and efforts in reviewing the manuscript. We have supplemented several experiments and revised the manuscript according to reviewers' suggestions, and we hope the revisions have addressed your valuable comments. Please find our point-by-point responses below.

1. The schematic diagram in Scheme 1 illustrates the structural innovation and advantages of the catalyst. However, corresponding experimental evidence, particularly regarding the intrinsic role of Co doping in enhancing performance, is lacking.

Response: Thank you very much for your constructive comment. To provide direct experimental evidence for the intrinsic role of Co doping in enhancing catalytic performance, we have compared the OER activity of IrO_x and $IrCoO_x$ catalysts. As shown in **Fig. R2** (see below), the OER activity was obviously improved after the introduction of Co, confirming the beneficial effect of Co doping.

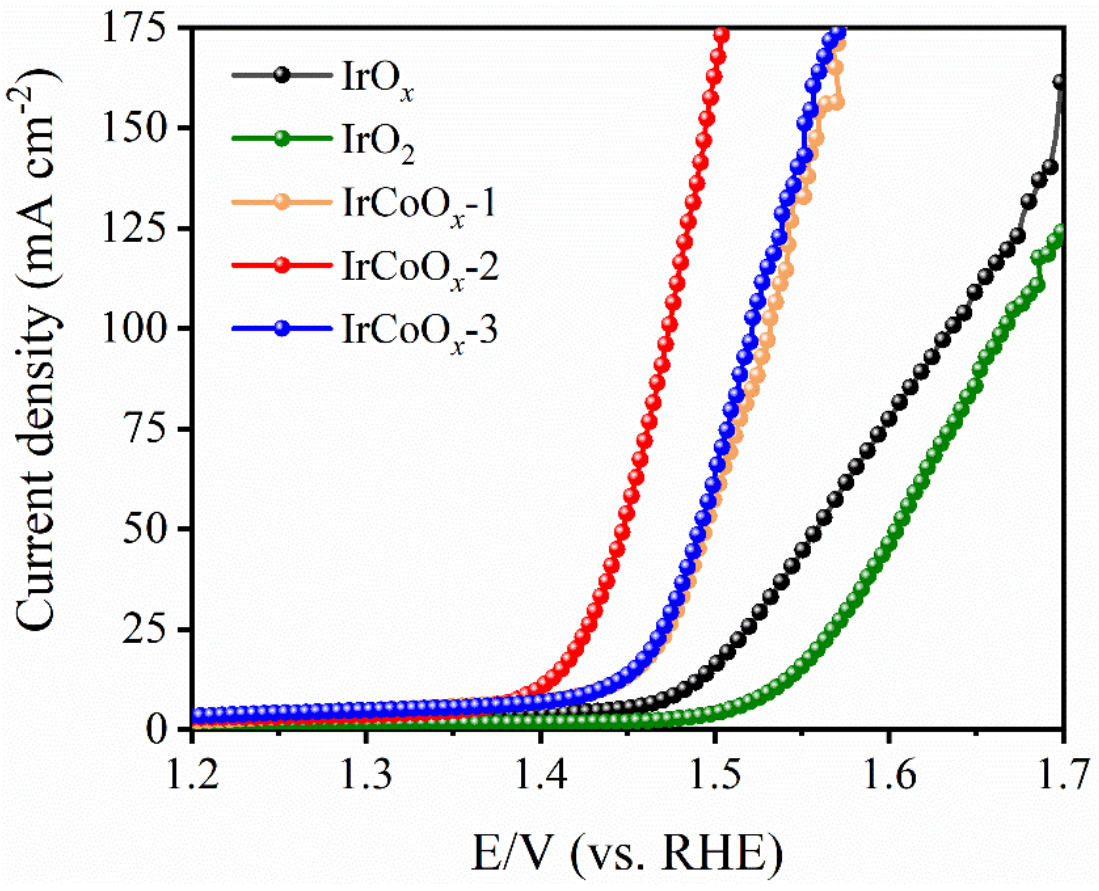

Fig. R2. LSV curves of IrO_x, $IrCoO_x$-1, $IrCoO_x$-2, $IrCoO_x$-3 and commercial IrO_2 catalysts in 0.5 M H_2SO_4.

2. Although the authors prepared an IrO_x catalyst, its electrochemical performance was not evaluated.

Response: Thanks a lot for your careful review. We have now evaluated the electrochemical performance of the as-prepared IrO_x catalyst, including LSV curve (**Fig. S12a**, see below) and ESI result (**Fig. S12b**, see below). As shown in above **Figure R2**, the OER activity of as-prepared IrO_x is higher than that of commercial IrO_2 owing to its amorphous nature, yet remains moderately lower than that of as-prepared $IrCoO_x$ catalysts. Corresponding description has been added to the revised manuscript, which reads: "The linear sweep voltammetry (LSV) curves in **Fig. 3a** reveal that $IrCoO_x$-2 exhibits the best OER activity in 0.5 M H_2SO_4, requiring only 170 mV overpotential to reach 10 mA cm^{-2}, which is significantly lower than that of commercial IrO_2 (305 mV), as-prepared IrO_x (253 mV, Fig. S12) and recently reported catalysts (Table S4)." (Page 10, Line 12-15)

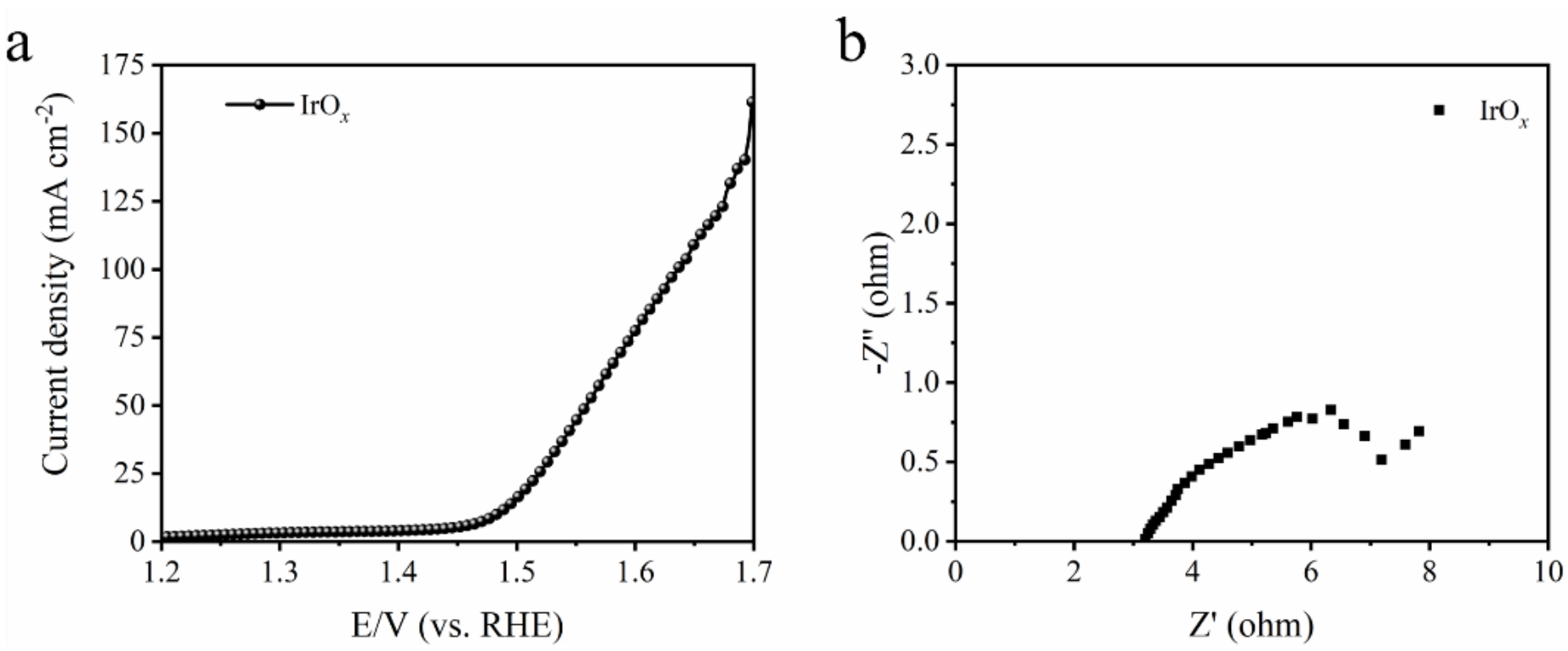

Fig. S12. (a) LSV curve and (b) ESI curve of the as-prepared IrO_x catalyst.

3. The XRD peak broadening is relatively subtle, which could also be attributed to the small nanoparticle size.

Response: Thank you very much for this comment. We acknowledge that the broadening XRD peak may indeed arise from either amorphization or small nanoparticle size. In our work, however, the assignment of a paracrystalline phase is

supported by multiple complementary characterizations including XRD, TEM, HRTEM and SAED images. Specifically, as shown in **Fig. 1b** (see below), $IrCoO_x$-2 consists of nanoparticles approximately 10-20 nm in size, which would not typically produce such featureless XRD peaks. Moreover, no any lattice fringes can be observed in HRTEM image (**Fig. 1c**, see below), and the SAED pattern (inset of **Fig. 1c**, see below), together with *in-situ* heating SAED images (**Fig. 2**, see below), clearly distinguishes the paracrystalline nature from crystalline or fully amorphous materials. Thus, while size effects cannot be completely excluded, the combined evidence strongly supports the paracrystalline assignment.

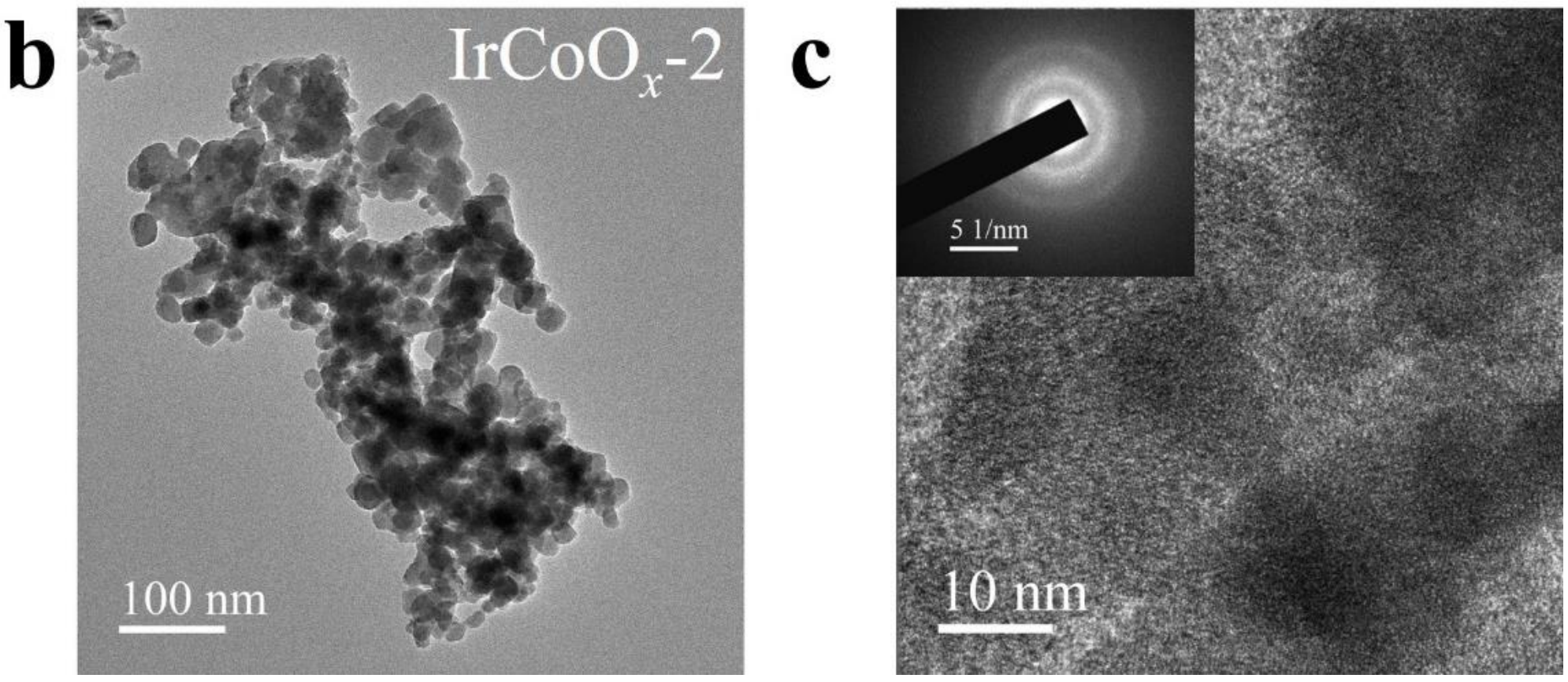

Fig. 1 (b) TEM and (c) HRTEM (inset: SAED pattern) images of $IrCoO_x$-2 sample.

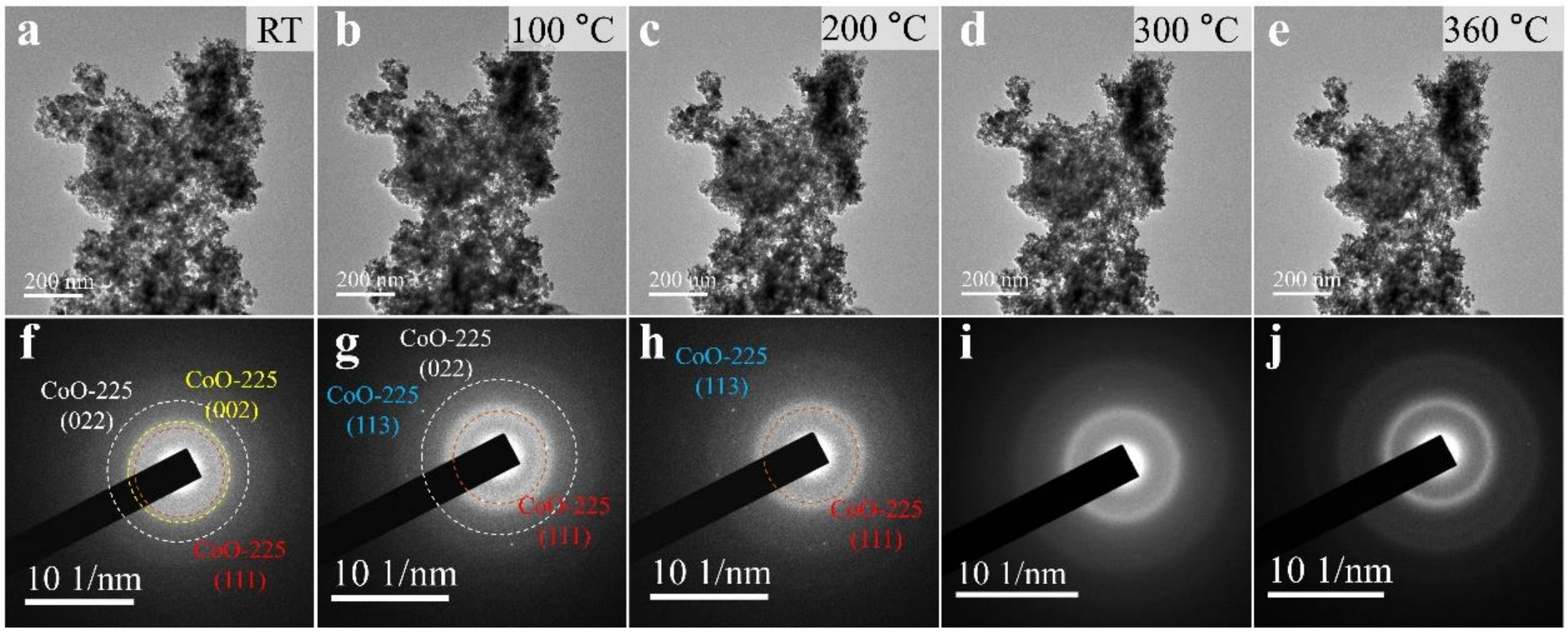

Fig. 2 TEM images and the corresponding SAED patterns of IrOx and CoOx mixture at (a, f) RT, (b, g) 100 ° C, (c, h) 200 ° C, (d, i) 300 ° C and (e, j) 360 ° C.

4. What is the thickness of the membrane shown in Figure 3g? Additionally, it is necessary to calculate the energy efficiency of the hydrogen to electricity conversion

process.

Response: Thanks a lot for your question and suggestion. Regarding the membrane thickness, the PEMWE measurement employed a Nafion-212 membrane, which has a thickness of ~50 μm.

Regarding the energy efficiency of the hydrogen-to-electricity conversion process, we would like to clarify that the integrated water splitting-fuel cell linkage system was constructed solely to visually demonstrate the reliability of the produced H_2. In practice, the energy efficiency of hydrogen-to-electricity conversion is determined exclusively by the commercial fuel cell device itself, and therefore does not affect the electrochemical water splitting process nor diminish the novelty of this study. We hope this clarification addresses the reviewer's concerns.

5. Deuterium isotope tracing experiments should be included to further confirm the lattice oxygen oxidation mechanism (LOM).

Response: Thank you very much for your professional suggestion. We have now performed O^{18} isotope-labeling differential electrochemical mass spectrometry (DEMS) to confirm the LOM process for as-prepared $IrCoO_x$-2@SnO_2 catalyst.

Specifically, we performed O^{18} labeling by immersing the working electrode in 0.5 M H_2SO_4 prepared with H_2O^{18} and multiple LSV scans were conducted between 1.1 and 1.5 V (vs. RHE). Then, after rinsed 10 times with H_2O^{16}, the same electrochemical procedure was repeated in 0.5 M H_2SO_4 prepared with H_2O^{16}, while recording the gaseous products and their isotopic composition in real time by mass spectrometry. As shown in **Fig. S52** (see below), the detection of $O^{18}O^{18}$ and $O^{16}O^{18}$ peaks confirms the exchange of O^{18} into the lattice of $IrCoO_x$-2@SnO_2 catalyst, providing direct evidence for the LOM pathway.

Corresponding description has been added to the revised manuscript, and the related texts read: "To clearly elucidate the OER pathway of as-prepared $IrCoO_x$-2@SnO_2, O^{18} isotope-labeling differential electrochemical mass spectrometry (DEMS) was employed. Firstly, the working electrode was immersed in 0.5 M H_2SO_4 prepared with

H_2O^{18} and then multiple LSV scans were made between 1.1-1.5 V (vs. RHE). After rinsed 10 times with H_2O^{16}, the same electrochemical procedure was repeated in 0.5 M H_2SO_4 prepared with H_2O^{16}, while recording the gaseous products and their isotopic composition in real time by mass spectrometry. As shown in Fig. S52, the presences of $O^{18}O^{18}$ and $O^{16}O^{18}$ peaks confirm the exchange of O^{18} into the lattice of IrCoO$_x$-2@SnO$_2$, thus consolidating the LOM mechanism." (Page 18, Line 17-25)

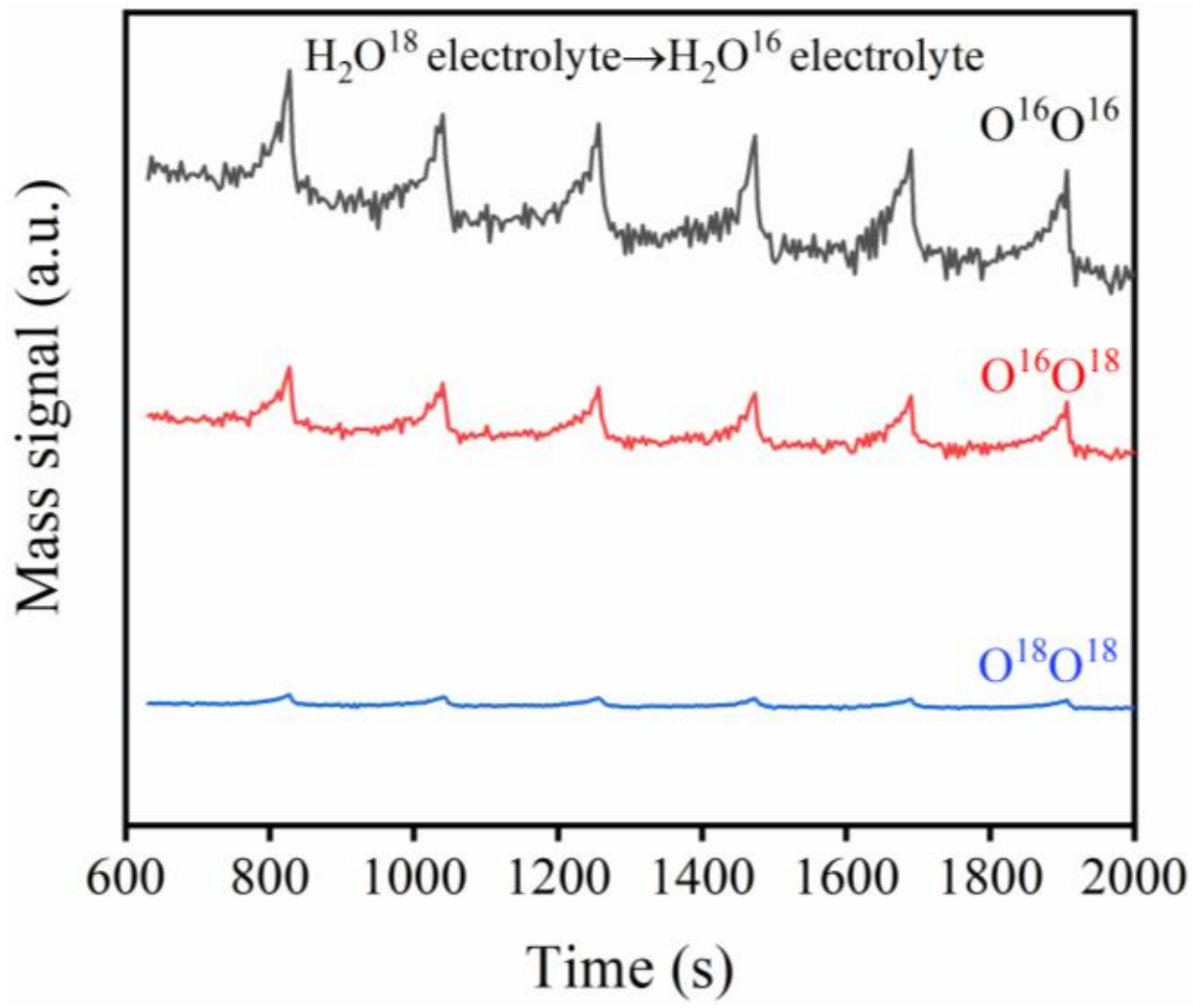

Fig. S52. DEMS signals of O_2 products for O^{18} labeled IrCoO$_x$-2@SnO$_2$ in 0.5 M H_2SO_4 using H_2O^{16} as electrolyte.

Responses to Reviewer 3

Comments: In their manuscript, the authors present a new method of stabilizing the OER catalyst $IrCoO_x$, which they identify as paracrystalline based on HAADF-STEM, by adding SnO_2. The electrochemical stability and metal dissolution rate of the SnO_2-modified material was compared with the SnO_2-free phase. From correlating these results to other comparative ex-situ and in-situ characterizations, the authors conclude that the interaction of the synthesized $IrCoO_x$ phase with the rutile structure of SnO_2 influences its structural properties and reduces metal dissolution, the metal oxidation state and particle agglomeration. In addition, theoretical calculations and SERS data support an OER-promoting influence of the SnO_2 support on electrochemical interface properties of the catalyst.

Although the idea of stabilizing the catalytic phase by adding a stable rutile structure seems to be a good approach, the manuscript is currently in a very raw state. The results are partly not well described, and some crucial information is missing in the text, which is also not well structured (e.g. sample names are used without explanation in the main text, the sources are interrupted by other content). Moreover, some of the data analyses need to be revised or explained in more detail. For some of the conclusions the authors draw, additional measurements may be necessary. Overall, my suggestion is that the manuscript can only be published after major revisions addressing at least the following aspects:

Response: Thank you very much for your positive comments and constructive suggestions. We appreciate the reviewer's time and efforts in reviewing the manuscript. We have supplemented several experiments and revised the manuscript according to reviewers' suggestions, and we hope the revisions have addressed your valuable comments. Please find our point-by-point responses below.

1. In the introduction, I missed a short summary of previous work on $IrCoO_x$, e.g. https://doi.org/10.1016/j.apcatb.2020.118820 and others, and a discussion how previous results are correlated to the new insights.

Response: Many thanks for your constructive comment. We have carefully read the recommended literature and related works. Accordingly, we have added a short summary and discussion in the Introduction of the revised manuscript, explicitly correlating previous findings with the new insights gained from our study. The added text reads as follows: "Generally, introducing a secondary metal such as Co or Ni is a feasible approach to achieve both the amorphization of IrO_x-based catalysts and reduced usage of Ir[19, 20]." (Page 3, Line 16-18) Besides, we also appropriately cited the relevant papers in the revised manuscript with Reference (19, 20). We believe this addition provides the necessary context and highlights the advancement of our work over previous studies.

2. Also, using SnO_2 for stabilizing Ir-based catalysts is not a completely new approach. The authors should include a brief overview of results presented in the literature.

Response: Thanks for your comment. We agree that using SnO_2 to stabilize Ir-based catalysts has been previously explored. To provide appropriate context and acknowledge prior work, we have added a brief overview in the revised manuscript, which reads as follows: "Tin oxide (SnO_2), a typical rutile-type oxide, has recently been recognized as a suitable corrosion-stable substrate material under PEM conditions[30,31]." (Page 3, Line 29-Page 4, Line 2) The relevant references (30, 31) have been cited accordingly. This addition clarifies how our work builds upon existing knowledge while highlighting the specific advancements.

3. How did the authors derive the space group of the paracrystalline $IrCoO_x$ structure?

Response: Thanks a lot for your question. The space group assignment of the paracrystalline $IrCoO_x$ structure was derived based on a combination of XRD patterns and *in-situ* heating SAED images.

First, unlike the featureless XRD peaks of amorphous phase, the paracrystalline $IrCoO_x$ samples display broadened but distinctive diffraction peaks. The as-prepared $IrCoO_x$ and commercial IrO_2 all show their highest XRD peaks at nearly identical

positions at ~35°, suggesting the same space group for paracrystalline $IrCoO_x$ and IrO_2.

Second, *in-situ* heating SAED images reveal that an amorphous diffuse halo at 300 °C transforms into paracrystalline diffraction rings at 360 °C. The observed diffraction pattern is almost identical to that of commercial IrO_2, which has a space group of $P4_2/mnm$. Therefore, we can reasonably derive that the space group of paracrystalline $IrCoO_x$ structure is also $P4_2/mnm$.

4. As mentioned, the sample names appear out of the blue in the main text. I would suggest including one sentence here, which describes the main variation between $IrCoO_x$-1, $IrCoO_x$-2 and $IrCoO_x$-3, and a hint to look into the materials section for more details.

Response: Thank you very much for your suggestion. We have supplemented the sample description in the revised manuscript, which is as follows: "The degrees of structural disorder for $IrCoO_x$-1, $IrCoO_x$-2 and $IrCoO_x$-3 could be controllably regulated by adjusting the relative amounts of $IrCl_3$ and $CoCl_2·6H_2O$ (see more experimental details in the Methods section)." (Page 6, Line 8-10)

5. In the paragraph in which the authors mention the modified one-step molten salt method in the main text for the first time, they should include a hint that the method is described in detail in the material section.

Response: Thanks for your suggestion. We have added the hint in the revised manuscript, and the related texts read: "(the method is described in detail in the Methods section)" (Page 6, Line 6)

6. The authors should briefly explain how they quantify the "amorphous degree" from XRD.

Response: Thanks for your question. We agree that XRD alone cannot quantitatively determine the amorphous degree. In our work, the broader half-peak width observed in XRD patterns (**Fig. 1a**) serves only as qualitative evidence of increased amorphous

character. To enable quantitative assessment, we introduced the concept of the amorphous factor (AF) based on XPS analysis. The calculated AF values increases progressively with Co doping (0.3→0.37→0.42), confirming that Co incorporation promotes the formation of the paracrystalline phase.

7. In the sentence "The calculated Ir^{4+}/Ir^{0} ratio in X-ray photoelectron spectroscopy (XPS, Table S1) decreases progressively with Co incorporation (Figs. 1f and S5)[32,33], a modulation that is anticipated to enhance the catalyst's operational stability during OER.", do the given sources explain why the catalyst stability is enhanced by the observed modulation? In this case, the should be placed to the end of the sentence, otherwise the authors should prove the statement by other sources.

Response: Thanks a lot for your careful review. High-valent Ir exhibits stronger oxidizing properties and is more prone to further oxidation or chemical dissolution under acidic, high-potential OER conditions. We have supplemented the new references (*J. Mater. Chem. A* **2021**, 9, 2980-2990; *Adv. Funct. Mater.* **2020**, 30, 2003935) to support the statement. These studies indicate that a higher proportion of metallic Ir (Ir^{0}) helps maintain structural integrity, while an appropriate amount of Ir^{4+} ensures catalytic activity. The progressive decrease in the Ir^{4+}/Ir^{0} ratio with Co incorporation leads to a mixed-valence state that balances activity and stability, thereby enhancing the catalyst's operational durability during OER.

8. The authors should explain how they attribute the different components they used to fit the O1s XPS spectra to the respective species. Moreover, especially the term "metal oxygen" for the component in XPS fitting seems to be a bit misleading, as metallic usually doesn't contain oxygen. I would recommend using a more comprehensible term like e.g., following the authors' explanation in the text, "disordered oxygen".

Response: Thank you very much for your careful evaluation. We attribute the O 1s XPS spectra to lattice oxygen (O_L) and metal oxygen (O_M) based on the guidance of corresponding reference (Ram *et al.*, *Science* **2024**, 384, 1373-1380), among which O_M

represents oxygen atoms directly bonded to metal ions in a disorder configuration. Besides, I highly acknowledge your recommendation that the concept of "metal oxygen" in this article is equivalent to that of "disordered oxygen".

9. In the paragraph "Evolution mechanism for paracrystalline structure" the authors mention the use of KOH, but the subsequent chemical equations describe reactions involving NaOH instead. Please clarify which substance was used.

Response: Thank you very much for your careful review. We apologize for our negligence and have now corrected the text by replacing "KOH" with "NaOH" in the revised manuscript. (Page 8, Line 15/16/22)

10. Regarding the sentence "Whereas the IrO_x sample is highly amorphous (Figs. S9-S11), the incorporation of Co induces a slight increase in structural order (i.e., paracrystalline), which is corroborated by a discernible right-shift in the XRD peaks.": What is a right-shift? Usually shifts of Bragg-reflections in XRD indicate lattice parameter changes. The authors should rather discuss the intensity differences and broadenings.

Response: Thanks for your professional question and suggestion. We agree that describing a "right-shift" in XRD peaks solely by angular position is insufficient, as changes in lattice parameters and crystallinity should be distinguished. The term "right-shift" refers specifically to an increase in the Bragg angle (2θ), which corresponds to a decrease in interplanar spacing according to Bragg's law. Accordingly, we have revised the sentence as follows: "Whereas the IrO_x sample is highly amorphous (Figs. S9-S11), the incorporation of Co induces a slight increase in structural ordering level (i.e., paracrystallinity), as evidenced by the appearance of more featured XRD peaks. Meanwhile, the diffraction peaks shift to higher angles due to the lattice contraction due to Co incorporation, and the peak broadening reflects the retained paracrystalline nature." (Page 8, Line 24-28) We believe that this revised description more accurately conveys the structural changes induced by Co incorporation.

11. Why did the authors decide to use IrO_2 as a reference for OER activity, which is a rather poor catalyst? State of the art electrolyzers usually use metallic Ir particles, which form a hydrous active surface. Wouldn't including this IrO_x type catalyst as a reference yield a fairer comparison?

Response: Thanks very much for your questions. We selected commercial IrO_2 as a reference precisely because of its excellent and representative OER performance in acidic media, enabling fair comparison with numerous existing studies. Under the identical test conditions, the as-prepared $IrCoO_x$-2@SnO_2 catalyst shown even better OER activity than commercial IrO_2. Besides, to address your concern, we have now added the comparison of OER performance for $IrCoO_x$ and IrO_x catalysts. As shown in **Fig. R3**, the as-prepared $IrCoO_x$-2@SnO_2 catalyst still shown better OER activity than IrO_x.

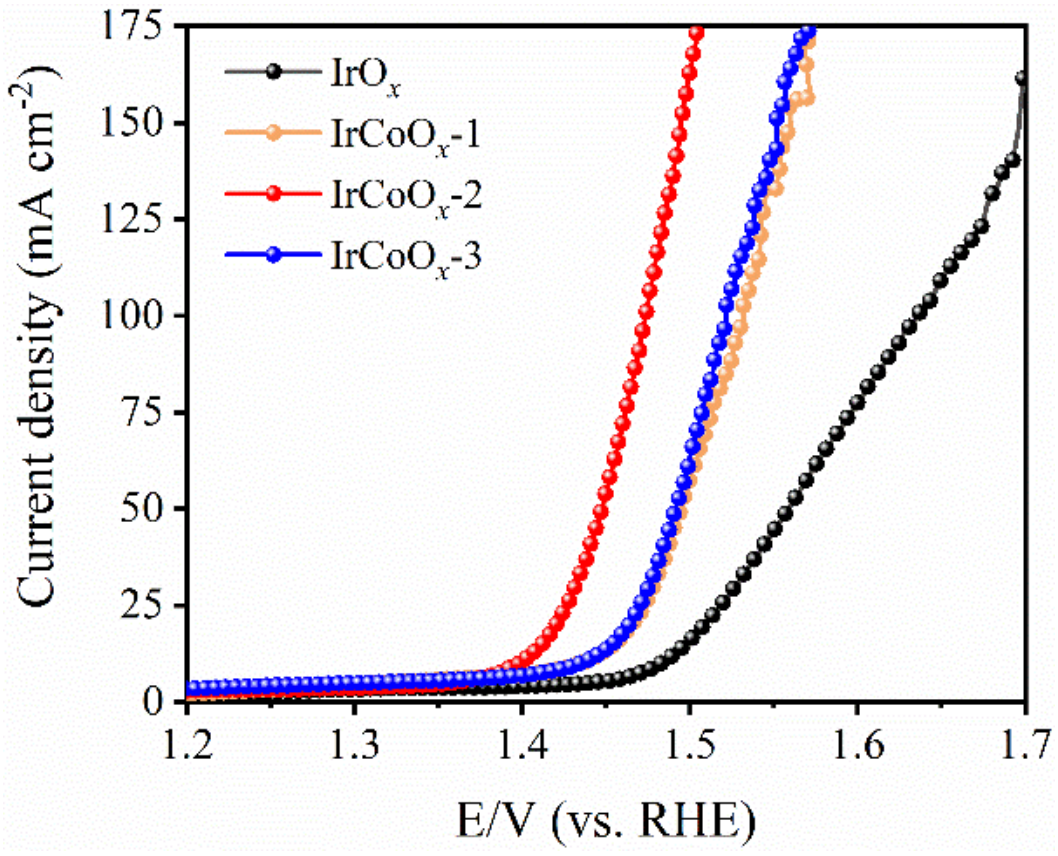

Fig. R3. LSV curves of $IrCoO_x$-1, $IrCoO_x$-2, $IrCoO_x$-3 and IrO_x catalysts in 0.5 M H_2SO_4.

12. Is a "yolk-like" morphology different from a core-shell morphology? If not, wouldn't this be a more comprehensible term?

Response: Thanks for your question. We acknowledge that the "yolk-like" morphology is not obviously different from the core-shell morphology. So, to avoid any possible misunderstanding, we have deleted all "yolk-like" descriptions and used the "core-shell" description consistently in the revised manuscript.

13. The discussion of the EXAFS results needs to be revised: The maxima of the FT EXAFS do not directly indicate average bond distances, as the main text implies, because the oscillations include not only single but multi scattering paths and contain energy-dependent phase shifts that shift peaks away from the true interatomic distances. Therefore, a model of the Ir-containing phase needs to be considered and fit to the FT-EXAFS. The model must be explained and all fit parameters including statistical errors need to be reported in the SI, e.g. in the form of a table. Given the small differences in the FT EXAFS shown in the manuscript, discussing the errors of the bond distances extracted from the fit is crucial for understanding the significance of differences.

Response: Thanks very much for your constructive suggestions. We have supplemented the EXAFS fitting analysis of Ir foil, IrO_2, $IrCoO_x$-2, $IrCoO_x$-2@SnO_2, $IrCoO_x$-2-after and $IrCoO_x$-2@SnO_2-after samples. The fitted plots are displayed in **Fig. S42** (see below) and all the detailed fit parameters are included in **Table S8** (see below) in the Supporting Information. As shown in Table S8, the fitted results are very similar to the inset of **Fig. 4f** and the changing trends are consistent with our previous analysis, among which the Ir-O bond of $IrCoO_x$-2 catalyst shifts to larger value after OER while $IrCoO_x$-2@SnO_2 does not really change. Besides, we have added the corresponding description in the revised manuscript, which reads: "which is further supported by the fitting results (Fig. S42 and Table S8)" (Page 15, Line 16-17) and "Data reduction, data analysis, and EXAFS fitting were performed and analyzed with the Athena and Artemis programs of the Demeter data analysis packages, which utilizes the FEFF6 program for EXAFS fitting. Energy calibration was conducted through standard and Ir foil, which as a reference was simultaneously measured. A linear function was subtracted from the pre-edge region, then the edge jump was normalized using Athena software. The $\chi(k)$ data were isolated by subtracting a smooth, third-order polynomial approximating the absorption background of an isolated atom. The k^3-weighted $\chi(k)$ data were Fourier transformed after applying a HanFeng window function (Δk = 1.0). For EXAFS modeling, the global amplitude EXAFS (CN, R, σ^2 and ΔE_0) were obtained by nonlinear least-squares refinement of the EXAFS equation to the Fourier-transformed

data in R-space using Artemis software. The amplitude reduction factor (S_0^2) was determined to be 0.855 from the EXAFS fit of IrO_2 and was subsequently fixed in the analysis to determine the coordination numbers (CNs) in the sample." (Page 31, Line 3-16)

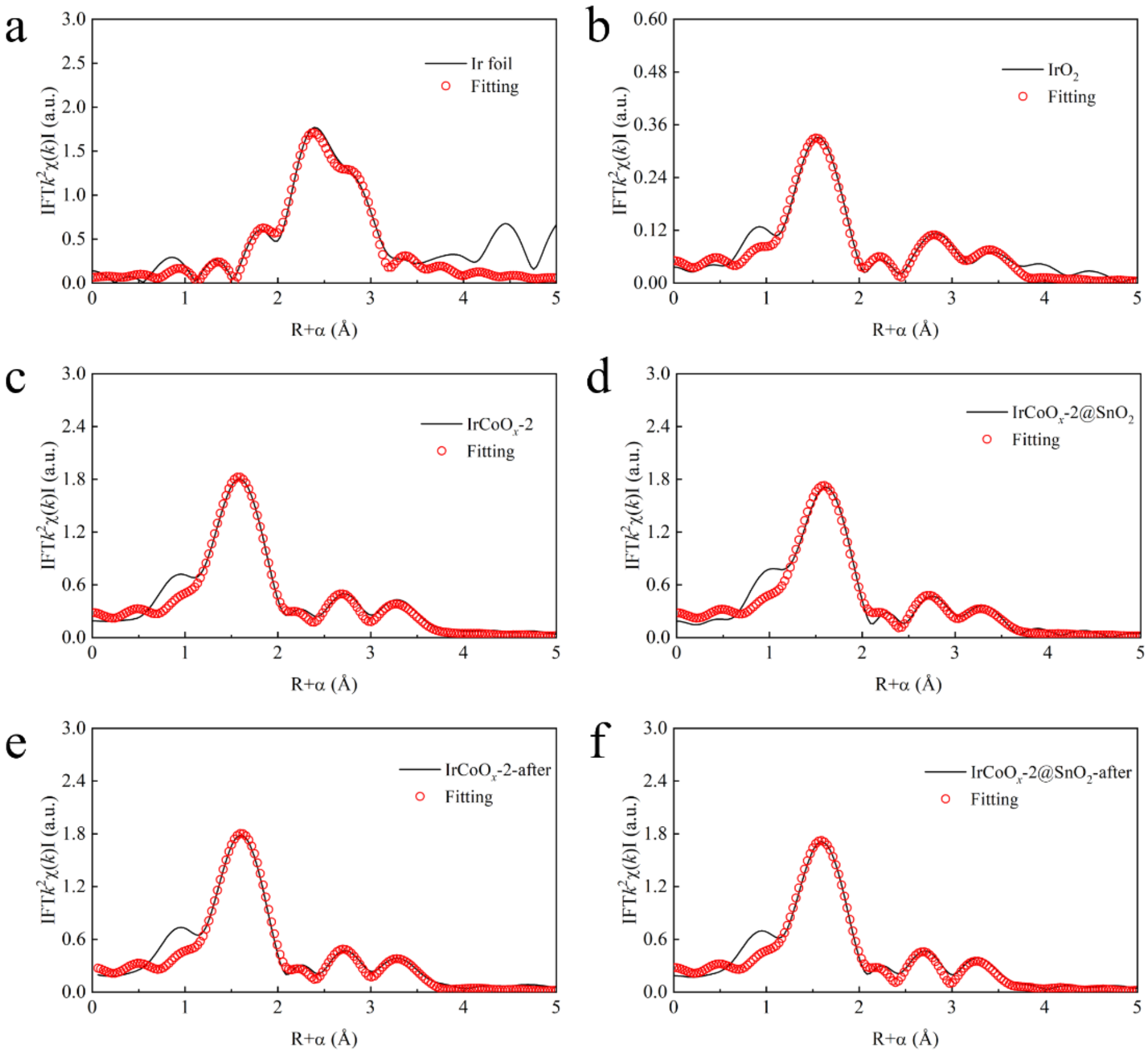

Fig. S42. EXAFS fitting results of (a) Ir foil, (b) commercial IrO_2, (c) $IrCoO_x$-2, (d) $IrCoO_x$-2@SnO_2, (e) $IrCoO_x$-2@SnO_2 and (f) $IrCoO_x$-2@SnO_2-after catalysts.

Table S8. EXAFS fitting parameters at the Ir L^3-edge for various samples.

Sample	Shell	CN[a]	R(Å)[b]	σ^2(Å^2)[c]	ΔE_0(eV)[d]	R factor
Ir foil	Ir-Ir	12*	2.70±0.01	0.0030	6.9	0.0073
IrO_2	Ir-O	6.0*	1.97±0.01	0.0028	9.3	0.0145
	Ir-Ir	6.0*	3.19±0.01	0.0042	9.3	
	Ir-Ir	6.0*	3.57±0.01	0.0192	4.0	
$IrCoO_x$-2	Ir-O	5.9±0.3	1.99±0.01	0.0060	13.0	0.0158
	Ir-Co	4.8±1.0	3.07±0.01	0.0187	8.4	
	Ir-Co	5.8±1.2	3.70±0.01	0.0140	-2.0	
$IrCoO_x$-	Ir-O	5.7±0.3	2.00±0.01	0.0059	12.4	0.0173

2@SnO_2	Ir-Co	3.9±0.8	3.10±0.01	0.0160	8.8	
	Ir-Co	5.8±1.2	3.70±0.01	0.0152	-2.5	
$IrCoO_x$-2-after	Ir-O	5.5±0.3	2.02±0.01	0.0048	13.2	0.0137
	Ir-Co	4.2±0.8	3.08±0.01	0.0169	7.1	
	Ir-Co	7.1±1.4	3.72±0.01	0.0166	-3.4	
$IrCoO_x$-2@SnO_2-after	Ir-O	5.5±0.3	2.02±0.01	0.0054	12.1	0.0156
	Ir-Co	2.5±0.5	3.08±0.01	0.0123	5.1	
	Ir-Co	3.4±0.7	3.72±0.01	0.0100	-3.2	

Note: (a) CN, coordination number; (b) R, distance between absorber and backscatter atoms; (c) σ^2, Debye-Waller factor to account for both thermal and structural disorders; (d) ΔE(0), inner potential correction; R factor indicates the goodness of the fit. S_0^2 was fixed to 0.855, according to the experimental EXAFS fit of Ir foil by fixing CN as the known crystallographic value. A reasonable range of EXAFS fitting parameters: $0.700 < S_0^2 < 1.000$; CN > 0; $\sigma^2 Å^2 > 0$; $|\Delta E_0| < 15$ eV; R factor < 0.02.

14. What additional information do the Wavelet transform graphs provide, which look very similar for both samples?

Response: Thanks a lot for your question. Although the wavelet transform patterns in **Fig. 4g-4j** appear similar at first glance, a closer comparison reveals subtle but important difference. Notably, the R value of $IrCoO_x$-2-after slightly increases compared to $IrCoO_x$-2 (the central core of $IrCoO_x$-2-after in **Fig. 4h** is above the white baseline). Correspondingly, there is hardly any alteration for $IrCoO_x$-2@SnO_2 after the stability test, providing definitive evidence for the stabilizing function of SnO_2.

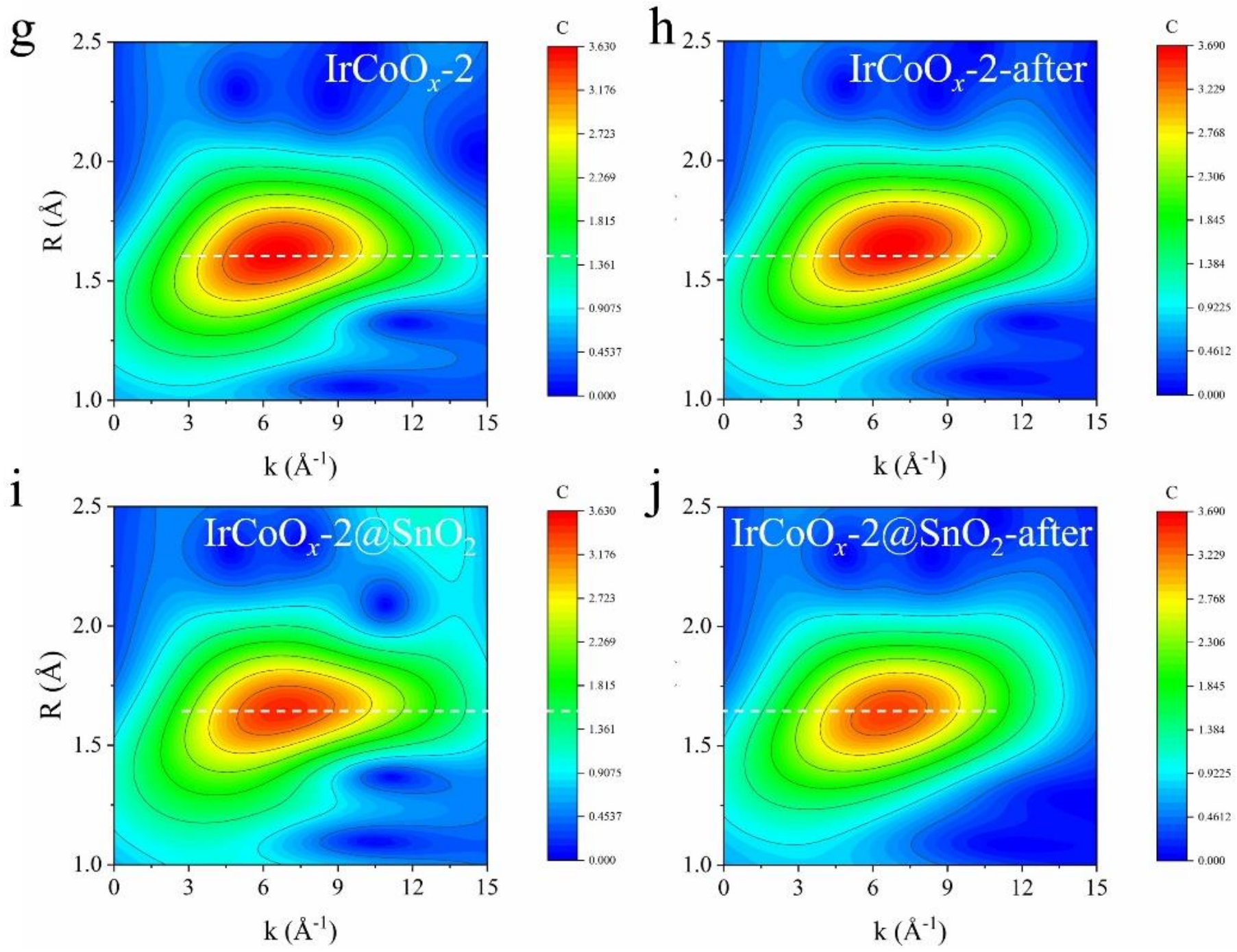

Fig. 4 The corresponding wavelet transform (WT) patterns for (g) IrCoO$_x$-2, (h) IrCoO$_x$-2-after, (i) IrCoO$_x$-2@SnO$_2$ and (j) IrCoO$_x$-2@SnO$_2$-after.

15. Why did the authors only study the Ir L-edge? Combination with Co K-edge EXAFS could yield information about the Co-M and Ir-M distances, which might support the proposed substitutional paracrystalline lattice structure, in which Co and Ir occupy equivalent sites.

Response: Thank you very much for your professional suggestion. In response, we have supplemented the Co K-edge EXAFS analysis in the revised manuscript. As shown in **Fig. S41** (see below), the larger R value of IrCoO$_x$-2 at ~2.5 Å compared to Co_3O_4 confirms the presence of Co-O-Ir signal. Therefore, the Co K-edge EXAFS result support the proposed substitutional lattice structure, in which Co and Ir occupy equivalent sites. Besides, we have added the corresponding description in the revised manuscript, which reads: "Moreover, the presence of Co-O-Ir signal in Fig. S41 also supports the substitution of Co into the IrO$_x$ lattice." (Page 15, Line 12-13)

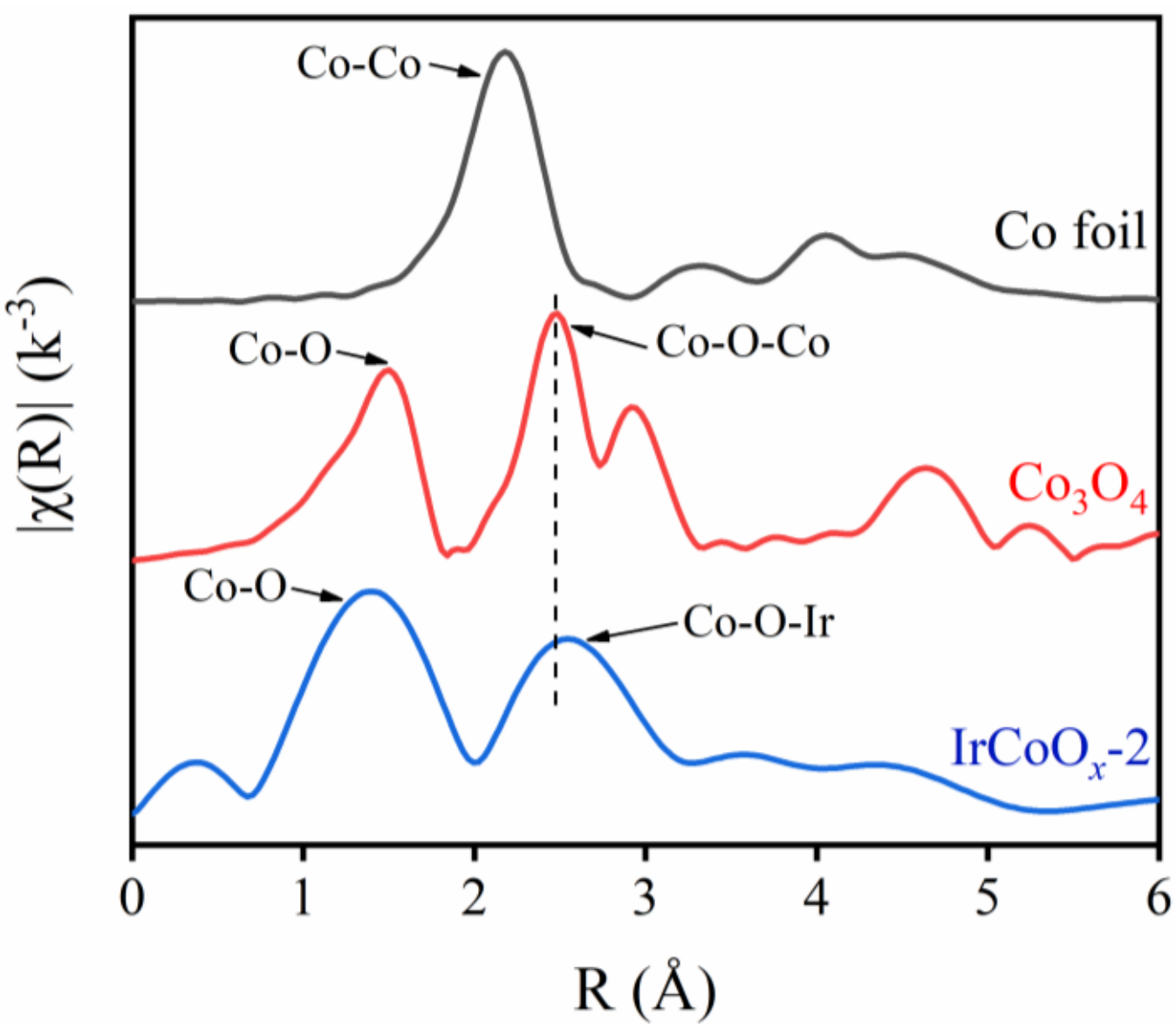

Fig. S41. The comparison of EXAFS spectra at Co K-edge among Co foil, Co_3O_4 and $IrCoO_x$-2.

16. How do the authors derive the conclusion that SnO_2 incorporation prevents overoxidation? Do they have evidence for different oxidation states from XANES or from fitting the Ir 4f XPS?

Response: Thank you for your questions. Our conclusion that SnO_2 incorporation prevents overoxidation is mainly supported by the XPS Ir 4f analysis. As shown in XPS Ir 4f spectra (**Fig. S34**), the post-OER $IrCoO_x$-2 sample exhibits a positive binding energy shift of 0.37 eV, indicating an undesired increase of Ir valence, which is usually considered as the critical deactivation reason for Ir-based catalysts. In stark contrast, $IrCoO_x$-2@SnO_2 exhibits a much smaller Ir 4f shift (0.17 eV, **Fig. S35**), consolidating the role of SnO_2 in enhancing stability by suppressing the oxidation-induced valence increase of Ir. We have also clarified this point in the revised manuscript, which reads: "XPS Ir 4f spectra (Fig. S34) reveals a positive binding energy shift by 0.37 eV for the post-OER $IrCoO_x$-2, proving the undesired increase of Ir valence, which is usually considered as the critical deactivation indicator for Ir-based catalysts. In stark contrast, $IrCoO_x$-2@SnO_2 exhibits a much smaller Ir 4f shift (0.17 eV, Fig. S35). The same trend is also found in XPS Co 2p spectra (2.25 eV vs. 1.55 eV) in Figs. S36-S37,

consolidating the role of SnO_2 for stability enhancement by inhibiting the valence increase." (Page 13, Line 8-14)

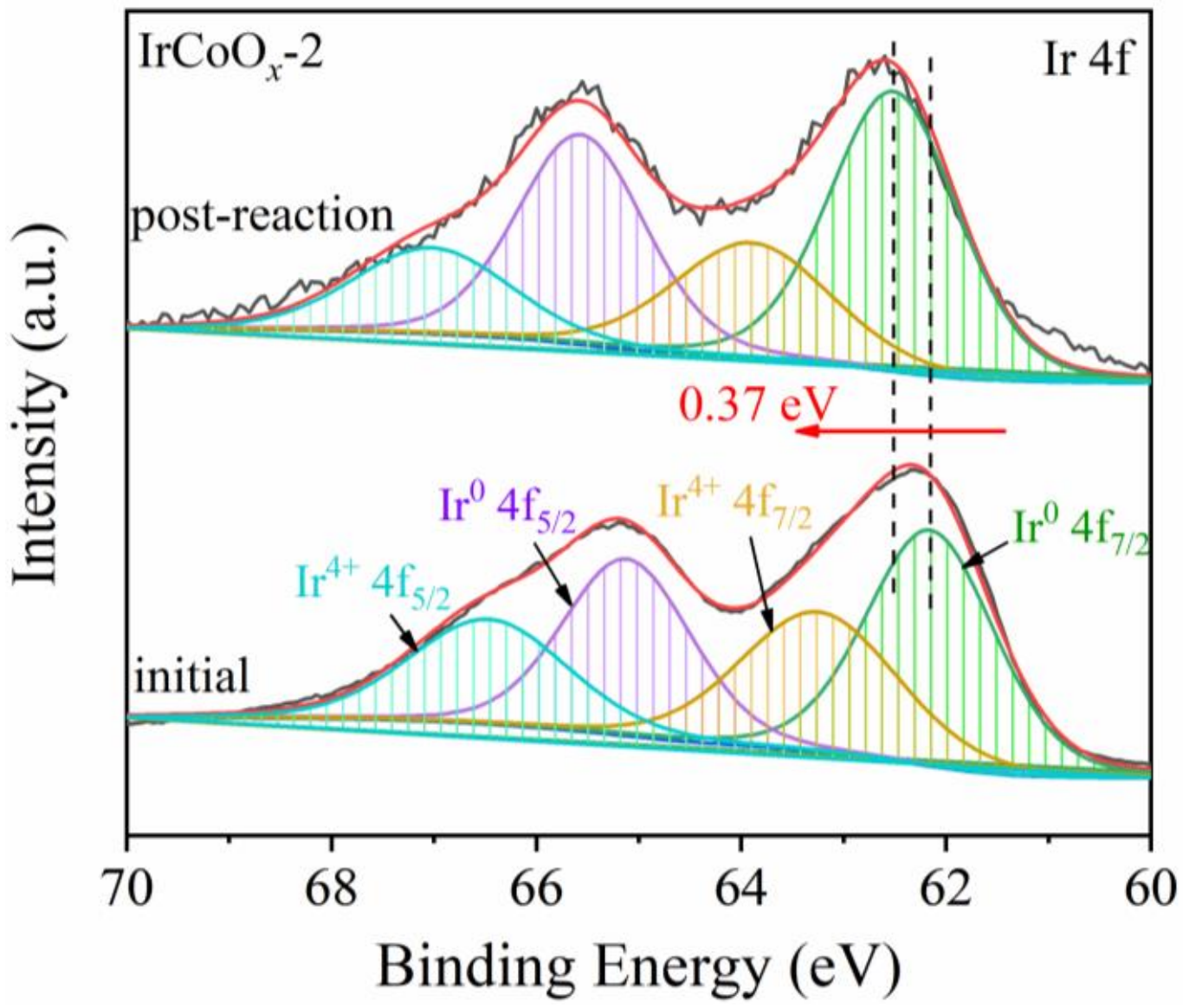

Fig. S34. The comparison of XPS Ir 4f spectra before and after OER reaction for $IrCoO_x$-2.

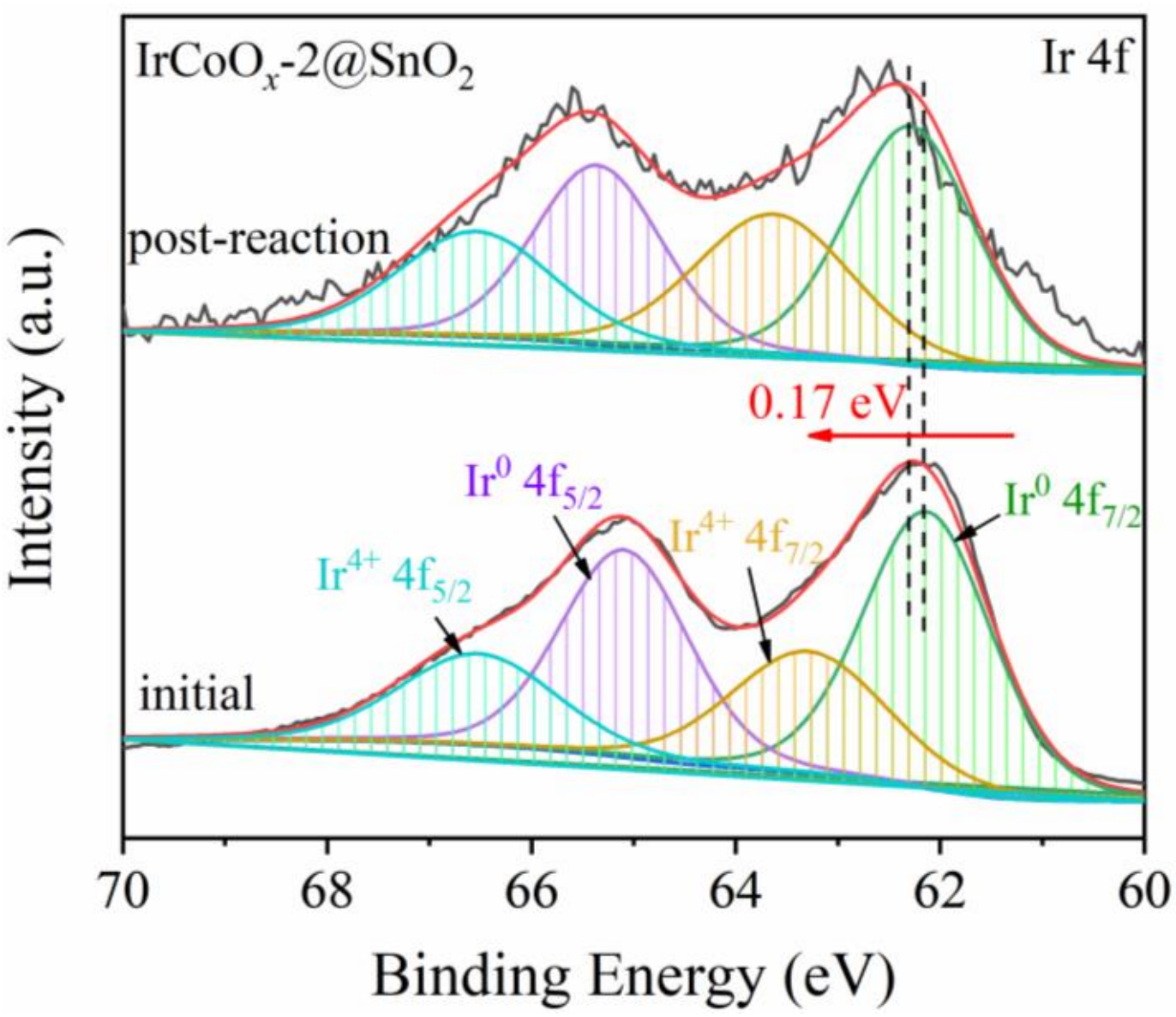

Fig. S35. The comparison of XPS Ir 4f spectra before and after OER reaction for $IrCoO_x$-2@SnO_2.

17. When stating the pH-dependent slopes in the text, please don't omit the units.

Response: Thanks a lot for your constructive suggestion. We have added the units for the pH-dependent slope values in the revised manuscript. Since pH has no units, the

units of the slope remain mA cm^{-2}.

18. How can the authors make sure that a different pH dependent slopes are unambiguously connected with the AEM and LOM? Even if it is based on previous results from literature, these should be mentioned and their transferability to the material investigated in this work should be discussed.

Response: Thanks for your careful questions. As you mentioned, we derived the conclusion of AEM or LOM mechanism based on previous results from literature (*Adv. Mater.* **2024**, 36, 2401648; *Nat. Commun.* **2023**, 14, 5119). We apology for our oversight and have added the corresponding description in the revised manuscript, which reads: "Largely differing from AEM prevalent in commercial IrO_2, the LOM involves non-proton coupled electron transfer (non-PCET) steps, rendering it highly sensitive to pH variations." (Page 16, Line 14-16)

19. The authors should explain why a lower pH-slope is connected with a lower *OH coverage.

Response: Thank you very much for your insightful question. In the revised manuscript, we have clarified the connection between a lower pH-slope and lower *OH coverage based on established literature (*J. Am. Chem. Soc.* **2024**, 146, 35438-35448; *Small* **2024**, 20, 1401964). Specifically, methanol is used as a molecular probe to assess the *OH surface coverage, as the methanol oxidation reaction (MOR) proceeds *via* nucleophilic attack of methanol molecules on electrophilic *OH species. Consequently, a catalyst that is less active toward MOR (indicated by a lower pH-slope) reflects lower *OH coverage on its surface. Thus, a lower pH-slope indicates that the as-prepared $IrCoO_x$-2@SnO_2 catalyst is more inert to MOR, suggesting a lower *OH coverage. We have supplemented the corresponding description in the revised manuscript, which reads: "Moreover, the over-oxidation of Ir ($Ir^{IV}O_2 \rightarrow Ir^{V}O_2$-$OH \rightarrow Ir^{VI}O_3$) to soluble high-valence [IrO_3] species is known as the main deactivation cause of commercial IrO_2. Therefore, the undesired electrophilic group-OH* should be rigorously monitored

during OER, which can be quantified by adding nucleophilic reagent-methanol (Figs. S46-S48)." (Page 16, Line 19-23)

20. The presented data represent rather indirect indications for the deduced mechanisms. In the current version, these mechanisms are not based on convincing evidence and so they can only represent a speculative interpretation and should also be described and discussed as such!

Response: Thank you very much for your rigorous assessment. We agree that indirect evidence is insufficient to conclusively establish the reaction mechanism. In response, we have now supplemented the O^{18} isotope labeling experiment coupled with differential electrochemical mass spectrometry (DEMS) to provide direct evidence.

Firstly, we perform O^{18} labeling by immersing the working electrode in 0.5 M H_2SO_4 prepared with H_2O^{18} and multiple LSV scans between 1.1-1.5 V (vs. RHE). Then, after rinsed 10 times with H_2O^{16}, the same electrochemical procedure was repeated in 0.5 M H_2SO_4 prepared with H_2O^{16}, while recording the gaseous products and their isotopic composition in real time by mass spectrometry. As shown in **Fig. S52** (see below), the presence of $O^{18}O^{18}$ and $O^{16}O^{18}$ peaks confirm the exchange of O^{18} into the lattice of $IrCoO_x$-2@SnO_2, thus consolidating the LOM mechanism for as-prepared $IrCoO_x$-2@SnO_2 catalyst.

In the revised version, we have added the corresponding description in the relevant sections of the manuscript, and the related texts read: "To clearly elucidate the OER pathway of as-prepared $IrCoO_x$-2@SnO_2, O^{18} isotope-labeling differential electrochemical mass spectrometry (DEMS) was employed. Firstly, the working electrode was immersed in 0.5 M H_2SO_4 prepared with H_2O^{18} and then multiple LSV scans were made between 1.1-1.5 V (vs. RHE). After rinsed 10 times with H_2O^{16}, the same electrochemical procedure was repeated in 0.5 M H_2SO_4 prepared with H_2O^{16}, while recording the gaseous products and their isotopic composition in real time by mass spectrometry. As shown in Fig. S52, the presences of $O^{18}O^{18}$ and $O^{16}O^{18}$ peaks confirm the exchange of O^{18} into the lattice of $IrCoO_x$-2@SnO_2, thus consolidating the

LOM mechanism for as-prepared $IrCoO_x$-2@SnO_2 catalyst." (Page 18, Line 17-25)

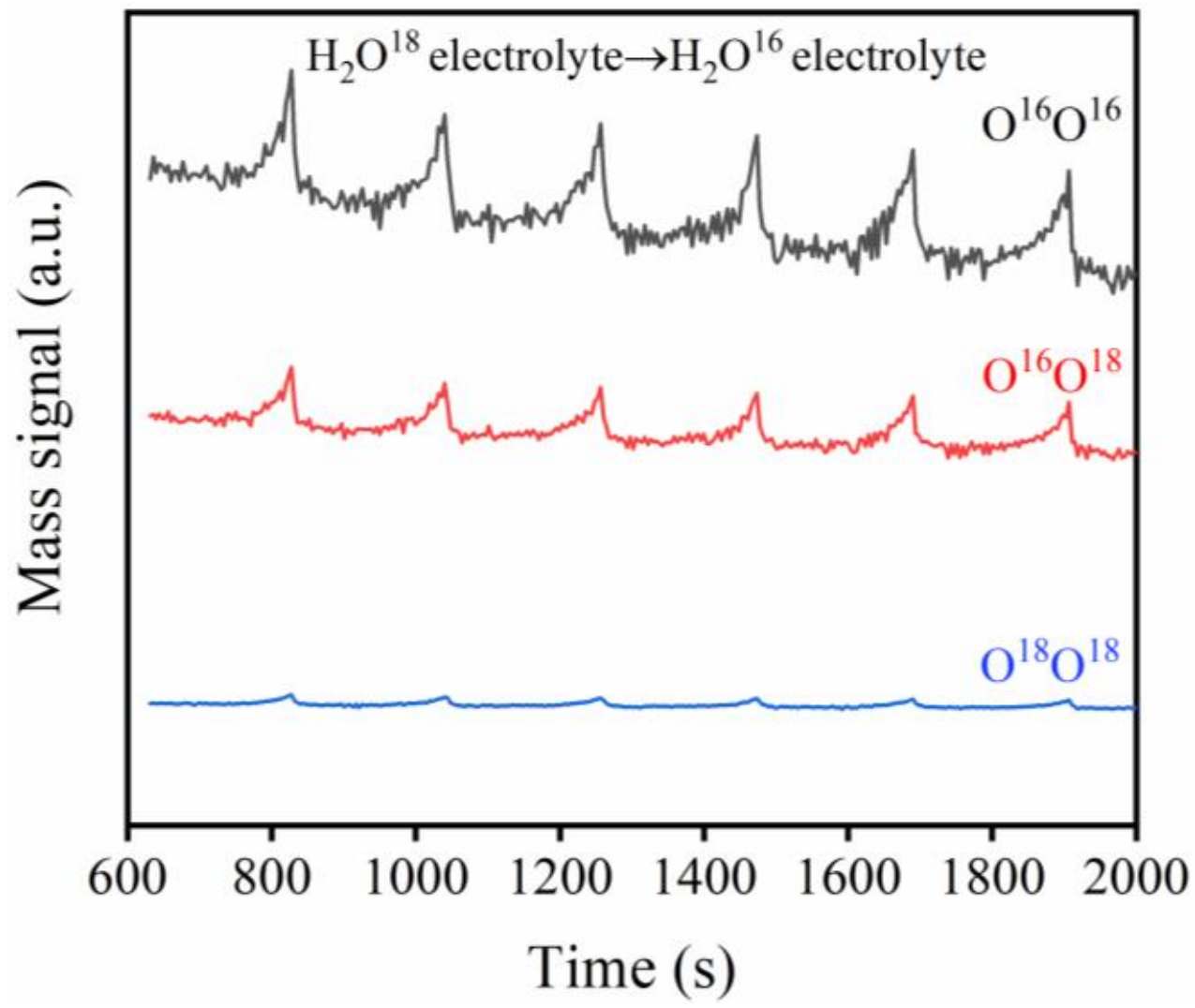

Fig. S52. DEMS signals of different O_2 products for O^{18} labeled $IrCoO_x$-2@SnO_2 in 0.5 M H_2SO_4 using H_2O^{16} as electrolyte.

21. What do the authors mean by the term "oxygen species cycling"? This should be defined in the text.

Response: Thanks a lot for your helpful suggestion. In the revised manuscript, we have clearly defined the term "oxygen species cycling". Specifically, for LOM-compliant catalysts such as $IrCoO_x$-2, the consumed lattice oxygen cannot usually be efficiently replenished (see Step iv in **Fig. R4**, see below), leading to irreversible structural damage and oxidation of Ir sites. Upon the introduction of SnO_2, the increase abundance of free water at the interface can supply necessary reactants at Step iv (O-M-◌ + H_2O → H^+ + e^- + O-M-OH) and sustain a reversible oxygen cycle during OER. Accordingly, we defined the "oxygen species cycling" as the regenerative process: H_2O→lattice oxygen→O_2. This definition has been added to the revised manuscript (Page 5, Line 5-6)

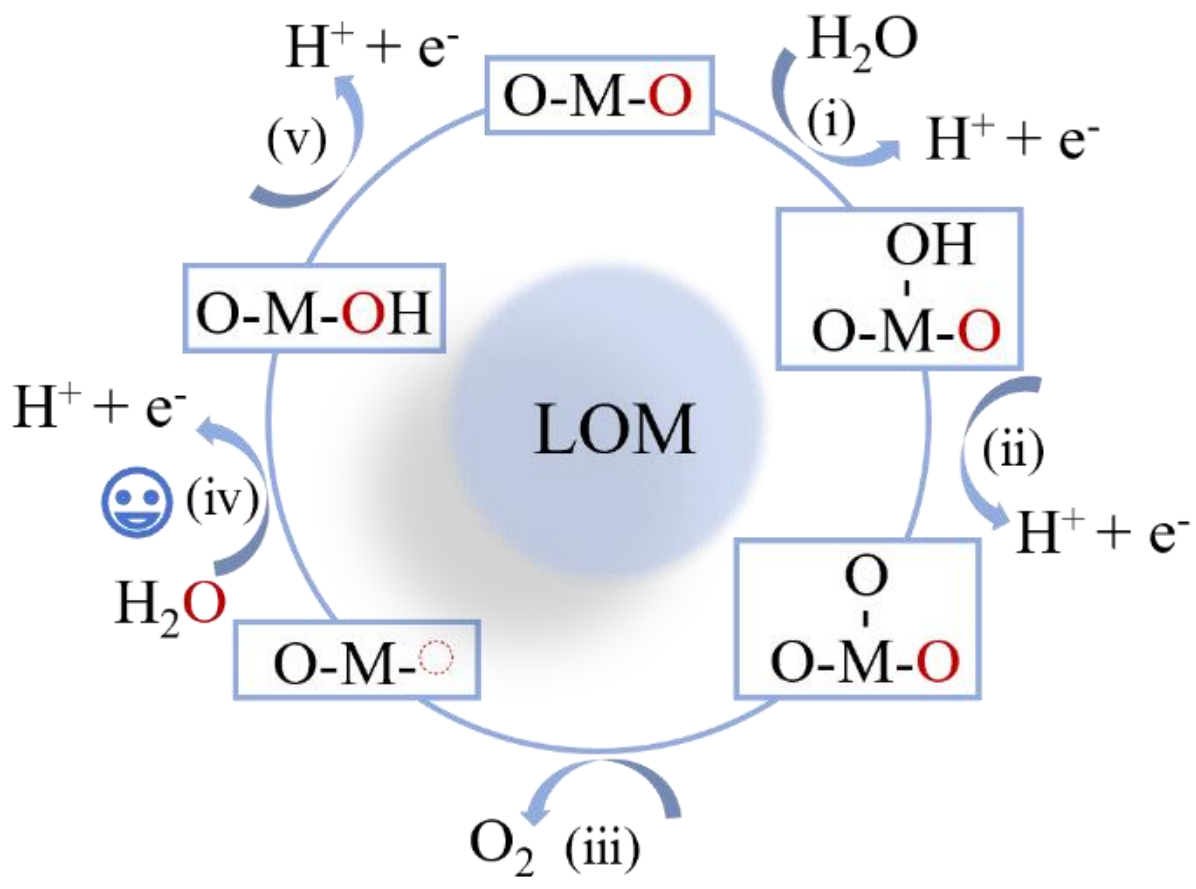

Fig. R4. The schematic illustration of reaction pathway with the reversible oxygen species cycling.

Point-to-point response and revisions

(Original Manuscript Number: NCOMMS-25-101304A)

Note: In the response manuscript, comments suggested by reviewers are in **black font**, our responses are in blue font, and revised parts in the text and SI are highlighted in yellow. The figures in our Manuscript, Supplementary Information and Response documents are displayed in the form of **Fig. XX**, **Fig. SXX** and **Fig. RXX**, respectively.

Responses to Reviewer 2

Comments: Regarding the third question raised by Reviewer 1, the authors conducted a set of control experiments with the commercial PtRu/C as the cathode material. However, this material operated stably for only 40 hours, which contrasts sharply with the stability of Pt/C as the cathode material (approximately 4,000 hours, as shown in Figure 3g). Although both are platinum-based materials, the difference in stability amounts to two orders of magnitude. What accounts for this significant discrepancy? Detailed discussion should be provided in the response. Furthermore, for the long-term stability test lasting 4,000 hours originally presented in Figure 3g, the original V-t curve must be supplied in the Supporting Information, rather than only a selection of data points.

This manuscript has been well revised with added experimental data and discussion, and it is acceptable for publication.

Response: Thank you very much for your positive recommendation. We appreciate the reviewer's time and efforts in reviewing the manuscript. We have supplemented the response and revised the manuscript according to reviewers' suggestions, and we hope the revisions have addressed your valuable comments. Please find our point-by-point responses below.

(1) Regarding your concern about the performance of the commercial PtRu/C cathode, we apologize for the unclear wording in our previous response. We would like to clarify that the stability of the PtRu/C cathode is not inferior to that of the Pt/C cathode. The

reason we only conducted a 40-hour stability test is that the PtRu/C cathode exhibits significantly poor activity (~1.9 V at 1 A cm^{-2}) under the same loading of noble metal, which made longer-term testing unnecessary. In other words, it was the poor activity, not poor stability, that limited the test duration. Furthermore, as our industry partners have informed us, even advanced Pt-based catalysts have not yet been deployed industrially. Pt/C still remains the benchmark catalyst for practical applications. Therefore, we did not perform control experiments lasting several hundred or thousand hours.

(2) Regarding your concern about the original V-t curve, we sincerely appreciate your suggestion. In the revised supporting information, we have added the original V-t curve for about 4000 h (**Fig. S23**, see below).

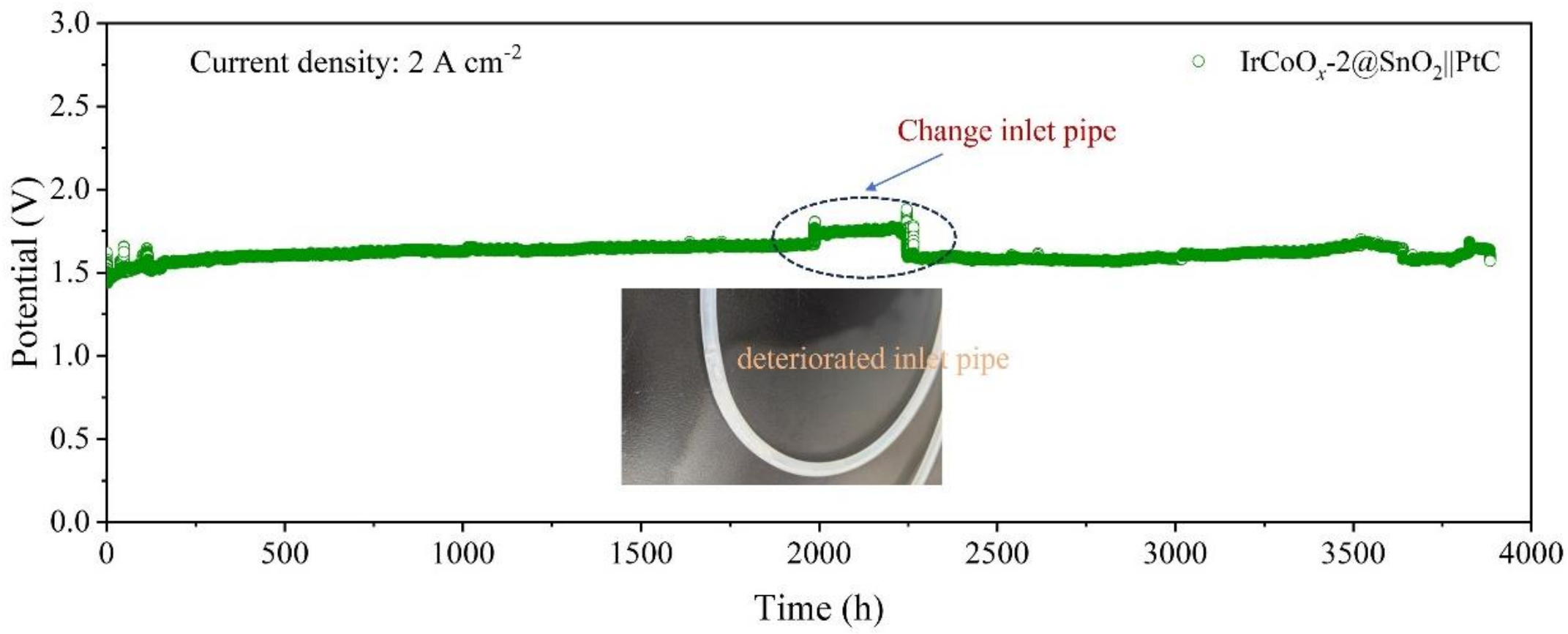

Fig. S23. The original V-t curve at 2.0 A cm^{-2} in a PEMWE electrolyzer using $IrCoO_x$-2@SnO_2 and commercial Pt/C as anode and cathode catalysts (Inset: The photograph for the deterioration of inlet pipe).

Responses to Reviewer 3

Comments: In the updated version, the authors put some effort into addressing the remarks, which was partly successful. I still highly recommend the following remaining aspects to be considered prior to publication:

Response: Thank you very much for your positive comments and constructive suggestions. We appreciate the reviewer's time and efforts in reviewing the manuscript. We have supplemented EXAFS fitting of Co K-edge spectra and revised the manuscript especially about the analysis of XAS results according to reviewer's suggestions, and we hope the revisions have addressed your valuable comments. Please find our point-by-point responses below.

1. Regarding 1 and 2: It is good to see that at least the previous studies are mentioned now, but for a journal of this rank I would recommend adding a brief overview of their outcome and how the authors' work is building up on that, i.e. which additional questions they address, which were left open so far.

Response: Thank you very much for your suggestion. We have carefully read the related literature. Accordingly, we have added a brief overview in the Introduction of the revised manuscript, explicitly clarifying the correlation and difference between previous findings and our study. The added texts read as follows: "For instance, Hyung-Suk Oh et al. developed carbon-supported $IrCoO_x$ core-shell nanoparticles exhibiting efficient OER activity and stability for 216 h in neutral tap-water. Nevertheless, amorphous materials still suffer from poor electrochemical and structure stability under acidic OER conditions.[21,22]" (Page 3, Line 18-22 in the revised manuscript) and "Bein et al. have reported that the interconnected, ultrasmall IrO_2 crystallites epitaxially growing along the isostructural SnO_2 substrate enable excellent conductivity and stability. However, the doped amorphous IrO_x-based material stably anchored in SnO_2 support in acidic OER still remains unexplored. Such a support is also expected to reasonably regulate the local reaction environment, such as interfacial water structure and proton transfer, which are critical for OER kinetics and catalyst durability,

especially under LOM operation[32,33]." (Page 4, Line 4-11 in the revised manuscript) We believe this addition provides the necessary context and highlights the advancement of our work over previous studies.

2. Regarding 3 and 8: It would be great if the authors could include the explanation into the SI to make this aspect more transparent to the readers.

Response: Thanks for your constructive suggestion. We have added the corresponding description in the revised supporting information, which reads: "The space group assignment of the paracrystalline $IrCoO_x$ structure was derived based on a combination of XRD patterns and *in-situ* heating SAED images. First, unlike the featureless XRD peaks of amorphous phase, the paracrystalline $IrCoO_x$ samples display broadened but distinctive diffraction peaks. The as-prepared $IrCoO_x$ and commercial IrO_2 all show their highest XRD peaks at nearly identical positions at ~35°, suggesting the same space group for paracrystalline $IrCoO_x$ and IrO_2. Second, *in-situ* heating SAED images reveal that an amorphous diffuse halo at 300 °C transforms into paracrystalline diffraction rings at 360 °C. The observed diffraction pattern is almost identical to that of commercial IrO_2, which has a space group of $P4_2/mnm$. Therefore, it is reasonable to derive that the space group of paracrystalline $IrCoO_x$ structure is also $P4_2/mnm$." (Page 2 in the revised SI) and "We attribute the O 1s XPS spectra to lattice oxygen (O_L) and metal oxygen (O_M) based on the guidance of corresponding reference (Ram *et al.*, *Science* **2024**, 384, 1373-1380), among which O_M represents oxygen atoms directly bonded to metal ions in a disorder configuration." (Page 8 in the revised SI)

3. Regarding 6: In this case, the following sentence in line 108 needs to be adjusted: "Notably, the amorphous degree of $IrCoO_x$ sample increases with the higher loading of Co element." It implies that the amorphous degree is derived from XRD and it must be made clear how this conclusion was derived.

Response: Thanks a lot for your professional review. We apologize for the inaccurate expression. As stated in our first revision, the broader half-peak width observed in XRD

patterns (**Fig. 1a**) serves only as qualitative evidence of increased amorphous character and is insufficient for a quantitative assessment. After careful consideration, we have decided to delete the sentence to avoid any possible misunderstanding. The quantitative evaluation of the amorphous degree has been systematically addressed in the subsequent XPS analysis.

4. Regarding 13: The following passage should be completely rewritten: "The Fourier-transformed extended X-ray absorption fine structure (EXAFS, Fig. 4e) 292 reveals a longer Ir-O bond in $IrCoO_x$-2 compared with commercial IrO_2, attributed to 293 the expanded lattice owing to Co incorporation. Moreover, the presence of Co-O-Ir 294 signal in Fig. S41 also supports the substitution of Co into the IrO_x lattice. Crucially, 295 this Ir-O bond length is preserved in $IrCoO_x$-2@SnO_2, indicating that SnO_2 does not 296 perturb the paracrystalline structure. From Fig. 4f, the Ir-O bond of $IrCoO_x$-2 catalyst 297 at ~1.6 Å shifts to larger value after OER while $IrCoO_x$-2@SnO_2 does not really change, 298 which is further supported by the fitting results (Fig. S42 and Table S8)." As already alluded to in the last revision, it contains statements which are just wrong. It is not possible to draw any conclusions about the bond length from the FT-EXAFS without fitting! Moreover, the fitting results indicate very minor differences in terms of the bond length change between the both materials (e.g. Ir-O[$IrCoO_x$-2]: 1.99+/-0.01 → 2.02+/-0.01 and Ir-O[$IrCoO_x$-2@SnO_2]: 2.00+/-0.01 → 2.02+/-0.01) The significance of these differences should be discussed in the main text.

Response: Thank you very much for your constructive suggestions. We fully agree that it is not possible to draw any conclusions about the bond length from the FT-EXAFS without fitting. Accordingly, we have carefully re-examined and completely revised the corresponding passage. The revised text reads as follows: "The Fourier-transformed extended X-ray absorption fine structure (EXAFS) and the fitting results (**Figs. 4e-4f** and S41 and Table S8) reveal a longer Ir-O bond in $IrCoO_x$-2 compared with commercial IrO_2, attributed to the expanded lattice owing to Co incorporation. Crucially, the negligible difference in Ir-O bond length (1.99±0.01 Å *vs.* 2.00±0.01 Å) between

$IrCoO_x$-2 and $IrCoO_x$-2@SnO_2 indicates that this Ir-O bond is well preserved after introducing the SnO_2 support, confirming the undisturbed paracrystalline structure in $IrCoO_x$-2@SnO_2. For the $IrCoO_x$-2 catalyst, there are obvious changes in both the coordination number (5.9±0.3 → 5.5±0.3) and bond length (1.99±0.01 → 2.02±0.01) after OER, differing from those of $IrCoO_x$-2@SnO_2. Thus, we can reasonably conclude that the introduction of SnO_2 is an effective strategy for maintaining the paracrystalline structure of $IrCoO_x$-2. Moreover, by combining the fitting results of Ir L_3-edge and Co K-edge EXAFS (Fig. S42 and Table S9), the presence of Co-Ir scattering contributions was confirmed, supporting the substitution of Co into the IrO_x lattice. Besides, the WT patterns of Ir L_3-edge EXAFS signals **(Figs. 4g-4j)** display the predominated Ir-O signals. Differing from the obvious shift of Ir-O signal for $IrCoO_x$-2 after OER, there is hardly any alteration for $IrCoO_x$-2@SnO_2 after the stability test (Figs. S43-S44), providing definitive evidence for the stabilizing function of SnO_2 at the atomic level." (Page 15, Line 9-26 in the revised manuscript)

Besides, we have replaced **Figs. 4e-4f** with the new **versions** (see below) to avoid any potential ambiguity.

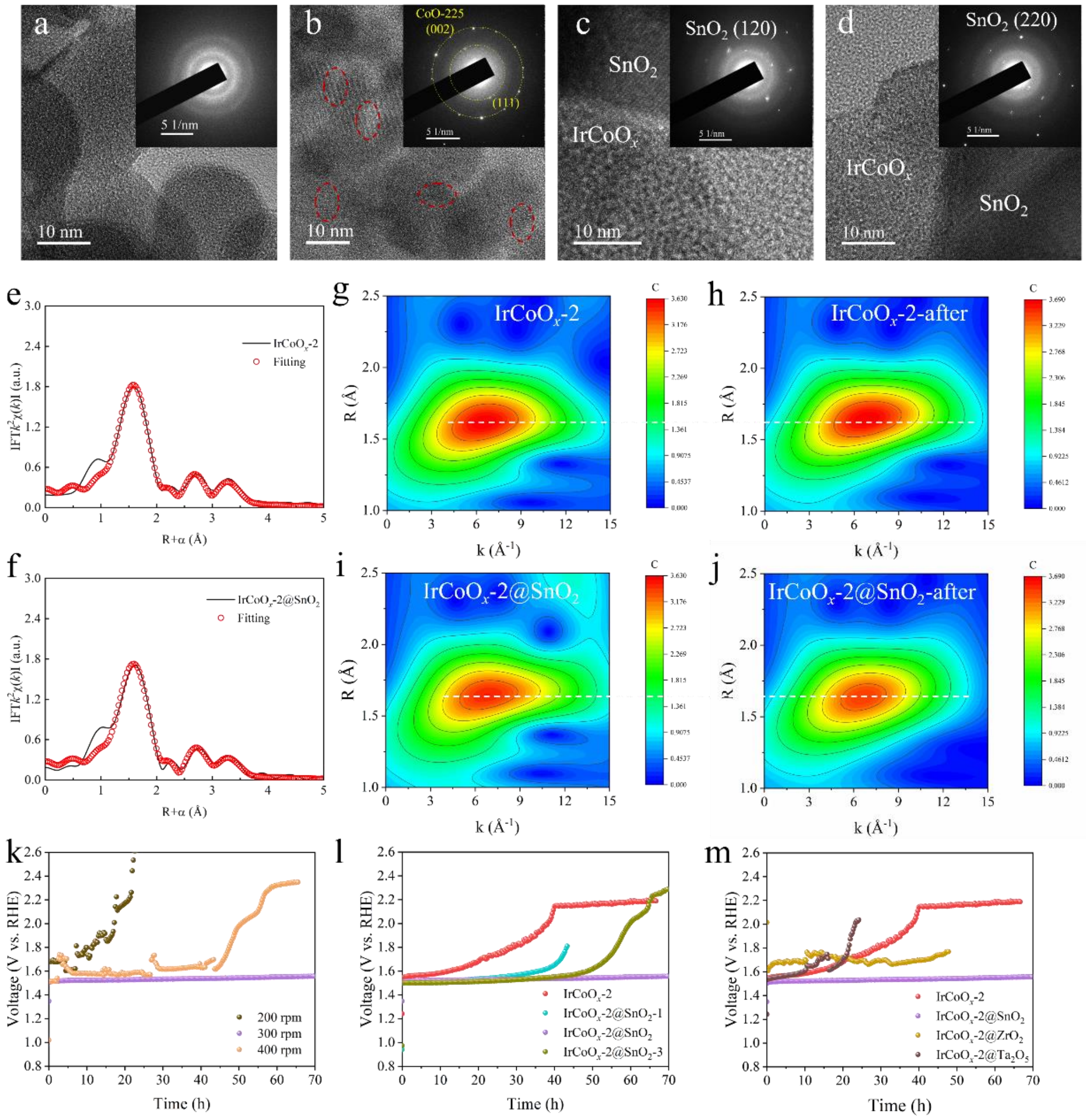

Fig. 4 Exploration of the role of SnO_2 in the composite catalyst. The TEM images of (a) $IrCoO_x$-2, (b) $IrCoO_x$-2-after, (c) $IrCoO_x$-2@SnO_2 and (d) $IrCoO_x$-2@SnO_2-after. (Insets: the corresponding SAED images). EXAFS fitting results of (e) $IrCoO_x$-2 and (f) $IrCoO_x$-2@SnO_2 catalysts. The corresponding wavelet transform (WT) patterns for (g) $IrCoO_x$-2, (h) $IrCoO_x$-2-after, (i) $IrCoO_x$-2@SnO_2 and (j) $IrCoO_x$-2@SnO_2-after. The chronopotentiometry curves at 10 mA cm^{-2} for as-prepared $IrCoO_x$-2@SnO_2 catalysts with (k) various ball milling rates and (l) varied introduction amounts of SnO_2. (m) The chronopotentiometry curves at 10 mA cm^{-2} for $IrCoO_x$-2, $IrCoO_x$-2@SnO_2, $IrCoO_x$-2@ZrO_2 and $IrCoO_x$-2@Ta_2O_5 catalysts.

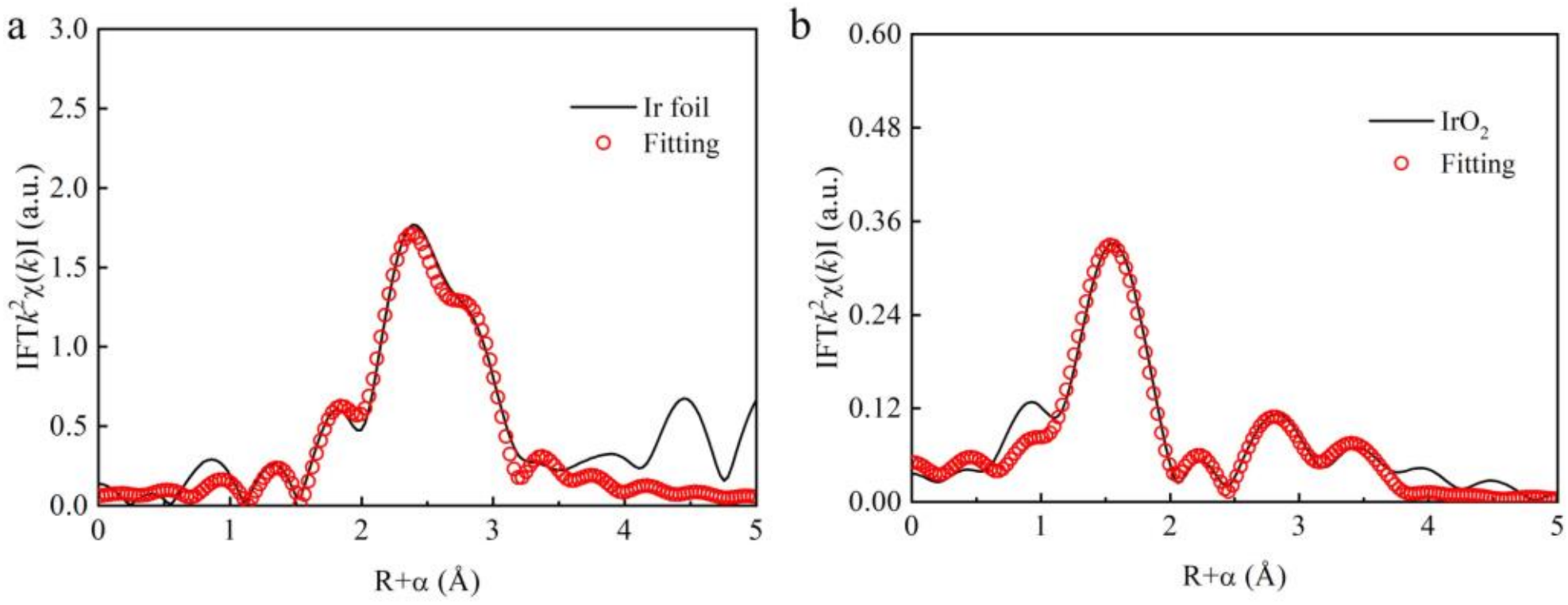

Fig. S41. EXAFS fitting results of (a) Ir foil and (b) commercial IrO_2 samples.

Table S8. EXAFS fitting parameters at the Ir L^3-edge for various samples.

Sample	Shell	CN[a]	R(Å)[b]	σ^2(Å^2)[c]	ΔE_0(eV)[d]	R factor
Ir foil	Ir-Ir	12*	2.70±0.01	0.0030	6.9	0.0073
IrO_2	Ir-O	6.0*	1.97±0.01	0.0028	9.3	0.0145
	Ir-Ir	6.0*	3.19±0.01	0.0042	9.3	
	Ir-Ir	6.0*	3.57±0.01	0.0192	4.0	
$IrCoO_x$-2	Ir-O	5.9±0.3	1.99±0.01	0.0060	13.0	0.0158
	Ir-Co	4.8±1.0	3.07±0.01	0.0187	8.4	
	Ir-Co	5.8±1.2	3.70±0.01	0.0140	-2.0	
$IrCoO_x$-2@SnO_2	Ir-O	5.7±0.3	2.00±0.01	0.0059	12.4	0.0173
	Ir-Co	3.9±0.8	3.10±0.01	0.0160	8.8	
	Ir-Co	5.8±1.2	3.70±0.01	0.0152	-2.5	
$IrCoO_x$-2-after	Ir-O	5.5±0.3	2.02±0.01	0.0048	13.2	0.0137
	Ir-Co	4.2±0.8	3.08±0.01	0.0169	7.1	
	Ir-Co	7.1±1.4	3.72±0.01	0.0166	-3.4	
$IrCoO_x$-2@SnO_2-after	Ir-O	5.5±0.3	2.02±0.01	0.0054	12.1	0.0156
	Ir-Co	2.5±0.5	3.08±0.01	0.0123	5.1	
	Ir-Co	3.4±0.7	3.72±0.01	0.0100	-3.2	

Note: (a) CN, coordination number; (b) R, distance between absorber and backscatter atoms; (c) σ^2, Debye-Waller factor to account for both thermal and structural disorders; (d) $\Delta E(0)$, inner potential correction; R factor indicates the goodness of the fit. S_0^2 was fixed to 0.855, according to the experimental EXAFS fit of Ir foil by fixing CN as the known crystallographic value. A reasonable range of EXAFS fitting parameters: $0.700 < S_0^2 < 1.000$; CN > 0; σ^2Å^2 > 0; $|\Delta E_0| < 15$ eV; R factor < 0.02.

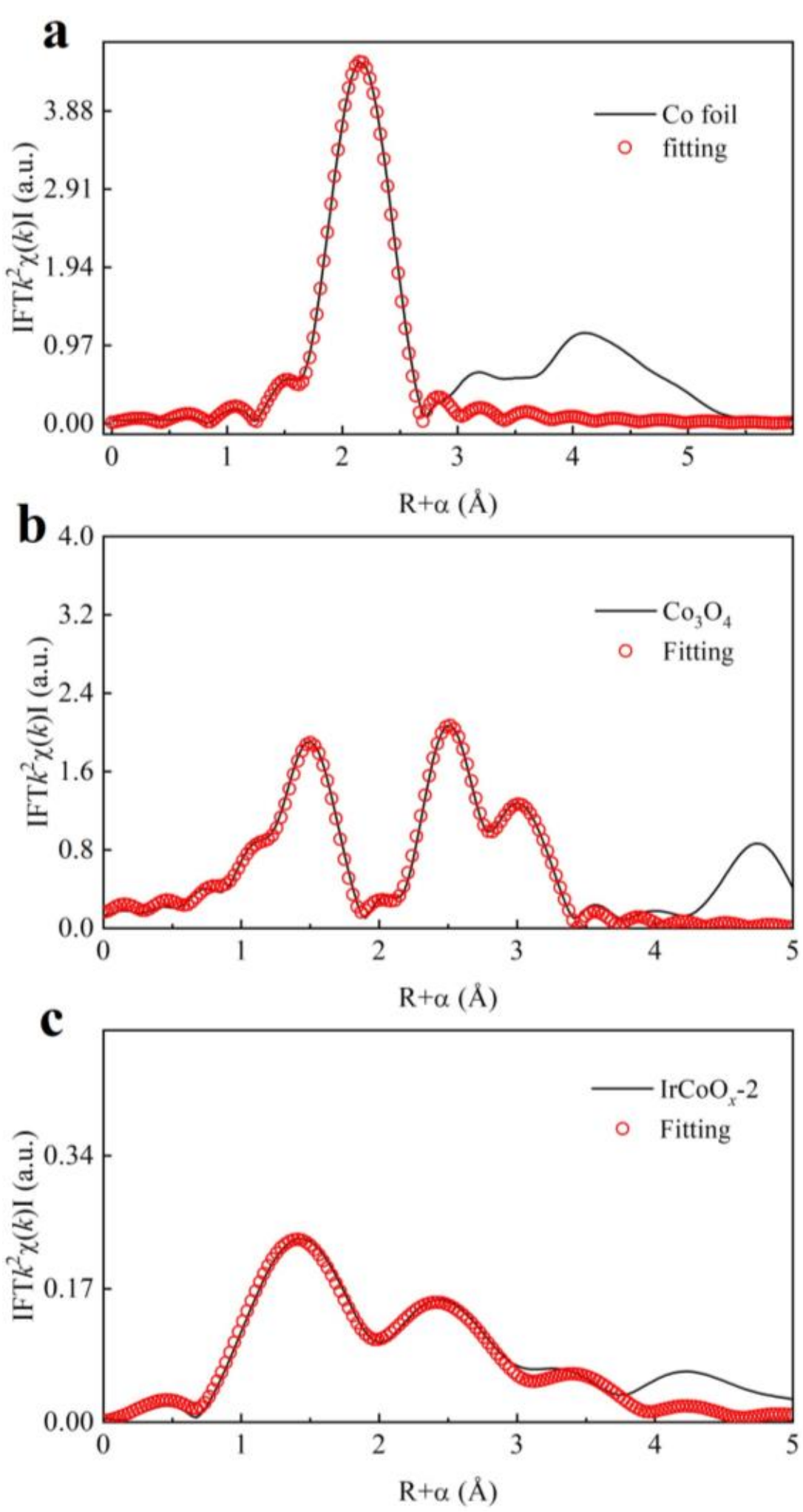

Fig. S42. EXAFS fitting results of (a) Co foil, (b) commercial Co_3O_4 and (c) $IrCoO_x$-2 samples.

Table S9. EXAFS fitting parameters at the Co K-edge for various samples.

Sample	Shell	CN[a]	R(Å)[b]	σ^2(Å^2)[c]	ΔE_0(eV)[d]	R factor
Co foil	Co-Co	12*	2.49±0.01	0.0059	5.8	0.0023
Co_3O_4	Co-O	5.5*	1.92±0.01	0.0042	4.5	0.0133
	Co-Co	6.0*	2.86±0.01	0.0065	-2.2	
	Co-Co	6.0*	3.36±0.01	0.0057	-2.8	
$IrCoO_x$-2	Co-O	0.8±0.2	1.94±0.01	0.0030	5.6	0.0095
	Co-Co	3.3±0.7	2.88±0.01	0.0030	0.2	
	Co-Ir	1.1±0.2	3.10±0.01	0.0196	1.9	

Note: (a) CN, coordination number; (b) R, distance between absorber and backscatter atoms; (c) σ^2, Debye-Waller factor to account for both thermal and structural disorders; (d) ΔE(0), inner potential correction; R factor indicates the goodness of the fit. S_0^2 was

fixed to 0.855, according to the experimental EXAFS fit of Ir foil by fixing CN as the known crystallographic value. A reasonable range of EXAFS fitting parameters: 0.700 < S_0^2 < 1.000; CN > 0; $\sigma^2 Å^2$ > 0; $|\Delta E_0|$ < 15 eV; R factor < 0.02.

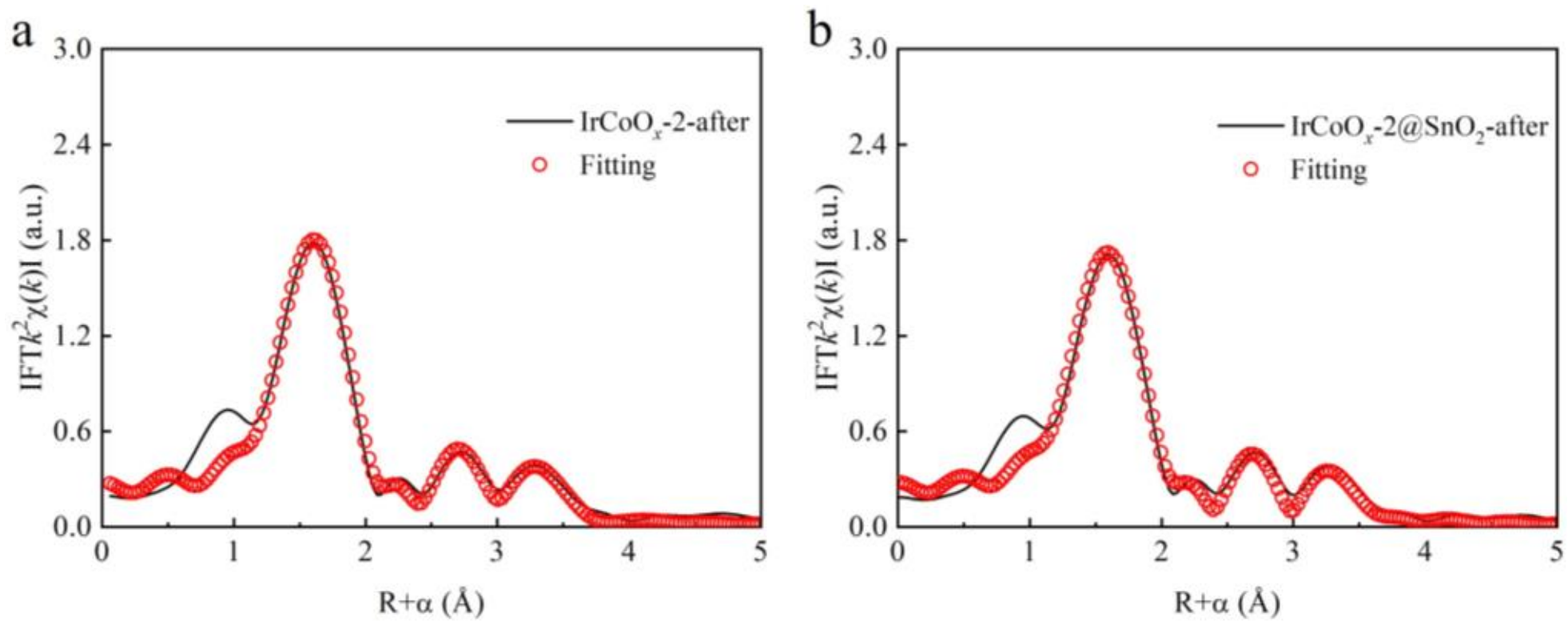

Fig. S43. EXAFS fitting results of (a) IrCoO$_x$-2-after and (b) IrCoO$_x$-2@SnO_2-after samples.

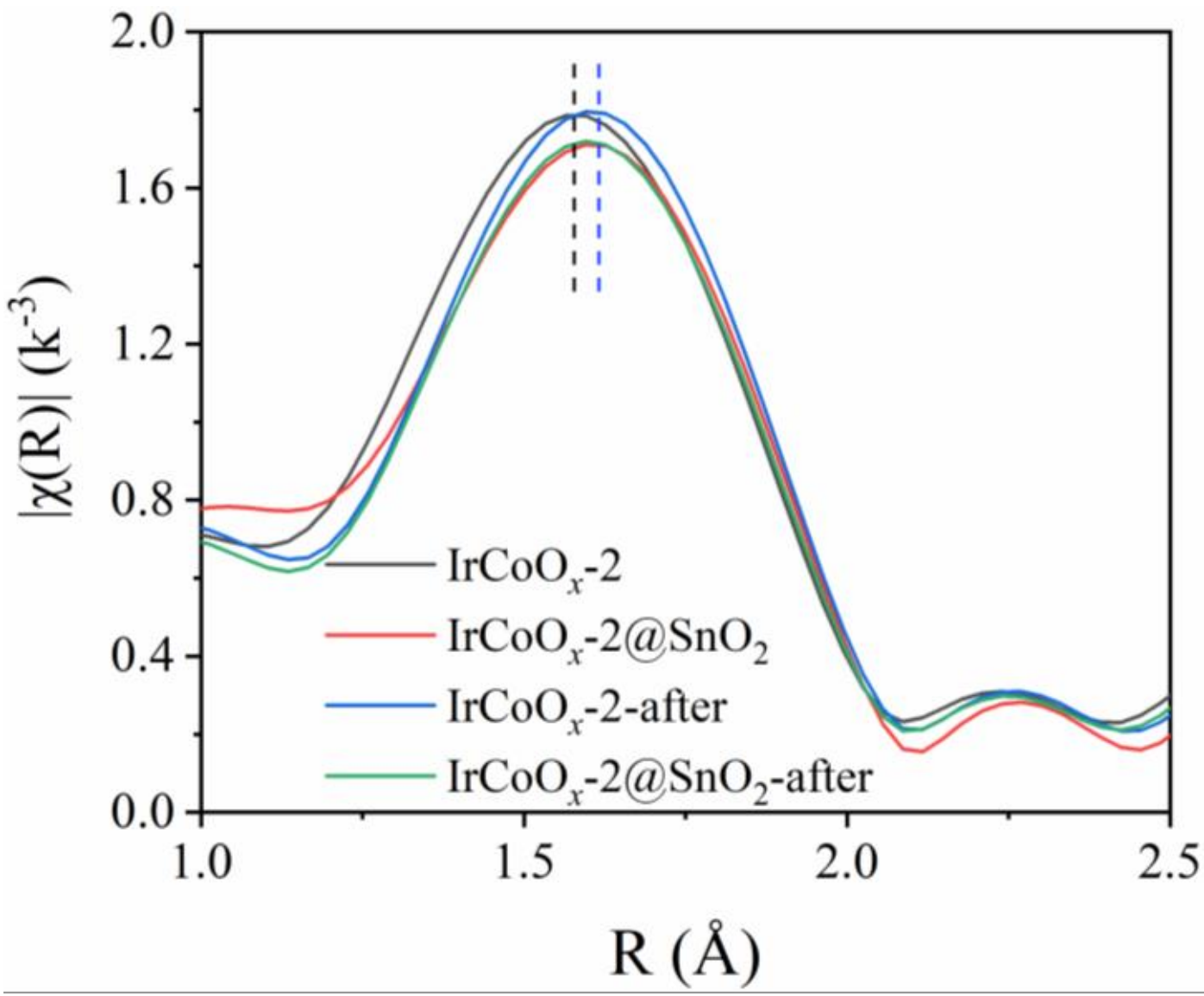

Fig. S44. Comparison of Ir L_3-edge EXAFS spectra for IrCoO$_x$-2@SnO_2 and IrCoO$_x$-2 before and after the OER stability test.

Regarding your concern about the bond length change for IrCoO$_x$-2 and IrCoO$_x$-2@SnO_2, we acknowledge that the fitted numerical difference is indeed minor. However, we would like to emphasize that, in addition to bond length, there were also notable changes in the coordination numbers of Ir-O bond ([IrCoO$_x$-2]: 5.9±0.3 → 5.5±0.3 and [IrCoO$_x$-2@SnO_2]: 5.7±0.3 → 5.5±0.3). Therefore, by combining the

differences of changes for both coordination numbers and bond length, we can reasonably conclude that the introduction of SnO_2 is an effective strategy for maintaining the paracrystalline structure of $IrCoO_x$-2. Moreover, the distinct difference in the Ir-O signal shift observed in the wavelet transform patterns further supports the stabilizing role of SnO_2. We have supplemented the discussions in the revised manuscript, which reads: "For the $IrCoO_x$-2 catalyst, there are obvious change for the coordination number (5.9±0.3 → 5.5±0.3) and bond length (1.99±0.01 → 2.02±0.01) after OER, differing from those of $IrCoO_x$-2@SnO_2. Thus, we can reasonably conclude that the introduction of SnO_2 is an effective strategy for maintaining the paracrystalline structure of $IrCoO_x$-2." (Page 15, Line 15-19 in the revised manuscript)

5. Regarding 14: From the explanation given in the text and the rebuttal letter, the wavelet transform shows a shift of the R value. The problem with this explanation is that a simple shift in R-value should be more clearly visible in the FT-EXAFS, in which one can barely see any difference by eye. So, the clearly visible shift in the wavelet transform could only be explained based on the k-space dependence, which should be implemented in the explanation (which should not only be given in the rebuttal letter).

Response: Thank you for your careful review. Firstly, in response to the concern of k-space dependence, we would like to clarify that we have consistently employed a k-space weighing of 2 for all samples in the wavelet transform analysis. The subtle visual difference observed in the FT-EXAFS spectra is primarily due to the broad range of the R-value coordinates, which tends to obscure the detailed variations. To address this, we have adjusted the FT-EXAFS plots to the same R-value range as that used in wavelet transform patterns. As shown in the newly added **Fig. S44** (see below), a clear shift in the R-value is now readily discernible for $IrCoO_x$-2 and $IrCoO_x$-2-after samples, consistent with the wavelet transform analysis. In contrast, the R-value remains almost unchanged for $IrCoO_x$-2@SnO_2 and $IrCoO_x$-2@SnO_2-after from FT-EXAFS and wavelet transform patterns. The corresponding discussion as well as the added new **Fig. S44** has been supplemented in the revised manuscript as follows: "Differing from the

obvious shift of Ir-O signal for $IrCoO_x$-2 after OER, there is hardly any alteration for $IrCoO_x$-2@SnO_2 after the stability test (Figs. S43-S44), providing definitive evidence for the stabilizing function of SnO_2 at the atomic level." (Page 15, Line 23-26 in the revised manuscript)

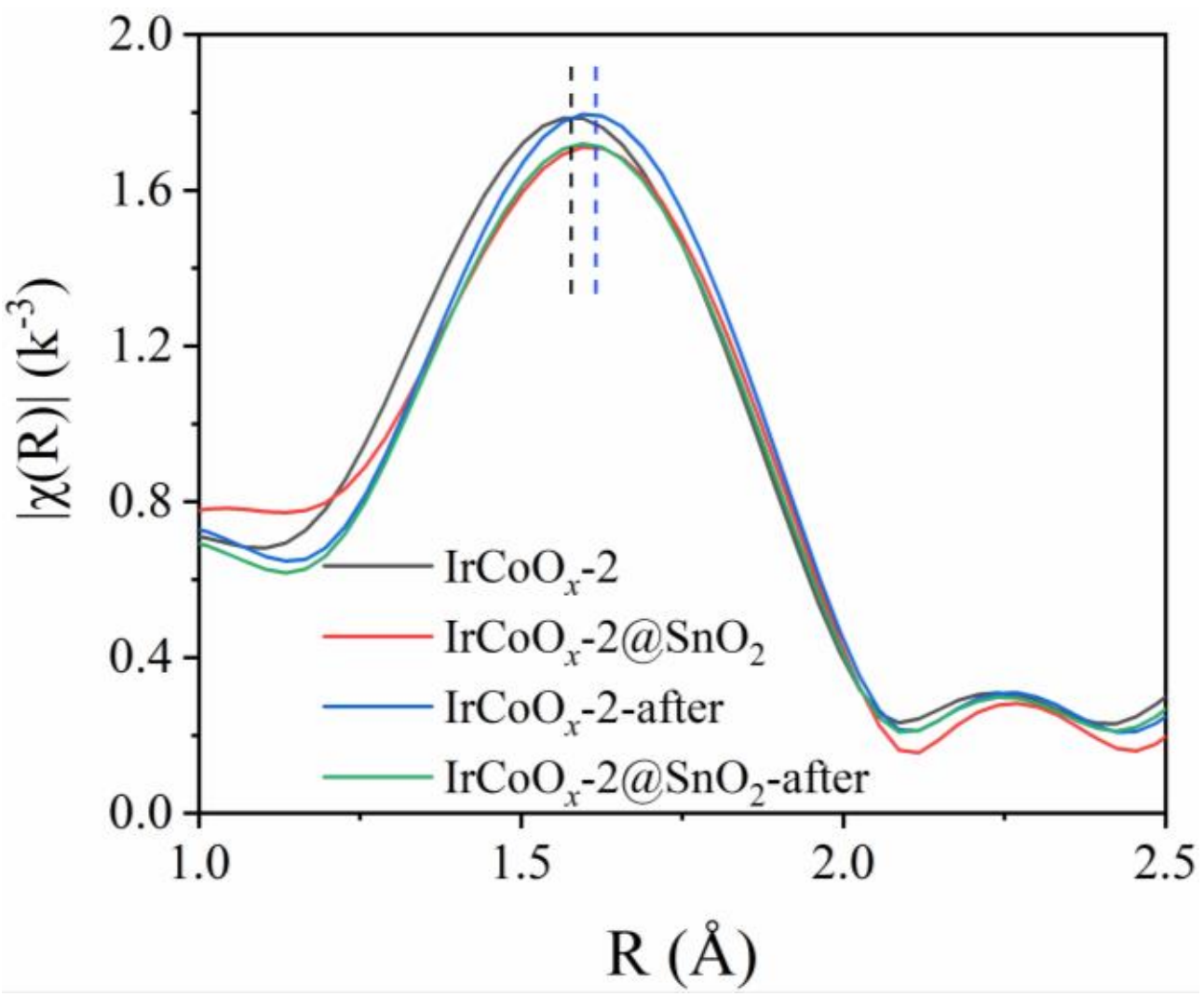

Fig. S44. Comparison of Ir L_3-edge EXAFS spectra for $IrCoO_x$-2@SnO_2 and $IrCoO_x$-2 before and after the OER stability test.

6. Regarding 15: Good to see that the authors were able to measure the Co K-edge EXAFS, too. But again, the interpretation can only be done based on EXAFS fitting as in case of the Ir L-edge. In the statement "Moreover, the presence of Co-O-Ir 294 signal in Fig. S41 also supports the substitution of Co into the IrO_x lattice." it appears dubious, that the second scattering shell is attributed only to a Co-O-Ir path. If that was the case, where are the usually predominant Co-Co and Ir-Co scattering contributions represented? What the authors would need to do to check whether the structure is substitutional would be to compare the Co-M distances extracted from fitting the FT-EXAFS at the Co K-edge with the Ir-M distances extracted from fitting the FT-EXAFS at the Ir L-edge.

Response: Thank you very much for your constructive question and valuable suggestion. We acknowledge that it is not rigorous to attribute the second scattering shell to a Co-O-Ir path without the further Co K-edge analysis of EXAFS fitting. Accordingly, we have supplemented the EXAFS fitting analysis of Co foil, Co_3O_4 and

$IrCoO_x$-2 samples. The fitted plots are presented in **Fig. S42** (see below), and all the detailed fitting parameters are summarized in **Table S9** (see below) of the Supporting Information.

As you correctly pointed out, the predominant Co-Co and Co-Ir scattering contributions are observed at approximately 2.88 Å and 3.10 Å, respectively. More importantly, the Co-Ir distance (3.10 Å) extracted from the Co K-edge FT-EXAFS fitting is in well accordance with the Ir-Co distance (3.07 Å) obtained from the Ir L_3-edge EXAFS fitting, providing strong evidence for the substitutional incorporation of Co into the IrO_x lattice.

We have also supplemented the corresponding description in the revised manuscript, which reads: "Moreover, by combining the fitting results of Ir L-edge and Co K-edge EXAFS (Fig. S42 and Table S9), the presence of Co-Ir scattering contributions was confirmed, supporting the substitution of Co into the IrO_x lattice." (Page 15, Line 19-22 in the revised manuscript)

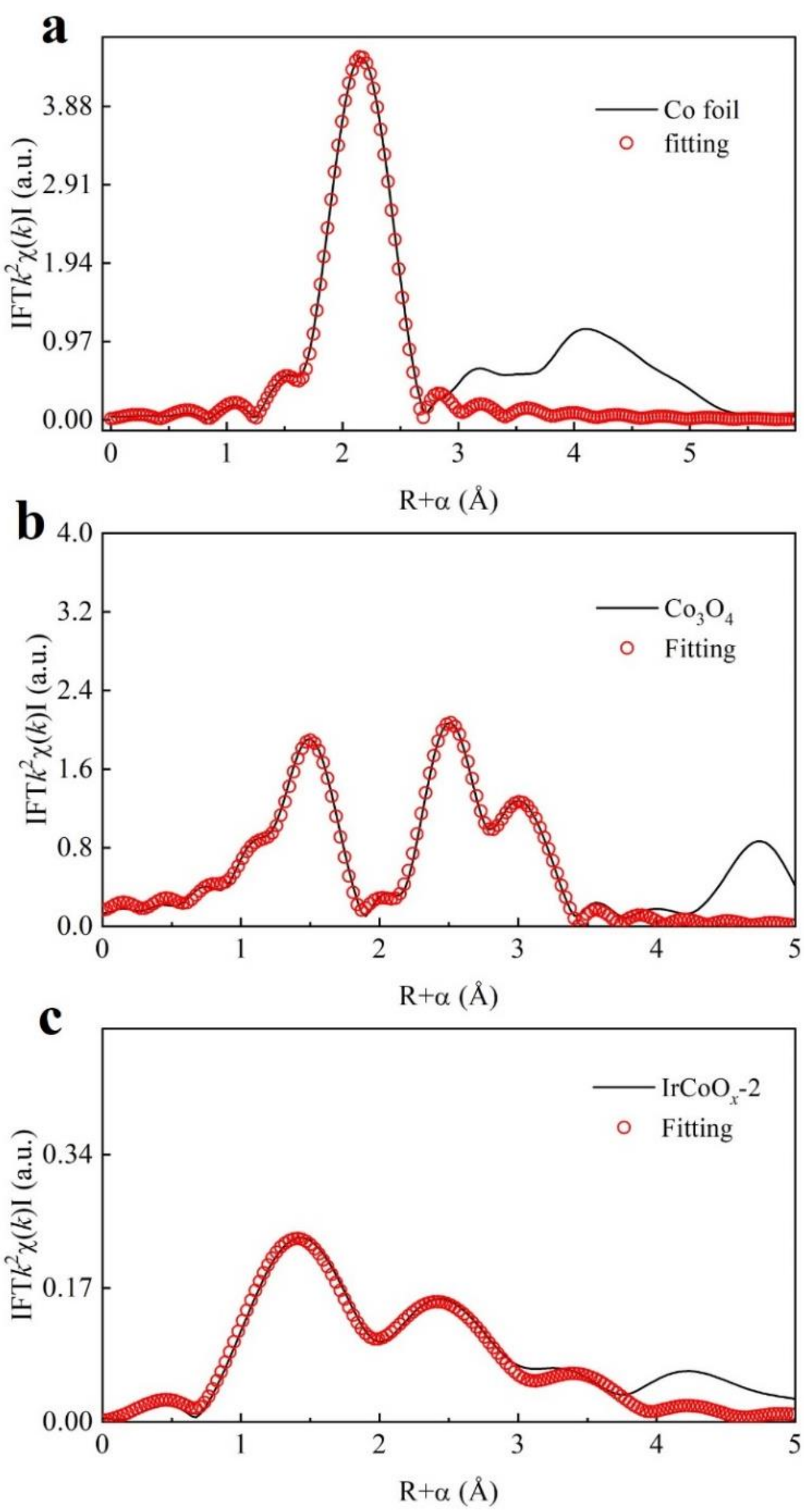

Fig. S42. EXAFS fitting results of (a) Co foil, (b) commercial Co_3O_4 and (c) $IrCoO_x$-2 samples.

Table S9. EXAFS fitting parameters at the Co K-edge for various samples.

Sample	Shell	CN[a]	R(Å)[b]	σ^2(Å^2)[c]	ΔE_0(eV)[d]	R factor
Co foil	Co-Co	12*	2.49±0.01	0.0059	5.8	0.0023
Co_3O_4	Co-O	5.5*	1.92±0.01	0.0042	4.5	0.0133
	Co-Co	6.0*	2.86±0.01	0.0065	-2.2	
	Co-Co	6.0*	3.36±0.01	0.0057	-2.8	
$IrCoO_x$-2	Co-O	0.8±0.2	1.94±0.01	0.0030	5.6	0.0095
	Co-Co	3.3±0.7	2.88±0.01	0.0030	0.2	
	Co-Ir	1.1±0.2	3.10±0.01	0.0196	1.9	

7. Regarding 19: Please implement the correlation between the MOR and the *OH coverage into the explanation in the main text to make it more comprehensible.

Response: Thanks a lot for your suggestion. We have supplemented the correlation between MOR and *OH coverage in the revised manuscript to make the underlying mechanism more comprehensible. The added text reads as follows: "Specifically, methanol is used as a molecular probe to assess the *OH surface coverage, as the methanol oxidation reaction (MOR) proceeds *via* nucleophilic attack of methanol molecules on electrophilic *OH species. Consequently, a catalyst that is less active toward MOR (indicated by a lower pH-slope) reflects lower *OH coverage on its surface." (Page 16, line 28-Page 17, Line 4 in the revised manuscript)

Point-to-point response and revisions

(Original Manuscript Number: NCOMMS-25-101304B)

Note: In the response manuscript, comments suggested by reviewers are in **black font**, our responses are in blue font, and revised parts in the text and SI are highlighted in yellow. The figures in our Manuscript, Supplementary Information and Response documents are displayed in the form of **Fig. XX**, **Fig. SXX** and **Fig. RXX**, respectively.

Responses to Reviewer 2

Comments: The authors supplied critical stability test data in the revised supporting information, and comprehensively addressed the points raised by the other reviewer.

Response: Thank you very much for your positive recommendation. We appreciate the reviewer's time and efforts in reviewing the manuscript.

Responses to Reviewer 3

Comments: In the updated version, the authors have addressed all concerns adequately and I suggest to proceed with the publication process.

Response: Thank you very much for your positive recommendation. We appreciate the reviewer's time and efforts in reviewing the manuscript.
